# Climatic niche pre-adaptation facilitated island colonization followed by budding speciation in the Madeiran ivy (*Hedera maderensis*, Araliaceae)

**DOI:** 10.3389/fpls.2022.935975

**Published:** 2022-07-25

**Authors:** Alejandro Alonso, Angélica Gallego-Narbón, Marina Coca-de-la-Iglesia, David Monjas, Nagore G. Medina, Mario Fernández-Mazuecos, Virginia Valcárcel

**Affiliations:** ^1^Departamento de Biología, Universidad Autónoma de Madrid, Madrid, Spain; ^2^Departamento de Biodiversidad y Conservación, Real Jardín Botánico (RJB-CSIC), Madrid, Spain; ^3^Centro de Investigación en Biodiversidad y Cambio Global (CIBC–UAM), Universidad Autónoma de Madrid, Madrid, Spain; ^4^Departamento de Biodiversidad, Ecología y Evolución, Facultad de Ciencias Biológicas, Universidad Complutense de Madrid, Madrid, Spain

**Keywords:** budding speciation, climatic pre-adaptation, functional traits, genotyping-by sequencing, geographic isolation, *Hedera*, island colonization, Madeira

## Abstract

The path followed by species in the colonization of remote oceanic islands ultimately depends on their phylogenetic constraints and ecological responses. In this study, we aim to evaluate the relative role of geographical and ecological forces in the origin and evolution of the Madeiran ivy (*Hedera maderensis*), a single-species endemic belonging to the western polyploid clade of *Hedera*. To determine the phylogenetic placement of *H. maderensis* within the western polyploid clade, we analyzed 40 populations (92 individuals) using genotyping-by-sequencing and including *Hedera helix* as outgroup. Climatic niche differences among the study species were evaluated using a database with 867 records representing the entire species ranges. To test species responses to climate, 13 vegetative and reproductive functional traits were examined for 70 populations (335 individuals). Phylogenomic results revealed a nested pattern with *H. maderensis* embedded within the south-western Iberian *H. iberica*. Gradual niche differentiation from the coldest and most continental populations of *H. iberica* to the warm and stable coastal population sister to *H. maderensis* parallels the geographical pattern observed in the phylogeny. Similarity in functional traits is observed for *H. maderensis* and *H. iberica*. The two species show leaves with higher specific leaf area (SLA), lower leaf dry matter content (LDMC) and thickness and fruits with lower pulp fraction than the other western polyploid species *H. hibernica*. Acquisition of a Macaronesian climatic niche and the associated functional syndrome in mainland European ivies (leaves with high SLA, and low LDMC and thickness, and fruits with less pulp content) was a key step in the colonization of Madeira by the *H. iberica*/*H. maderensis* lineage, which points to climatic pre-adaptation as key in the success of island colonization (dispersal and establishment). Once in Madeira, budding speciation was driven by geographical isolation, while ecological processes are regarded as secondary forces with a putative impact in the lack of further *in situ* diversification.

## Introduction

The path followed by species to reach oceanic islands, and then establish and speciate on them, remains a challenging open question in biogeography (Patiño et al., 2017). Achieving island colonization (including dispersal and establishment) involves the successful interaction between a mixture of ecological and evolutionary processes that are difficult to unravel (Alzate et al., 2020). The dispersal filter depends on the propagule pressure (frequency of dispersal events and number of propagules per dispersal event), which is mainly determined by the traits of the propagules and the distance from the source to the island. The environmental filter depends on the biotic and abiotic pressures during dispersal and mostly upon arrival, and it is related to pre-adaptation or easiness to adapt to new environments. Oceanic islands host the most spectacular radiations (e.g., Fernández-Mazuecos et al., 2020), but the course of evolution after island colonization is not limited to that, and also includes non-speciation and speciation without diversification. This is so because the evolutionary fate of a new island colonizer depends on an array of multiple factors such as the time elapsed since colonization, the degree of isolation from the mainland, the geographical and ecological opportunities for speciation (biotic and abiotic pressures), the propagule pressure and the phylogenetic constraints of the lineage (Stuessy et al., 2006; Takayama et al., 2015). Indeed, the high endemicity levels on oceanic islands do not only come from astonishing radiations but also from a surprisingly high proportion of lineages that speciated once on the islands and did not further diversify (single-species endemics; Patiño et al., 2014). However, the factors driving the evolutionary path of lineages that do not radiate after island colonization have been much less studied, and the ways ecological factors interact with geographical isolation and phylogenetic constraints to produce such pattern remain unclear. In this context, studying how functional traits have changed during island colonization can help unravel the role of ecological factors. Functional traits are those plant characteristics that affect individual fitness and represent ecological strategies related to the responses of plants to environment (Pérez-Harguindeguy et al., 2013). As such, they provide a unique perspective about how plants have undergone changes related to environmental factors.

Macaronesia is a keystone biogeographic region for the study of island colonization and speciation processes. It includes four major oceanic archipelagos (Azores, Madeira, Canary Islands, and Cape Verde) in the northeast Atlantic Ocean (Takhtajan, 1986) and displays a combination of iconic radiations and single-species endemics. The proximity of most Macaronesian archipelagos to the mainland makes their patterns of colonization and speciation unusual in the context of the world’s oceanic archipelagos (Kim et al., 2008) and these patterns have deserved much attention from naturalists and scientists since Humboldt visited Tenerife in 1799 (Gebauer, 2014). Madeira is the second island of Macaronesia after Tenerife (Canary Islands) in terms of vascular plant richness (1,136 species, including 94 endemics; Borges et al., 2008). Yet, it is relatively small and geologically recent, and it is located at an intermediate distance from the mainland in comparison to other Macaronesian islands. Thus, the evolutionary history of Madeiran plants deserves much attention.

The Madeiran ivy (*Hedera maderensis* K. Koch ex A. Rutherford) is a frequent liana element in the laurel forests (laurisilva; Capelo et al., 2005) of Madeira and a single-species endemic. Despite being one of the most representative species of the well-known laurisilva, the origin and speciation of the Madeiran ivy remain poorly understood. Previous phylogenetic studies have identified a clade including *H. maderensis* and two western European ivy species: *H. hibernica* (G. Kirchn.) Bean and *H. iberica* (McAll.) Ackerf. & J.Wen (hereafter the “western polyploid clade”). The three species show robust morphological and cytogenetic differences (Vargas et al., 1999; Valcárcel and Vargas, 2010). On the one hand, *H. hibernica* can be easily distinguished by its unique trichomes (rotate with few rays and a medium-sized central part), which largely differ from those of *H. maderensis* and *H. iberica* (scale-like, with multiple rays and a large central part; Valcárcel and Vargas, 2010). Also, *H. hibernica* is tetraploid while *H. maderensis* and *H. iberica* are hexaploids (Vargas et al., 1999). On the other hand, *H. maderensis* and *H. iberica* are quite distinct regarding the leaves in the vegetative phase (juvenile phase). Indeed, *H. iberica* has deeply lobate juvenile leaves (>50% of the blade) with a long central lobe that is normally much longer than wide (up to three times longer), whereas *H. maderensis* has shallowly lobate vegetative leaves (<50% of the blade) with a central lobe that is wider than long (Ackerfield and Wen, 2003; Valcárcel and Vargas, 2010). Nonetheless, the species monophyly and their phylogenetic relationships remain unresolved (Vargas et al., 1999; Valcárcel et al., 2003a). While polyploidization may have played a fundamental role in the evolution of the western polyploid clade of *Hedera* as a whole (Vargas et al., 1999; Valcárcel et al., 2003a; Green et al., 2011; Escudero et al., 2014a), geographical isolation has been hypothesized as the main speciation driver in *H. maderensis* (Vargas et al., 1999). However, geographical distance cannot be an explanation by itself since ivies seem to display great dispersal capacity, as inferred from the multiple non-specialized dispersers that have been reported for ivies (Ridley, 1930; Guitián, 1987; Passinelli, 2003; Heleno et al., 2011) and the large territories that ivy species tend to occupy. This capacity for dispersal by animal vectors is not surprising since the fleshy fruits of ivies have high content of soluble carbohydrates and lipids and are produced during winter, a period when there is little food for animals. In addition, the extent to which ecological processes might have also contributed to the speciation of the Madeiran ivy has not been explored so far, even though the three species of the western polyploid clade show large differences in their distributional ranges that might be habitat-dependent. Whereas *H. hibernica* extends through a large latitudinal range (from mid-western Portugal to North England; Valcárcel et al., 2003b; Metcalfe, 2005) and a wide altitudinal range (from sea level to 1,800 m; Valcárcel, 2008), the latitudinal spread of *H. iberica* is restricted to the southwestern half of the Iberian Peninsula, where it occurs from sea level locations to 850 m altitude, and *H. maderensis* only occurs on the island of Madeira from sea level to 900 (1,450) m(Valcárcel, 2008). Indeed, while all of them are associated with oceanic conditions, the three species seem to occupy different habitats (Valcárcel et al., 2017): *H. maderensis* primarily occurs in oceanic laurel forests of Madeira, *H. iberica* in the subtropical Tertiary refugia of SW Iberia (Rutherford, 1989; Costa et al., 1997; Calleja et al., 2009) and *H. hibernica* in mild Atlantic deciduous forests (McAllister, 1990). Thus, the Madeiran ivy is an interesting study system for analyzing the evolutionary forces leading to single-species island endemics.

In this study, we aimed to analyze the main factors that drove the evolution of the lineage of *H. maderensis* and to disentangle the relative importance of geographic barriers and environmental pressures for the evolutionary path followed by this lineage on Madeira. We hypothesized that *H. maderensis* is sister to *H. iberica* because of their similarity in ploidy and morphological characters (Vargas et al., 1999; Valcárcel and Vargas, 2010). Furthermore, because of the similarity between the main habitats of the two species (Madeiran laurisilva vs. subtropical refugia in the Iberian Peninsula) we expected limited divergence in their climatic preferences and their ecological responses in the field despite their geographical distance. To test these hypotheses, we addressed four specific objectives: (1) to clarify the phylogenetic relationships among the western polyploid species of *Hedera* by using the high-throughput sequencing genotyping-by-sequencing (GBS) technique; (2) to characterize the differences and similarities in climatic niche preferences of the western polyploid species by using a geo-referenced database; (3) to analyze whether phylogenetic divergence is coupled with phenotype differences that are associated with responses to climate by studying 13 functional traits (Pérez-Harguindeguy et al., 2013); and (4) to clarify the evolutionary history and biogeographic origin of *H. maderensis*.

## Materials and methods

### Sampling

**FIGURE 1:**
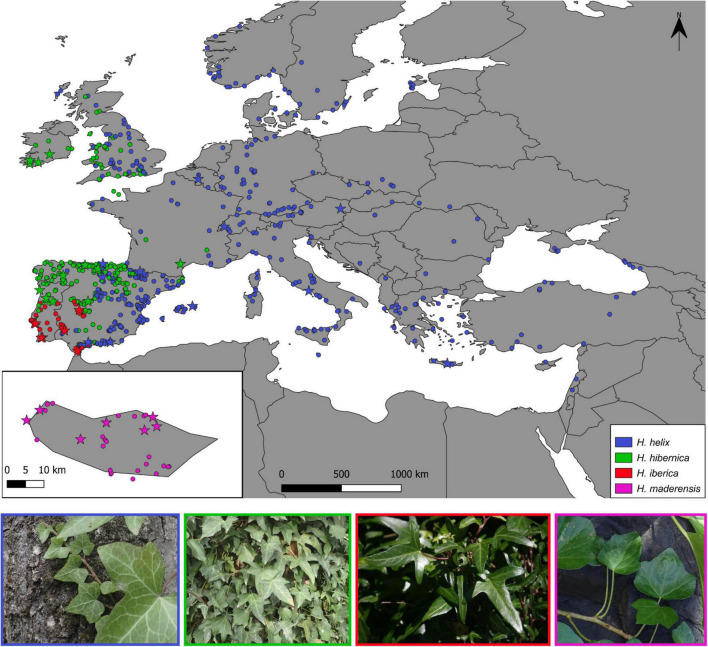
Occurrence-based map of the distribution ranges of the three species of the western polyploidy clade of *Hedera* (*H. hibernica*, *H. iberica*, *H. maderensis*) and *H. helix*. Circles indicate samples included for the climatic niche study. Stars indicate samples also included in the phylogenetic study.

For the phylogenomic study, dry leaf tissue was collected from 40 populations (92 individuals, 1-5 per population; Supplementary Table 1 and Figure 1), including 13 populations of *H. helix* (25 individuals), 11 populations of *H. hibernica* (20 individuals), 9 populations of *H. iberica* (26 individuals), and 7 populations of *H. maderensis* (21 individuals). This sampling strategy covered the whole distribution range of the three species of the western polyploid clade and a good representation of the range of the outgroup species *H. helix* (Figure 1). Special sampling effort was made to represent the distribution in the Iberian Peninsula of the two most widespread species (*H. helix* and *H. hibernica*) because this is the area where the three mainland species come into contact (Valcárcel, 2008; Valcárcel et al., 2017) and where the greatest number of plastid haplotypes have been detected (Valcárcel et al., 2017).

**FIGURE 2:**
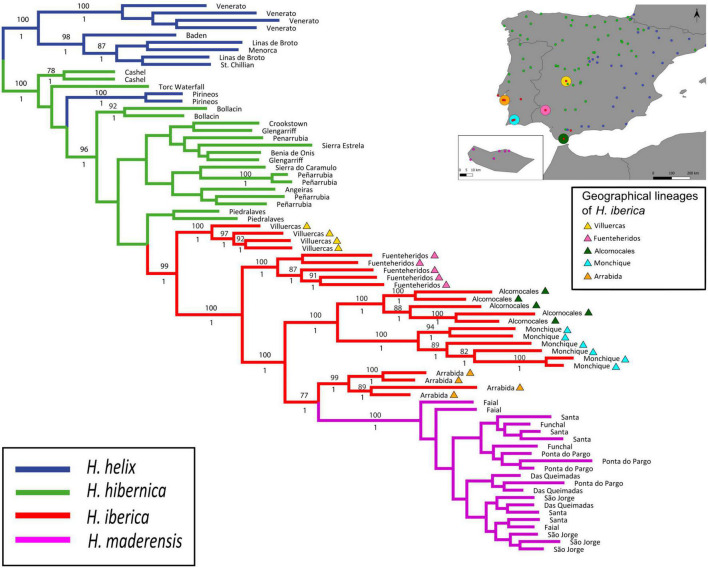
Phylogenetic tree of the western polyploid clade of *Hedera* based on phylogenomic analysis of GBS data *(c80m15p6r3)*, and using *H. helix* as outrgoup. The maximum-likelihood tree obtained in RAxML is shown, with bootstrap support values ≥75% indicated above branches. Posterior probability values obtained in the Bayesian analysis in ExaBayes are shown below branches. The inset shows a map of the Iberian Peninsula and Madeira indicating sampling localities for the study of functional traits and the geographical lineages of *H. iberica*.

For the study of functional traits in the western polyploid clade, leaves and fruits were sampled from 70 populations in the Iberian Peninsula and Madeira (335 individuals; Supplementary Table 2, map in Figure 2), including 52 populations of *H. hibernica* (248 individuals), 9 populations of *H. iberica* (53 individuals), and 9 populations of *H. maderensis* (34 individuals). To account for intra-individual variability, we collected five replicates per individual. Ivies have heteroblasty with two distinct phases: the juvenile non-flowering phase (herein called “vegetative phase”) and the adult reproductive phase (herein called “reproductive phase”). The most conspicuous difference between the two phases is the type of growth, which is plagiotropic in the vegetative phase and orthotropic in the reproductive phase (Robbins, 1957; Stein and Fosket, 1969; Poethig, 1990). The vegetative phase grows as part of the forest understory (it spreads on the ground or climbs up on tree trunks or rocks) and its leaves can be considered shade leaves, whereas the reproductive phase occurs in the forest canopy and develops sun leaves (Rehm et al., 2014). Because of this, we collected leaves from both the vegetative and the reproductive phases.

### Genotyping-by-sequencing library preparation

Total genomic DNA was isolated from dry leaf tissue using a modified CTAB protocol (Doyle and Doyle, 1987; Cullings, 1992). DNA concentrations were assessed with a Qubit 3.0 Fluorometer (Invitrogen, Carlsbad, CA, United States) using the dsDNA BR Assay Kit. A GBS library was prepared using 500 ng of DNA per sample and the *Pst*I-HF restriction enzyme. We followed the protocol of Fernández-Mazuecos et al. (2018), which is based on the original GBS protocol of Elshire et al. (2011) and incorporates modifications published by Escudero et al. (2014b) and Grabowski et al. (2014). After the digestion, ligation and pooling steps, DNA fragments were PCR-amplified for 19 cycles using the NEB 2X Taq Master Mix (NEB, MA, United States) and 35 ng of starting DNA in an Eppendorf Mastercycler ep gradient S (Eppendorf, Hamburg, Germany). The amplified library was purified using AMPure XP magnetic beads (Beckman Coulter, CA, United States). Quantification and quality control of the library were carried out using a 2100 Bioanalyzer (Agilent, CA, United States). Additionally, cloning was conducted using a pGEM-T Easy Vector (Promega Biotech Ibérica, Spain), and the inserts of 10 positive colonies were PCR-amplified and sequenced using the Sanger method (Sanger et al., 1977) to confirm the correct ligation of adapters. The library was submitted to Macrogen Inc. (Seoul, South Korea) for sequencing using Illumina HiSeq 4000 (Illumina, Inc., San Diego, CA, United States) paired-end technology.

### Genotyping-by-sequencing data assembly

Genotyping-by-sequencing matrices were assembled from FASTQ files in seven steps using the ipyrad software^1^ with the methodology for *de novo* assembly (without a reference genome; Eaton, 2014; Eaton and Overcast, 2020) and treating data as single-end. Assembly parameters followed Fernández-Mazuecos et al. (2020), except where indicated. For the demultiplexing step, a maximum of two base mismatches was allowed in barcode sequences. Three different clustering threshold values (*c*: 80, 85, and 90) and two minimum taxon coverage values (*m*: 4 and 15) were used. Additionally, since the study species display three ploidy levels (2×, 4×, and 6×), we assembled the data using two alternative values (*p*: 2 and 6) for the maximum number of alleles per locus, corresponding to the maximum and minimum ploidy levels. Although the assembly of GBS data is challenging for polyploids, and particularly for a dataset with mixed ploidy levels, we adopted this pragmatic approach to assess the robustness of our results to different values of *p* applied to the whole dataset. Combination of values for these 3 parameters (*c*, *m*, and *p*) led to 12 assemblies of GBS loci.

Individuals with low locus recovery showed uncertain positions in preliminary phylogenetic analyses. To avoid the phylogenetic noise caused by these individuals, 3 matrices were prepared from each of the 12 assemblies: 2 matrices including only individuals with at least 500 and 1,000 recovered loci in preliminary assemblies (hereafter denoted as *r1* and *r2*, respectively); and a matrix including individuals with at least 1,000 loci that did not change phylogenetic position across analyses (hereafter denoted as *r3*). Therefore, a total of 36 datasets were generated, each of them denoted as *c*X*m*Y*p*Z*r*W, with X being the clustering threshold, Y the minimum taxon coverage, Z the ploidy level, and W the level of filtering of individuals (see above).

### Phylogenetic analyses

Concatenation-based maximum-likelihood (ML) phylogenies were constructed for all 36 matrices using RAxML-HPC 8.2.12 (Stamatakis, 2014) with the GTR + CAT substitution model during tree search, followed by evaluation of the final tree under the GTR + GAMMA model. The number of bootstrap replicates was determined by the bootstopping criterion (Pattengale et al., 2010). The highest bootstrap support values for major clades in RAxML analyses were obtained for the *r*3 matrices obtained from the dataset generated using a clustering threshold of 0.80, a minimum taxon coverage of 15 and a maximum number of alleles per loci of 6 (*c*80*m*15*p*6*r*3 dataset). This matrix was selected for all remaining analyses.

We conducted an additional concatenation-based analysis of the *c*80*m*15*p*6*r*3 dataset using Bayesian inference (BI), implemented in ExaBayes 1.5 (Aberer et al., 2014). The GTR + GAMMA substitution model, default priors and a parsimony starting tree were used. Two parallel MCMC runs, with two coupled chains each, were carried out for 1.2 million generations, sampled every 500 generations. Convergence of each run was assessed by evaluating trace plots and effective sample size values in Tracer 1.7 (Rambaut et al., 2018). An extended majority-rule (MR) consensus tree was calculated for each run after discarding a burn-in of 10%. Although the two runs converged to slightly different likelihood values, the resulting MR trees were virtually identical. Therefore, the two runs were combined for calculation of the final MR tree.

We also analyzed the *c*80*m*15*p*6*r*3 dataset using the coalescent-based method SVDquartets (Chifman and Kubatko, 2014) implemented in PAUP* 4.0a (Swofford, 2002). The multispecies coalescent option was used, with exhaustive quartet sampling and eight taxon partitions corresponding to *H. helix*, *H. hibernica*, *H. maderensis*, and the five disjunct populations of *H. iberica*. We ran 100 bootstrap replicates, and the resulting trees were summarized in a 50% MR consensus tree.

All phylogenetic analyses were carried out through the CIPRES Science Gateway V. 3.3.^2^

### Population genetic structure

To evaluate the genetic structure of the study group and test the correspondence between species and genetic clusters, we analyzed an unlinked single nucleotide polymorphism (SNP) matrix corresponding to the sequence matrix selected from phylogenetic analyses (*c*80*m*15*p*6*r*3, see above). Genetic PCA was performed using the Jalview 2 software (Waterhouse et al., 2009). Bayesian analysis of population structure was carried out in BAPS (Corander et al., 2008). Two BAPS analyses were conducted, one of them taxonomically unconstrained and the other taxonomically constrained. For the taxonomically unconstrained analysis, a two-step analysis was conducted to identify the total number of clusters. First, a mixture analysis was run using all individuals, which detected two clusters. Then, a further mixture analysis was conducted within each of them to discover finer genetic structure. As a result of this two-step taxonomically unconstrained approach, we detected a total of three clusters (*K* = 3), which were used as pre-defined populations in a subsequent admixture analysis including all populations together again and a minimum population size of 5, 100 iterations, 200 reference individuals from each population and 20 iterations for reference individuals. For the taxonomically constrained analysis, an admixture analysis was conducted with the same parameters and using the four species as pre-defined populations (*K* = 4).

### Climatic niche study

To characterize the climatic niches and evaluate niche shifts during speciation, the database of Coca-de-la-Iglesia et al. (M. Coca-de-la-Iglesia, UAM, Madrid, Spain, unpubl. res.) was used. This database includes 867 records from the entire geographical ranges of the three species of the western polyploid clade (254 of *H. hibernica*, 76 of *H. iberica*, and 34 of *H. maderensis*) and *H. helix* (503). In this database, species identification of the records is taxonomically certain because they were all identified by the specialist of the genus (V. Valcárcel). Climatic data were obtained from WorldClim (Hijmans et al., 2005) with a resolution of 2.5 min. The 19 bioclimatic variables were clipped in ArcGIS 10.6.1 (ESRI) using the maximum and minimum latitude and longitude coordinates in our database of occurrences. We calculated pairwise Pearson correlations among the 19 bioclimatic variables. To avoid collinearity effects, when two variables had pairwise correlations above 0.8 we excluded one of them. Subsequent analyses were done using the resulting nine variables (bio03: isothermality; bio07: temperature annual range; bio08: mean temperature of wettest quarter; bio09: mean temperature of driest quarter; bio10: mean temperature of warmest quarter; bio11: mean temperature of coldest quarter; bio15: precipitation seasonality; bio16: precipitation of wettest quarter; and bio18: precipitation of warmest quarter).

Climatic niches of the three species of the western polyploid clade plus *H. helix* were evaluated using a PCA-env approach implemented in the R package *ecospat* (Warren et al., 2008; R Core Team, 2013; Di Cola et al., 2017). PCA-env is a principal component analysis calibrated using the entire environmental space of the study area (Broennimann et al., 2012). Niche overlap for all three species pairs of the western polyploid clade was quantified by means of the *D* metric (Schoener, 1970), which was selected due to its long history of use and simplicity of application (Warren et al., 2008). *D* values range from 0 (no overlap between niches) to 1 (complete overlap between niches). The first two dimensions of the PCA were summarized in the form of violin plots using the function “geom_violin” in the *ggplot2* package (Wickham, 2016).

Niche equivalency and niche similarity tests (Warren et al., 2008; Broennimann et al., 2012) were also conducted in *ecospat* for each pair of species of the western polyploid clade. Niche equivalency tests were used to evaluate niche divergence, i.e., whether the observed niche overlap between two species (*D*) is significantly lower than a null distribution generated by randomly reallocating the occurrences of both species between their ranges (the alternative = “lower” option was used). Specifically, for each pair of species the null distribution was obtained by randomly generating 100 pseudo-replicates that kept the number of occurrences for each species constant and reallocated occurrence between them. The equivalence hypothesis was rejected when the observed *D* was below the lower limit of the 95% confidence interval of the null distribution.

Niche similarity tests were used to evaluate niche conservatism, i.e., whether the observed niche overlap between two species (*D*) is significantly greater than a null distribution obtained by allowing random shifts of the niches within the environmental space (the alternative = “greater” option was used). These tests were based on the Chi-square test of Peterson et al. (1999) with the modifications described in Warren et al. (2008). Null distributions were generated with 100 iterations, and allowing both ranges to be randomly shifted (rand.type = 1). Niche conservatism was inferred if the *D* value was above the upper limit of the 95% confidence interval of the null distribution.

R scripts for all niche analyses are available online in Supplementary Script 1.

### Functional traits

We analyzed 13 functional traits (6 vegetative traits and 7 reproductive traits) recognized as representative of ecological strategies of plants (Pérez-Harguindeguy et al., 2013). These traits have an impact on individual growth and reproduction (Violle et al., 2007) and are indicative of species responses to the environment (Pérez-Harguindeguy et al., 2013). As vegetative traits, we used specific leaf area (SLA), leaf dry matter content (LDMC) and the traits used to estimate both functional traits (leaf area, leaf dry mass and leaf fresh mass). This strategy allowed us to identify which individual traits were responsible for the changes in SLA and LDMC. SLA is the area of a fresh leaf divided by its dry mass. SLA is frequently related to higher growth rates and is linked to more productive environments. An increase in SLA can be obtained either by increasing area or by decreasing the biomass, and thus analyzing SLA and its component traits can help understand how plants have changed their phenotype. Similarly, LDMC is the oven-dry mass of a leaf divided by the water-saturated mass of the same leaf. Higher LDMC values are usually related to lower growth rates and less productive environments. Higher LDMC values can be the consequence of an increase in the dry mass or a decrease in the fresh mass. Finally, we also measured leaf thickness because it is one of the key components of SLA (it is directly related to leaf density and biomass) and plays a key role in determining the physical strength of the leaves (see Pérez-Harguindeguy et al., 2013).

The vegetative traits were evaluated using vegetative and reproductive leaves to account for intra-individual variability (Herrera, 2017) and because previous studies detected differences in functional traits between the two types of leaves (anthocyanin, Murray and Hackett, 1991). Therefore, we took 12 measures per individual (6 traits per leaf × 2 types of leaves per individual).

We also analyzed several reproductive traits including fruit fresh mass, fruit dry mass, fruit dry matter content, seed dry mass, number of seeds per fruit, total seed dry mass per fruit, and pulp dry matter content. In ivies, the dispersal unit is a fleshy fruit that is composed of the pulp and several seeds. The traits related to fruit and pulp size and biomass are linked to higher efficiency of animal-mediated dispersal, as larger fruits provide a higher reward (Edwards, 2005). Meanwhile, the traits related to seed mass and size are mostly linked to the resources invested by the plant to ensure seedling survival (Pérez-Harguindeguy et al., 2013). Since each component of the fruit can be decoupled (Wheelwright, 1993) and thus evolve independently, we decided to measure them separately.

Sampling, storing and measurement of traits were done following Pérez-Harguindeguy et al. (2013). Mass measurements are provided in grams and area in mm^2^. For fresh weight measuring, all samples were stored at 4°C in zipped plastic bags with moist paper for the 24 h following field collection to ensure samples were at full turgor. Leaf area and leaf dry mass were measured on pressed leaves after oven-drying samples for 48 h at 70°C. Scaled pictures of leaves were taken, and leaf area was calculated with Fiji software (Schindelin et al., 2012). Images were converted to 32-bit RGB and to a three-slice stack (blue, green, red), and blue images were kept because they provided the highest contrast. SLA was calculated as the ratio of leaf area to leaf dry mass (mm^2^/g). LDMC was computed as the ratio of leaf dry mass to leaf fresh mass. Leaf thickness was measured as leaf fresh mass divided by leaf area. Fruit dry mass was computed after oven-drying the samples for 48 h at 70°C. Seeds were extracted from dry fruits, the number of seeds per fruit were counted, and seeds were weighed. Total dry seed mass was calculated per fruit as the sum of the dry mass of all seeds. Fruit dry matter content was estimated as the ratio of fruit dry mass to fruit fresh mass. The pulp dry matter content was computed as the difference between fruit dry mass and total seed dry mass.

We conducted two types of analyses to explore the relationships between traits and the differences between species. First, we evaluated correlations among the 19 measures (6 vegetative traits × 2 types of leaves plus 7 reproductive traits) according to the Pearson correlation coefficient using the “cor” function in the R base package, and summarized the results in a correlation plot using the “chart.Correlation” function in the *PerformanceAnalytics* package (Supplementary Figure 1; Peterson and Carl, 2020). Second, we compared the differences between species for each trait individually, as the main aim was to have an in-depth characterization of traits changes. Since each trait reflects a different facet of the species ecological behavior (Lavorel and Garnier, 2002; Violle et al., 2007), the analysis of traits individually allows, for example, disentangling whether species that differ in leaf size also differ in other traits such as SLA or LDMC. This, in turn, is key for a nuanced interpretation of the significance of the traits. Indeed, trait-by-trait analysis is a recommended practice because it provides specific information on trait responses that can be difficult to identify when traits are aggregated (Backhaus et al., 2021). Since our trait measures had a nested design (several individuals were measured in each population), we first assessed whether it was justified to apply a mixed model with the population variable as a random factor. To do so, we compared models using each trait as response variable and species as main factor with and without population random factor (Zuur et al., 2008). Diagnostic plots were inspected to check normality and homoscedasticity (Supplementary Figure 2). Since the models under comparison are not nested (they do not have the same structure) we used “gls” for the linear model, “lme” for the mixed model, and restricted maximum likelihood to fit them. We then compared them with the “anova” function (Zuur et al., 2008). The models including the random factor were significantly better than the linear ones in all cases. Therefore, we fitted mixed models including the traits as response variables, species as a factor and populations as random factors using the “lmer” function in the *lme4* package (Bates et al., 2015). When the species effect was significant, we further compared between species using the function “emmeans” in the package *lsmeans* (Lenth, 2016). Data were summarized in the form of violin plots using the function “geom_violin” in the *ggplot2* package (Wickham, 2016).

## Results

### Genotyping-by-sequencing data assembly

Illumina sequencing yielded 514,582,774 paired-end raw reads with a length of 101 bp. The GC content was 41.83% and the percentage of bases with a quality score of Q20 was 96.62%. Numbers of filtered loci, sites (bp), SNPs, phylogenetically informative sites (PISs), and percentages of missing data varied with assembly parameters and kept individuals, as summarized in Supplementary Table 3. The dataset selected for extensive analysis (*c*80*m*15*p*6*r*3) had 10,799 filtered loci, 913,235 bp, 53,123 SNPs, 30,793 PIS, and 42.4% of missing data. In general, a higher number of loci were recovered for the most recent samples. GBS data are available in the Sequence Read Archive (NCBI) under BioProject ID PRJNA842926.

### Phylogenetic inference

Results from maximum likelihood and BI analyses of the *c*80*m*15*p*6*r*3 dataset were congruent and revealed two well supported main clades, one including most of the samples of *H. helix* (which was used to root the tree) and the other including the three species of the western polyploid clade plus one population of *H. helix* from northern Spain (100% ML-BS, 1 PP; Figure 2). Within the western polyploid clade, three samples of *H. hibernica* appeared in an early-diverging position together with the *H. helix* population. The remaining samples of *H. hibernica* were clustered with all samples *of H. iberica* and *H. maderensis* in a strongly supported clade (96% ML-BS, 1 PP). Within this clade, samples of *H. hibernica* formed a large basal grade with poorly supported relationships, whereas all samples of *H. iberica* and *H. maderensis* constituted a well-supported clade (99% ML-BS, 1 PP). *Hedera maderensis* appeared embedded within this clade as sister to the Arrabida population of *H. iberica* (77% ML-BS, 1 PP; Figure 2). Both the ML and BI phylogenies supported a strong population and geographical structure in *H. iberica* with diverging lineages corresponding to different populations located in distant geographical areas. Villuercas, the most northern and inland population of *H. iberica*, was the earliest diverging population (100% ML-BS, 1 PP; Figure 2). Fuenteheridos, the second most inland population of *H. iberica*, was also the second diverging population (100% BS, 1 PP; Figure 2). The next diverging clade included the two populations of *H. iberica* from Alcornocales and Monchique, both located along the southwestern Iberian coast (100% BS, 1 PP; Figure 2). The latter clade was sister to another clade including the Arrabida population (located on the western Iberian coastline) as sister to *H. maderensis* (Figure 2). Fully congruent population-level relationships within the *H. iberica*/*H. maderensis* clade were recovered by the coalescent-based SVDquartets analysis, with BS values ranging from 86 to 100% (Supplementary Figure 3). The same relationships were supported by most ML analyses using other datasets, but these had generally lower support values (Supplementary Figure 4).

### Population genetic structure

The first three components of the genetic PCA (Supplementary Figure 5A) accounted for 72% of the variability. Four clusters were identified, corresponding to species delimitation. The *H. maderensis* cluster was clearly isolated, while the other three clusters overlapped to some extent. In particular, the *H. hibernica* cluster was placed in an intermediate position between *H. helix* and *H. iberica*, and overlapped with these two species. The narrowest multivariate space of the genetic PCA was occupied by *H. helix*, followed by *H. hibernica*, *H. maderensis* and *H. iberica*. In the taxonomically unconstrained BAPS analysis with *K* = 3, one cluster included all individuals of *H. helix* and *H. hibernica* as well as the two inland populations of *H. iberica* (Villuercas and Fuenteheridos), another cluster contained *H. iberica* individuals from the three populations closest to the coast, and the third cluster included all the individuals of *H. maderensis*. In the taxonomically constrained BAPS analysis with *K* = 4, the four genetic groups matched species delimitation, with some significant admixture suggested, including admixture from *H. hibernica* into the Villuercas population of *H. iberica* (Supplementary Figure 5B).

### Climatic niche

**FIGURE 3:**
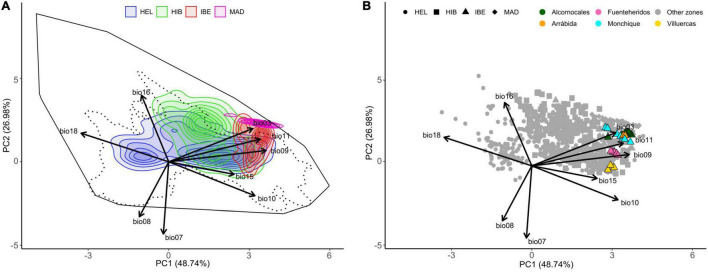
Climatic niche ordination analysis. **(A)** Climatic niche space of the three species of the western polyploidy clade of *Hedera* (*H. hibernica*, *H. iberica*, *H. maderensis*) and *H. helix* as inferred from their entire geographical ranges. The colors of the density envelopes correspond to species, with shading intensity indicating the density based on point occurrences. The solid black line delimits the multivariate space corresponding to the 100% of the available environment for all the species, and the dotted line contains the 95% of the available environment. **(B)** Climatic space occupied by the geographical lineages of *H. iberica* according to the GBS phylogeny (Figure 2). Contributions of the original WorldClim variables are provided: bio03, isothermality; bio07, temperature annual range; bio08, mean temperature of wettest quarter; bio09, mean temperature of driest quarter; bio10, mean temperature of warmest quarter; bio11, mean temperature of coldest quarter; bio15, precipitation seasonality; bio16, precipitation of wettest quarter; bio18, precipitation of warmest quarter. HEL, *Hedera helix*; HIB, *Hedera hibernica*; IBE, *Hedera iberica*; MAD, *Hedera maderensis*.

The first two principal components of the climatic PCA accounted for 77.05% of the observed variance (Figure 3). The first axis mostly accounts for temperature variables (bio09: mean temperature of the driest quarter; bio10: mean temperature of the warmest quarter; and bio11: mean temperature of the coldest quarter), precipitation during the warmest quarter (bio18) and precipitation seasonality (bio15). This axis runs from warm places with high isothermality and dry summers to cold places with low isothermality and rainy summers (right to left axis 1, Figure 3A). As a result, the first dimension discriminates the three species of the western polyploid clade (Supplementary Figure 6A). The second axis mostly accounts for annual temperature range (bio07), precipitation of wettest quarter (bio16) and mean temperature of wettest quarter (bio08). This axis runs from places with low annual temperature contrast, rainy and warm wettest season to places with high annual temperature contrast, and dry and cold wettest season (up to down axis 2, Figure 3A). The climatic niches of the study species mostly occur along the upper half of this axis (positive values of axis 2; Figure 3A). This second dimension discriminates *H. maderensis* from *H. iberica*, with the former displaying high values while the latter concentrates on lower values (Supplementary Figure 6B). In all cases, except for *H. maderensis*, species density plots show more than one peak (Figure 3A). *Hedera helix* and *H. hibernica* are the species with the broadest niches followed by *H. iberica* and *H. maderensis*. The niche of *H. helix* occupies the central part of the multivariate space and comprises a broad range of environmental conditions, including cold areas with low isothermality and rainy summers (left density peak) and warm areas with high isothermality and dry summers (right density peak). The niche of *H. hibernica* does not include the coldest areas occupied by *H. helix*, but also occurs in contrasting environments including areas with low annual temperature contrast and a relatively cold and rainy wettest season (upper density peak) and areas with higher contrast in annual temperature and with a warmer and drier wettest season (lower density peak). The climatic niche of *H. iberica* includes three density peaks, representing the warmest environments occupied by ivies in Europe and spreading across a gradient from low annual temperature contrast and relatively mild and rainy wettest season (upper density peak; Alcornocales, Monchique and Arrabida, see map in Figure 2) to more contrast in annual temperature with warm and dry wettest season (lower density peak; Fuenteheridos, see map in Figure 2). The niche of *H. maderensis* exhibits a single density peak placed at one extreme of the European climatic space of ivies, specifically in warm areas with a rainy wettest season and high isothermality (upper right part). The analysis of climatic niche overlap (Table 1) reveals a certain degree of overlap for all species pair comparisons, with the lowest value detected between *H. hibernica* and *H. maderensis* (*D* = 0.037) and the highest value between *H. iberica* and *H. maderensis* (*D* = 0.198). The climatic niches of the species are not equivalent (Table 1). Climatic niche similarity is detected between *H. maderensis* and the other two species of the western polyploid clade (Table 1).

**TABLE 1:** Pairwise climatic niche overlap statistics of the western polyploid clade of *Hedera*.

	*H. iberica*	*H. maderensis*
*H. hibernica*	*D* = 0.091	*D* = 0.037
	*p^e^*: 0.0099	*p^e^*: 0.0099
	*p^s^*: 0.25743	*p^s^*: 0.0297
*H. iberica*		*D* = 0.198
		*p^e^*: 0.0099
		*p^s^*: 0.0198

For each species pair, the first row indicates the niche overlap metric D, the second row (p^e^) is the p-value of the niche equivalency test and the third row is the p-value of the similarity test (p^s^).

### Functional traits

**FIGURE 4:**
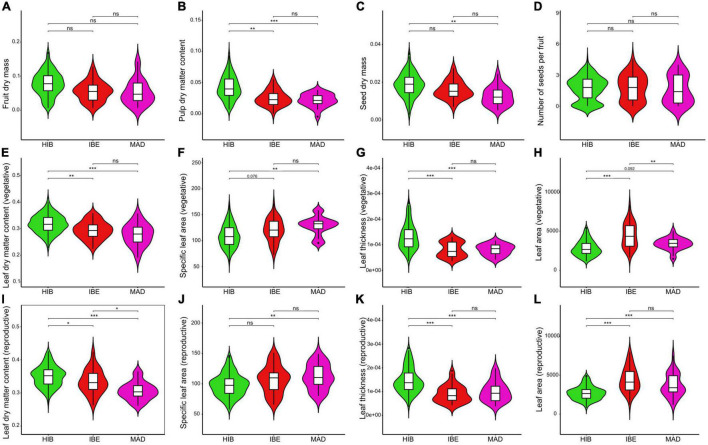
Fruit **(A-D)**, vegetative leaf **(E-H)**, and reproductive leaf **(I-L)** functional trait differences among the species of the western polyploid clade of *Hedera*: *H. hibernica* (HIB), *H. iberica* (IBE), and *H. maderensis* (MAD). The crossbar within the boxplot shows the median, and the length of the box indicates the interquartile range. Shape of the violin plot reflects the kernel density plot of data. Level of significance is shown. ***p ≤ 0.001, **p ≤ 0.01, *p ≤ 0.05, *^ns^p* > 0.05. Marginal significance is indicated with the corresponding *p*-value.

Significant differences were detected in 14 of the 19 measures analyzed when comparing the functional traits of the three species of the western polyploid clade, whereas only six traits showed significant differences between *H. iberica* and *H. maderensis* (Figure 4 and Supplementary Figure 7). On the one hand, *H. hibernica* has greater pulp dry matter content than the other two species (Figure 4B) and bigger seeds than *H. maderensis* (Figure 4C). However, no difference was detected for the remaining fruit traits (Figures 4A,D and Supplementary Figures 7A–C). Also, the leaves of *H. hibernica* (reproductive and vegetative) are smaller in size (lower values of leaf area and leaf dry mass; Figures 4H,L and Supplementary Figures 7E,G, respectively), denser (higher values of LDMC, Figures 4E,I) and thicker (higher values of leaf thickness, Figures 4G,K) than those of *H. iberica* and *H. maderensis*. On the other hand, *H. iberica* and *H. maderensis* are similar in reproductive traits (Figures 4A–D and Supplementary Figures 7A–C). As for the leaves, *H. maderensis* displays smaller leaves than *H. iberica* (leaf area, leaf dry mass and leaf fresh mass, Figure 4H and Supplementary Figures 7D–G) while no difference was detected for SLA, leaf thickness or LDMC (Figures 4E–G,J,K).

## Discussion

### Budding speciation in the western polyploid clade of *Hedera*

The application of the GBS technique has helped elucidate species-level phylogenetic relationships within the western polyploid clade of *Hedera* and has fully resolved population-level relationships in the *H. iberica*/*H. maderensis* clade. Indeed, our results clarify the evolutionary history of the group by revealing a nested phylogenetic pattern of speciation in which monophyly is only recovered for one of the three species of the clade (*H. maderensis*). The progenitor-derivative species relationship typical of nested speciation (Crawford, 2010) was robustly supported for *H. iberica* and *H. maderensis* (Figure 2). Specifically, the monophyletic *H. maderensis* is embedded within *H. iberica*, thus making the latter species paraphyletic. This result is robust to different combinations of assembly parameters in spite of uncertainties introduced by the multiple ploidy levels present in the dataset (see Supplementary Figure 4). Although reciprocal monophyly is not observed for *H. iberica* and *H. maderensis*, the two species are clearly distinct in macro- and micromorphological characters (Ackerfield and Wen, 2003; Valcárcel and Vargas, 2010), and they are also distinct genetically (Supplementary Figure 5). The lack of reciprocal monophyly can therefore be attributed to a case of budding speciation.

This type of speciation was originally proposed by Mayr (1954) as “peripatric speciation” to describe the differentiation of a new species (budded species) from a small new population that achieves reproductive isolation after dispersal from a widespread parental species. This concept essentially describes an evolutionary process in which a new species is originated through evolutionary change without the extinction of the parental species (“budding,” Mayr and Bock, 2002). In the short term, this results in a progenitor-derivative species pair scheme rather than a true sister relationship (Crawford, 2010). In fact, monophyly is not expected for the parental species soon after the speciation event because lineage sorting is not likely to be completed (Neigel and Avise, 1986), while monophyly is expected for the budded species early in its evolution because few individuals are involved in the origin, and evolutionary change is accelerated *via* founder effect and genetic drift (Rieseberg and Brouillet, 1994). In our study case, it is probable that the recovery of paraphyly for the parental species (*H. iberica*) is due to its relatively large and patchy range (68 km^2^ of area of occupancy vs. 139.708 km^2^ of extent of occurrence; M. Coca-de-la-Iglesia, UAM, Madrid, Spain, unpubl. res.) rather than a mere lack of time to coalesce, given that a middle Pliocene divergence time has been estimated for the western polyploid clade (Valcárcel et al., 2017). Indeed, strong geographical structure is detected in *H. iberica*, with diverging lineages matching populations located in distant geographical areas (Figure 2). In such cases of high levels of geographic differentiation, achieving complete lineage sorting for a majority of the genome (thus leading to monophyly in phylogenomic analyses) may be impeded even over long periods of time (Rieseberg and Brouillet, 1994; Funk and Omland, 2003). Interestingly, the area of occupancy of *H. iberica* is twice the area of *H. maderensis* (68 vs. 28 km^2^, M. Coca-de-la-Iglesia, UAM, Madrid, Spain, unpubl. res.), which is consistent with predictions of budding speciation in which an asymmetry in the ranges is expected, with widespread parental vs. restricted budded species (Barraclough and Vogler, 2000; Fitzpatrick and Turelli, 2006; Anacker and Strauss, 2014). Also, the climatic space occupied by *H. maderensis* is almost three times smaller than that of *H. iberica* and overlaps with one of its extremes (Table 1 and Figure 3A). This parallelism in the geographical range and niche breadth asymmetries is also typical of budding speciation (Grossenbacher et al., 2014) due to the positive correlation between the extent of geographical range and realized niche breadth (Slatyer et al., 2013).

In the past, budding speciation was considered a rather unusual speciation model. As molecular phylogenies have become routine in systematic studies, evidence of budding speciation has greatly increased and this phenomenon is now accepted as a common evolutionary scenario for plant speciation (Gottlieb, 2004; Crawford, 2010; Hörandl and Stuessy, 2010). However, the frequency of this mode of speciation is unevenly distributed across phylogeny and geography (Grossenbacher et al., 2014). For example, organisms with dispersal limitations or strong local adaptation are more prone to speciate following a budding-off pattern (i.e., *Mimulus*, Grossenbacher et al., 2014). In the same way, regions with heterogeneous landscape and complex geology may concentrate cases of budding speciation because isolation and local adaptation are facilitated (i.e., California Floristic Province, Anacker and Strauss, 2014). Similarly, oceanic islands like Madeira may favor budding speciation (Vanderpoorten and Long, 2006).

Our results also suggest that the *H. iberica*/*H. maderensis* clade may have evolved as another progenitor-derivative case from *H. hibernica*, but this remains an open question. This is partly due to the poor internal resolution of *H. hibernica* (Figure 2), but also to the potential introgression with *H. helix* and with inland populations of *H. iberica*, which show different degrees of genetic similarity with *H. hibernica* in taxonomically constrained and unconstrained BAPS analyses (see Supplementary Figure 5B). This introgression may have obscured to some degree phylogenetic patterns in the western polyploid clade. A more comprehensive and targeted sampling of *H. helix* and *H. hibernica* is needed to evaluate the extent and timing of the introgression and further clarify the patterns of speciation and population differentiation within the western polyploid clade of *Hedera*.

### Adaptive island colonization and non-adaptive *in situ* evolution of *Hedera maderensis*

Most studies of adaptive pressures on oceanic islands have focused on the effects of ecology on *in situ* evolution and its relevance as a driver of speciation (Emerson, 2002), while its effects during the preliminary steps of the colonization process have received comparatively little attention (Patiño et al., 2017). Besides, our knowledge on adaptive pressures is biased toward radiating lineages (Losos and Ricklefs, 2009) while we know little about the *in situ* speciation processes of non-radiating insular lineages (Patiño et al., 2017). In this study, we provide insights into the impact of ecological drivers on the colonization and speciation processes of the single-species endemic *H. maderensis*. To do so, we have combined information from phylogenetic relationships and environmental niches of *H. maderensis* and closely related species together with a set of reproductive and vegetative traits that are known to reflect responses to environmental factors. Although experimental approaches would be needed to confirm the adaptive signal herein observed, our procedure provides robust evidence to propose a fundamental role of ecological processes in the successful colonization of Madeira by ivies.

Regarding the dispersal step in the colonization of the island, monophyly of *H. maderensis* supports a single successful event of dispersal from the continent, and the close relationship between *H. maderensis* and the coastal population of *H. iberica* in Arrabida (Central Portugal) suggests an origin of dispersal in that geographic region. This route involves traveling c. 1,000 km over the Atlantic Ocean. Although no dispersal study has been conducted on any of the three species of the western polyploid clade, several non-specialized animals have been reported as effective dispersers of ivy fruits, putatively those of *H. helix* (*Silvia atricapilla, Erithacus rubecula, Garrulus glandarius, Sturnus vulgaris, Turdus iliacus, Turdus merula, T. philomelos, T. pilaris*, *T. viscivurus*; Guitián, 1987; Heleno et al., 2011; *Columba palumbus*; Ridley, 1930, Hugh A. McAllister pers. obs.; *Dendrocopos medius*, Passinelli, 2003). Among them, *T. merula* followed by *E. rubecula* are the most frequent feeding visitors of *H.* cf. *helix* in NW Spain, both displaying high percentage of ivy seeds in their feces (85.7 and 66.6%, respectively; Guitián, 1987). *Turdus viscivorus* and *S. atricapilla* also displayed high percentage of fecal ivy seeds (50 and 42.8%, respectively; Guitián, 1987). *Turdus merula* and *S. atricapilla* have also been reported as feeding on *H. azorica* individuals in Azores (Heleno et al., 2011). Given the great similarity of fruits among ivy species (Valcárcel and Vargas, 2010), similar results would be expected for our study group as well. Assuming this endozoochorous dispersal, relatively big fleshy fruits would be expected because large birds select larger fruits (Jordano, 2000), keep seeds in their guts for a longer time (Herrera, 1984; Fukui, 1996) and tend to travel longer distances (Jordano et al., 2007). Such disperser-mediated selection is not statistically supported in our study because the overall fruit mass is similar among the three species (Figure 4A). However, changes in fruit size and in the relative allocation of fruit components can be decoupled (Wheelwright, 1993). This is the case of the western polyploid clade of *Hedera*, where fruits with similar mass display significant differences in pulp and seed mass (Figures 4B–D). Relative allocation of fruit components has a major impact on the species fitness through dispersal efficiency and offspring survival (Edwards, 2005). For example, fruits with higher pulp content provide more energetic reward and thus tend to show greater dispersal efficiency (i.e., Wheelwright, 1993), and higher seed mass tends to increase chances of seedling survival (Leishman et al., 2000). In our study case, *H. maderensis* and *H. iberica* have the less pulpy fruits (Figures 4B,D) and *H. maderensis* the seeds with the least mass (Figure 4C), a pattern opposite to that expected under disperser-mediated selection. However, the selective force of dispersers can be modulated or even erased through opposed post-dispersal pressures on seeds (Alcántara and Rey, 2003; Gómez, 2004; Martínez et al., 2007). For example, smaller fruits may result in increased chances of effective dispersal if they carry small seeds that are less affected by predatory pressures (Alcántara and Rey, 2003; Gómez, 2004). We hypothesize that the decreasing pattern of pulpiness and seed size in the western polyploid clade toward *H. maderensis* is the result of a trade-off between biotic pressures before and after dispersal with impact on the successful colonization of the island.

Our findings also show that *H. maderensis* represents the last step of gradual climatic niche differentiation in *H. iberica* that brought about the pre-adaptations needed for the colonization of Madeira. Upon arrival in Madeira, these pre-adaptations and the climatic similarity between mainland and island localities contributed to population establishment, followed by speciation without further *in situ* diversification. The Madeiran ivy occupies a narrow climatic niche restricted to the wettest and least continental areas of the climatic space of *H. iberica* (Figure 3A). Rather than displaying a monotonic space, the niche of *H. iberica* shows three density peaks (Figure 3A). Interestingly, these three peaks mimic the geographic pattern observed in the phylogenetic tree (Figure 2). Indeed, the early-diverging populations in the phylogenetic tree (Villuercas and Fuenteheridos) are located far away from the coast (Figure 2) and occupy the coldest and most continental areas within the climatic preferences of *H. iberica* (Figure 3B), whereas later-diverging populations (Monchique, Alcornocales, and Arrabida) occur close to the coast (Figure 2) and occupy the warmest and least continental areas within the species preferences (Figure 3B). The latter three populations, among which the sister lineage to *H. maderensis* is included (Arrabida), occupy the warmest and most oceanic part of the climatic niche of *H. iberica*, overlapping with the niche of *H. maderensis* (Figure 3A). This climatic gradient in *H. iberica* and *H. maderensis* is part of a broader evolutionary pattern in the western polyploid clade starting from the putatively ancestral cold environments occupied by *H. hibernica* and leading to the colonization of progressively warmer areas. Plant lineages displaying evolutionary patterns of gradual climatic niche change congruent with spatially structured phylogenetic divergence and budding speciation could be more common than previously thought (i.e., Otero et al., 2022) but they have rarely been reported for Macaronesia mostly because they have not been explicitly tested.

The geographically structured variation in climatic preferences of the western polyploid clade coupled with a geographically structured phylogenetic pattern is also accompanied by a congruent pattern of divergence in functional traits. Functional divergence associated with climate has been well documented for plant radiations on oceanic islands (*Aeonium*, Jorgensen and Olesen, 2001; *Sonchus* alliance, Santiago and Kim, 2009; Hawaiian lobeliads, Givnish et al., 2009), while examples focused on non-radiating lineages like the Madeiran ivy are more limited (but see *Periploca* and *Kleinia*, García-Verdugo et al., 2019, García−Verdugo et al., 2020). In our case study, *H. hibernica* displays small leaves with low SLA and high LDMC and thickness (Figures 4E–L and Supplementary Figure 7), which is related to high investment in the structural component of leaves and indicates that the species has more “conservative” leaves (i.e., more stress tolerant, Díaz et al., 2016). In contrast, *H. iberica* and *H. maderensis* have more “acquisitive” leaves, that is, relatively big leaves with high SLA and low LDMC and thickness (Figures 4E–L and Supplementary Figure 7). These acquisitive leaves are cheap in terms of economic investment and have higher productivity and shorter lifespans (Díaz et al., 2016) which are typical of the mild climatic conditions with low abiotic stress occupied by the species. Altogether, these results point to divergent selection across habitats (Anacker and Strauss, 2014) as a determinant process in the evolution of the mainland lineages of the western polyploid clade resulting in a transition from more “conservative” to more “acquisitive” leaves (Figures 4E–L). However, the directionality of the mainland functional divergence is broken upon the establishment in the more oceanic conditions of Madeira. Indeed, the leaves of the island lineage (*H. maderensis*) are smaller in size than those of its mainland parental species (*H. iberica*, Figures 4H,L) which challenges the expected increase in leaf size from mainland to island species (García-Verdugo et al., 2010, 2019; Burns et al., 2012). These results cannot be attributed to a sampling bias in terms of altitudinal ranges or exposure to sunlight, since all sampled individuals of both *H. iberica* and *H. maderensis* were collected between sea level and 950 m in forested areas under similar sunlight conditions (the only exception was one population of *H. maderensis* in the western side of Madeira where individuals were highly exposed). Also, contrary to expectations that predict an increase in leaf traits related to higher productivity in island taxa due to the more oceanic conditions (García-Verdugo et al., 2010), the leaf economic spectrum (SLA, LDMC, and thickness) of *H. iberica* and *H. maderensis* does not show significant differences. Yet, this result is not surprising because the oceanic climatic niche of *H. maderensis* is embedded within that of *H. iberica* (Figure 3A). This suggests that the mainland populations were well adapted to the conditions found in Madeira prior to the migration event, and points to niche pre-adaptation that facilitated establishment after dispersal, and therefore the success of island colonization. Furthermore, the fact that the smaller leaves of *H. maderensis* display an economic spectrum similar to that of *H. iberica* points to a proportional reduction of the investment in structural tissues (leaf density and thickness) in *H. maderensis* (Figures 4G,K) to compensate the reduction in size and keep the photosynthesis and growth efficiency under the Madeiran climate.

Finally, the prior acquisition of adaptations to mild climates in the mainland populations and other *in situ* ecological constraints marked the evolutionary path of the lineage once on the island. Indeed, speciation of *H. maderensis* with respect to the parental *H. iberica* was probably driven by geographic isolation between island and mainland populations, while the leaf economic spectrum remained unchanged (Figure 4) likely because of the climatic similarity between the source population in coastal Iberia and Madeira. This lack of difference in the leaf economic spectrum is striking because there are major morphological differences between the two species in other features that are less affected by selective pressures, such as the morphology of leaves from the vegetative phase or the shape of their lobes (Ackerfield and Wen, 2003; Valcárcel and Vargas, 2010). The fact that no further *in situ* diversification has occurred in Madeira is intriguing, since dispersal limitations seem to boost local adaptation for fleshy fruit plants in tropical forests (Givnish et al., 2009). However, the lack of diversification in Madeira is not an isolated case in *Hedera*, since the other two Macaronesian ivy lineages are single-species endemics as well (*H. canariensis* and *H. azorica*). Interestingly, it has been shown that the Macaronesian laurel forest (where ivy species occur) is the ecosystem of this biogeographic region with the highest proportion of single-species endemics in seed plants and bryophytes (Patiño et al., 2014). In fact, there are several other single-species endemics or species-poor lineages in the woody plant biota of the Macaronesian laurel forest (Bramwell and Bramwell, 2001; Sequeira et al., 2011). This suggests some sort of ecological constraint that limits the opportunities for lineage diversification in this habitat. Indeed, the species saturation and long-term stability typical of the laurel forests have been used as a likely explanation for this pattern (Patiño et al., 2014). Since all the Macaronesian ivies are single-species endemics that occur in the laurel forest, we wonder if the predominant role of ecology in the evolution of *H. maderensis* detected here (first as an intrinsic driving force facilitating island colonization through pre-adaptation and later as an extrinsic constraint putatively limiting further *in situ* diversification) may be extended to the other Macaronesian ivy species.

Our preliminary expectations were that *H. maderensis* and *H. iberica* display similar climatic preferences and ecological responses and so we hypothesized that ecological processes had little impact on speciation of *H. maderensis*. However, our results indicate that ecology was key to the colonization of Madeira, and while almost irrelevant during the speciation process it may have been relevant in the lack of further *in situ* diversification of the lineage. To our knowledge, this is the first evidence that explicitly supports mainland pre-adaptation in a Macaronesian plant lineage, which is not totally surprising given that most of the populations of *H. iberica* occur in mainland Iberian locations that have been frequently considered climatically and floristically related to Macaronesia (Rutherford, 1989; Costa et al., 1997; Calleja et al., 2009). This mainland pre-adaptation contrasts with previous findings that show phenotypic divergence between sister plant lineages in Macaronesian islands and the mainland suggesting adaptive pressures for *in situ* evolution (García-Verdugo et al., 2019, García−Verdugo et al., 2020). A fruitful future research direction can emerge from examining the prevalence of this evolutionary pattern in single-species island endemics, and particularly in Macaronesian ivies.

## Data availability statement

The datasets presented in this study can be found in online repositories. The names of the repository/repositories and accession number(s) can be found below: Short Read Archive (SRA), reference PRJNA842926.

## Author contributions

MF-M, NM, and VV designed the study. AA and DM performed the laboratory work for the GBS library. AA and MF-M performed the phylogenetic, population structure, and climatic niche analyses. AA, AG-N, MC-D-L-I, and VV sampled the *Hedera* populations. AG-N and MC-D-L-I measured the functional traits. MC-D-L-I analyzed the functional trait data. AA, AG-N, VV, MF-M, and NM participated in the writing of the manuscript. All authors contributed to the article and approved the submitted version.

## Footnotes

1
http://ipyrad.readthedocs.io

2
http://www.phylo.org

## Supplementary material

Supplementary Figure 1 Correlogram of the 19 functional trait variables according to Pearson correlation coefficients.

Supplementary Figure 2 Diagnostic plots of the mixed models of functional trait variables.

Supplementary Figure 3 Consensus tree from the coalescent-based SVDquartets analysis.

Supplementary Figure 4 Consensus trees from the RAxML analyses of the 36 datasets.

Supplementary Figure 5 Results from population genetic structure analyses (genetic PCA and BAPS).

Supplementary Figure 6 Violin plots of the first two axes of the climatic PCA.

Supplementary Figure 7 Violin plots of the functional traits.

## References

[B1] Aberer A. J. Kobert K. Stamatakis A. (2014). ExaBayes: massively parallel Bayesian tree inference for the whole-genome era. *Mol. Biol. Evol.* 31 2553–2556. 10.1093/molbev/msu236 25135941 PMC4166930

[B2] Ackerfield J. Wen J. (2003). Evolution of *Hedera* (the ivy genus, Araliaceae): insights from chloroplast DNA data. *Int. J. Plant Sci.* 164 593–602. 10.1086/375423

[B3] Alcántara J. M. Rey P. J. (2003). Conflicting selection pressures on seed size: evolutionary ecology of fruit size in a bird-dispersed tree, *Olea europaea*. *J. Evol. Biol.* 16 1168–1176. 10.1046/j.1420-9101.2003.00618.x 14640408

[B4] Alzate A. Onstein R. E. Etienne R. S. Bonte D. (2020). The role of preadaptation, propagule pressure and competition in the colonization of new habitats. *Oikos* 129 820–829. 10.1111/oik.06871

[B5] Anacker B. L. Strauss S. Y. (2014). The geography and ecology of plant speciation: range overlap and niche divergence in sister species. *Proc. R. Soc. B* 281:20132980. 10.1098/rspb.2013.2980 24452025 PMC3906944

[B6] Backhaus L. Albert G. Cuchietti A. Nino J. M. J. Fahs N. Lisner A. (2021). Shift from trait convergence to divergence along old-field succession. *J. Veg. Sci.* 32:e12986. 10.1111/jvs.12986

[B7] Barraclough T. G. Vogler A. P. (2000). Detecting the geographical pattern of speciation from species-level phylogenies. *Am. Nat.* 155 419–434. 10.1086/303332 10753072

[B8] Bates D. Maechler M. Bolker B. Walker S. (2015). Fitting linear mixed-effects models using lme4. *J. Stat. Softw.* 67 1–48. 10.18637/jss.v067.i01

[B9] Borges P. A. V. Abreu C. Aguiar A. M. Carvalho C. (eds) (2008). *Listagem Dos Fungos, Flora e Fauna Terrestres Dos Arquipélagos da Madeira e Selvagens: A List of the Terrestrial Fungi, Flora and Fauna of Madeira and Selvagens Archipelagos.* Madeira: Direcção Regional do Ambiente da Madeira and Universidade dos Açores.

[B10] Bramwell D. Bramwell Z. I. (2001). *Flores Silvestres de las Islas Canarias.* Madrid: Editorial Rueda SL.

[B11] Broennimann O. Fitzpatrick M. C. Pearman P. B. Petitpierre B. Pelliser L. Yoccoz N. G. (2012). Measuring ecological niche overlap from occurrence and spatial environmental data. *Glob. Ecol. Biogeogr.* 21 481–497. 10.1111/j.1466-8238.2011.00698.x

[B12] Burns K. C. Herold N. Wallace B. (2012). Evolutionary size changes in plants of the south-west Pacific: insular size evolution. *Glob. Ecol. Biogeogr.* 21 819–828. 10.1111/j.1466-8238.2011.00730.x

[B13] Calleja J. A. Benito Garzón M. Sainz Ollero H. (2009). A Quaternary perspective on the conservation prospects of the Tertiary relict tree *Prunus lusitanica* L. *J.Biogeogr.* 36 487–498. 10.1111/j.1365-2699.2008.01976.x

[B14] Capelo J. Sequeira M. Jardim R. Mesquita S. Costa J. C. (2005). The vegetation of Madeira Island (Portugal). A brief overview and excursion guide. *Quercetea* 7 95–122.

[B15] Chifman J. Kubatko L. (2014). Quartet inference from SNP data under the coalescent model. *Bioinformatics* 30 3317–3324. 10.1093/bioinformatics/btu530 25104814 PMC4296144

[B16] Corander J. Marttinen P. Sirén J. Tang J. (2008). Enhanced Bayesian modelling in BAPS software for learning genetic structures of populations. *BMC Bioinform.* 9:539. 10.1186/1471-2105-9-539 19087322 PMC2629778

[B17] Costa T. Morla M. Sáinz Ollero H. (1997). *Los Bosques Ibéricos: Una Interpretación Geobotánica.* Barcelona: Planeta.

[B18] Crawford D. J. (2010). Progenitor-derivative species pairs and plant speciation. *TAXON* 59 1413–1423. 10.1002/tax.595008

[B19] Cullings K. W. (1992). Design and testing of a plant-specific PCR primer for ecological and evolutionary studies. *Mol. Ecol.* 1 233–240. 10.1111/j.1365-294X.1992.tb00182.x

[B20] Di Cola V. Broennimann O. Petitpierre B. Breiner F. T. D’Amen M. Randin C. (2017). Ecospat: an R package to support spatial analyses and modeling of species niches and distributions. *Ecography* 40 774–787. 10.111/ecog.02671

[B21] Díaz S. Kattge J. Cornelissen J. H. C. Wright I. J. Lavorel S. Dray S. (2016). The global spectrum of plant form and function. *Nature* 529 167–171. 10.1038/nature16489 26700811

[B22] Doyle J. J. Doyle J. L. (1987). A rapid DNA isolation procedure for small quantities of fresh leaf tissue. *Phytochem. Bull.* 19 11–15.

[B23] Eaton D. A. R. (2014). PyRAD: assembly of de novo RADseq loci for phylogenetic analyses. *Bioinformatics* 30 1844–1849. 10.1093/bioinformatics/btu121 24603985

[B24] Eaton D. A. R. Overcast I. (2020). Ipyrad: interactive assembly and analysis of RADseq datasets. *Bioinformatics* 36 2592–2594. 10.1093/bioinformatics/btz966 31904816

[B25] Edwards W. (2005). Within- and between-species patterns of allocation to pulp and seed in vertebrate dispersed plants. *Oikos* 110 109–114. 10.1111/j.0030-1299.2005.12846.x

[B26] Elshire R. J. Glaubitz J. C. Sun Q. Poland J. A. Kawamoto K. Buckler E. S. (2011). A robust, simple genotyping-by-sequencing (GBS) approach for high diversity species. *PLoS One* 6:e19379. 10.1371/journal.pone.0019379 21573248 PMC3087801

[B27] Emerson B. C. (2002). Evolution on oceanic islands: molecular phylogenetic approaches to understanding pattern and process. *Mol. Ecol.* 11 951–966. 10.1046/j.1365-294X.2002.01507.x 12030975

[B28] Escudero M. Martín-Bravo S. Mayrose I. Fernández-Mazuecos M. Fiz-Palacios O. Hipp A. L. (2014a). Karyotypic changes through dysploidy persist longer over evolutionary time than polyploid changes. *PLoS One* 9:e85266. 10.1371/journal.pone.0085266 24416374 PMC3887030

[B29] Escudero M. Eaton D. A. R. Hahn M. Hipp A. L. (2014b). Genotyping-by-sequencing as a tool to infer phylogeny and ancestral hybridization: a case study in Carex (Cyperaceae). *Mol. Phylogenet. Evol.* 79 359–367. 10.1016/j.ympev.2014.06.026 25010772

[B30] Fernández-Mazuecos M. Mellers G. Vigalondo B. Sáez L. Vargas P. Glover B. J. (2018). Resolving recent plant radiations: power and robustness of genotyping-by-sequencing. *Syst. Biol.* 67 250–268. 10.1093/sysbio/syx062 28973686

[B31] Fernández-Mazuecos M. Vargas P. McCauley R. A. Monjas D. Otero A. Chaves J. A. (2020). The radiation of Darwin’s giant daisies in the Galápagos Islands. *Curr. Biol.* 30 4989–4998. 10.1016/j.cub.2020.09.019 33007244

[B32] Fitzpatrick B. M. Turelli M. (2006). The geography of mammalian speciation: mixed signals from phylogenies and range maps. *Evolution* 60 601–615. 10.1111/j.0014-3820.2006.tb01140.x 16637504

[B33] Fukui A. (1996). Retention time of seeds in bird guts: costs and benefits for fruiting plants and frugivorous birds. *Plant Species Biol.* 11 141–147. 10.1111/j.1442-1984.1996.tb00139.x

[B34] Funk D. J. Omland K. E. (2003). Species-level paraphyly and polyphyly: frequency, causes, and consequences, with insights from animal mitochondrial DNA. *Annu. Rev. Ecol. Evol. Syst.* 34 397–423. 10.1146/annurev.ecolsys.34.011802.132421 19716428

[B35] García-Verdugo C. Caujapé-Castells J. Mairal M. Monroy P. (2019). How repeatable is microevolution on islands? Patterns of dispersal and colonization-related plant traits in a phylogeographical context. *Ann. Bot.* 123 557–568. 10.1093/aob/mcy191 30380011 PMC6377097

[B36] García-Verdugo C. Méndez M. Velázquez-Rosas N. Balaguer L. (2010). Contrasting patterns of morphological and physiological differentiation across insular environments: phenotypic variation and heritability of light-related traits in *Olea europaea*. *Oecologia* 164 647–655. 10.1007/s00442-010-1672-7 20532918

[B37] García-Verdugo C. Monroy P. Pugnaire F. I Jura−Morawiec J. Moreira X. Flexas J. (2020). Leaf functional traits and insular colonization: subtropical islands as a melting pot of trait diversity in a widespread plant lineage. *J. Biogeogr.* 47 2362–2376. 10.1111/jbi.13956

[B38] Gebauer A. (2014). *Alexander Von Humboldt: Su Semana en Tenerife 1799: Inicio Del Viaje a Suramérica, Su Vida y Obra.* Cadolzburg: Editorial Zech.

[B39] Givnish T. J. Millam K. C. Mast A. R. Patterson T. B. Theim T. J. Hipp A. L. (2009). Origin, adaptive radiation and diversification of the Hawaiian lobeliads (Asterales: Campanulaceae). *Proc. R. Soc. B* 276 407–416. 10.1098/rspb.2008.1204 18854299 PMC2664350

[B40] Gómez J. M. (2004). Bigger is not always better: conflicting selection pressures on seed size in *Quercus ilex*. *Evolution* 58 71–80. 10.1111/j.0014-3820.2004.tb01574.x 15058720

[B41] Gottlieb L. D. (2004). Rethinking classic examples of recent speciation in plants. *New Phytol.* 161 71–82. 10.1046/j.1469-8137.2003.00922.x

[B42] Grabowski P. P. Morris G. P. Casler M. D. Borevitz J. O. (2014). Population genomic variation reveals roles of history, adaptation and ploidy in switchgrass. *Mol. Ecol.* 23 4059–4073. 10.1111/mec.12845 24962137 PMC4142443

[B43] Green A. F. Ramsey T. S. Ramsey J. (2011). Phylogeny and biogeography of ivies (*Hedera* spp., Araliaceae), a polyploidy complex of woody wines. *Syst. Bot.* 36 1114–1127. 10.1600/036364411X605100

[B44] Grossenbacher D. L. Veloz S. D. Sexton J. P. (2014). Niche and range size patterns suggest that speciation begins in small, ecologically diverged populations in North American monkeyflowers (*Mimulus* spp.). *Evolution* 68 1270–1280. 10.1111/evo.12355 24433389

[B45] Guitián J. (1987). *Hedera helix* y los pajaros dispersantes de sus semillas: tiempo de estancia en la planta y eficiencia de movilización. *Ardeola* 34 25–35.

[B46] Heleno R. H. Ross G. Everard A. Memmott J. Ramos J. A. (2011). The role of avian “seed predators” as seed dispersers. *Ibis* 153 199–203. 10.1111/j.1474-919x.2010.01088.x

[B47] Herrera C. M. (1984). Selective pressures on fruit seediness: differential predation of fly larvae on the fruits of *Berberis hispanica*. *Oikos* 42 166–170. 10.2307/3544789

[B48] Herrera C. M. (2017). The ecology of subindividual variability in plants: patterns, processes, and prospects. *Web Ecol.* 17 51–64. 10.5194/we-17-51-2017

[B49] Hijmans R. J. Cameron S. Parra J. Jones P. G. Jarvis A. (2005). *WorldClim, version 1.3.* Berkeley: University of California.

[B50] Hörandl E. Stuessy T. F. (2010). Paraphyletic groups as natural units of biological classification. *TAXON* 59 1641–1653. 10.1002/tax.596001

[B51] Jordano P. (2000). “Fruits and frugivory,” in *Seeds: The Ecology of Regeneration in Plant Communities*, ed. Fenner M. (Wallingford: CABI), 125–166. 10.1079/9780851994321.0125

[B52] Jordano P. Garcia C. Godoy J. A. Garcia-Castano J. L. (2007). Differential contribution of frugivores to complex seed dispersal patterns. *Proc. Natl. Acad. Sci. U.S.A* 104 3278–3282. 10.1073/pnas.0606793104 17360638 PMC1805555

[B53] Jorgensen T. H. Olesen J. M. (2001). Adaptive radiation of island plants: evidence from *Aeonium* (Crassulaceae) of the Canary Islands. *Perspect. Plant Ecol. Evol. Syst.* 4 29–42. 10.1078/1433-8319-00013

[B54] Kim S.-C. McGowen M. R. Lubinsky P. Barber J. C. Mort M. E. Santos-Guerra A. (2008). Timing and tempo of early and successive adaptive radiations in Macaronesia. *PLoS One* 3:e2139. 10.1371/journal.pone.0002139 18478126 PMC2367450

[B55] Lavorel S. Garnier E. (2002). Predicting changes in community composition and ecosystem functioning from plant traits: revisiting the holy grail. *Funct. Ecol.* 16 545–556. 10.1046/j.1365-2435.2002.00664.x

[B56] Leishman M. R. Wright I. J. Moles A. T. Westoby M. (2000). “The evolutionary ecology of seed size,” in *Seeds: The Ecology of Regeneration in Plant Communities*, ed. Fenner N. (Wallingford: CABI), 31–57.

[B57] Lenth R. V. (2016). Least-squares means: the R package lsmeans. *J. Stat. Softw.* 69 1–33. 10.18637/jss.v069.i01

[B58] Losos J. B. Ricklefs R. E. (2009). Adaptation and diversification on islands. *Nature* 457 830–836. 10.1038/nature07893 19212401

[B59] Martínez I. García D. Obeso J. R. (2007). Allometric allocation in fruit and seed packaging conditions the conflict among selective pressures on seed size. *Evol. Ecol.* 21 517–533. 10.1007/s10682-006-9132-x

[B60] Mayr E. (1954). “Change of genetic environment and evolution,” in *Evolution as a Process*, eds Huxley J. Hardy A. C. (New York, NY: Collier Books), 157–180.

[B61] Mayr E. Bock W. J. (2002). Classifications and other ordering systems. *J. Zool. Syst. Evol. Res.* 40 169–194. 10.1046/j.1439-0469.2002.00211.x

[B62] McAllister H. A. (1990). *Hedera helix* L. and *H. hibernica* (Kirchner) Bean (Araliaceae) in the British Isles. *Watsonia* 18 7–15.

[B63] Metcalfe D. J. (2005). *Hedera helix* L. *J. Ecol.* 93 632–648. 10.1111/j.1365-2745.2005.01021.x

[B64] Murray J. R. Hackett W. P. (1991). Dihydroflavonol reductase activity in relation to differential anthocyanin accumulation in juvenile and mature phase *Hedera helix* L. *Plant Physiol.* 97 343–351. 10.1104/pp.97.1.343 16668393 PMC1081004

[B65] Neigel J. E. Avise J. C. (1986). “Phylogenetic relationships of mitochondrial DNA under various demographic models of speciation,” in *Evolutionary Processes and Theory*, eds Karlin S. Nevo E. (New York, NY: Academic Press), 515–534.

[B66] Otero A. Vargas P. Fernández-Mazuecos M. Jiménez-Mejías P. Valcárcel V. Villa-Machío I. (2022). A snapshot of progenitor-derivative speciation in *Iberodes* (Boraginaceae). *Mol. Ecol.* 31, 3192–3209. 10.1111/mec.16459 35390211

[B67] Passinelli G. (2003). *Dendrocopus medius* Middle spotted woodpecker. *BWP Update* 5 49–99.

[B68] Patiño J. Carine M. Fernández-Palacios J. M. Otto R. Schaefer H. Vanderpoorten A. (2014). The anagenetic world of spore-producing land plants. *New Phytol.* 201 305–311. 10.1111/nph.12480 24010958

[B69] Patiño J. Whittaker R. J. Borges P. A. V. Fernández-Palacios J. M. Ah-Peng C. Aráujo M. B. (2017). A roadmap for island biology: 50 fundamental questions after 50 years of The Theory of Island Biogeography. *J. Biogeogr.* 44 963–983. 10.1111/jbi.12986

[B70] Pattengale N. D. Alipour M. Bininda-Emonds O. R. P. Moret B. M. E. Stamatakis A. (2010). How many bootstrap replicates are necessary? *J. Comput. Biol.* 17 337–354. 10.1089/cmb.2009.0179 20377449

[B71] Pérez-Harguindeguy N. Díaz S. Garnier E. Lavorel S. Poorter H. Jaureguiberry P. (2013). New handbook for standardised measurement of plant functional traits worldwide. *Aust. J. Bot.* 61 167–234. 10.1071/BT12225

[B72] Peterson A. T. Soberón J. Sánchez-Cordero V. (1999). Conservatism of ecological niches in evolutionary time. *Science* 285 1265–1267. 10.1126/science.285.5431.1265 10455053

[B73] Peterson B. G. Carl P. (2020). *PerformanceAnalytics: Econometric Tools for Performance and Risk Analysis. R Package Version 2.0.4.*

[B74] Poethig R. S. (1990). Phase change and the regulation of shoot morphogenesis in plants. *Science* 250 923–930. 10.1126/science.250.4983.923 17746915

[B75] R Core Team (2013). *R: A Language and Environment for Statistical Computing.* Vienna: R Foundation for Statistical Computing.

[B76] Rambaut A. Drummond A. J. Xie D. Baele G. Suchard M. A. (2018). Posterior summarization in Bayesian phylogenetics using Tracer 1.7. *Syst. Biol.* 67 901–904. 10.1093/sysbio/syy032 29718447 PMC6101584

[B77] Rehm E. M. Lenz A. Hoch G. Körner C. (2014). Spring patterns of freezing resistance and photosynthesis of two leaf phenotypes of *Hedera helix*. *Basic Appl. Ecol.* 15 543–550. 10.1016/j.baae.2014.07.009

[B78] Ridley H. N. (1930). *The Dispersal of Plants Throughout the World.* Kent: Reeve & Co.

[B79] Rieseberg L. H. Brouillet L. (1994). Are many plant species paraphyletic? *TAXON* 43 21–32. 10.2307/1223457

[B80] Robbins W. J. (1957). Gibberellic acid and the reversal of adult *Hedera* to a juvenile state. *Am. J. Bot.* 44 743–746. 10.1002/j.1537-2197.1957.tb08259.x

[B81] Rutherford A. (1989). The ivies of Andalusia (Southern Spain). *Ivy J.* 15 7–17.

[B82] Sanger F. Nicklen S. Coulson R. (1977). DNA sequencing with chain-terminating inhibitors. *Proc. Natl. Acad. Sci. U.S.A.* 74 5463–5467. 10.1073/pnas.74.12.5463 271968 PMC431765

[B83] Santiago L. S. Kim S. (2009). Correlated evolution of leaf shape and physiology in the woody *Sonchus* alliance (Asteraceae: Sonchinae) in Macaronesia. *Int. J. Plant Sci.* 170 83–92. 10.1086/593044

[B84] Schindelin J. Arganda-Carreras I. Frise E. Kaynig V. Longair M. Pietzsch T. (2012). Fiji: an open-source platform for biological-image analysis. *Nat. Methods* 9 676–682. 10.1038/nmeth.2019 22743772 PMC3855844

[B85] Schoener T. W. (1970). Nonsynchronous spatial overlap of lizards in patchy habitats. *Ecology* 51 408–418. 10.2307/1935376

[B86] Sequeira M. Espírito-Santo D. Aguiar C. Capelo J. Honrado J. (2011). *Checklist da *Flora* de Portugal (Continental, Açores e Madeira).* Lisboa: Associação Lusitana de Fitossociologia.

[B87] Slatyer R. A. Hirst M. Sexton J. P. (2013). Niche breadth predicts geographical range size: a general ecological pattern. *Ecol. Lett.* 16 1104–1114. 10.1111/ele.12140 23773417

[B88] Stamatakis A. (2014). RAxML version 8: a tool for phylogenetic analysis and post-analysis of large phylogenies. *Bioinformatics* 30 1312–1313. 10.1093/bioinformatics/btu033 24451623 PMC3998144

[B89] Stein O. L. Fosket E. B. (1969). Comparative developmental anatomy of shoots of juvenile and adult *Hedera helix*. *Am. J. Bot.* 56 546–551. 10.1002/j.1537-2197.1969.tb07568.x

[B90] Stuessy T. F. Jakubowsky G. Gomez R. S. Pfosser M. Schlüter P. M. Fer T. (2006). Anagenetic evolution in island plants. *J. Biogeogr.* 33 1259–1265. 10.1111/j.1365-2699.2006.01504.x

[B91] Swofford D. L. (2002). *PAUP*: Phylogenetic Analysis Using Parsimony.* Sunderland, MA: Sinauer Associates.

[B92] Takayama K. López-Sepúlveda P. Greimler J. Crawford D. J. Peñailillo P. Baeza M. (2015). Genetic consequences of cladogenetic vs. anagenetic speciation in endemic plants of oceanic islands. *AoB Plants* 7:lv102. 10.1093/aobpla/plv102 26311732 PMC4605995

[B93] Takhtajan A. L. (1986). *Floristic Regions of the World.* Berkeley, CA: University of California Press.

[B94] Valcárcel V. (2008). *Taxonomy, Systematics and Evolution of Hedera L. (Araliaceae).* Ph.D. Thesis. Spain: Universidad Pablo de Olavide.

[B95] Valcárcel V. Fiz O. Vargas P. (2003a). Chloroplast and nuclear evidence for multiple origins of polyploids and diploids of *Hedera* (Araliaceae) in the Mediterranean basin. *Mol. Phylogenet. Evol.* 27 1–20. 10.1016/S1055-7903(02)00364-0 12679067

[B96] Valcárcel V. Rutherford A. Miller R. McAllister H. A. (2003b). “*Hedera* L,” in *Flora ibérica. Vol. X. Araliaceae-Umbelliferae*, ed. Nieto Feliner G. (Madrid: Departamento de publicaciones del CSIC), 3–12.

[B97] Valcárcel V. Guzmán B. Medina N. G. Vargas P. Wen J. (2017). Phylogenetic and paleobotanical evidence for late Miocene diversification of the Tertiary subtropical lineage of ivies (*Hedera* L., Araliaceae). *BMC Evol. Biol.* 17:146. 10.1186/s12862-017-0984-1 28641575 PMC5480257

[B98] Valcárcel V. Vargas P. (2010). Quantitative morphology and species delimitation under the general lineage concept: optimization for *Hedera* (Araliaceae). *Am. J. Bot.* 97 1555–1573. 10.3732/ajb.1000115 21616907

[B99] Vanderpoorten A. Long D. G. (2006). Budding speciation and neotropical origin of the Azorean endemic liverwort, *Leptoscyphus azoricus*. *Mol. Phylogenet. Evol.* 40 73–83. 10.1016/j.ympev.2006.02.013 16581268

[B100] Vargas P. McAllister H. A. Morton C. Jury S. L. Wilkinson M. J. (1999). Polyploid speciation in *Hedera* (Araliaceae): Phylogenetic and biogeographic insights based on chromosome counts and ITS sequences. *Plant Syst. Evol.* 219 165–179. 10.007/BF00985577

[B101] Violle C. Navas M. L. Vile D. Kazakou E. Fortunal C. Hummel I. (2007). Let the concept of trait be functional! *Oikos* 116 882–892. 10.1111/j.0030-1299-2007-15559.x

[B102] Warren D. L. Glor R. E. Turelli M. (2008). Environmental niche equivalency versus conservatism: quantitative approaches to niche evolution. *Evolution* 62 2868–2883. 10.1111/j.1558-5646.2008.00482.x 18752605

[B103] Waterhouse A. M. Procter J. B. Martin D. M. A. Clamp M. Barton G. J. (2009). Jalview Version 2–a multiple sequence alignment editor and analysis workbench. *Bioinformatics* 25 1189–1191. 10.1093/bioinformatics/btp033 19151095 PMC2672624

[B104] Wheelwright N. T. (1993). Fruit size in a tropical tree species: variation, preference by birds, and heritability. *Vegetatio* 107 163–174. 10.1007/BF00052219

[B105] Wickham H. (2016). *Ggplot2: Elegant Graphics for Data Analysis.* New York, NY: Springer-Verlag.

[B106] Zuur A. F. Ieno E. N. Walker N. J. Saveliev A. A. Smith G. M. (2008). *Mixed Effects Models and Extensions in Ecology with R.* New York, NY: Springer Science + Business Media LLC.

