# Supplementary material for: Climatic niche pre-adaptation facilitated island colonization followed by budding speciation in the Madeiran ivy (Hedera maderensis, Araliaceae)
Source: Front Plant Sci. 2022 Jul 25;13:935975. doi: 10.3389/fpls.2022.935975 (PMC9358290; doi:10.3389/fpls.2022.935975)
Supplement: Supplementary file 1 [file Data_Sheet_1.docx]

**Supplementary Data Sheet 1**. R script employed for the niche overlap analyses. Comments between hash marks (###) are informative and are not essential to run the analyses.

### Load functions and packages ###

install.packages("ecospat")

install.packages("ade4")

install.packages("adehabitatHR")

install.packages("sp")

install.packages("dplyr")

library(ecospat)

library(ade4)

library(adehabitatHR)

library(sp)

library(dplyr)

library(ggplot2)

### Preparation of datasets ###

### Load climate variable for all site of the study area 1 (column names should be x,y,X1,X2,...,Xn) ###

clim<-na.exclude(read.delim("Variables_Hedera2.txt",h=T,sep=","))

### Loading occurence sites for the species (column names should be x,y) ###

sp<-read.delim("ENSAMBLAJE5.txt",h=T,sep="\t",dec=".")

colnames(sp)[1] <- "Species" ###change the name of the column###

sp$x <- as.numeric(as.character(sp$x)) ### Change to numeric ###

sp$y <- as.numeric(as.character(sp$y))

sp$Species<-factor(sp$Species,levels = c("HIB","IBE","MAD","HEL")) ###Set the species###

### Sample environmental values for all occurences ###

occ.sp<na.exclude(ecospat.sample.envar(dfsp=sp,colspxy=2:3,colspkept=1:3,dfvar=clim,colvarxy=21:22,colvar=2:20,resolution=0.16666))

occ.sp<-cbind(occ.sp,sp[,1]) ###add species names ###

### List of species###

sp.list<-levels(occ.sp[,1])

sp.nbocc<-c()

for (i in 1:length(sp.list)){sp.nbocc<-c(sp.nbocc,length(which(occ.sp[,1] == sp.list[i])))}

###Calculate the number of occurrences per species###

sp.list<-sp.list[sp.nbocc>4] ### Remove species with less than 5 occurrences ###

nb.sp<-length(sp.list) ###Number of species###

### Selection of climatic variables to include in the analyses ###

Xvar<-c(2:20)

nvar<-length(Xvar)

### Number of iterations for the tests of equivalency and similarity ###

iterations<-100

#Resolution of the gridding of the climate space

R=100

### PCA ###

data.0<-rbind(occ.sp[,Xvar+2],clim[,Xvar]) ###Dataset for the analysis, includes all the sites of the study area and the occurrences for all the species ###

###Remove autocorrelated variables ###

var.remove.0 <- c("bio01","bio02","bio05","bio06","bio12","bio13","bio14", "bio17", "bio19")

data <- data.0 [, !(colnames(data.0) %in% var.remove.0), drop=FALSE]

w<-c(rep(0,nrow(occ.sp)),rep(1,nrow(clim))) ###Vector of weight, 0 for the occurences, 1 for the sites of the study area###

pca.cal <-dudi.pca(data, row.w = w, center = T, scale = T, scannf = F, nf = 2) # The PCA is calibrated on all the sites of the study area###

### Plot all the the species ###

sc1<- pca.cal$li[,1]

sc2<- pca.cal$li[,2]

### Selection of species ###

sp.choice<- c("HIB","HEL","MAD","IBE") ###choose the set of species for pairwise analyses###

sp.combn<-combn(sp.choice,2)

nsp<-ncol(sp.combn)

overlap<-matrix(nrow=nb.sp,ncol=nb.sp,dimnames = list(sp.choice,sp.choice)) ###Matrix to store overlap values###

equivalency<-matrix(nrow=nb.sp,ncol=nb.sp,dimnames = list(sp.choice,sp.choice)) ###Matrix to store the p-values for sp1-sp2 equivalency tests###

similarity<-matrix(nrow=nb.sp,ncol=nb.sp,dimnames = list(sp.choice,sp.choice)) ###Matrix to store the p-values for sp2 vs. sp1 similarity tests###

for(i in 1:ncol(sp.combn)) { ###For each combination of species###

row.spa<-which(occ.sp[,1] == sp.combn[1,i]) ###Rows in data corresponding to spa###

row.spb<-which(occ.sp[,1] == sp.combn[2,i]) ### Rows in data corresponding to spb###

name.spa<-sp.combn[1,i]

name.spb<-sp.combn[2,i]

### Predict the scores on the axes###

scores.clim<- pca.cal$li[(nrow(occ.sp)+1):nrow(data),] ###Scores for global climate###

scores.spa<- pca.cal$li[row.spa,] ###Scores for spa###

scores.spb<- pca.cal$li[row.spb,] ###Scores for spb###

### Calculation of occurrence density and test of niche equivalency and similarity ###

za<- ecospat.grid.clim.dyn(scores.clim,scores.clim,scores.spa,R)

zb<- ecospat.grid.clim.dyn(scores.clim,scores.clim,scores.spb,R)

###Test of niche equivalency and similarity according to Warren et al. 2008###

equ<-ecospat.niche.equivalency.test(za,zb,rep=100, alternative="lower") ### At least 100 for final analyses###

sim<-ecospat.niche.similarity.test(za,zb,rep=100,rand.type = 1, alternative="greater") ### Both za and zb are randomly shifted in the background (previous versions of ecospat implemented rand.type =2) ###

###plot###

pdf(file=paste(name.spa," x ",name.spb,".pdf",sep="")) ### Create a pdf file named from the names of the 2 species###

layout(matrix(c(1,1,2,2,1,1,2,2,3,3,4,5,3,3,6,7), 4, 4, byrow = TRUE))

ecospat.plot.niche(za,title=name.spa,name.axis1="PC1",name.axis2="PC2")

ecospat.plot.niche(zb,title=name.spb,name.axis1="PC1",name.axis2="PC2")

ecospat.plot.contrib(pca.cal$co,pca.cal$eig)

plot.new();text(0.5,0.5,paste("nicheoverlap:","\n","D=",round(as.numeric(ecospat.niche.overlap(za,zb,cor=T)[1]),3)))

plot.new()

ecospat.plot.overlap.test(equ,"D","Equivalency")

ecospat.plot.overlap.test(sim,"D","Similarity")

dev.off()

overlap[sp.combn[1,i],sp.combn[2,i]]<-ecospat.niche.overlap(za,zb,cor=T)[[1]] ###Store overlap value###

equivalency[sp.combn[1,i],sp.combn[2,i]]<-equ$p.D ###Store equivalency value###

similarity[sp.combn[1,i],sp.combn[2,i]]<-sim$p.D ###Store similarity value###}

### Niche position and breadth###

niche<-matrix(nrow=nb.sp,ncol=4,dimnames=list(sp.choice,c("pos1","breadth1","pos2","breadth2")))###Matrix to store niche characteristics###

for(i in 1:length(sp.choice)) { ###For each chosen species###

row.sp<-which(occ.sp[,1] == sp.choice[i]) ###Rows in data corresponding to sp###

name.sp<-sp.choice[i]

scores.sp<- pca.cal$li[row.sp,] ###Scores for sp###

### Calculation of occurrence density and test of niche equivalency and similarity ###

z<- ecospat.grid.clim.dyn(scores.clim,scores.clim,scores.sp,R)

c<-sample(1:(R*R),1000,prob=values(z$z.uncor)) ###Indices of 1000 random pixel weighted by density in the CP1 and CP2 PCA space ###

y=(c%/%R)+1;x=c%%R ### Coordinates of the pixels along CP1 and CP2###

CP.sim<-z$x[x] ###Scores of random pixels on CP1###

niche[i,1]<-median(CP.sim) ### Niche position on CP1###

niche[i,2]<-var(CP.sim) ### Niche breadth on CP1###

CP2.sim<-z$y[y] ### Scores of random pixels on CP2###

niche[i,3]<-median(CP2.sim) ### Niche position on CP2###

niche[i,4]<-var(CP2.sim) ### Niche breadth on CP2###}
