# Supplementary material for: Climatic niche pre-adaptation facilitated island colonization followed by budding speciation in the Madeiran ivy (Hedera maderensis, Araliaceae)
Source: Front Plant Sci. 2022 Jul 25;13:935975. doi: 10.3389/fpls.2022.935975 (PMC9358290; doi:10.3389/fpls.2022.935975)
Supplement: Supplementary file 2 [file Table_1.docx]

**Supplementary table 1.** List of plant material used for the molecular study indicating the assigned SVDQuartet. Abbreviations of the SVDQuartet groups are as follows: ALC represents the *H. iberica* quartet from S Spain; ARR represents the *H. iberica* quartet from SW Portugal; EST represents the *H. hibernica cf.* quartet from C Portugal; FUE represents the *H. iberica cf.* quartet from SW Spain; HEL the *H. helix* quartet; HIB represents the *H. hibernica* quartet; MAD represents the *H. maderensis* quartet; MON represents the *H. iberica* quartet from S Portugal; PIE represents the *H. hibernica cf.* quartet from C Spain; VIL represents the *H. iberica cf.* quartet from C Spain. All specimens are kept at MAUM herbarium in Universidad Autónoma de Madrid. When several individuals were sampled at the same location, the individual number is provided within parentheses.

| Species | Population Number  (N individuals) | Country | Locality | Voucher | Coordinates |  |  |  | SVDquartets |  |  |  |
| --- | --- | --- | --- | --- | --- | --- | --- | --- | --- | --- | --- | --- |
| *H. helix* | 1(2) | Austria | Niederösterreich,  Baden | Valcárcel, V.  43VV03(4,10) | 48.007103,  16.20197 |  |  |  | HEL |  |  |  |
| *H. helix* | 2(1) | Belgium | Hainaut,  Charleroi | Vargas, P.  114PV03(1) | 50.465588,  4.424228 |  |  |  | HEL |  |  |  |
| *H. helix* | 3(2) | France | Pyrénées-Atlantiques,  Gabas | Vargas, P.  338PV02(1,7) | 42.817114,  -0.403234 |  |  |  | HEL |  |  |  |
| *H. helix* | 4(1) | France | Occitanie,  Saint-Chinian | Vargas, P.  229PV06 | 43.426471, 2.934946 |  |  |  | HEL |  |  |  |
| *H. helix* | 5(5) | Greece | Creta,  Venerato | Martín Bravo, S.  338SMB05(1-5) | 35.195155,  25.042142 |  |  |  | HEL |  |  |  |
| *H. helix* | 6(2) | Italy | Lazio,  Gaeta | Vargas, P.  209PV01(3,9) | 41.213530, 13.576462 |  |  |  | HEL |  |  |  |
| *H. helix* | 7(1) | Spain | Almería,  Fondón | Vargas, P.  12PV05(1) | 36.948539,  -2.869739 |  |  |  | HEL |  |  |  |
| *H. helix* | 8(1) | Spain | Burgos,  Atapuerca | Vargas, P.  129PV01(1) | 42.350021,  -3.519477 |  |  |  | HEL |  |  |  |
| *H. helix* | 9(2) | Spain | Cantabria,  Santoña | Vargas, P.  125PV01(5,10) | 43.447193,  -3.477687 |  |  |  | HEL |  |  |  |
| *H. helix* | 10(2) | Spain | Huesca,  Linas de Broto | Vargas, P.  335PV02(1,9) | 42.625886,  -0.149739 |  |  |  | HEL |  |  |  |
| *H. helix* | 11(2) | Spain | Menorca,  Cala Galdana | Vargas, P.  329PV02(1,7) | 39.938619,  3.936604 |  |  |  | HEL |  |  |  |
| *H. helix* | 12(2) | Spain | Málaga,  Valle de Abdalajís | Valcárcel, V.  8VV02(2,12) | 36.939987,  -4.68868 |  |  |  | HEL |  |  |  |
| *H. helix* | 13(2) | Switzerland | Vaud,  Lausanne | Vargas, P.  132PV04(3,6) | 46.520887,  6.57852 |  |  |  | HEL |  |  |  |
| *H. hibernica* | 1(2) | Ireland | Tipperary,  Cashel | Vargas, P.  180PV10(1,5) | 52.516994,  -7.891180 |  |  |  | HIB |  |  |  |
| *H. hibernica* | 2(1) | Ireland | Cork,  Crookstown | Vargas, P.  170PV10 | 51.843063,  -8.830303 |  |  |  | HIB |  |  |  |
| *H. hibernica* | 3(2) | Ireland | Cork,  Glengarriff | Vargas, P.  177PV10(1,5) | 51.750558,  -9.552308 |  |  |  | HIB |  |  |  |
| *H. hibernica* | 4(2) | Ireland | Kerry,  Torc Waterfall | Vargas, P.  171PV10(1,5) | 52.007438,  -9.507470 |  |  |  | HIB |  |  |  |
| *H. hibernica* | 5(1) | Portugal | Lavra,  Angeiras | Fiz, O.  221OF00 | 41.267134,  -8.710806 |  |  |  | HIB |  |  |  |
| *H. hibernica cf.* | 6(1) | Portugal | Loriga,  Serra da Estrela | Fiz, O.  220OF00 | 40.322023,  -7.6132 |  |  |  | HIB |  |  |  |
| *H. hibernica* | 7(1) | Portugal | Viseu,  Serra do Caramulo | Ribeiro, P.  336PR | 40.588686,  -8.166273 |  |  |  | HIB |  |  |  |
| *H. hibernica* | 8(1) | Spain | Asturias,  Benia de Onís | Vargas, P.  412PV00 | 43.33634,  -4.96627 |  |  |  | HIB |  |  |  |
| *H. hibernica cf.* | 9(2) | Spain | Ávila,  Piedralaves | Valcárcel, V.  02VV18(1*,10) | 40.340236,  -4.70847 |  |  |  | PIE |  |  |  |
| *H. hibernica* | 10(2) | Spain | Cantabria,  Bollacín | Vargas, P.  127PV01(3,10) | 43.04324,  -3.87686 |  |  |  | HIB |  |  |  |
| *H. hibernica* | 11(5) | Spain | León,  Carucedo | Nieto Feliner, G  4615(2,4,6,7,8). | 42.49166,  -6.76109 |  |  |  | HIB |  |  |  |
| *H. iberica* | 1(1) | Portugal | Setúbal,  Arrabida | Valcárcel, V.  04VV18(1) | 38.50551,  -9.149715 |  |  |  | ARR |  |  |  |
| *H. iberica* | 2(4) | Portugal | Setúbal,  Arrabida | Valcárcel, V.  05VV18(2,4,7,9) | 38.497361,  -9.054198 |  |  |  | ARR |  |  |  |
| *H. iberica* | 3(1) | Portugal | Algarve,  Monchique | Valcárcel, V.  06VV18 | 37.342989,  -8.484002 |  |  |  | MON |  |  |  |
| *H. iberica* | 4(1) | Portugal | Algarve,  Foia peak | Valcárcel, V.  07VV18 | 37.342989,  -8.484002 |  |  |  | MON |  |  |  |
| *H. iberica* | 5(2) | Portugal | Algarve,  Monchique | Valcárcel, V.  08VV18(1,5) | 37.307021,  -8.58707 |  |  |  | MON |  |  |  |
| *H. iberica* | 6(2) | Portugal | Algarve,  Foia peak | Valcárcel, V.  09VV18(1,2) | 37.314972,  -8.591478 |  |  |  | MON |  |  |  |
| *H. iberica cf.* | 7(5) | Spain | Cáceres,  Villuercas | Valcárcel, V.  03VV18(1,2,3,4*,5*) | 39.620785,  -5.439747 |  |  |  | VIL |  |  |  |
| *H. iberica* | 8(5) | Spain | Cádiz,  Alcornocales | Valcárcel, V.  10VV18(1,3,5,6,8) | 36.22511,  -5.582609 |  |  |  | ALC |  |  |  |
| *H. iberica cf.* | 9(5) | Spain | Huelva,  Fuenteheridos | Valcárcel, V.  11VV18  (1*,3*,5*,7*,10*) | 37.90834,  -6.658308 |  |  |  | FUE |  |  |  |
| *H. maderensis* | 1(3) | Portugal | Madeira,  Santana | Valcárcel, V.  01VV08(1-3) | 32.794021,  -16.870569 |  |  |  | MAD |  |  |  |
| *H. maderensis* | 2(3) | Portugal | Madeira,  Das Queimadas | Valcárcel, V.  03VV08(1-3) | 32.782955,  -16.906232 |  |  |  | MAD |  |  |  |
| *H. maderensis* | 3(4) | Portugal | Madeira,  São Vicente | Valcárcel, V.  04VV08(1-4) | 32.80527,  -17.016722 |  |  |  | MAD |  |  |  |
| *H. maderensis* | 4(5) | Portugal | Madeira,  Achadas da Cruz | Valcárcel, V.  05VV08(2-6) | 32.841595,  -17.209293 |  |  |  | MAD |  |  |  |
| *H. maderensis* | 5(4) | Portugal | Madeira,  Ponta do Pargo | Valcárcel, V.  06VV08(1-4) | 32.811861,  -17.247745 |  |  |  | MAD |  |  |  |
| *H. maderensis* | 6(1) | Portugal | Madeira,  São Vicente | Navarro, C.  3394CN | 32.756727,  -17.091288 |  |  |  | MAD |  |  |  |
| *H. maderensis* | 7(1) | Portugal | Madeira,  Santana | Velayos, M.  9818MV | 32.821443,  -16.881931 |  |  |  | MAD |  |  |  |
