# Supplementary material for: Climatic niche pre-adaptation facilitated island colonization followed by budding speciation in the Madeiran ivy (Hedera maderensis, Araliaceae)
Source: Front Plant Sci. 2022 Jul 25;13:935975. doi: 10.3389/fpls.2022.935975 (PMC9358290; doi:10.3389/fpls.2022.935975)
Supplement: Supplementary file 3 [file Table_2.docx]

**Supplementary table 2.** List of plant material used for the functional trait study. All specimens are kept in MAUAM herbarium at Universidad Autónoma de Madrid.

| **Locality** | **Voucher** | **Coordinates** |
| --- | --- | --- |
| ***Hedera hibernica*** | | |
| Spain, Sevilla, Las Navas de la Concepción | 01VV20 (1-5) | 37.926989, -5.489869 |
| Spain, Ávila, Piedralaves | 02VV18 (1-10) | 40.340236, -4.70847 |
| Spain, Cádiz, Grazalema, Benamahoma | 02VV20 (1-5) | 36.770783, -5.478387 |
| Spain, Segovia, Sepúlveda | 03AG20 | 41.29317, -3.776106 |
| Spain, Zaragoza, Moncayo | 03AG21 (1-5) | 41.811524, -1.819827 |
| Spain, Cáceres, Guadisa | 03VV19 (1-5) | 39.375049, -5.028613 |
| Spain, Segovia, Villaseca | 04AG20 (1-5) | 41.292368, -3.842561 |
| Spain, Zaragoza, Anento | 04AG21 (1-5) | 41.071658, -1.327443 |
| Spain, Salamanca, Las Casas del Conde | 05AG20 | 40.505107, -6.045838 |
| Spain, Salamanca, Mogarraz | 06AG20 (1-5) | 40.505084, -6.045221 |
| Spain, Ávila, Navatejares | 08AG20 (1-5) | 40.328257, -5.522129 |
| Spain, Barcelona, Cerdanyola del Vallès | 08AG21 (1-5) | 41.445822, 2.129541 |
| Spain, Gerona, Calonge | 10AG21 (1-5) | 41.897548, 3.058039 |
| Spain, Ciudad Real, Fuencaliente | 10VV20 (1-5) | 38.422496, -4.297459 |
| Spain, Albacete, Tarazona de la Mancha | 11AG20 (1-5) | 39.562831, -4.584926 |
| Spain, Gerona, Castellón de Ampurias | 11AG21 (1-5) | 42.222625, 3.089219 |
| Spain, Córdoba, Trasierra | 11VV20 (1-5) | 37.940796, -4.895269 |
| Spain, Toledo, Navas del Estena | 12AG20 (1-5) | 39.488786, -4.610263 |
| Spain, Segovia province | 12VV18 (1,2) | 40.953708, -4.126171 |
| Spain, Cáceres, Guadalupe | 13AG20 (1-5) | 39.442638, -5.351212 |
| Spain, Soria, Velilla de Medinaceli | 14AG20 (1-5) | 41.157843, -2.34093 |
| Spain, Zaragoza, Huérmeda | 14AG21 (1-5) | 41.388157, -1.594476 |
| Spain, Orense, Ginzo da Limia | 14VV19 (1-5) | 42.073943, -7.707421 |
| Spain, Soria, Valdelavilla | 15AG20 (1-5) | 41.971229, -2.205101 |
| Spain, Orense, Ribadavia | 15VV19 | 42.303708, -8.130766 |
| Portugal, Braga, Caldas de Jerez | 16AG21 (1-5) | 41.760674, -8.149292 |
| Spain, Pontevedra, A Barosela | 16VV19 (1-5) | 42.565822, -8.608592 |
| Portugal, Viseu, Castro Daire | 17AG21 (1-5) | 40.920817, -7.962651 |
| Spain, A Coruña, Carnota | 17VV19 (1-5) | 42.853045, -9.070197 |
| Portugal, Guarda, Manteigas | 18AG21 (1-5) | 40.416113, -7.527149 |
| Spain, Lugo, Germade | 18VV19 (1-5) | 43.402009, -7.786319 |
| Portugal, Serra do Açor, Benfeita, Fraga da Pena waterfall | 19AG21 (1-5) | 40.22032354, -7.936101732 |
| Spain, Galicia, Lugo, Nadela, service road to San Mamede | 19VV19 (1-5) | 42.977833, -7.516703 |
| Spain, Burgos, Covarrubias | 20AG20 (1-5) | 42.052734, -3.558726 |
| Portugal, Leiría, Louriçal | 20AG21 (1-5) | 40.014277, -8.785078 |
| Spain, Asturias, Tapia de Casariego | 20VV19 (1-5) | 43.56304, -6.912319 |
| Spain, Burgos, Valle de Sedano | 21AG20 (1-5) | 42.777558, -3.769798 |
| Portugal, Leiría, Porto de Mós | 21AG21 (1-5) | 39.578466, -8.802222 |
| Spain, Asturias, Nava | 21VV19 (1-5) | 43.357293, -5.448783 |
| Spain, Asturias, Ponga | 22VV19 (1-5) | 43.189776, -5.080974 |
| Spain, León, Crémenes | 23VV19 (1-3) | 42.885759, -5.153301 |
| Spain, La Rioja, Ezcaray | 24AG21 (1-5) | 42.295444, -2.978026 |
| Spain, Zamora, Benavente | 24VV19 (1-5) | 42.007651, -5.658967 |
| Spain, Navarra, Mendaza | 25AG21 (1-5) | 42.681938, -2.268423 |
| Spain, Zamora, Ferreruela | 25VV19 (1-5) | 41.76362, -6.069816 |
| Spain, Burgos, Las Bárcenas de Cirión | 26AG21 (1-5) | 43.096063, -3.158155 |
| Spain, Palencia, Aguilar de Campoo | 26VV19 (1-5) | 42.895, -4.240833333 |
| Spain, Cantabria, Suances | 27AG21 (1-5) | 43.418241, -4.032012 |
| Spain, Gipuzkoa, Eibar | 28AG21 (1-5) | 43.178718, -2.465117 |
| Spain, Madrid, San Lorenzo de El Escorial | 28VV9 (1-5) | 40.574948, -4.152708 |
| Spain, Navarra, Igantzi | 29AG21 (1-5) | 43.217033, -1.700117 |
| Spain, Navarra, Romanzado | 30AG21 (1-5) | 42.662142, -1.222917 |
| ***Hedera iberica*** | | |
| Spain, Cáceres, Villuercas | 03VV18 (1-5) | 39.620785, -5.439747 |
| Portugal, Setúbal, Arrábida | 04VV18 | 38.50551, -9.149715 |
| Portugal, Setúbal, Arrábida | 05VV18 (1-10) | 38.497361, -9.054198 |
| Portugal, Algarve, Monchique | 08VV18 (1-5) | 37.307021, -8.58707 |
| Portugal, Algarve, Foia peak | 09VV18 (1,2) | 37.314972, -8.591478 |
| Spain, Cádiz, Alcornocales | 10VV18 (1-10) | 36.22511, -5.582609 |
| Spain, Huelva, Fuenteheridos | 11VV18 (1-10) | 37.90834, -6.658308 |
| Portugal, Lisboa, Colares | 22AG21 (1-5) | 38.771511, -9.446594 |
| Portugal, Évora, Nossa Senhora da Boa Fé | 23AG21 (1-5) | 38.55477, -8.102469 |
| ***Hedera maderensis*** | | |
| Portugal, Madeira, São Vicente | 04VV19 (1-5) | 32.729942, -17.030522 |
| Portugal, Madeira, Ponta do Pargo | 06VV19 (1-5) | 32.764073, -17.027144 |
| Portugal, Madeira, Ponta Delgada | 07VV19 (1-3) | 32.825539, -16.981865 |
| Portugal, Madeira, Achadas da Cruz | 08VV19 (1,2) | 32.840347, -17.193568 |
| Portugal, Madeira, Levada Grande | 09VV19 (1-4) | 32.847879, -17.194113 |
| Portugal, Madeira, Prazeres | 10VV19 (1-5) | 32.755229, -17.217884 |
| Portugal, Madeira, São Gonçalo | 11VV19 (1-5) | 32.673849, -16.86322 |
| Portugal, Madeira, Sao Jorge | 12VV19 | 32.827432, -16.902176 |
| Portugal, Madeira, Terras da Fora | 13VV19 (1-4) | 32.823432, -16.934766 |
