# Supplementary material for: Climatic niche pre-adaptation facilitated island colonization followed by budding speciation in the Madeiran ivy (Hedera maderensis, Araliaceae)
Source: Front Plant Sci. 2022 Jul 25;13:935975. doi: 10.3389/fpls.2022.935975 (PMC9358290; doi:10.3389/fpls.2022.935975)
Supplement: Supplementary file 4 [file Table_3.docx]

**Supplementary table 3.** Characteristics of 36 genotyping-by-sequencing datasets used in phylogenetic analyses of the western polyploid clade of *Hedera*. Each dataset is denoted as *c*X*m*Y*p*Z*r*W, with X being the clustering threshold, Y the minimum taxon coverage, Z the ploidy level, and W the level of filtering of individuals (see text). Numbers of filtered loci, sites, single nucleotide polymorphisms (SNPs), phylogenetically informative sites (PIS) and percentage of missing data for each dataset are indicated. *Dataset selected for extensive analysis.

|  | **# filtered loci** | **# sites** | **# SNPs** | **# PIS** | **% missing data** |
| --- | --- | --- | --- | --- | --- |
| ***c*80*m*4*p*2*r*1** | 22055 | 1854939 | 136025 | 51336 | 81.7 |
| ***c*80*m*4*p*2*r*2** | 21508 | 1808144 | 131711 | 49601 | 80.9 |
| ***c*80*m*4*p*2*r*3** | 20955 | 1761164 | 127993 | 48157 | 80.6 |
| ***c*80*m*4*p*6*r*1** | 30737 | 2602302 | 215125 | 94676 | 74.8 |
| ***c*80*m*4*p*6*r*2** | 30109 | 2547255 | 208530 | 91779 | 74.1 |
| ***c*80*m*4*p*6*r*3** | 29511 | 2496295 | 204200 | 90035 | 73.7 |
| ***c*80*m*15*p*2*r*1** | 5900 | 494976 | 18805 | 8703 | 51.1 |
| ***c*80*m*15*p*2*r*2** | 5664 | 474635 | 17348 | 8000 | 49.2 |
| ***c*80*m*15*p*2*r*3** | 5595 | 468697 | 17121 | 7844 | 49.0 |
| ***c*80*m*15*p*6*r*1** | 11235 | 951544 | 57110 | 32880 | 44.2 |
| ***c*80*m*15*p*6*r*2** | 10881 | 920287 | 53571 | 31046 | 42.6 |
| ***c*80*m*15*p*6*r*3*** | 10799 | 913235 | 53123 | 30793 | 42.4 |
| ***c*85*m*4*p*2*r*1** | 21978 | 1850633 | 101330 | 38327 | 81.1 |
| ***c*85*m*4*p*2*r*2** | 21445 | 1804681 | 97990 | 36942 | 80.4 |
| ***c*85*m*4*p*2*r*3** | 20910 | 1759330 | 95476 | 35952 | 80.1 |
| ***c*85*m*4*p*6*r*1** | 30978 | 2625360 | 175593 | 79495 | 74.1 |
| ***c*85*m*4*p*6*r*2** | 30361 | 2570970 | 170188 | 76998 | 73.4 |
| ***c*85*m*4*p*6*r*3** | 29769 | 2520811 | 167056 | 75685 | 73.0 |
| ***c*85*m*15*p*2*r*1** | 6141 | 515247 | 18170 | 8279 | 51.2 |
| ***c*85*m*15*p*2*r*2** | 5908 | 495068 | 16925 | 7675 | 49.3 |
| ***c*85*m*15*p*2*r*3** | 5832 | 488595 | 16665 | 7504 | 49.1 |
| ***c*85*m*15*p*6*r*1** | 11748 | 995395 | 56941 | 32661 | 44.1 |
| ***c*85*m*15*p*6*r*2** | 11389 | 963773 | 53692 | 30950 | 42.5 |
| ***c*85*m*15*p*6*r*3** | 11297 | 955882 | 53232 | 30673 | 42.3 |
| ***c*90*m*4*p*2*r*1** | 22356 | 1885295 | 73526 | 27690 | 80.3 |
| ***c*90*m*4*p*2*r*2** | 21793 | 1836877 | 70798 | 26623 | 79.5 |
| ***c*90*m*4*p*2*r*3** | 21261 | 1791862 | 69019 | 25938 | 79.2 |
| ***c*90*m*4*p*6*r*1** | 31495 | 2670428 | 138063 | 63483 | 73.5 |
| ***c*90*m*4*p*6*r*2** | 30846 | 2613484 | 133377 | 61435 | 72.7 |
| ***c*90*m*4*p*6*r*3** | 30247 | 2562629 | 130964 | 60392 | 72.4 |
| ***c*90*m*15*p*2*r*1** | 6658 | 559417 | 18232 | 8231 | 51.1 |
| ***c*90*m*15*p*2*r*2** | 6407 | 537801 | 17025 | 7685 | 49.3 |
| ***c*90*m*15*p*2*r*3** | 6324 | 530706 | 16797 | 7544 | 49.0 |
| ***c*90*m*15*p*6*r*1** | 12397 | 1049683 | 54663 | 30941 | 44.3 |
| ***c*90*m*15*p*6*r*2** | 12010 | 1015764 | 51523 | 29370 | 42.7 |
| ***c*90*m*15*p*6*r*3** | 11898 | 1006193 | 51012 | 29043 | 42.5 |
