# Supplementary figures and images for: Climatic niche pre-adaptation facilitated island colonization followed by budding speciation in the Madeiran ivy (Hedera maderensis, Araliaceae)
Source: Front Plant Sci. 2022 Jul 25;13:935975. doi: 10.3389/fpls.2022.935975 (PMC9358290; doi:10.3389/fpls.2022.935975)

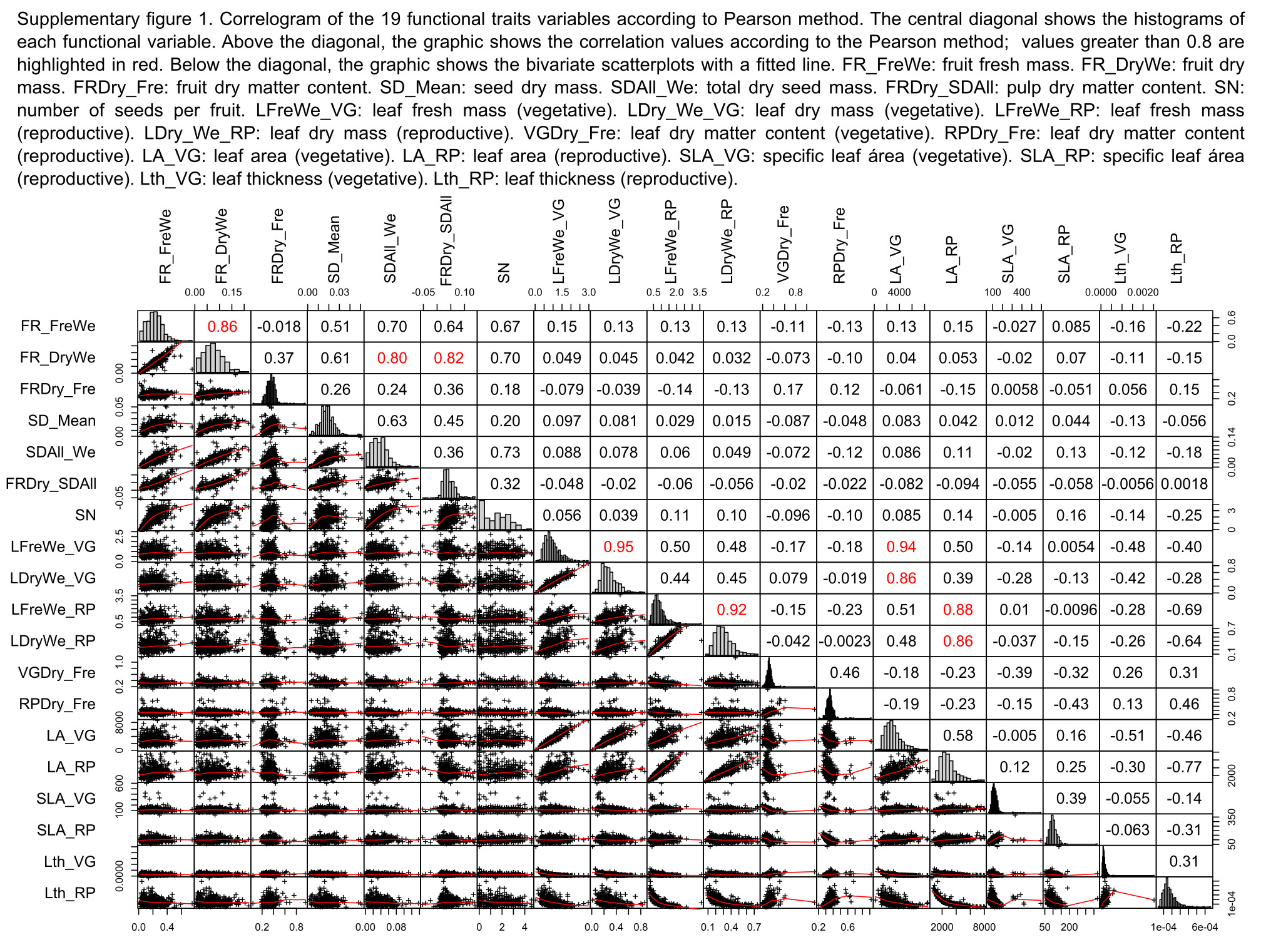

Supplement: Supplementary Figure 1 — Correlogram of the 19 functional trait variables according to Pearson correlation coefficients. [file Image_1.JPEG]

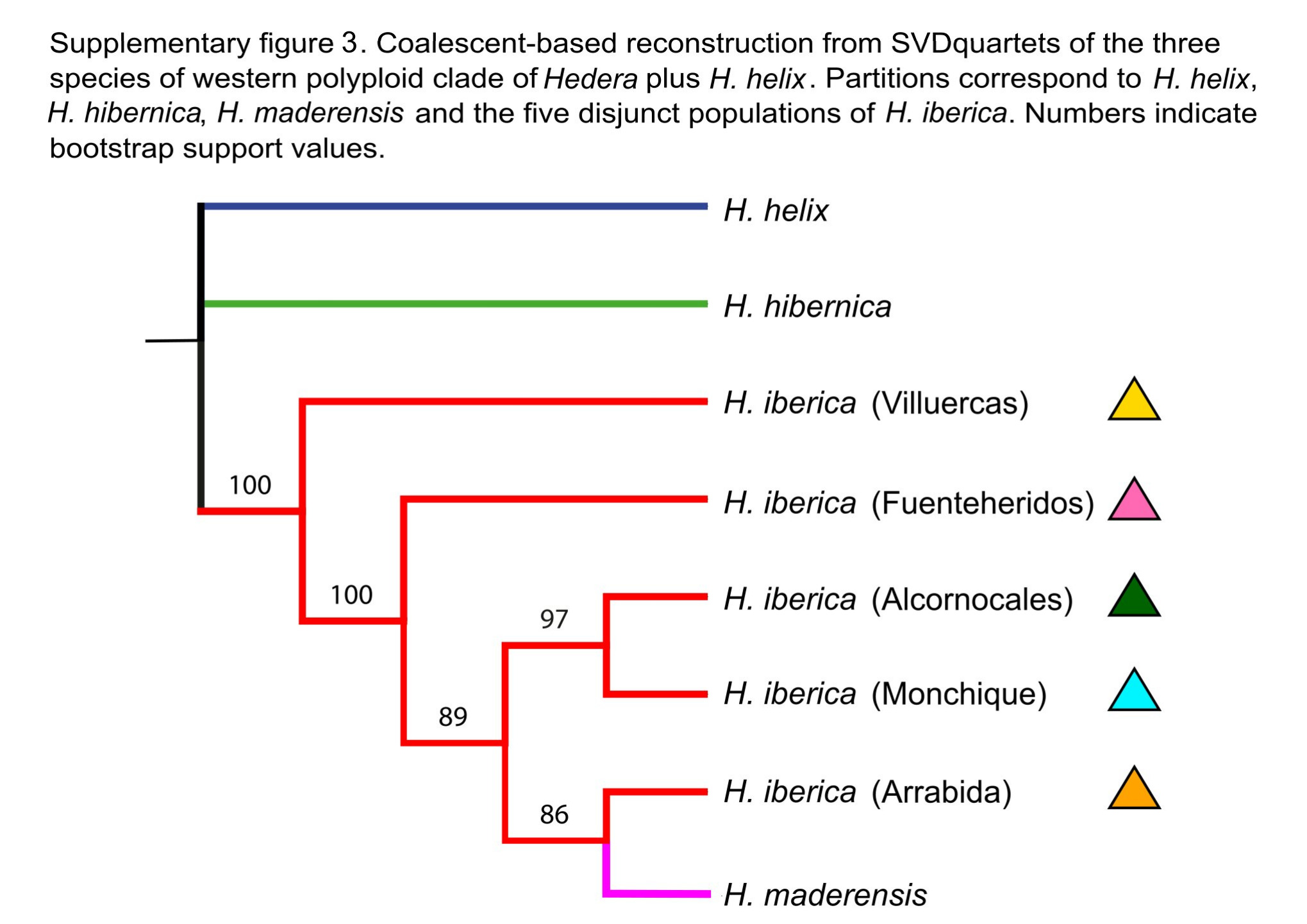

Supplement: Supplementary Figure 3 — Consensus tree from the coalescent-based SVDquartets analysis. [file Image_3.jpeg]

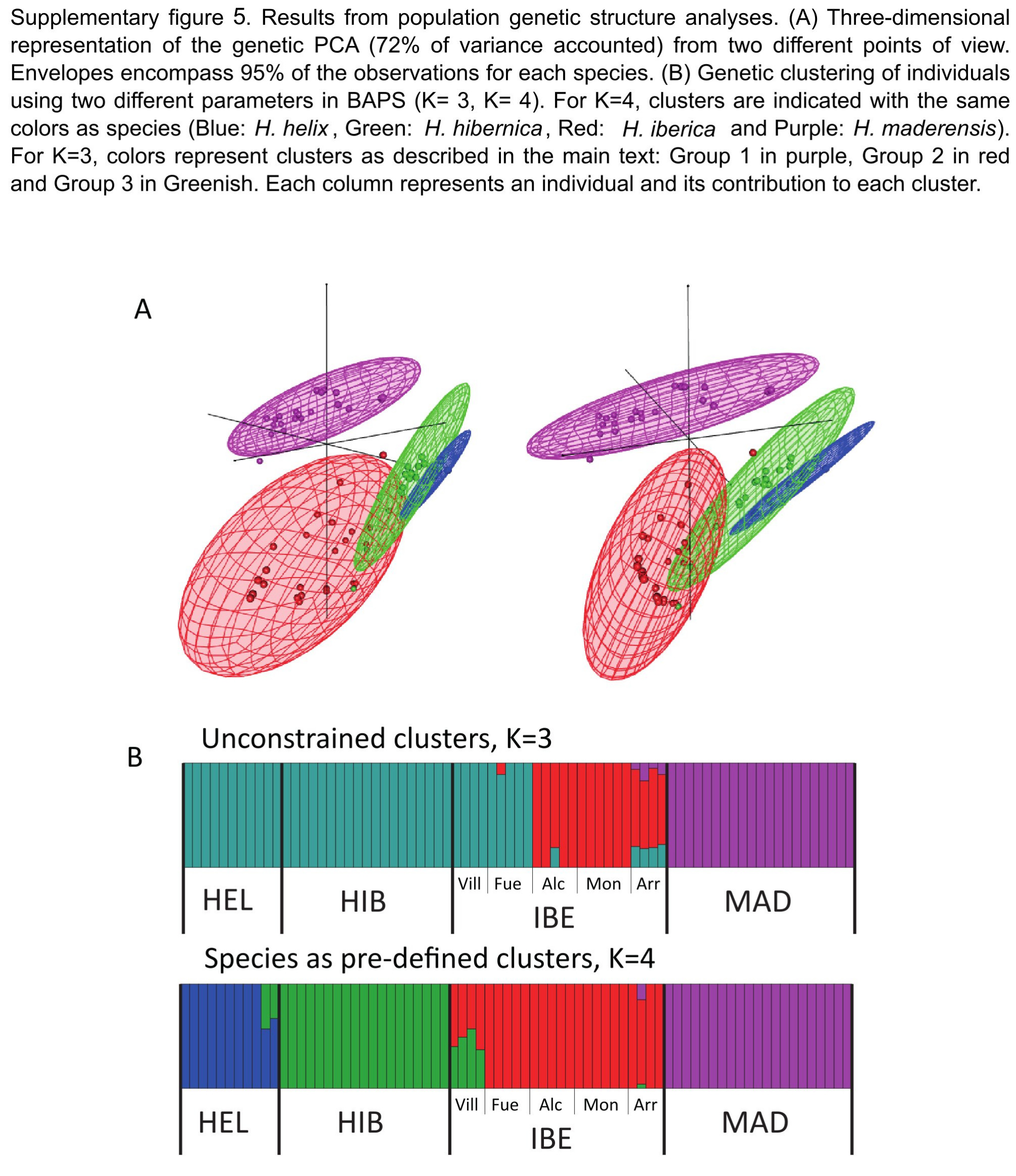

Supplement: Supplementary Figure 5 — Results from population genetic structure analyses (genetic PCA and BAPS). [file Image_5.jpeg]

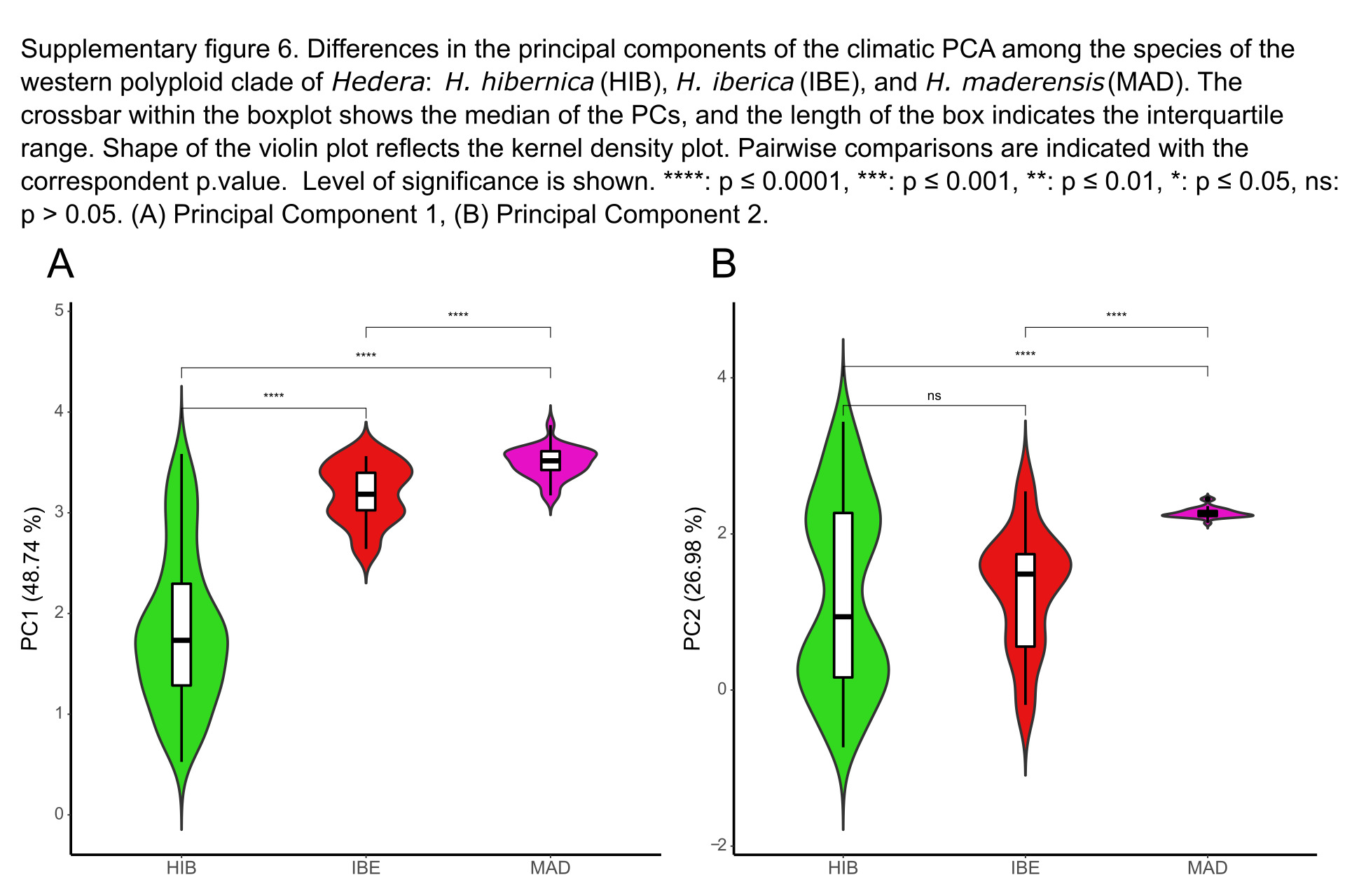

Supplement: Supplementary Figure 6 — Violin plots of the first two axes of the climatic PCA. [file Image_6.jpeg]

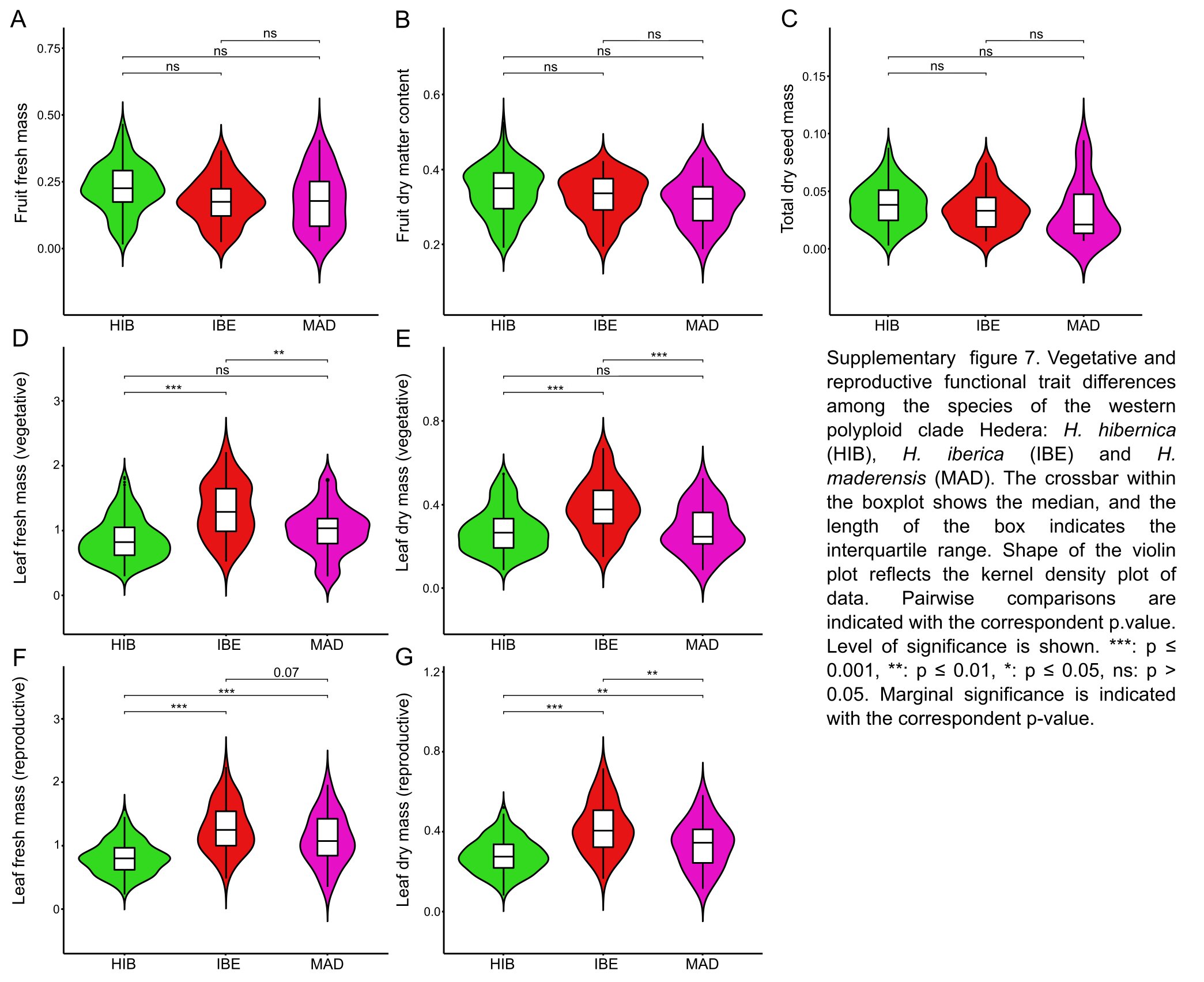

Supplement: Supplementary Figure 7 — Violin plots of the functional traits. [file Image_7.jpeg]
