# Supplementary material for: Climatic niche pre-adaptation facilitated island colonization followed by budding speciation in the Madeiran ivy (Hedera maderensis, Araliaceae)
Source: Front Plant Sci. 2022 Jul 25;13:935975. doi: 10.3389/fpls.2022.935975 (PMC9358290; doi:10.3389/fpls.2022.935975)

Fruit fresh mass

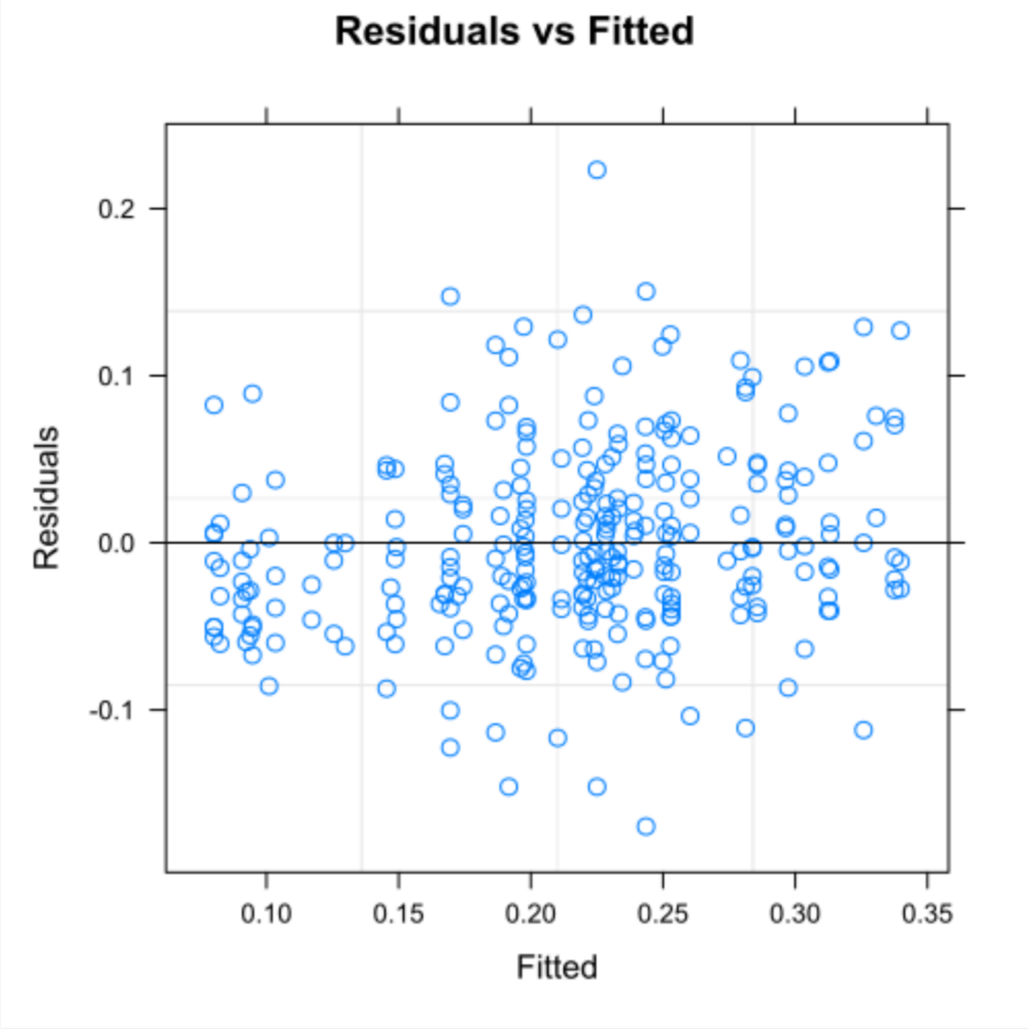

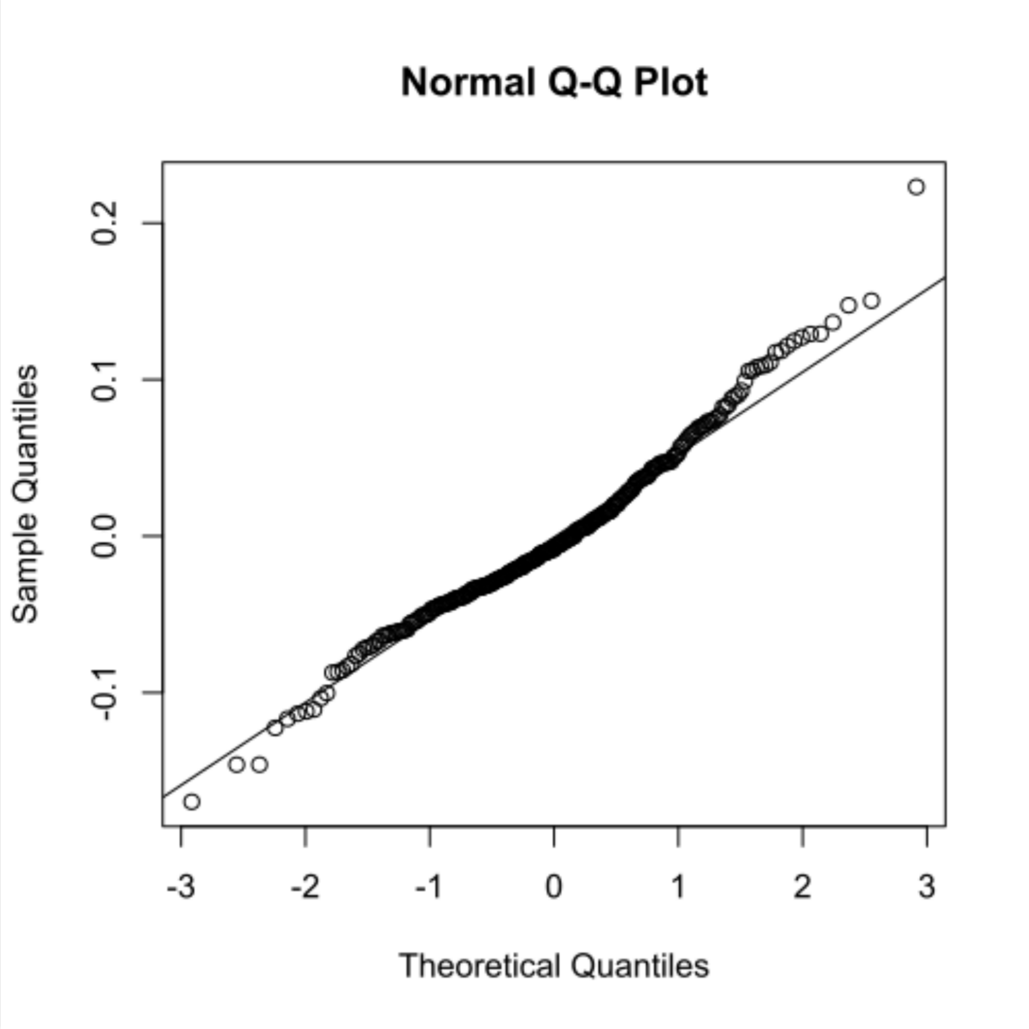

Fruit dry mass

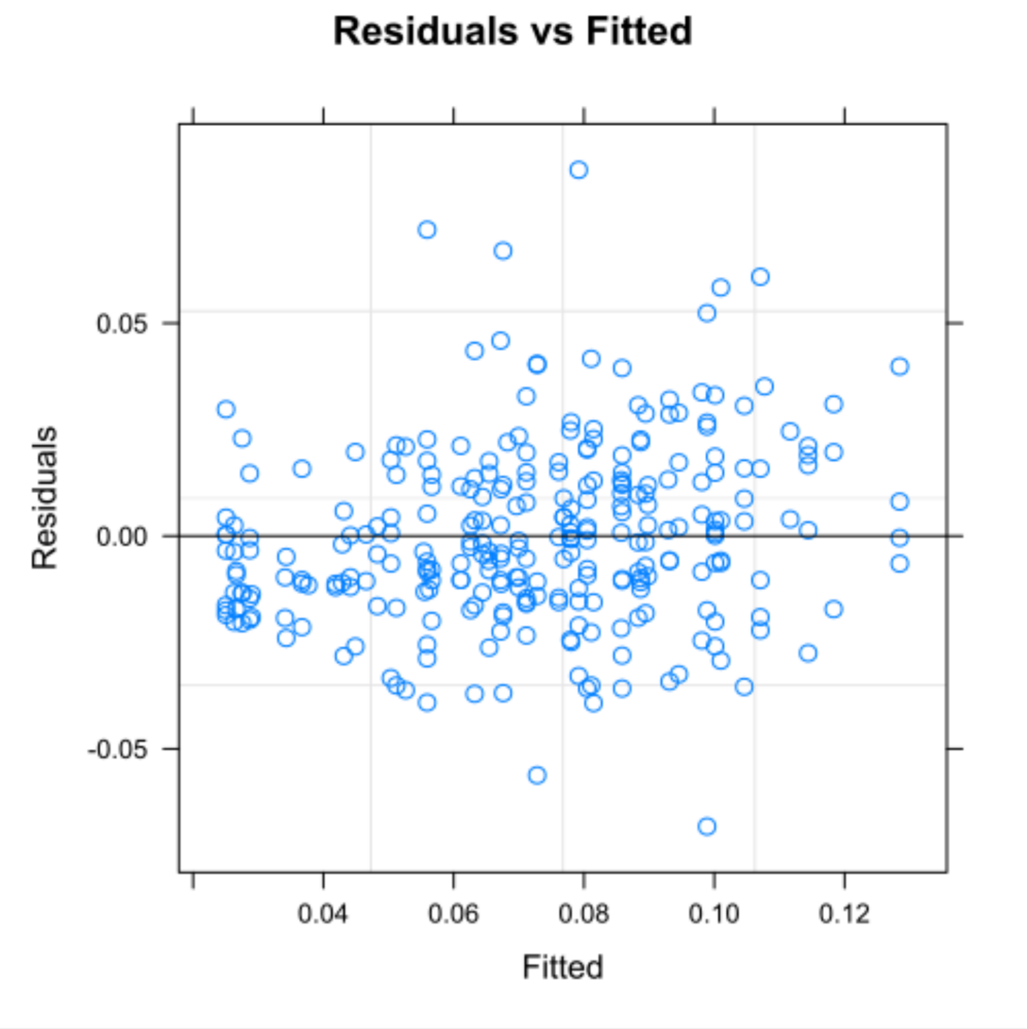

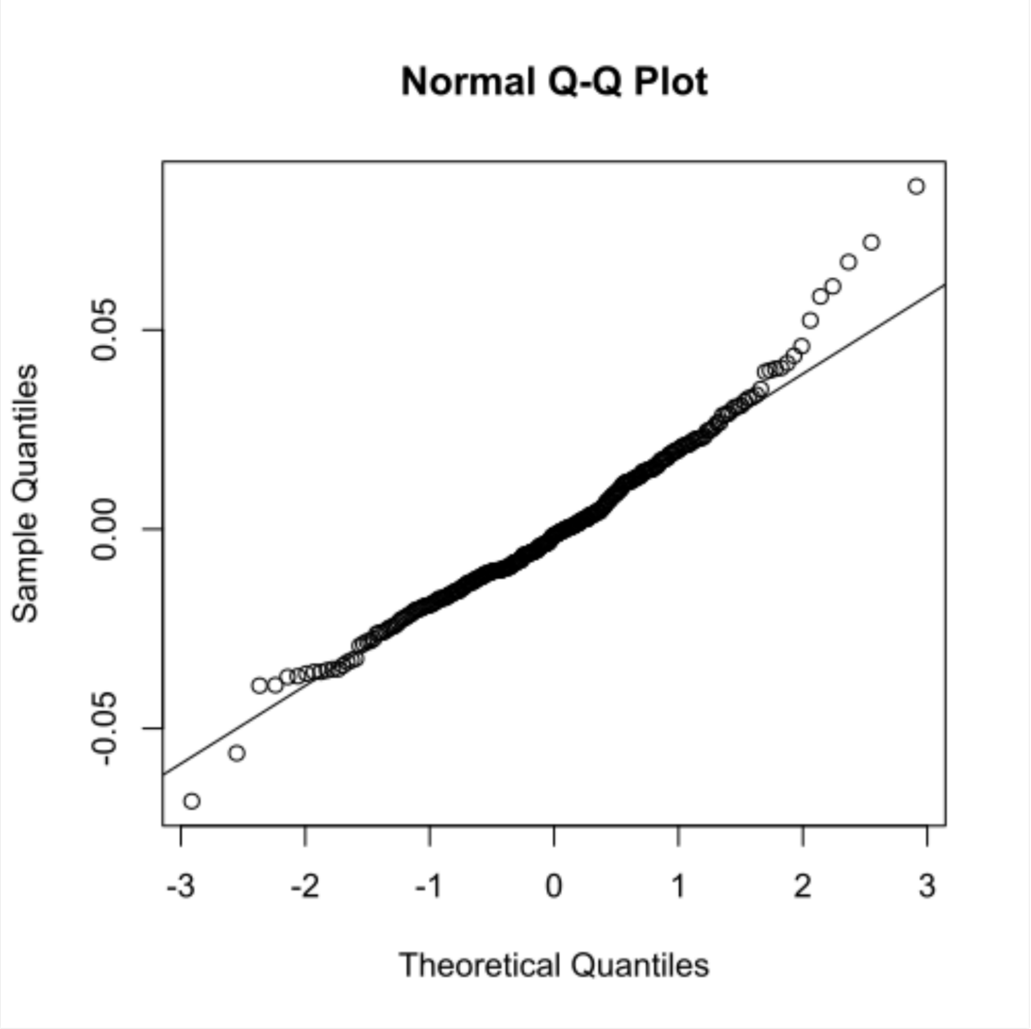

Fruit dry matter content

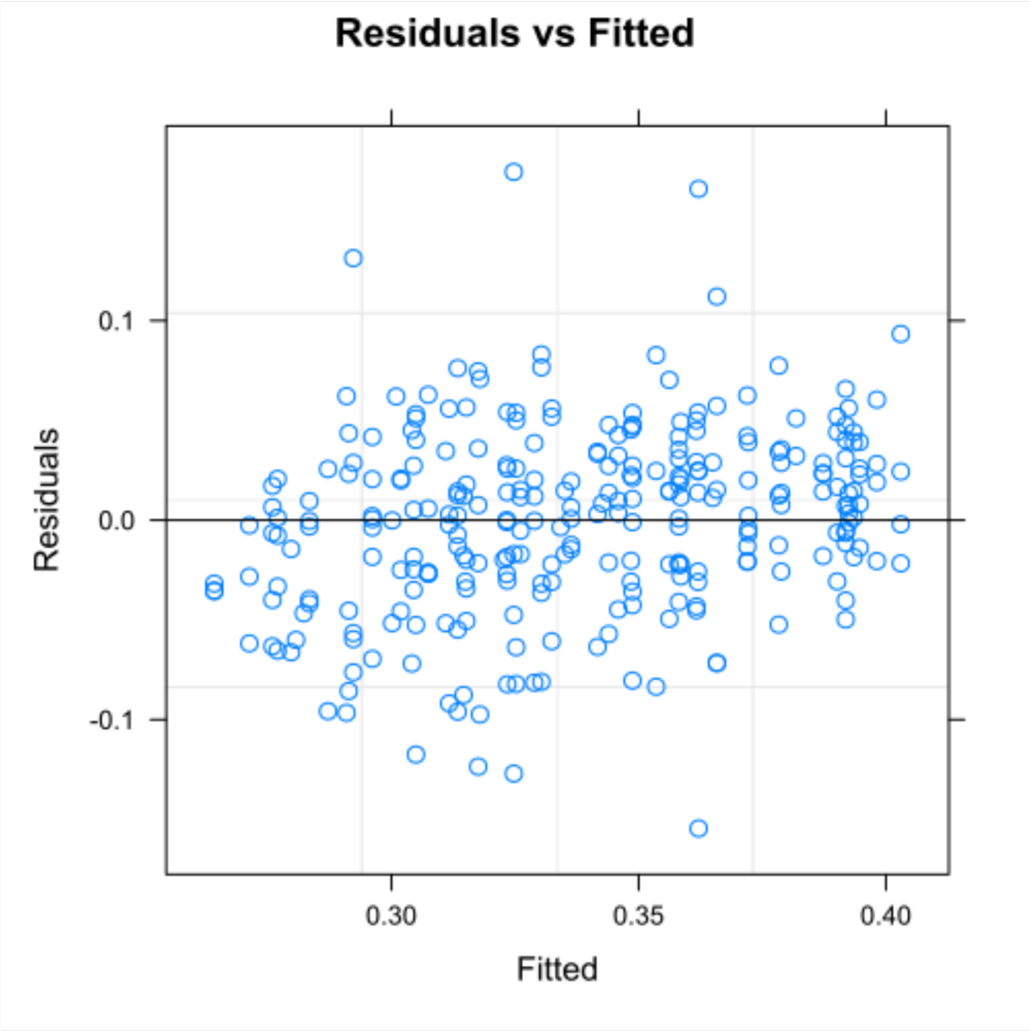

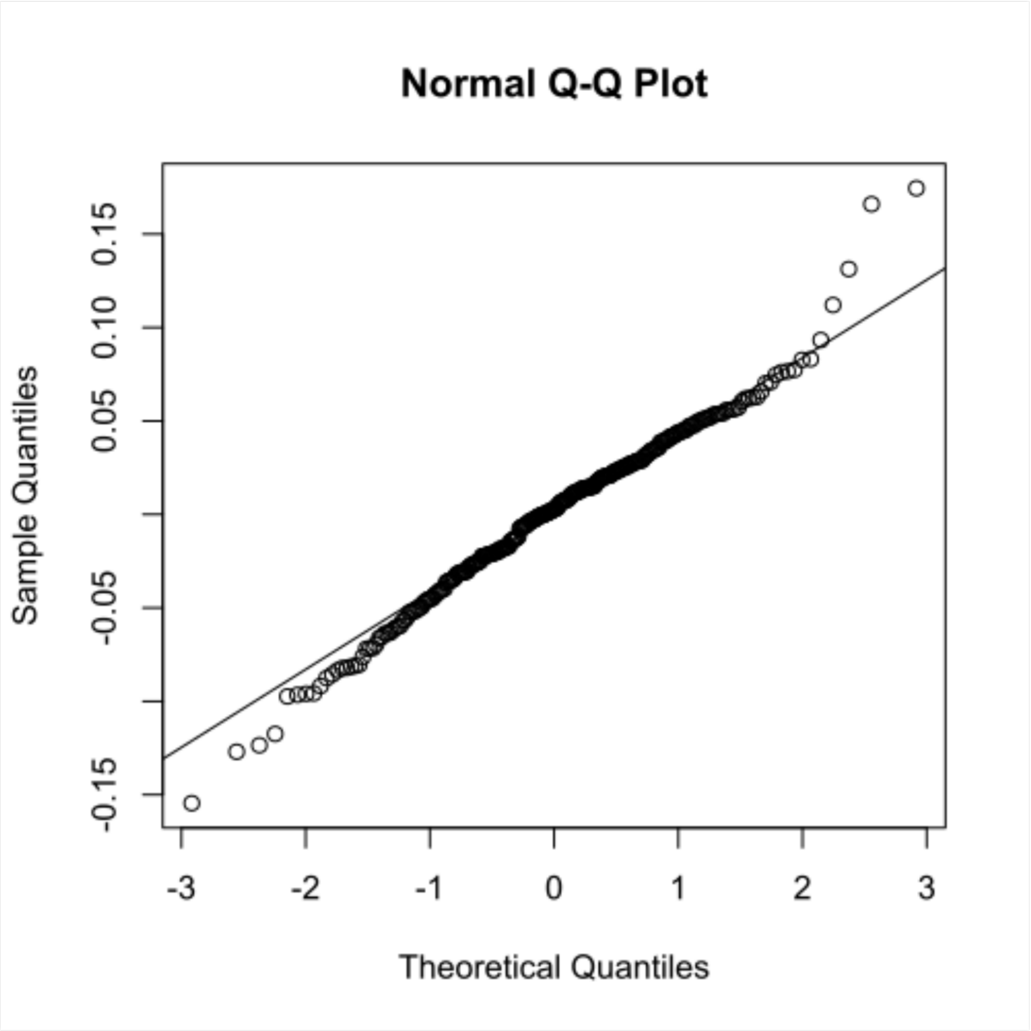

Seed dry mass

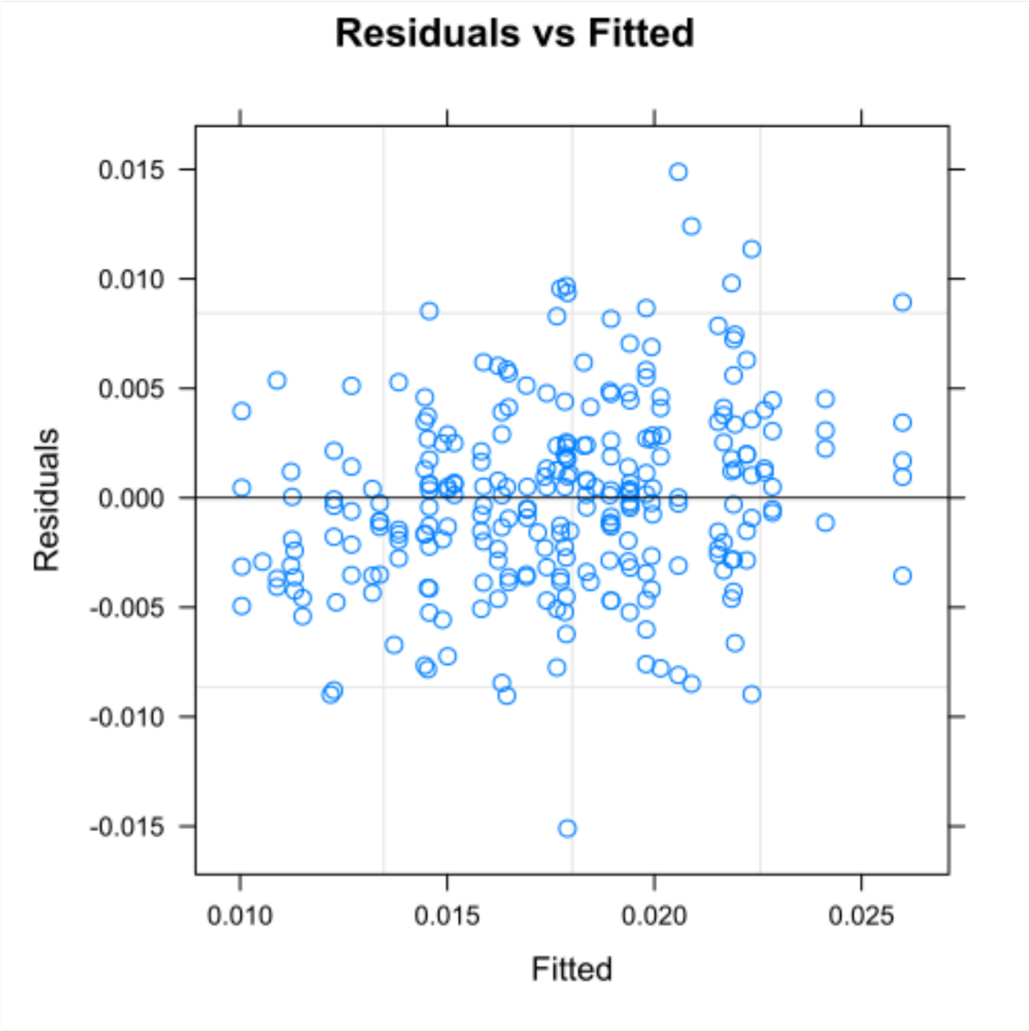

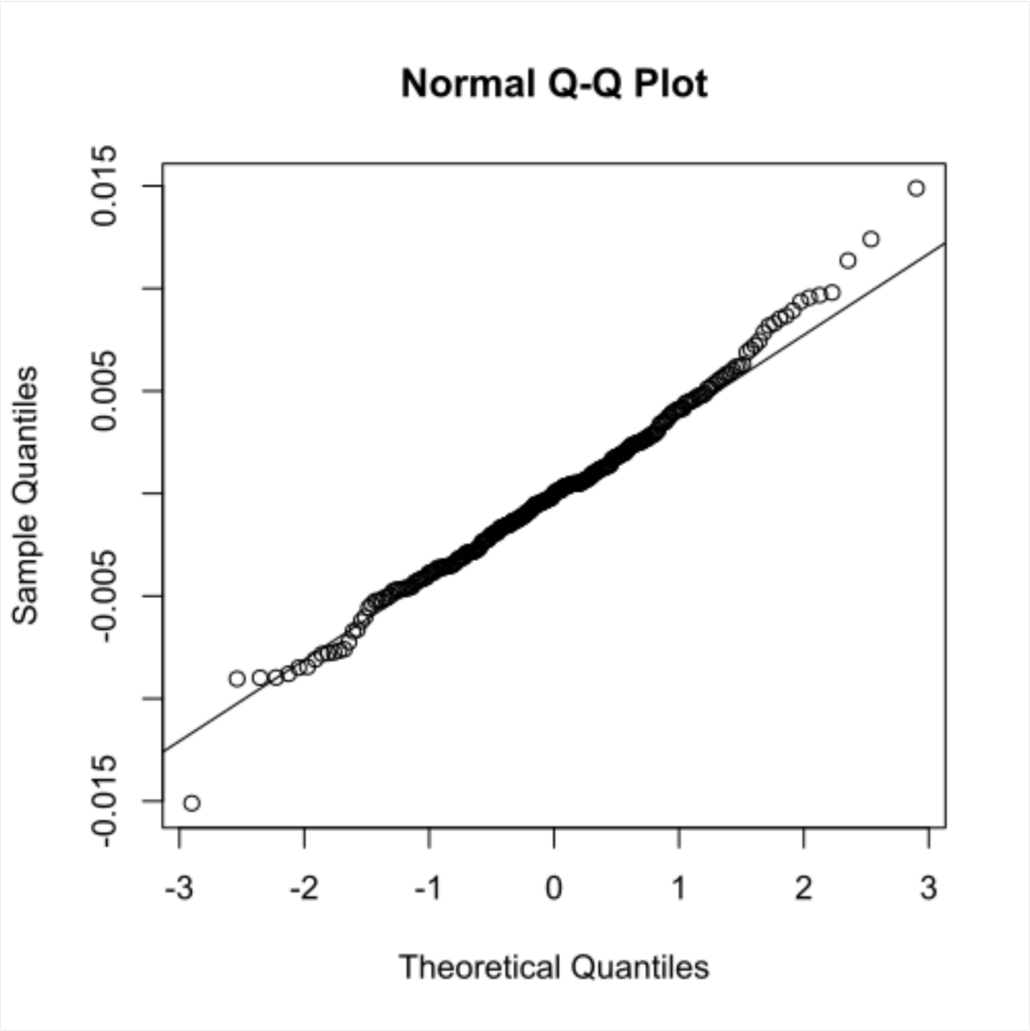

Total dry seed mass

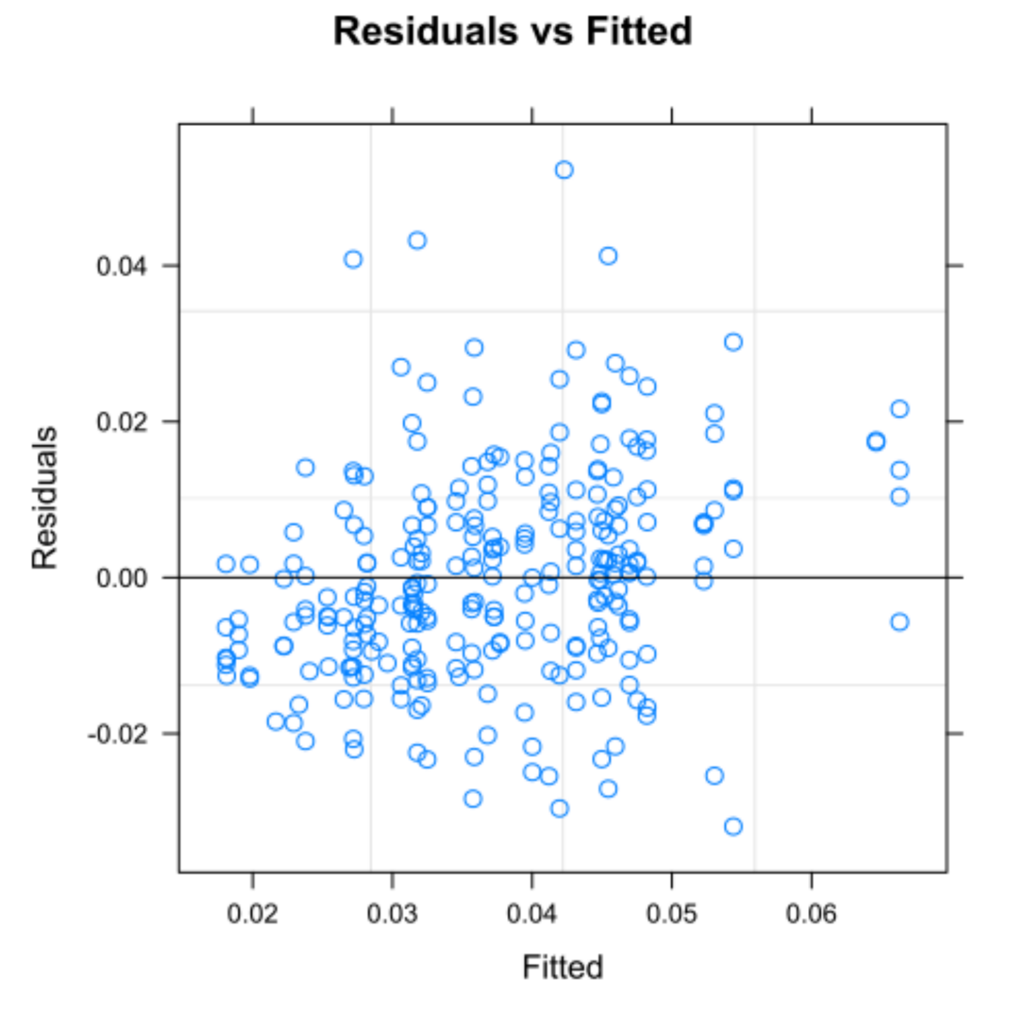

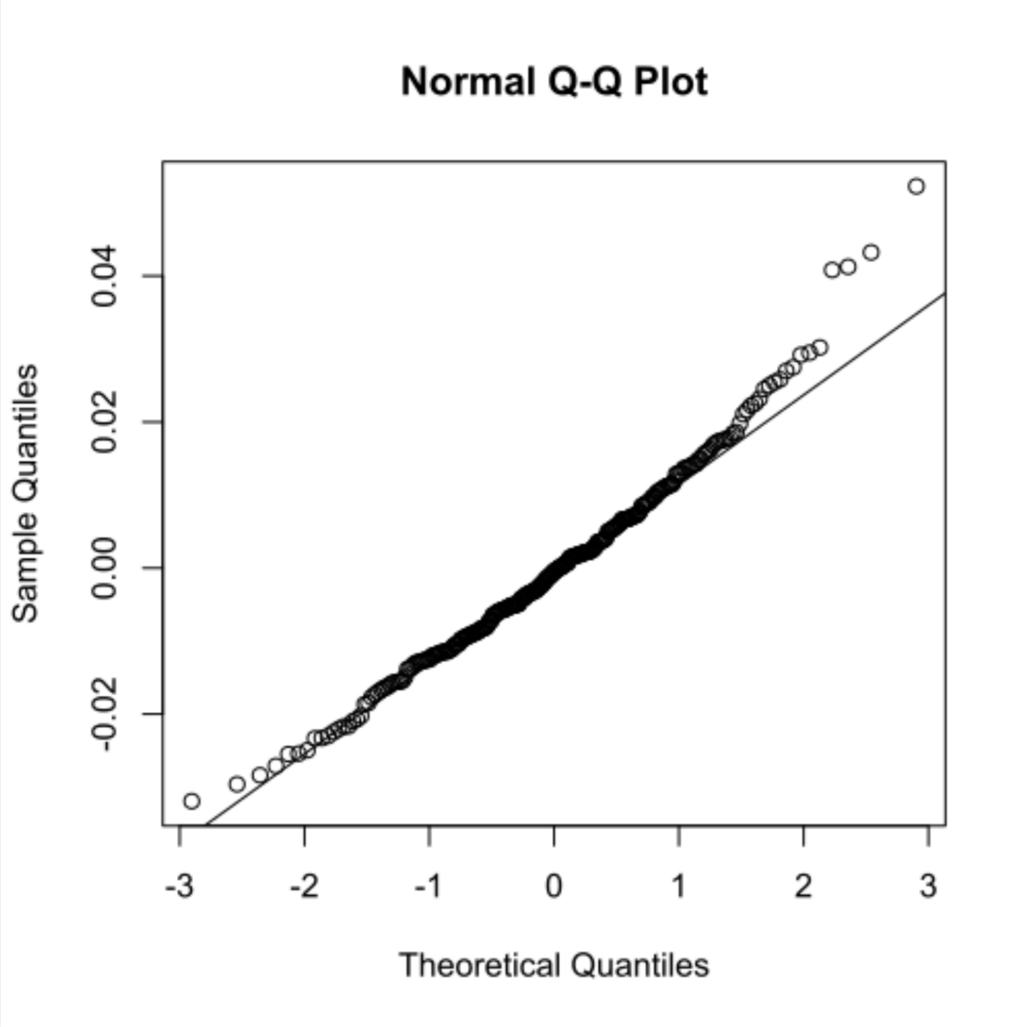

Pulp dry matter content

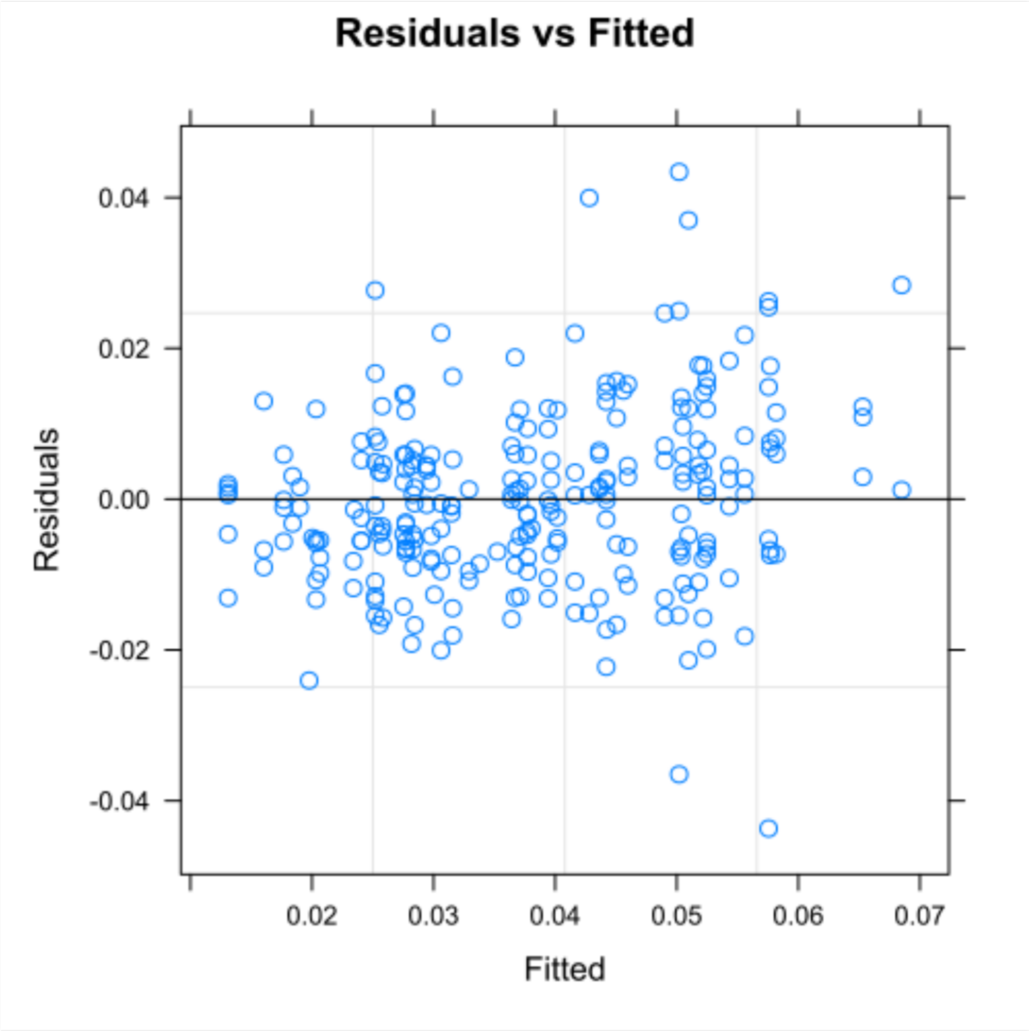

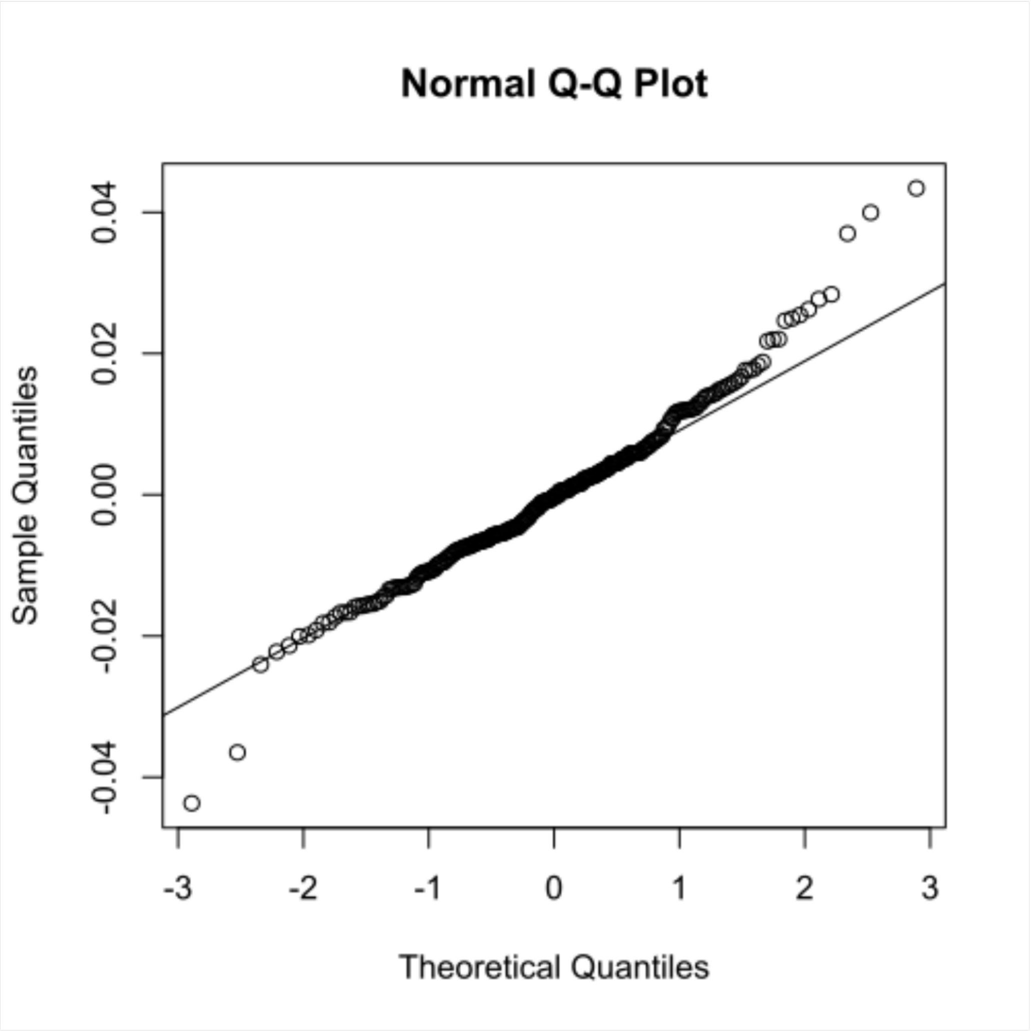

Number of seeds per fruit

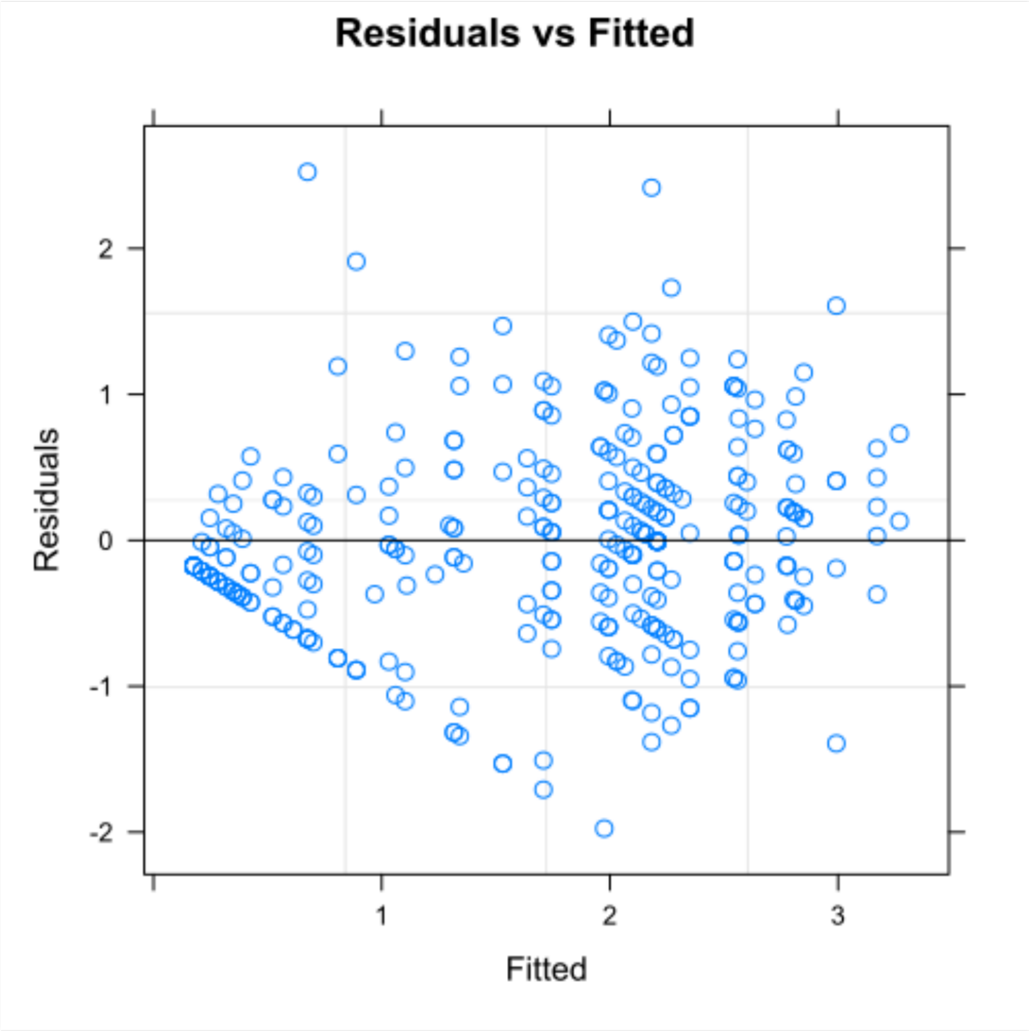

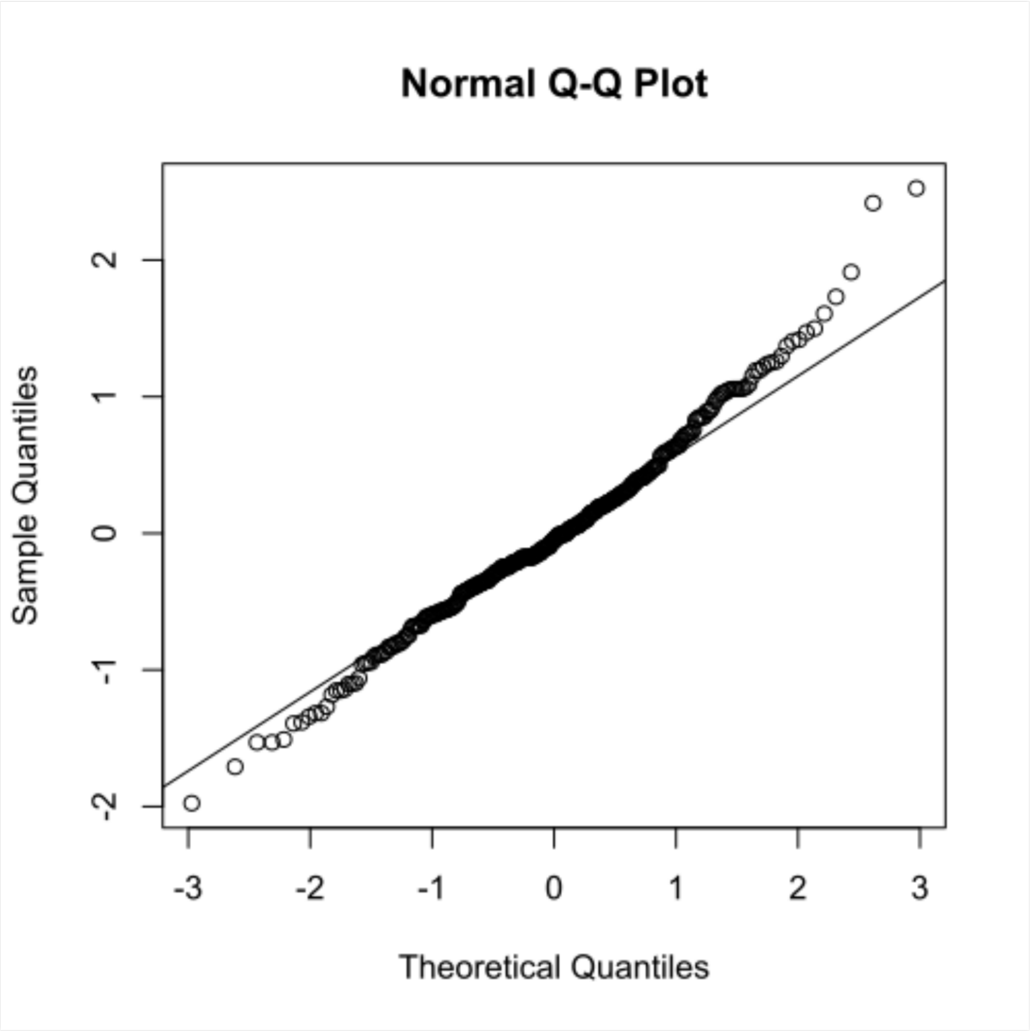

Leaf fresh mass (vegetative)

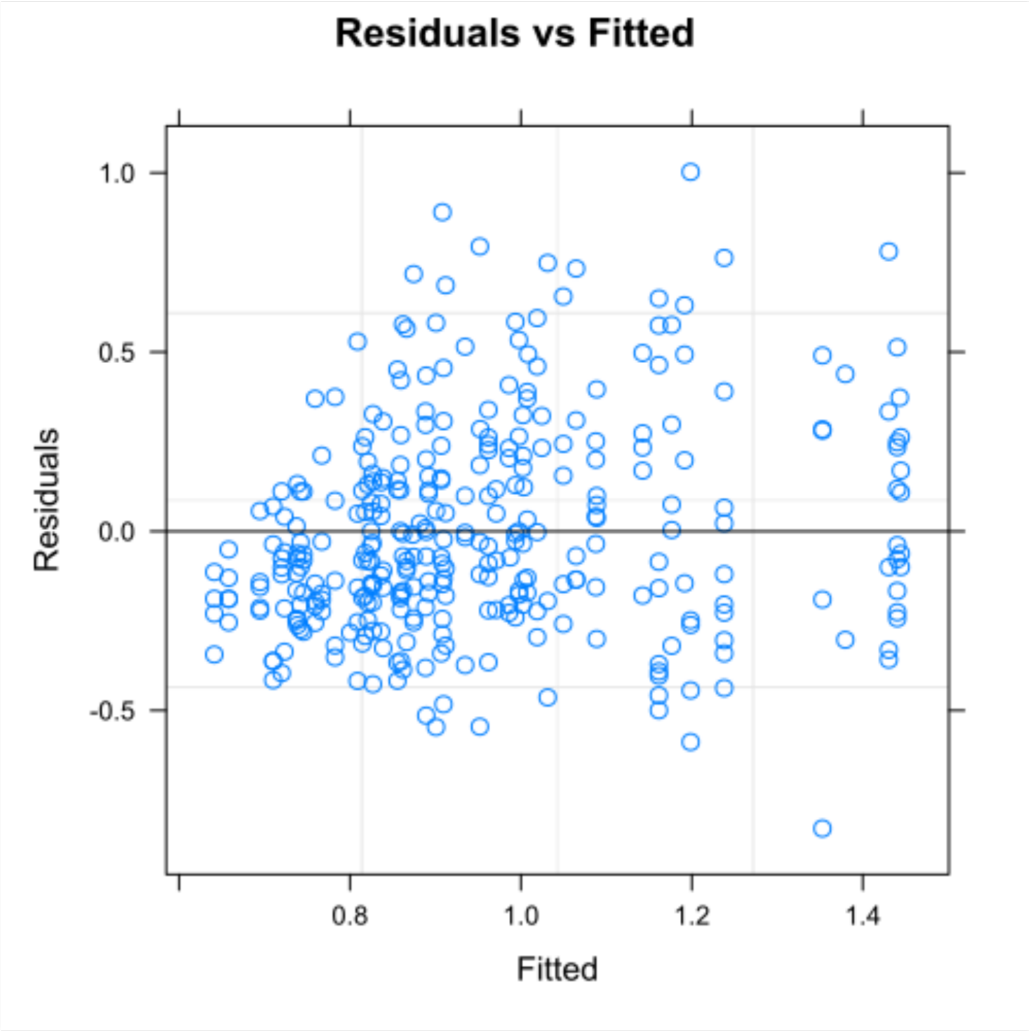

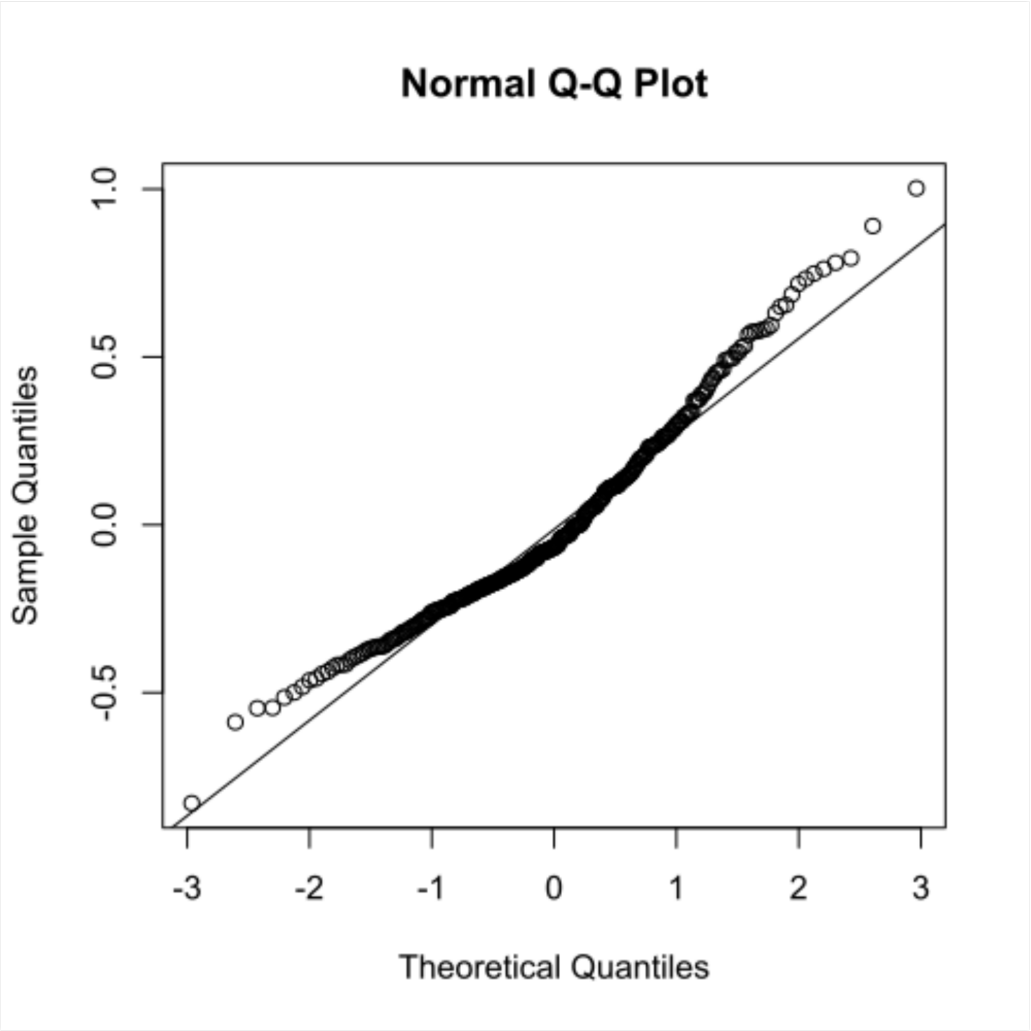

Leaf dry mass (vegetative)

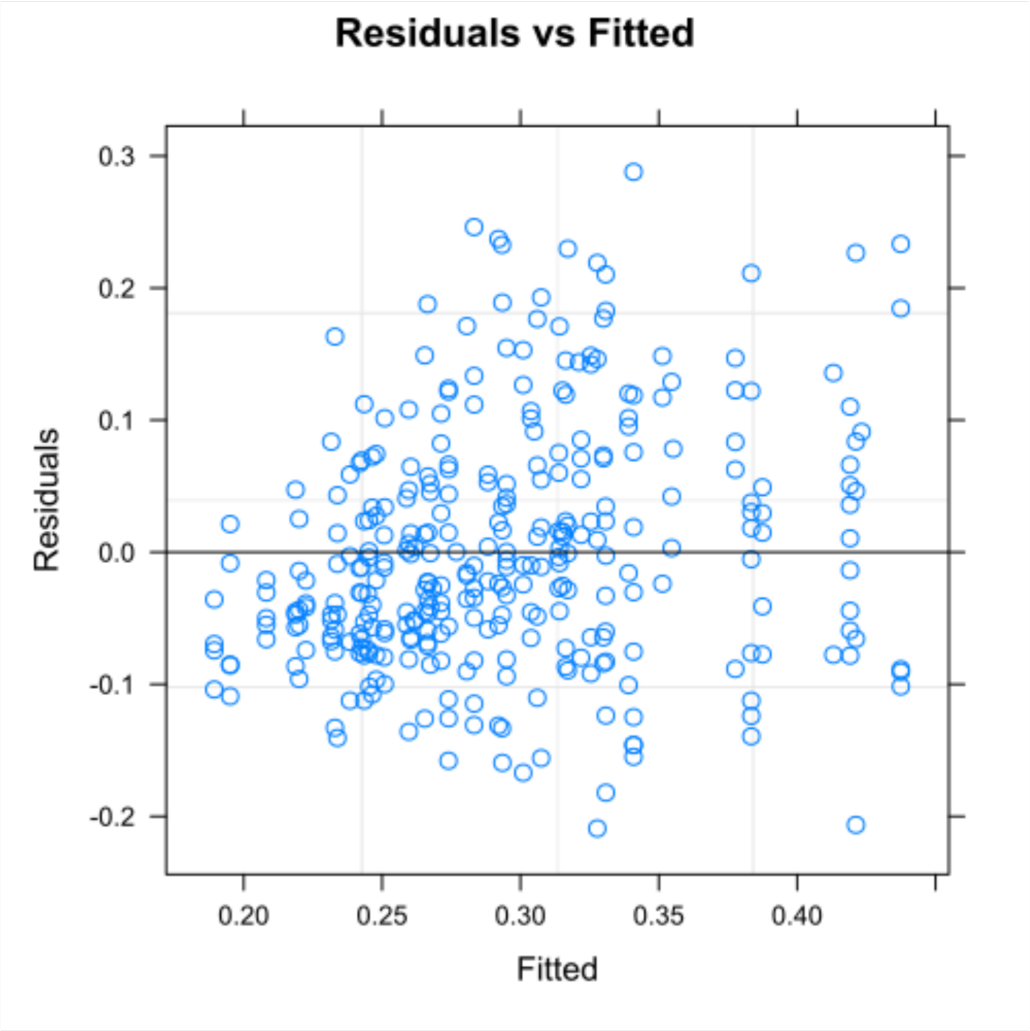

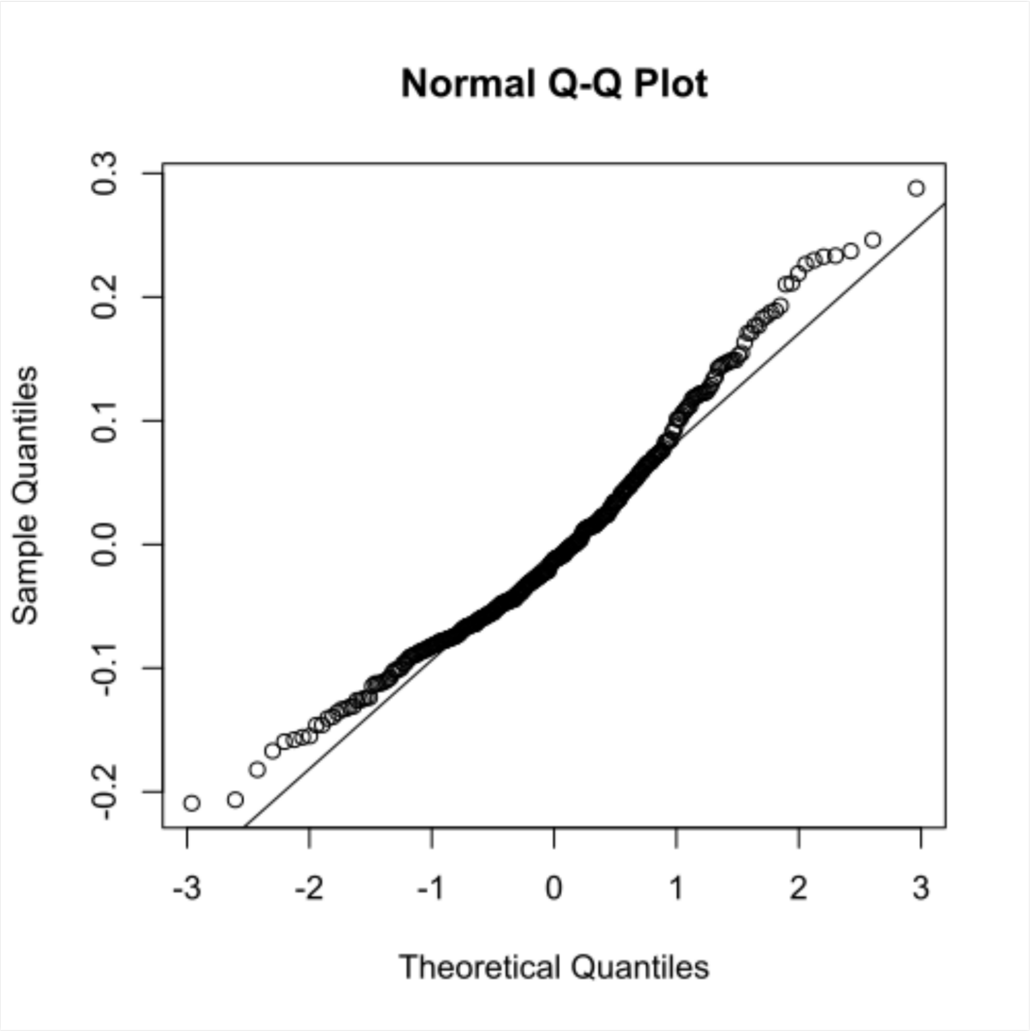

Leaf fresh mass (reproductive)

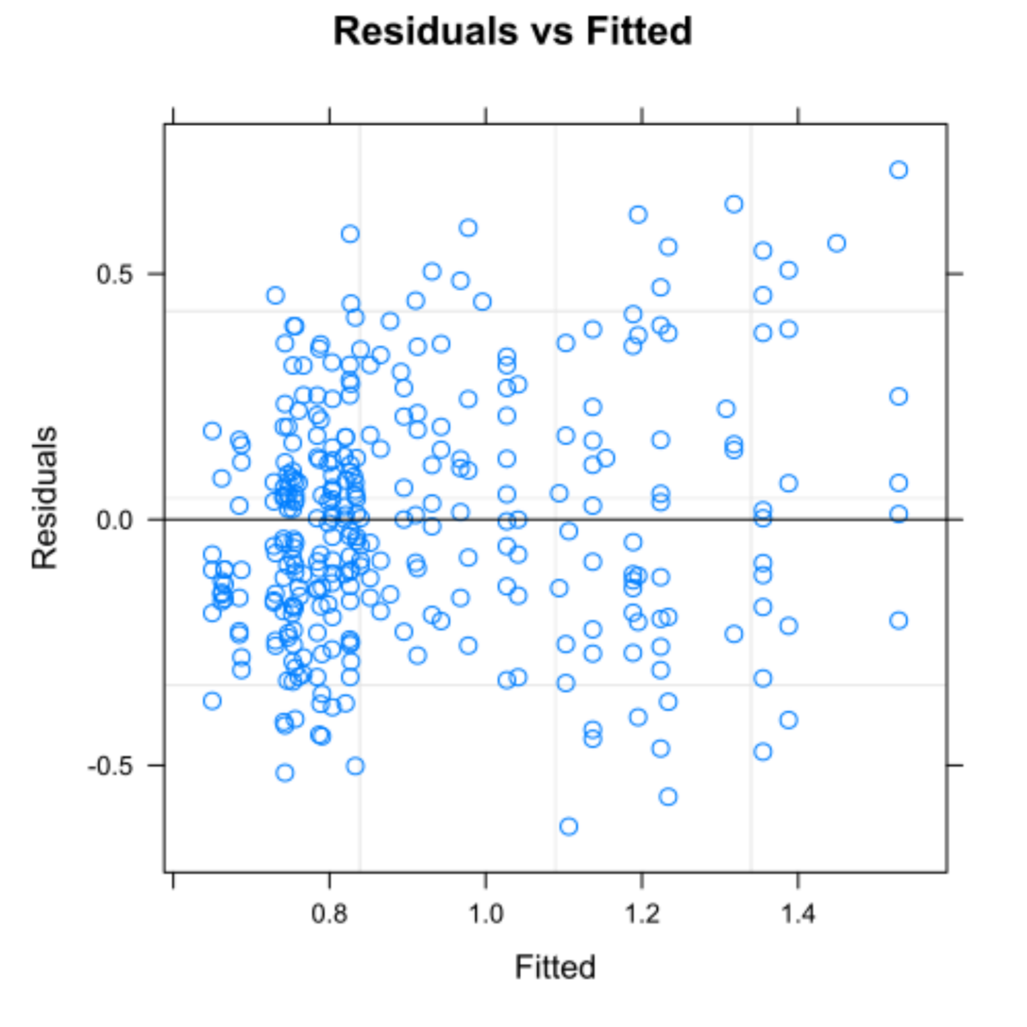

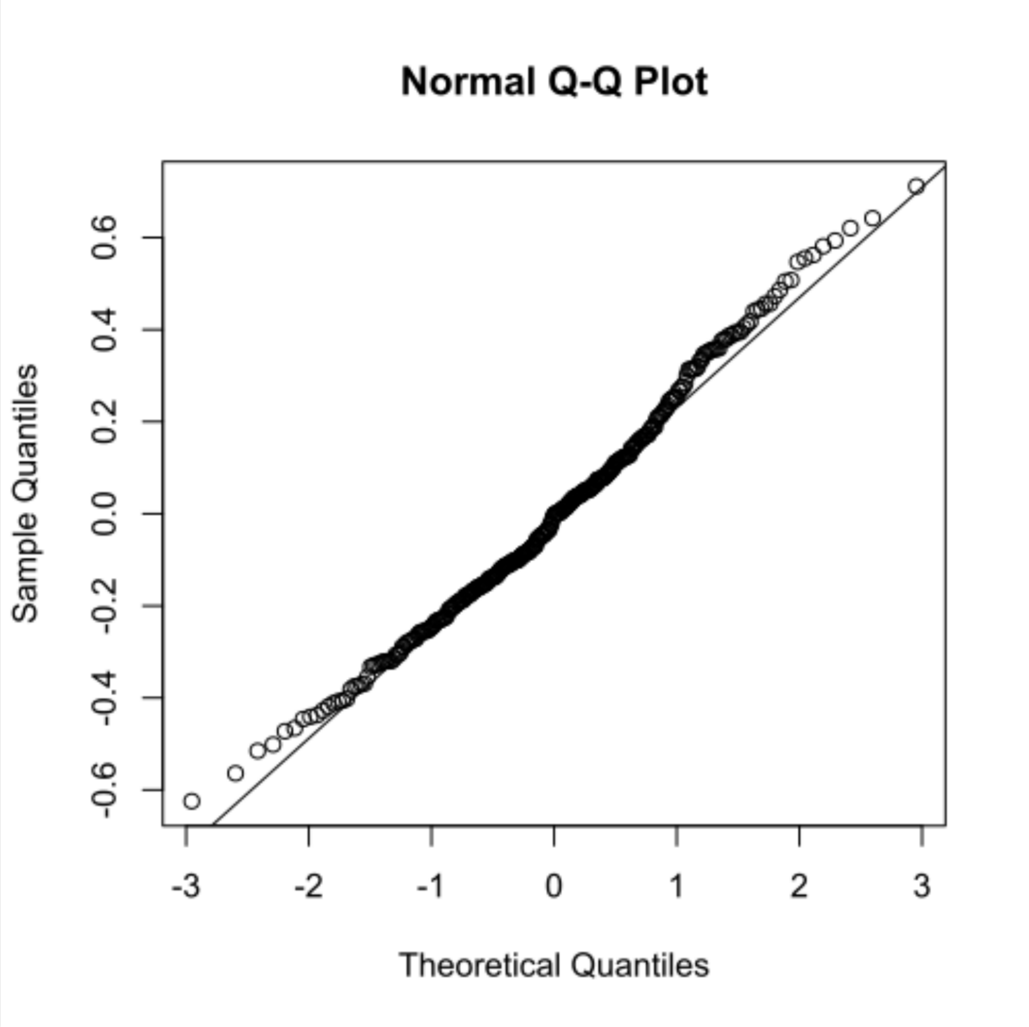

Leaf dry mass (reproductive)

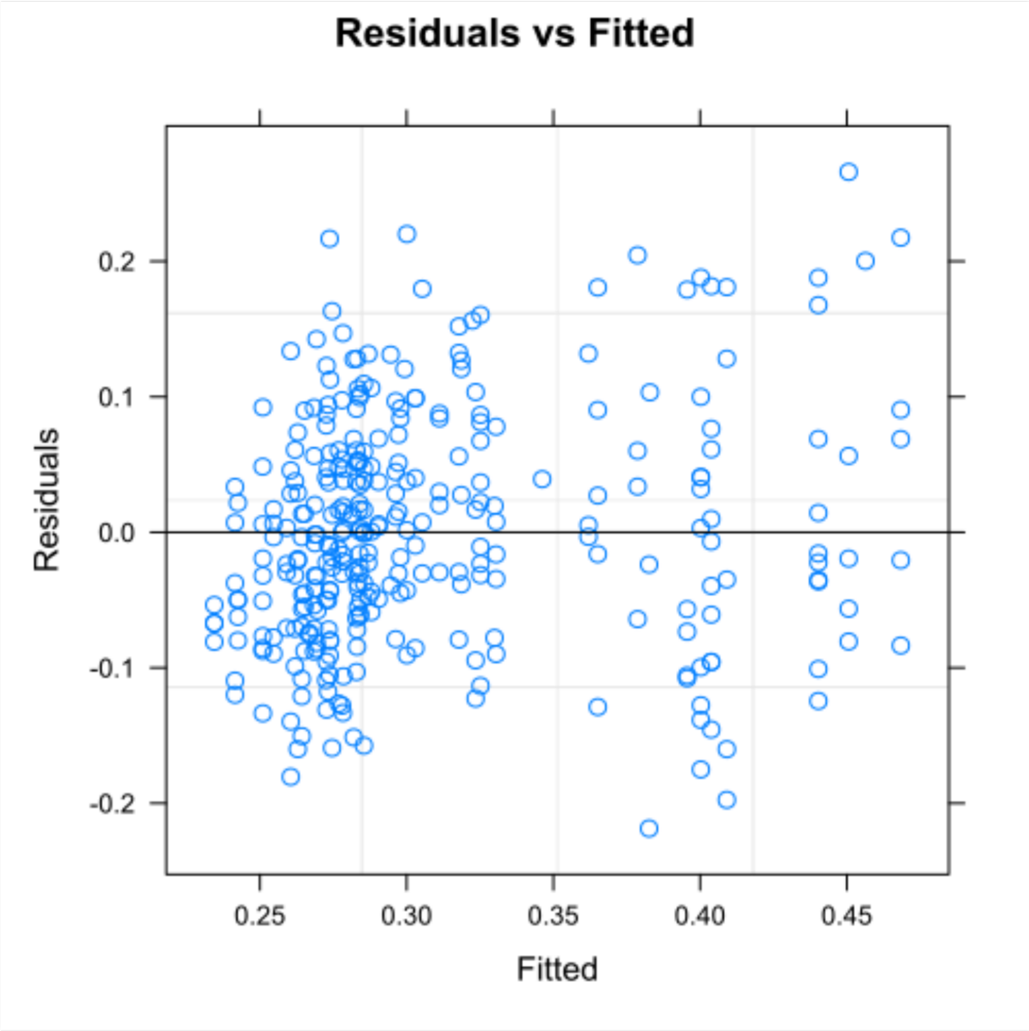

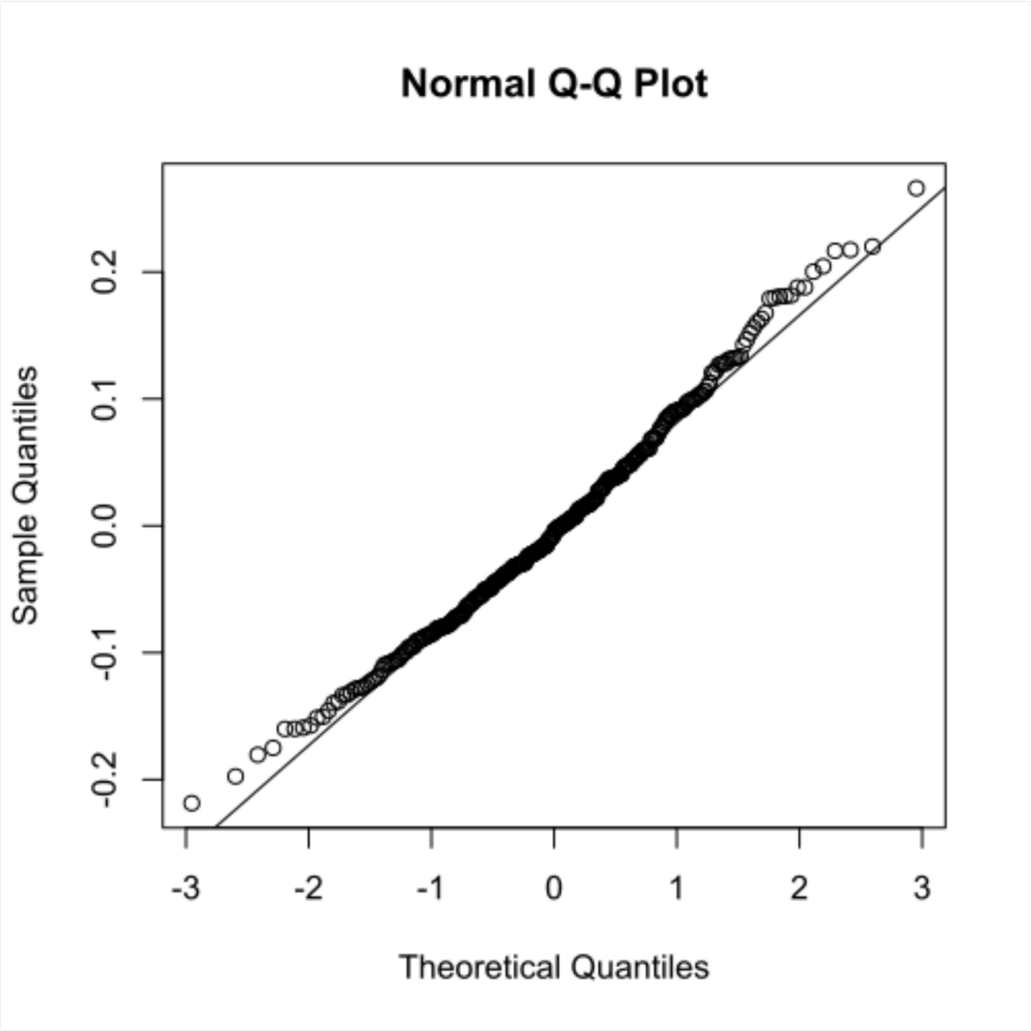

Leaf dry matter content (vegetative)

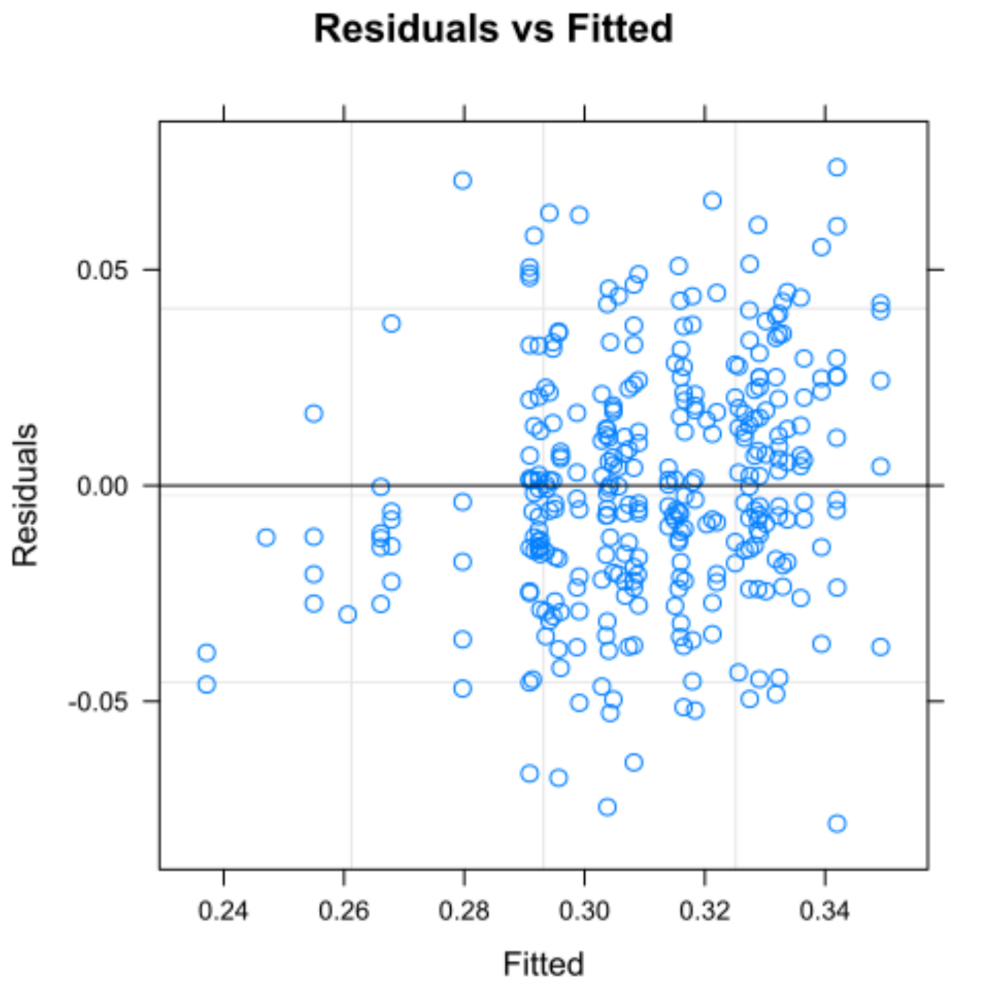

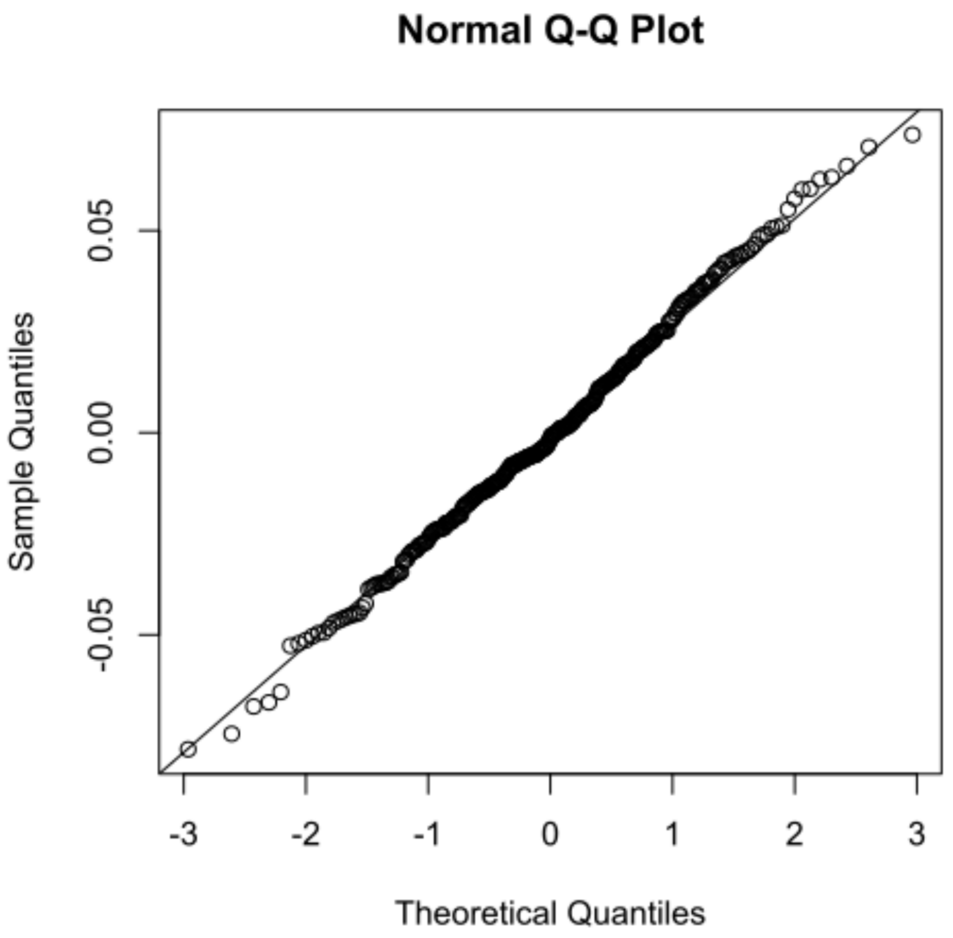

Leaf dry matter content (reproductive)

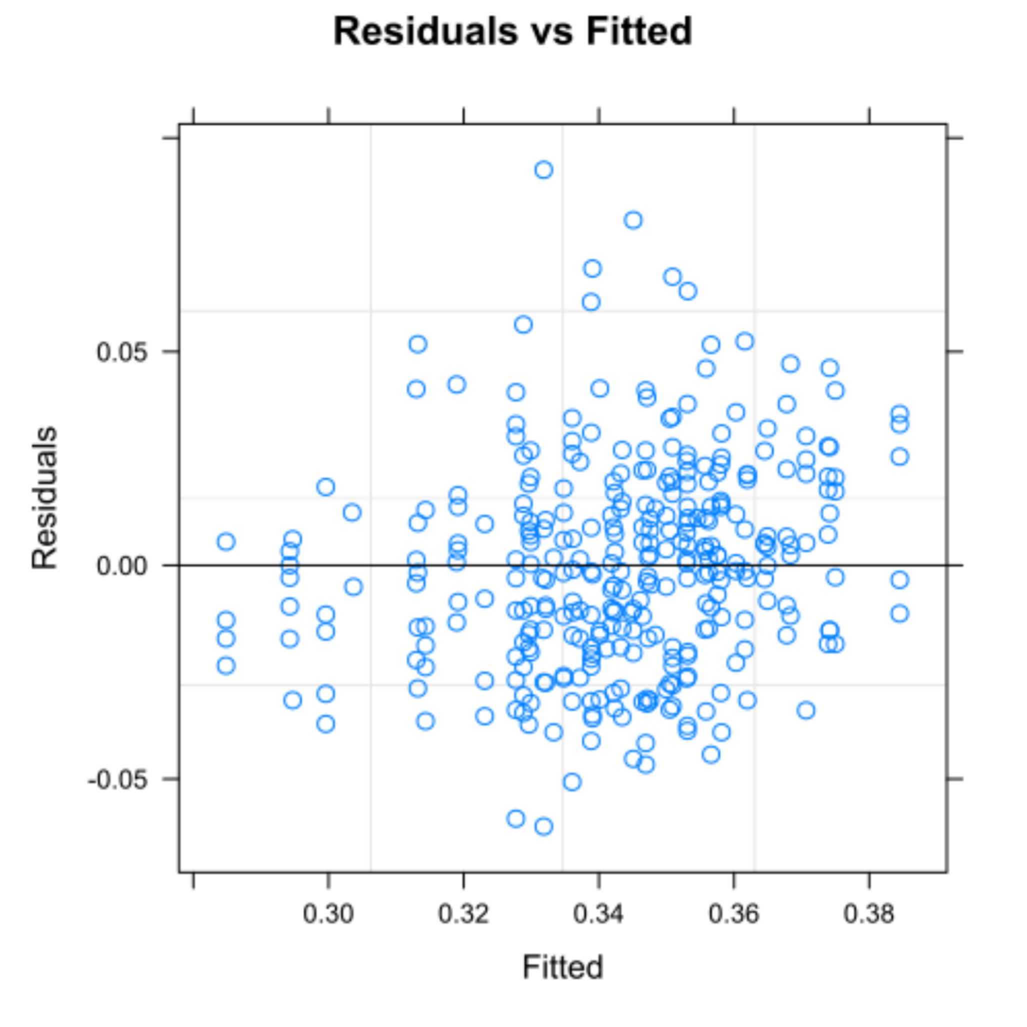

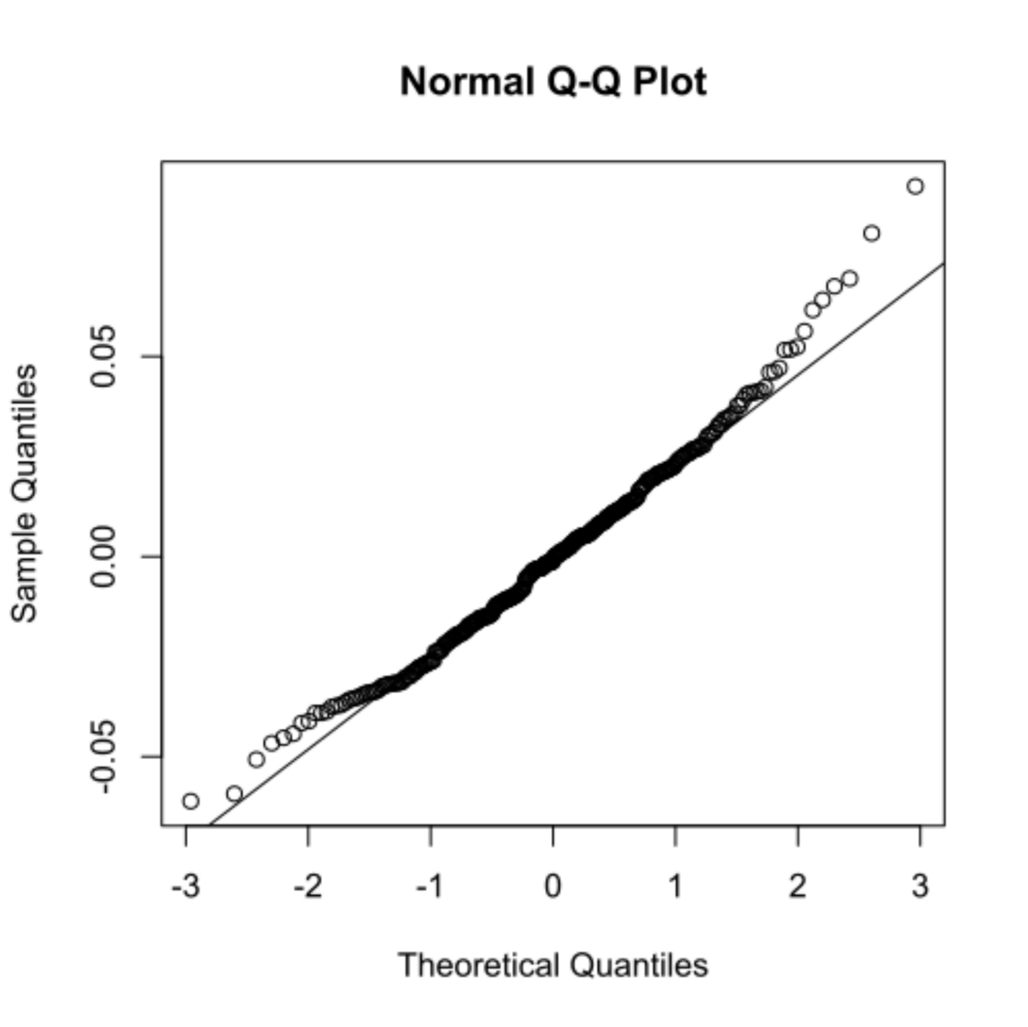

Leaf area (vegetative)

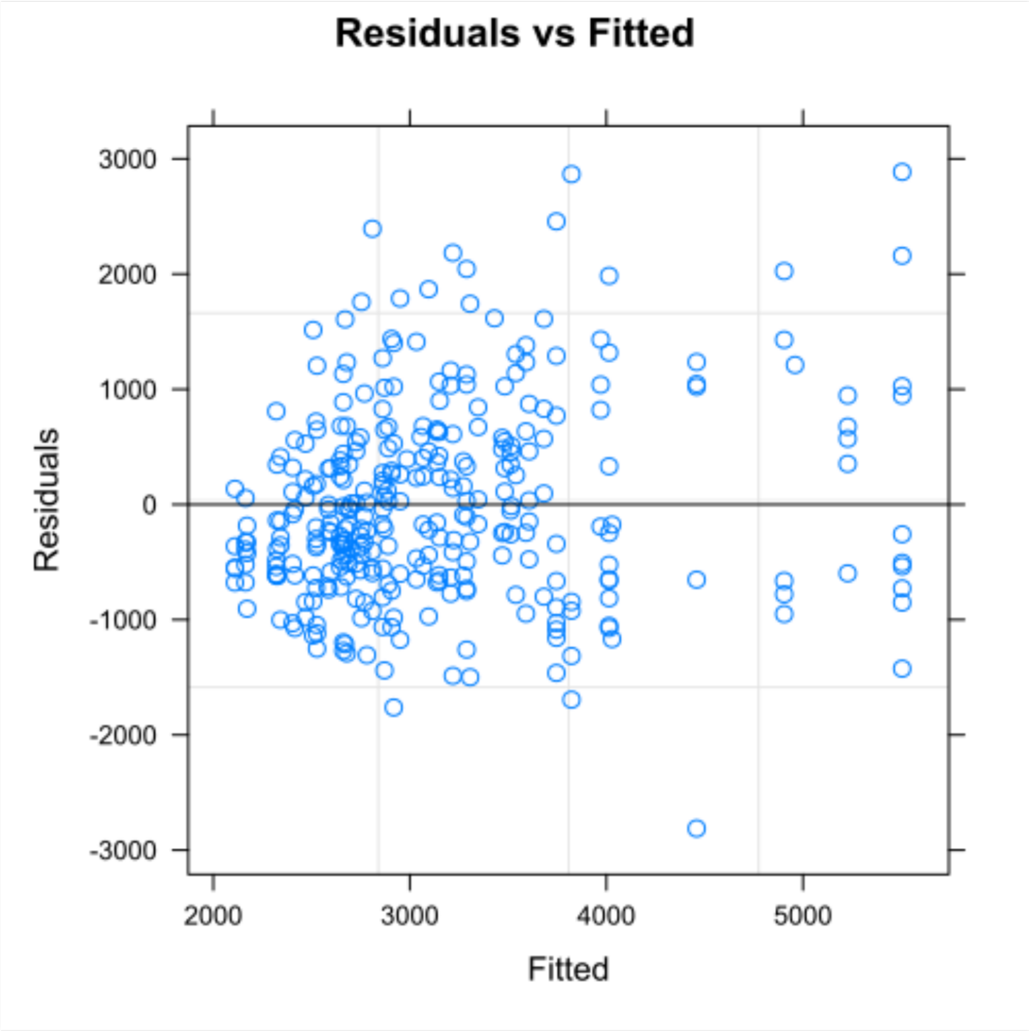

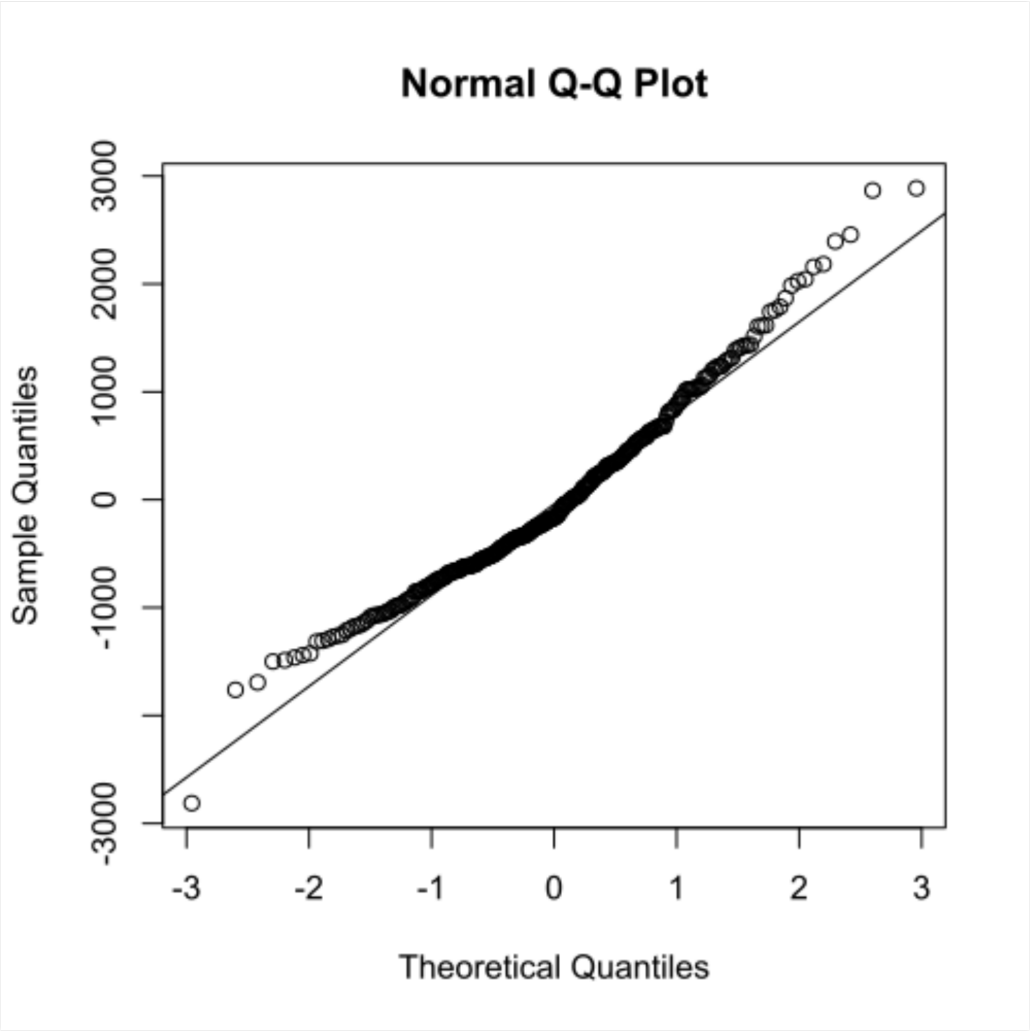

Leaf area (reproductive)

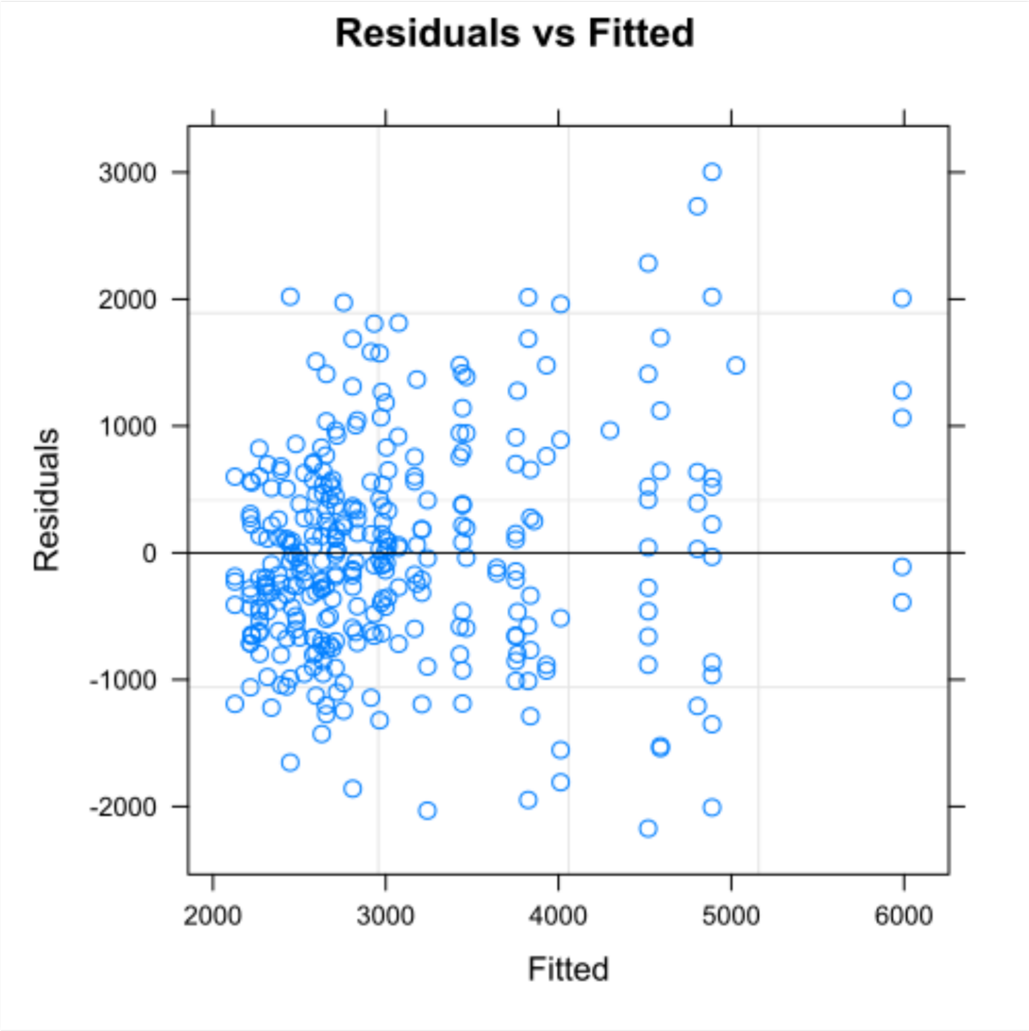

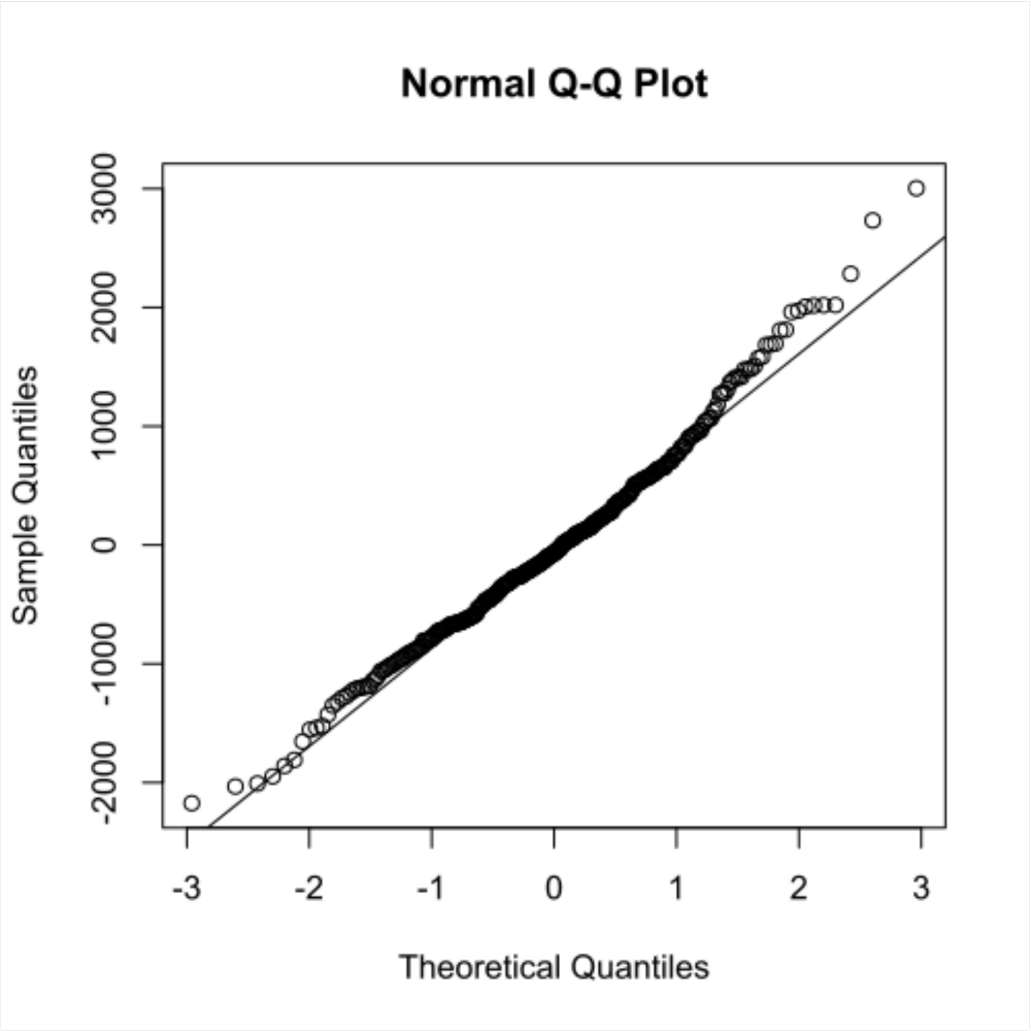

Specific leaf area (vegetative)

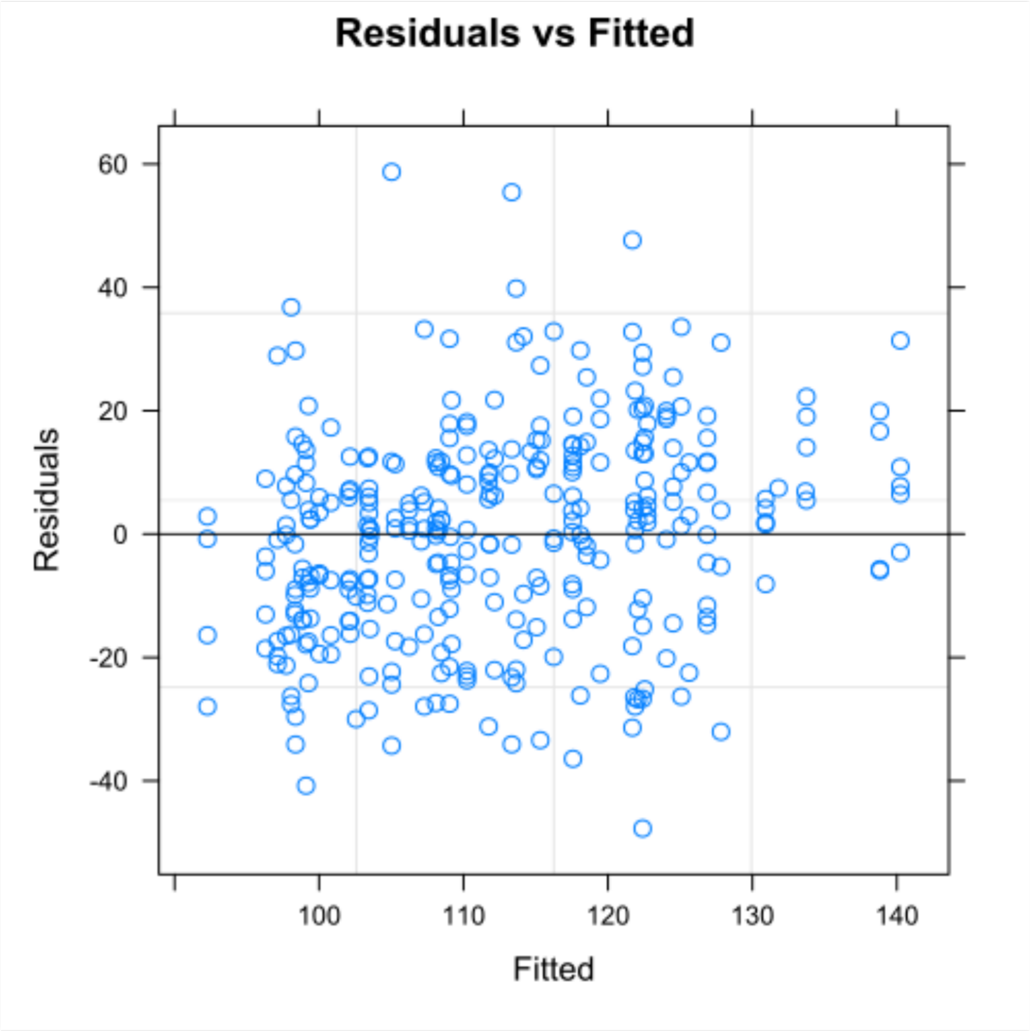

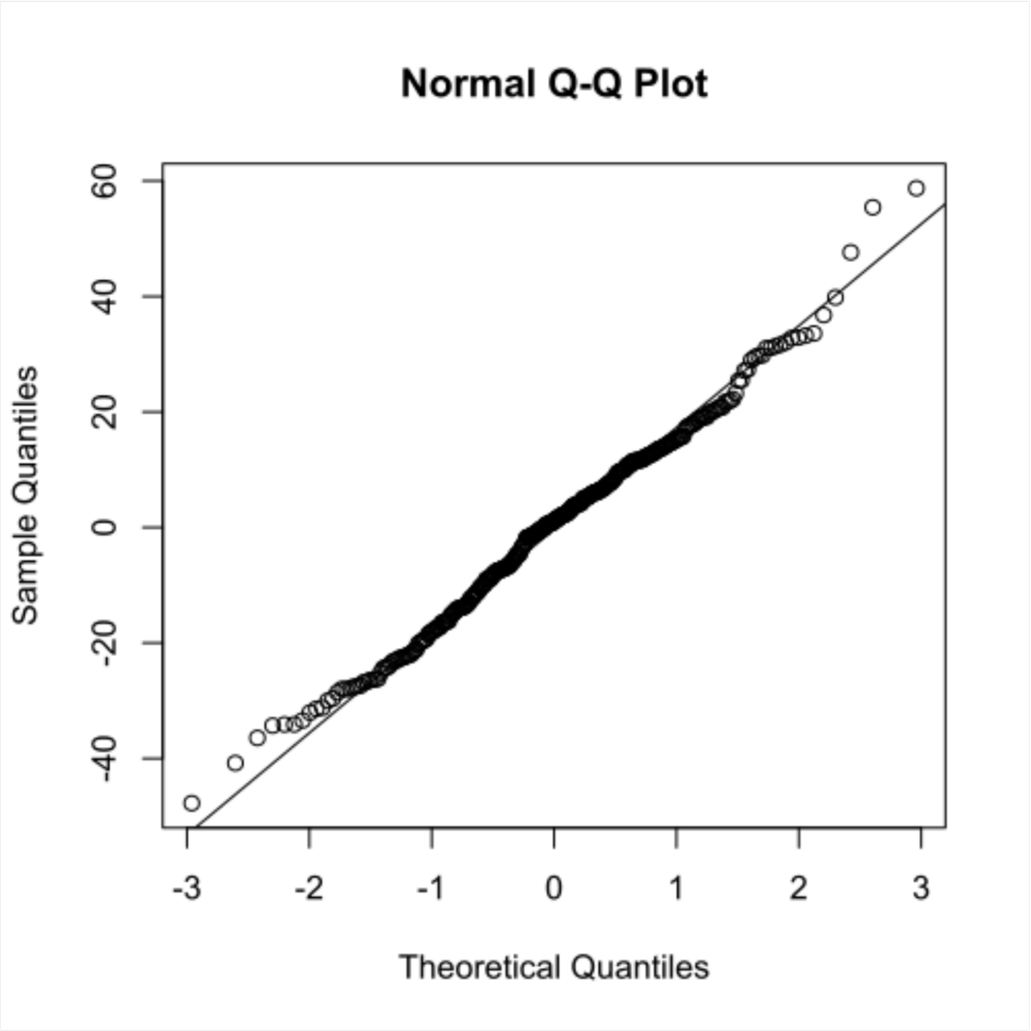

Specific leaf area (reproductive)

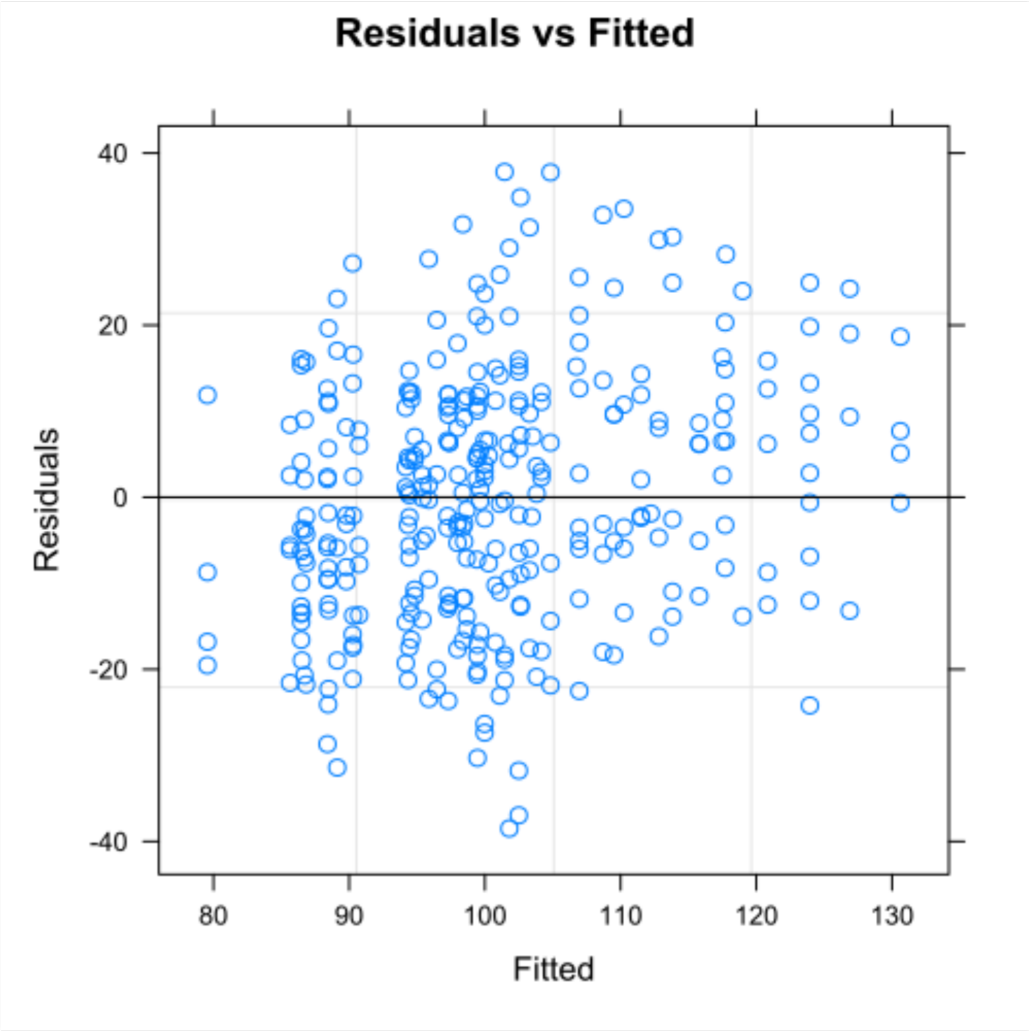

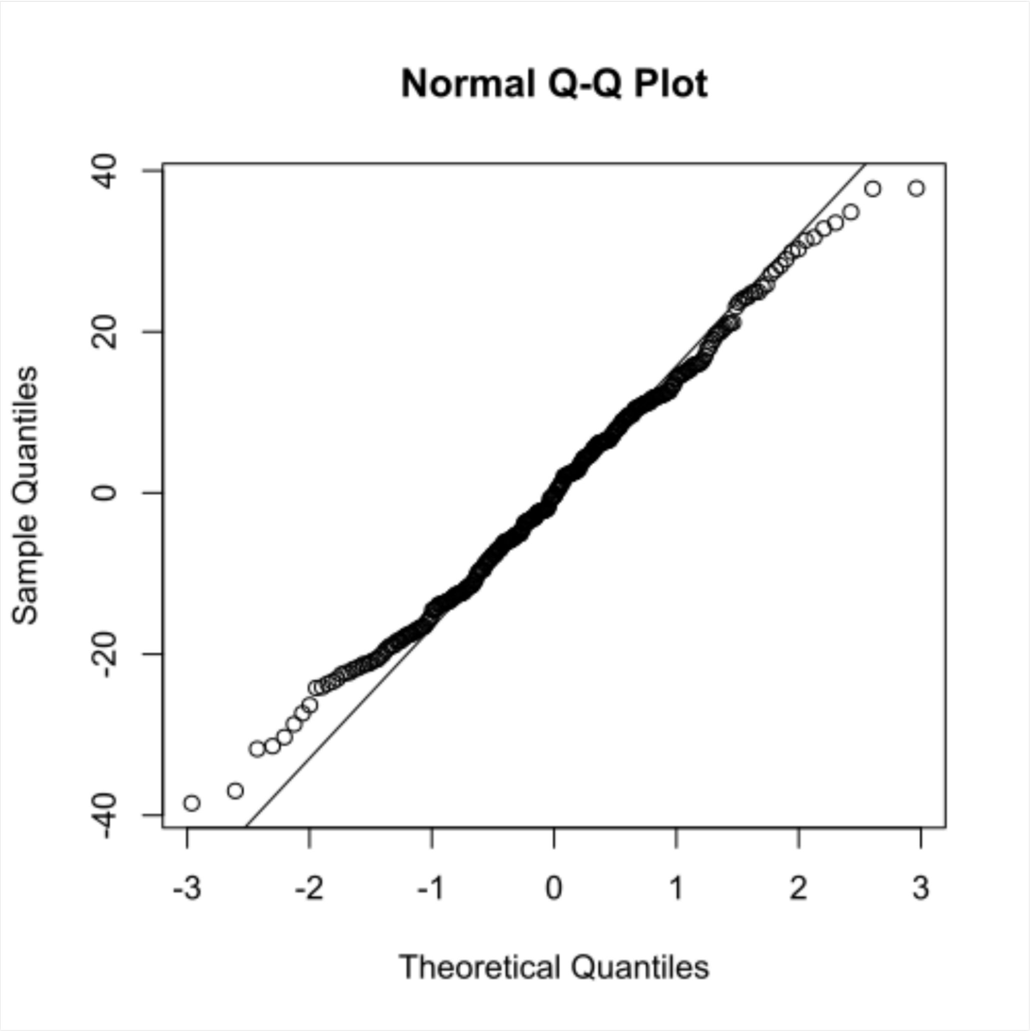

Leaf thickness (vegetative)

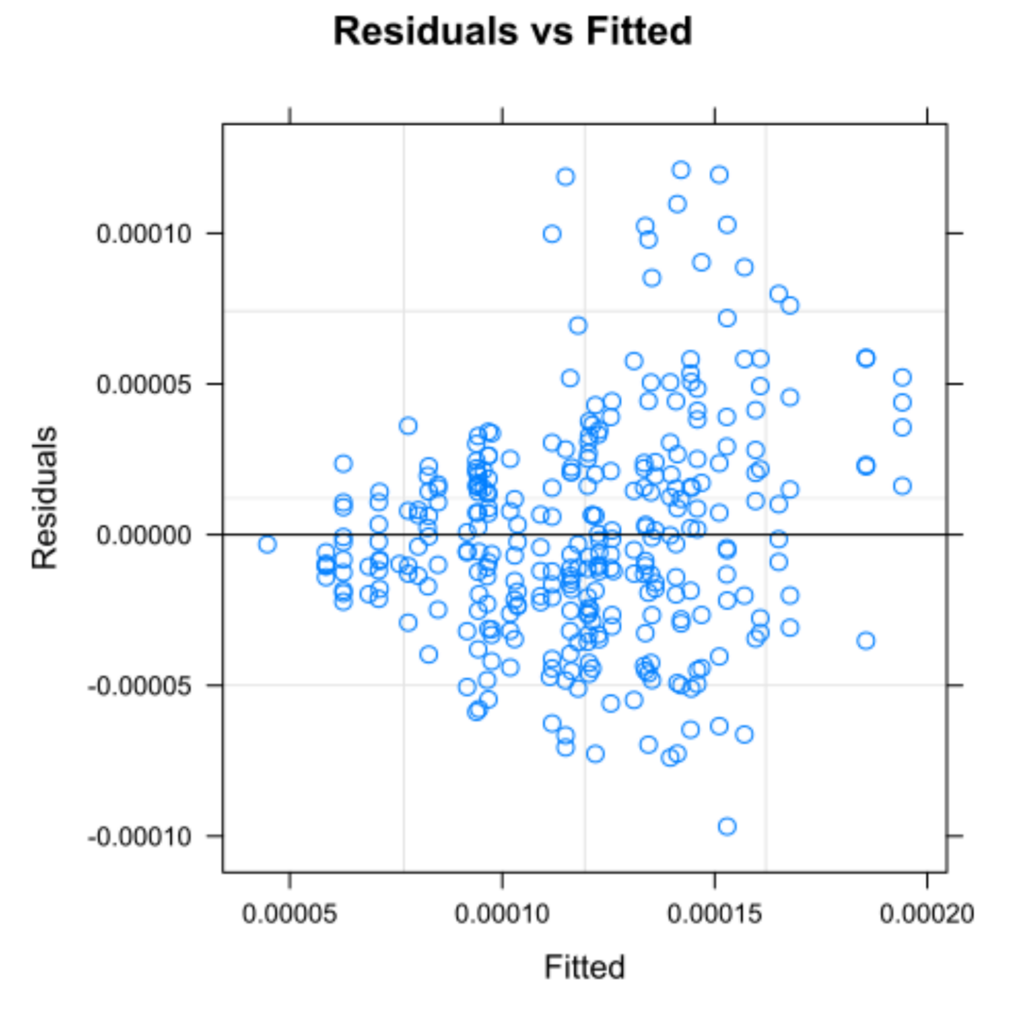

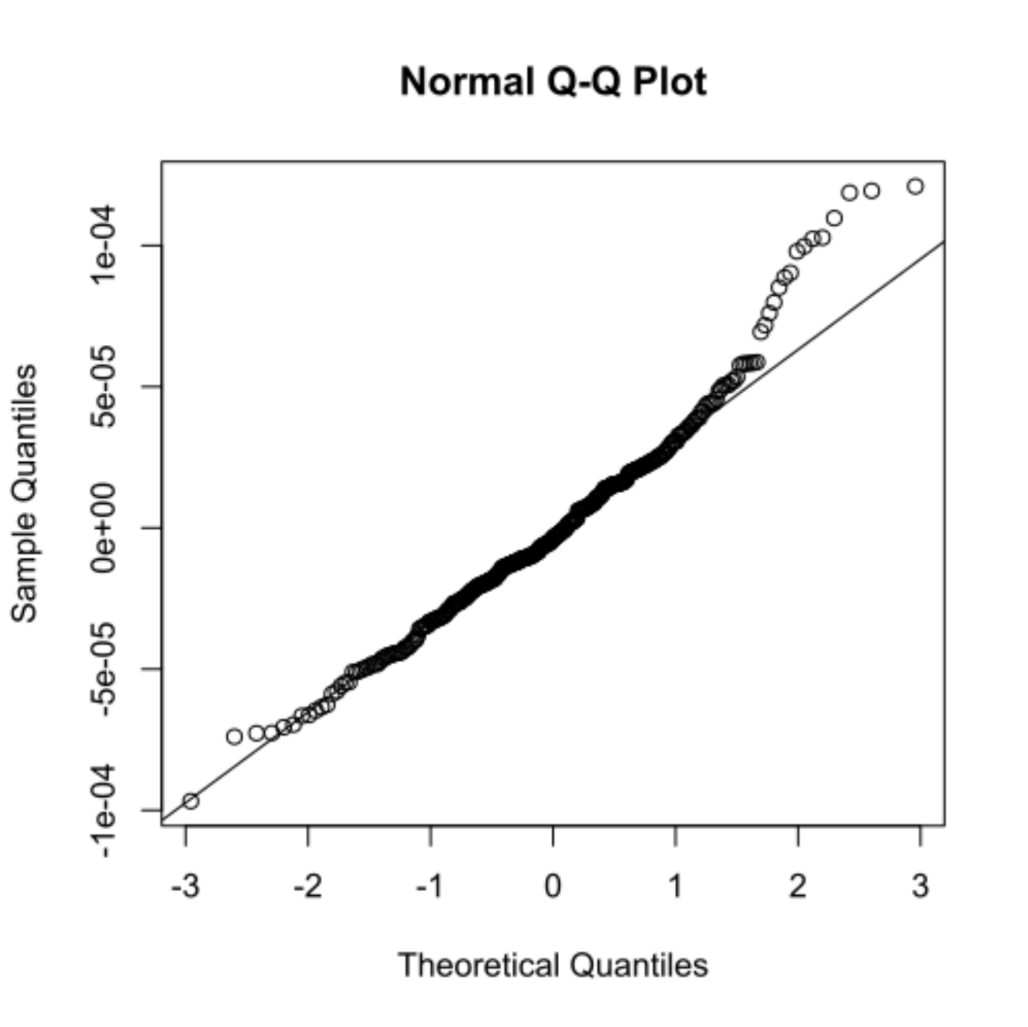

Leaf thickness (reproductive)

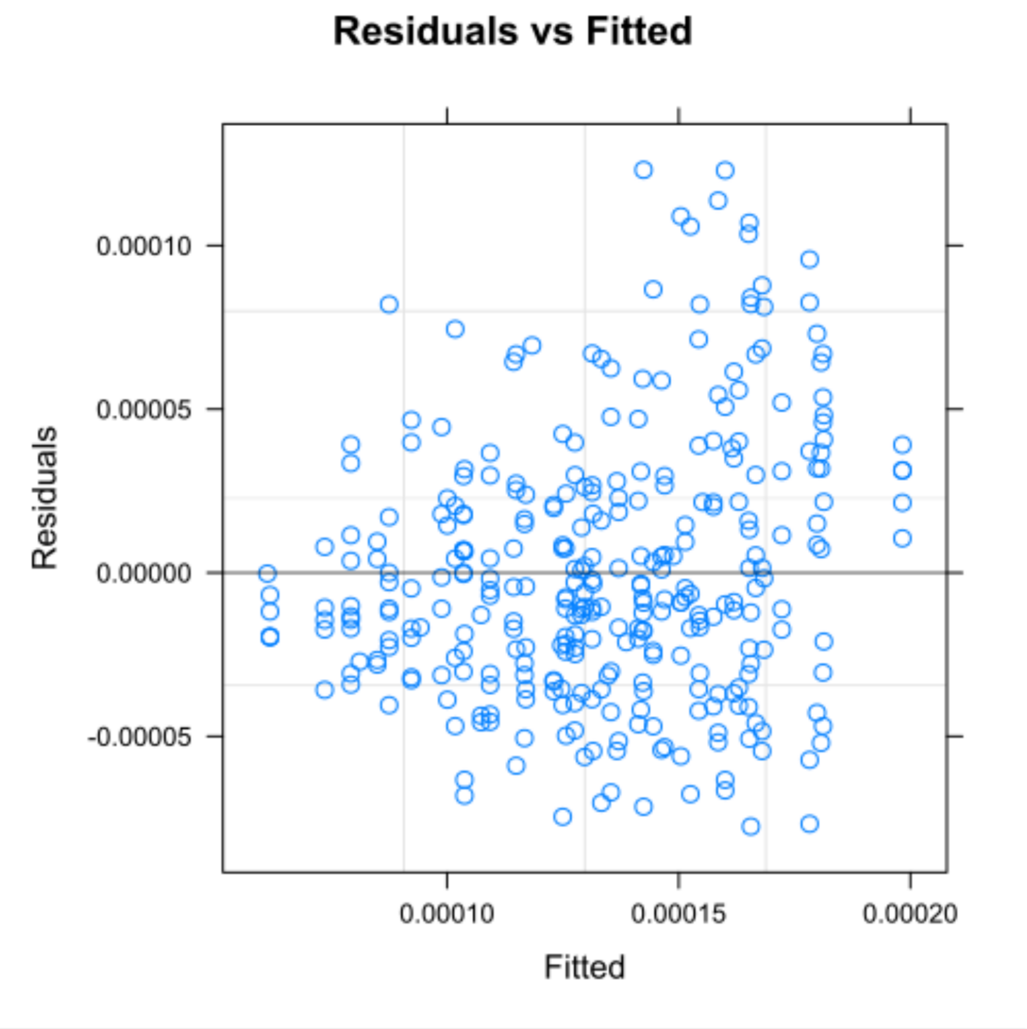

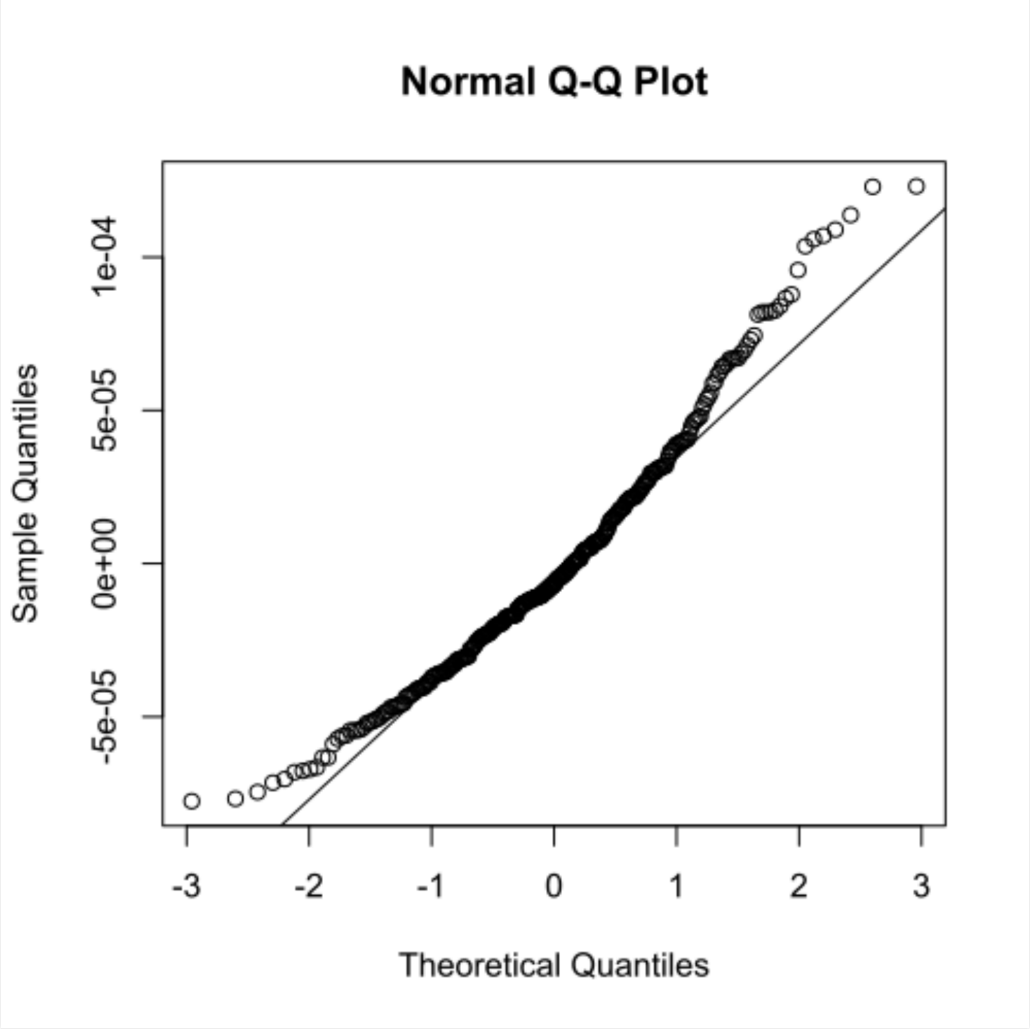

Supplement: Supplementary Figure 2 — Diagnostic plots of the mixed models of functional trait variables. [file Image_2.pdf]
