# Supplementary material for: Climatic niche pre-adaptation facilitated island colonization followed by budding speciation in the Madeiran ivy (Hedera maderensis, Araliaceae)
Source: Front Plant Sci. 2022 Jul 25;13:935975. doi: 10.3389/fpls.2022.935975 (PMC9358290; doi:10.3389/fpls.2022.935975)
Supplement: Supplementary Figure 4 — Consensus trees from the RAxML analyses of the 36 datasets. [file Image_4.pdf]

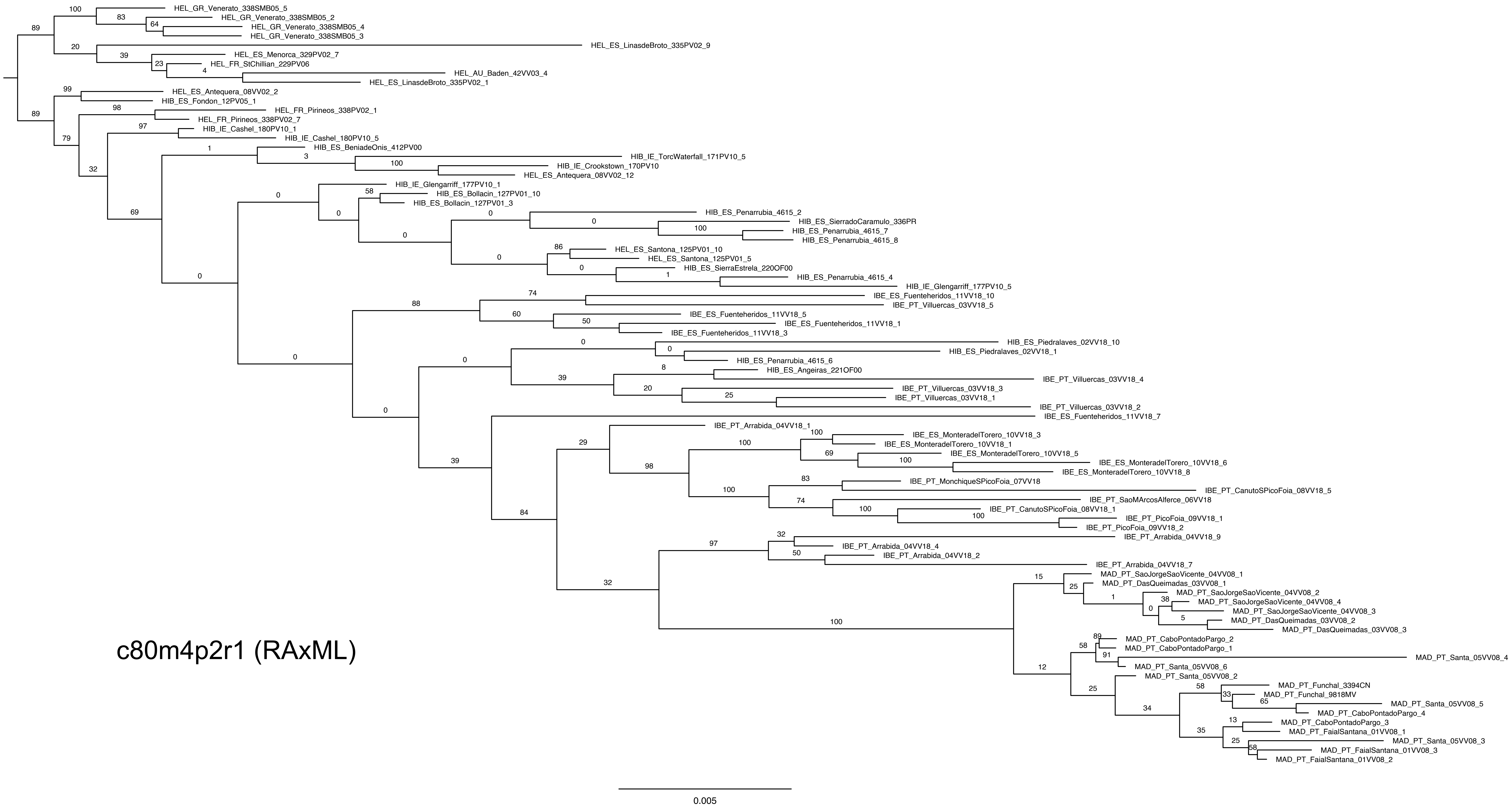
HEL_GR_Venerato_338SMB05_5
HEL_GR_Venerato_338SMB05_2
HEL_GR_Venerato_338SMB05_4
HEL_GR_Venerato_338SMB05_3
HEL_ES_LinasdeBroto_335PV02_9
HEL_ES_Menorca_329PV02_7
HEL_FR_StChillian_229PV06
HEL_AU_Baden_42VV03_4
HEL_ES_LinasdeBroto_335PV02_1
HEL_ES_Antequera_08VV02_2
HIB_ES_Fondon_12PV05_1
HEL_FR_Pirineos_338PV02_1
HEL_FR_Pirineos_338PV02_7
HIB_IE_Cashel_180PV10_1
HIB_IE_Cashel_180PV10_5
HIB_ES_BeniadeOnis_412PV00
HIB_IE_TorcWaterfall_171PV10_5
HIB_IE_Crookstown_170PV10
HEL_ES_Antequera_08VV02_12
HIB_IE_Glengarriff_177PV10_1
HIB_ES_Bollacin_127PV01_10
HIB_ES_Bollacin_127PV01_3
HIB_ES_Penarrubia_4615_2
HIB_ES_SierradoCaramulo_336PR
HIB_ES_Penarrubia_4615_7
HIB_ES_Penarrubia_4615_8
HEL_ES_Santona_125PV01_10
HEL_ES_Santona_125PV01_5
HIB_ES_SierraEstrela_220OF00
HIB_ES_Penarrubia_4615_4
HIB_IE_Glengarriff_177PV10_5
IBE_ES_Fuenteheridos_11VV18_10
IBE_PT_Villuercas_03VV18_5
IBE_ES_Fuenteheridos_11VV18_5
IBE_ES_Fuenteheridos_11VV18_1
IBE_ES_Fuenteheridos_11VV18_3
HIB_ES_Piedralaves_02VV18_10
HIB_ES_Piedralaves_02VV18_1
HIB_ES_Penarrubia_4615_6
HIB_ES_Angeiras_221OF00
IBE_PT_Villuercas_03VV18_4
IBE_PT_Villuercas_03VV18_3
IBE_PT_Villuercas_03VV18_1
IBE_PT_Villuercas_03VV18_2
IBE_ES_Fuenteheridos_11VV18_7
IBE_PT_Arrabida_04VV18_1
IBE_ES_MonteradelTorero_10VV18_3
IBE_ES_MonteradelTorero_10VV18_1
IBE_ES_MonteradelTorero_10VV18_5
IBE_ES_MonteradelTorero_10VV18_6
IBE_ES_MonteradelTorero_10VV18_8
IBE_PT_MonchiqueSPicoFoia_07VV18
IBE_PT_CanutoSPicoFoia_08VV18_5
IBE_PT_SaoMArcosAlferce_06VV18
IBE_PT_CanutoSPicoFoia_08VV18_1
IBE_PT_PicoFoia_09VV18_1
IBE_PT_PicoFoia_09VV18_2
IBE_PT_Arrabida_04VV18_9
IBE_PT_Arrabida_04VV18_4
IBE_PT_Arrabida_04VV18_2
IBE_PT_Arrabida_04VV18_7
MAD_PT_SaoJorgeSaoVicente_04VV08_1
MAD_PT_DasQueimadas_03VV08_1
MAD_PT_SaoJorgeSaoVicente_04VV08_2
MAD_PT_SaoJorgeSaoVicente_04VV08_4
MAD_PT_SaoJorgeSaoVicente_04VV08_3
MAD_PT_DasQueimadas_03VV08_2
MAD_PT_DasQueimadas_03VV08_3
MAD_PT_CaboPontadoPargo_2
MAD_PT_CaboPontadoPargo_1
MAD_PT_Santa_05VV08_4
MAD_PT_Santa_05VV08_6
MAD_PT_Santa_05VV08_2
MAD_PT_Funchal_3394CN
MAD_PT_Funchal_9818MV
MAD_PT_Santa_05VV08_5
MAD_PT_CaboPontadoPargo_4
MAD_PT_CaboPontadoPargo_3
MAD_PT_FaialSantana_01VV08_1
MAD_PT_Santa_05VV08_3
MAD_PT_FaialSantana_01VV08_3
MAD_PT_FaialSantana_01VV08_2
c80m4p2r1 (RAxML)
0.005

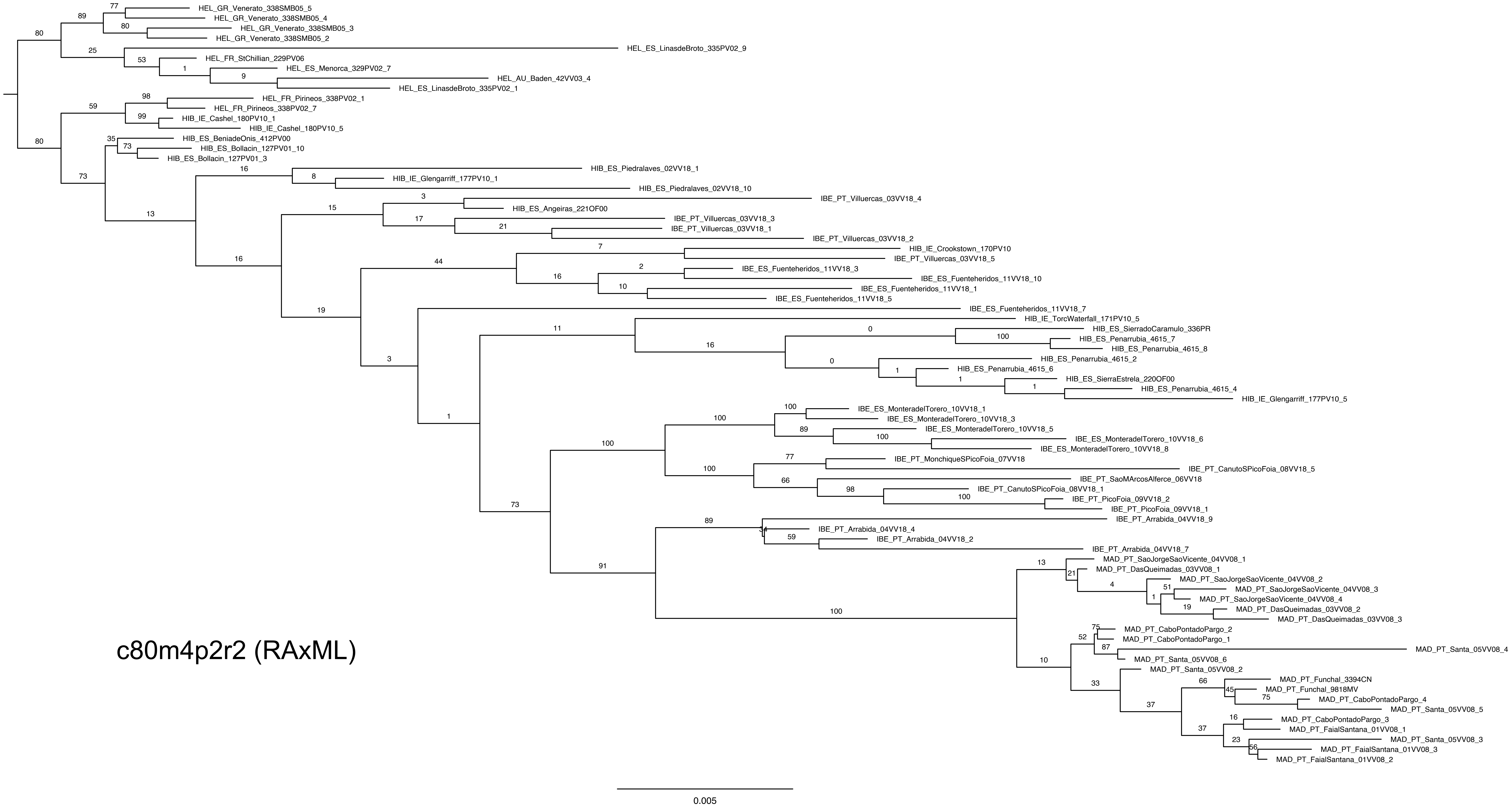
HEL_GR_Venerato_338SMB05_5
HEL_GR_Venerato_338SMB05_4
HEL_GR_Venerato_338SMB05_3
HEL_GR_Venerato_338SMB05_2
HEL_ES_LinasdeBroto_335PV02_9
HEL_FR_StChillian_229PV06
HEL_ES_Menorca_329PV02_7
HEL_AU_Baden_42VV03_4
HEL_ES_LinasdeBroto_335PV02_1
HEL_FR_Pirineos_338PV02_1
HEL_FR_Pirineos_338PV02_7
HIB_IE_Cashel_180PV10_1
HIB_IE_Cashel_180PV10_5
HIB_ES_BeniadeOnis_412PV00
HIB_ES_Bollacin_127PV01_10
HIB_ES_Bollacin_127PV01_3
HIB_ES_Piedralaves_02VV18_1
HIB_IE_Glengarriff_177PV10_1
HIB_ES_Piedralaves_02VV18_10
IBE_PT_Villuercas_03VV18_4
HIB_ES_Angeiras_221OF00
IBE_PT_Villuercas_03VV18_3
IBE_PT_Villuercas_03VV18_1
IBE_PT_Villuercas_03VV18_2
HIB_IE_Crookstown_170PV10
IBE_PT_Villuercas_03VV18_5
IBE_ES_Fuenteheridos_11VV18_3
IBE_ES_Fuenteheridos_11VV18_10
IBE_ES_Fuenteheridos_11VV18_1
IBE_ES_Fuenteheridos_11VV18_5
IBE_ES_Fuenteheridos_11VV18_7
HIB_IE_TorcWaterfall_171PV10_5
HIB_ES_SierradoCaramulo_336PR
HIB_ES_Penarrubia_4615_7
HIB_ES_Penarrubia_4615_8
HIB_ES_Penarrubia_4615_2
HIB_ES_Penarrubia_4615_6
HIB_ES_SierraEstrela_220OF00
HIB_ES_Penarrubia_4615_4
HIB_IE_Glengarriff_177PV10_5
IBE_ES_MonteradelTorero_10VV18_1
IBE_ES_MonteradelTorero_10VV18_3
IBE_ES_MonteradelTorero_10VV18_5
IBE_ES_MonteradelTorero_10VV18_6
IBE_ES_MonteradelTorero_10VV18_8
IBE_PT_MonchiqueSPicoFoia_07VV18
IBE_PT_CanutoSPicoFoia_08VV18_5
IBE_PT_SaoMArcosAlferce_06VV18
IBE_PT_CanutoSPicoFoia_08VV18_1
IBE_PT_PicoFoia_09VV18_2
IBE_PT_PicoFoia_09VV18_1
IBE_PT_Arrabida_04VV18_9
IBE_PT_Arrabida_04VV18_4
IBE_PT_Arrabida_04VV18_2
IBE_PT_Arrabida_04VV18_7
MAD_PT_SaoJorgeSaoVicente_04VV08_1
MAD_PT_DasQueimadas_03VV08_1
MAD_PT_SaoJorgeSaoVicente_04VV08_2
MAD_PT_SaoJorgeSaoVicente_04VV08_3
MAD_PT_SaoJorgeSaoVicente_04VV08_4
MAD_PT_DasQueimadas_03VV08_2
MAD_PT_DasQueimadas_03VV08_3
MAD_PT_CaboPontadoPargo_2
MAD_PT_CaboPontadoPargo_1
MAD_PT_Santa_05VV08_4
MAD_PT_Santa_05VV08_6
MAD_PT_Santa_05VV08_2
MAD_PT_Funchal_3394CN
MAD_PT_Funchal_9818MV
MAD_PT_CaboPontadoPargo_4
MAD_PT_Santa_05VV08_5
MAD_PT_CaboPontadoPargo_3
MAD_PT_FaialSantana_01VV08_1
MAD_PT_Santa_05VV08_3
MAD_PT_FaialSantana_01VV08_3
MAD_PT_FaialSantana_01VV08_2
c80m4p2r2 (RAxML)
0.005

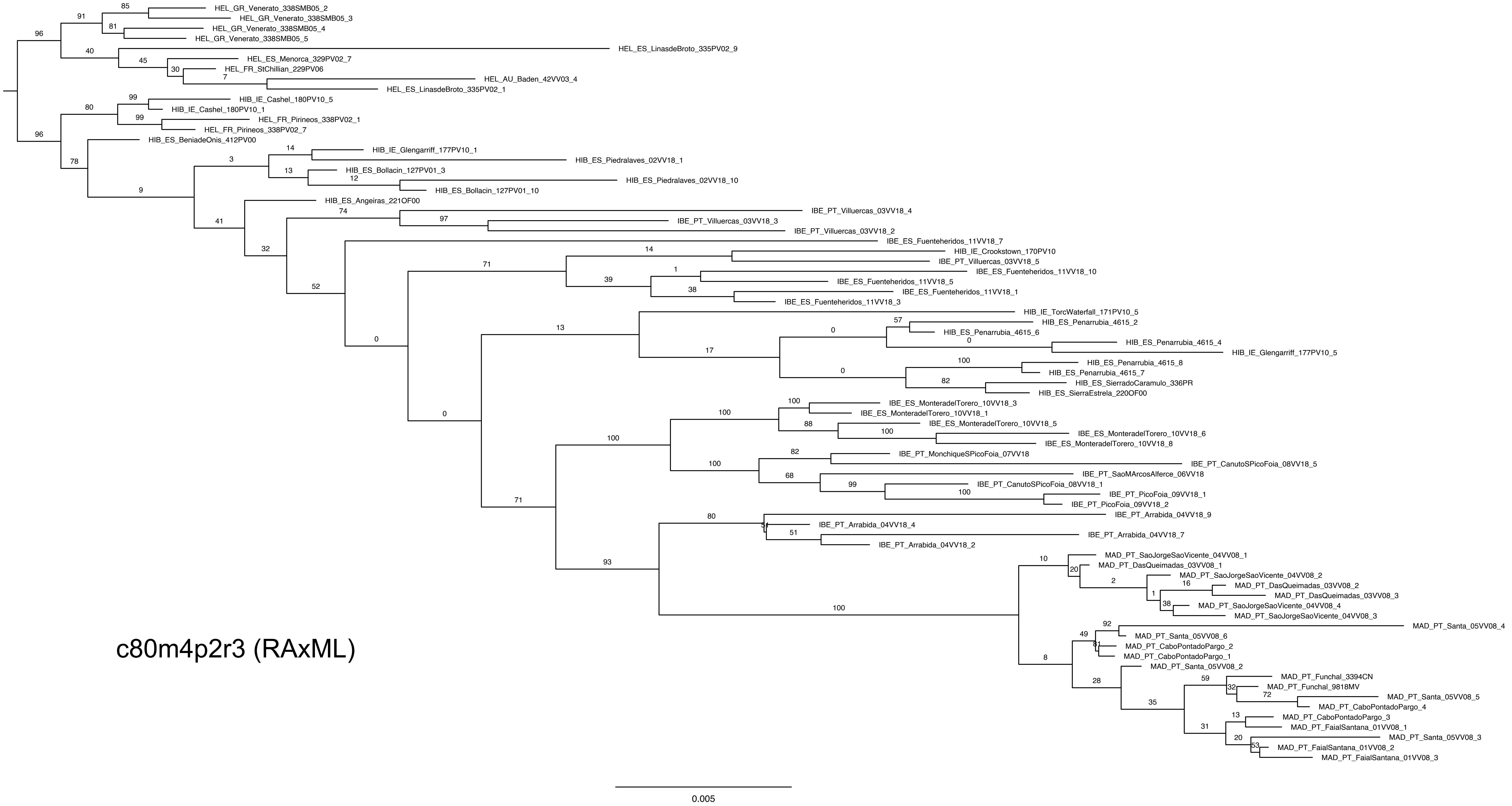
HEL_GR_Venerato_338SMB05_2
HEL_GR_Venerato_338SMB05_3
HEL_GR_Venerato_338SMB05_4
HEL_GR_Venerato_338SMB05_5
HEL_ES_LinasdeBroto_335PV02_9
HEL_ES_Menorca_329PV02_7
HEL_FR_StChillian_229PV06
HEL_AU_Baden_42VV03_4
HEL_ES_LinasdeBroto_335PV02_1
HIB_IE_Cashel_180PV10_5
HIB_IE_Cashel_180PV10_1
HEL_FR_Pirineos_338PV02_1
HEL_FR_Pirineos_338PV02_7
HIB_ES_BeniadeOnis_412PV00
HIB_IE_Glengarriff_177PV10_1
HIB_ES_Piedralaves_02VV18_1
HIB_ES_Bollacin_127PV01_3
HIB_ES_Piedralaves_02VV18_10
HIB_ES_Bollacin_127PV01_10
HIB_ES_Angeiras_221OF00
IBE_PT_Villuercas_03VV18_4
IBE_PT_Villuercas_03VV18_3
IBE_PT_Villuercas_03VV18_2
IBE_ES_Fuenteheridos_11VV18_7
HIB_IE_Crookstown_170PV10
IBE_PT_Villuercas_03VV18_5
IBE_ES_Fuenteheridos_11VV18_10
IBE_ES_Fuenteheridos_11VV18_5
IBE_ES_Fuenteheridos_11VV18_1
IBE_ES_Fuenteheridos_11VV18_3
HIB_IE_TorcWaterfall_171PV10_5
HIB_ES_Penarrubia_4615_2
HIB_ES_Penarrubia_4615_6
HIB_ES_Penarrubia_4615_4
HIB_IE_Glengarriff_177PV10_5
HIB_ES_Penarrubia_4615_8
HIB_ES_Penarrubia_4615_7
HIB_ES_SierradoCaramulo_336PR
HIB_ES_SierraEstrela_220OF00
IBE_ES_MonteradelTorero_10VV18_3
IBE_ES_MonteradelTorero_10VV18_1
IBE_ES_MonteradelTorero_10VV18_5
IBE_ES_MonteradelTorero_10VV18_6
IBE_ES_MonteradelTorero_10VV18_8
IBE_PT_MonchiqueSPicoFoia_07VV18
IBE_PT_CanutoSPicoFoia_08VV18_5
IBE_PT_SaoMArcosAlferce_06VV18
IBE_PT_CanutoSPicoFoia_08VV18_1
IBE_PT_PicoFoia_09VV18_1
IBE_PT_PicoFoia_09VV18_2
IBE_PT_Arrabida_04VV18_9
IBE_PT_Arrabida_04VV18_4
IBE_PT_Arrabida_04VV18_7
IBE_PT_Arrabida_04VV18_2
MAD_PT_SaoJorgeSaoVicente_04VV08_1
MAD_PT_DasQueimadas_03VV08_1
MAD_PT_SaoJorgeSaoVicente_04VV08_2
MAD_PT_DasQueimadas_03VV08_2
MAD_PT_DasQueimadas_03VV08_3
MAD_PT_SaoJorgeSaoVicente_04VV08_4
MAD_PT_SaoJorgeSaoVicente_04VV08_3
MAD_PT_Santa_05VV08_4
MAD_PT_Santa_05VV08_6
MAD_PT_CaboPontadoPargo_2
MAD_PT_CaboPontadoPargo_1
MAD_PT_Santa_05VV08_2
MAD_PT_Funchal_3394CN
MAD_PT_Funchal_9818MV
MAD_PT_Santa_05VV08_5
MAD_PT_CaboPontadoPargo_4
MAD_PT_CaboPontadoPargo_3
MAD_PT_FaialSantana_01VV08_1
MAD_PT_Santa_05VV08_3
MAD_PT_FaialSantana_01VV08_2
MAD_PT_FaialSantana_01VV08_3
c80m4p2r3 (RAxML)
0.005

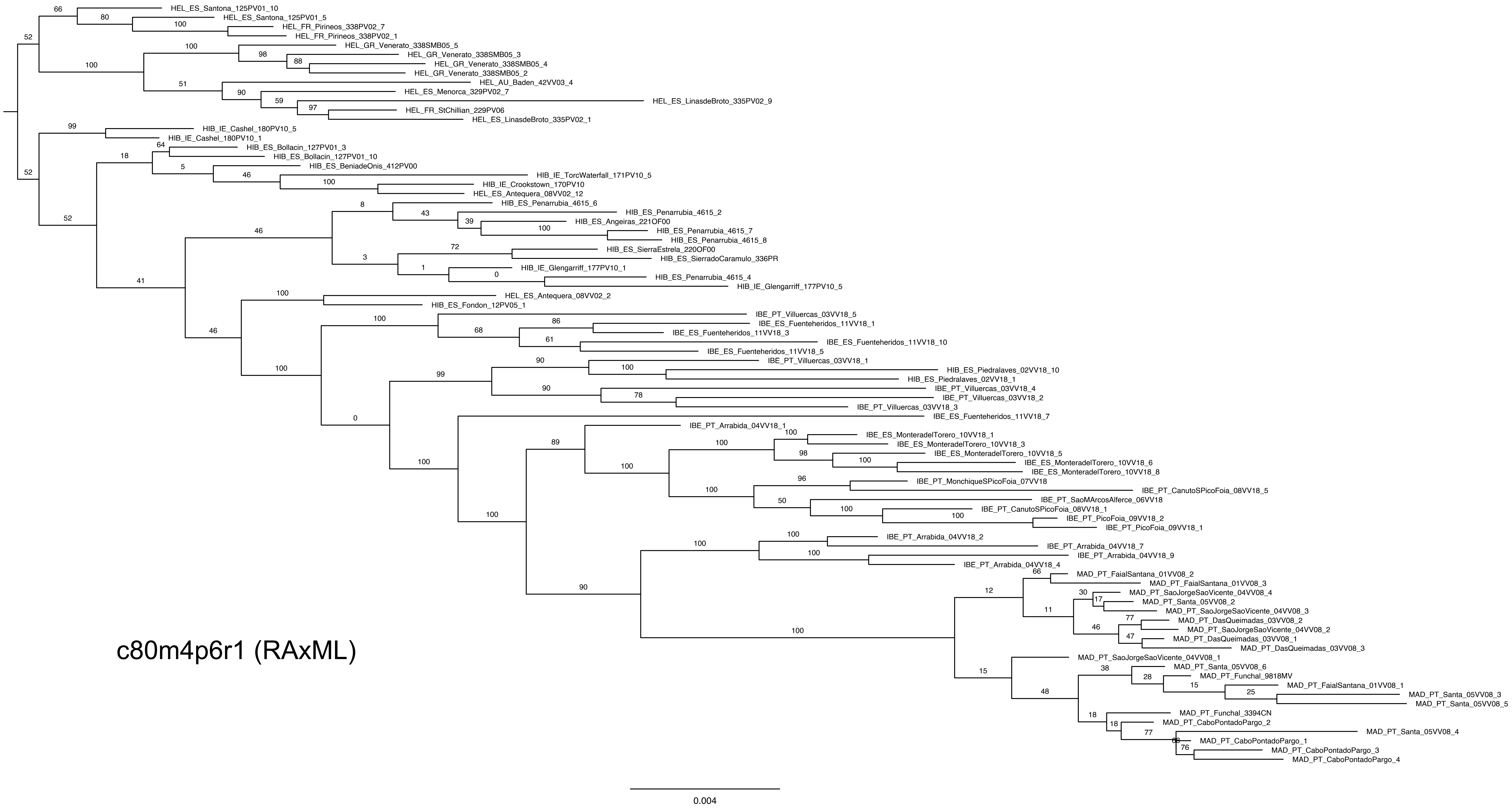

HEL_ES_Santona_125PV01_10
HEL_ES_Santona_125PV01_5
HEL_FR_Pirineos_338PV02_7
HEL_FR_Pirineos_338PV02_1
HEL_GR_Venerato_338SMB05_5
HEL_GR_Venerato_338SMB05_3
HEL_GR_Venerato_338SMB05_4
HEL_GR_Venerato_338SMB05_2
HEL_AU_Baden_42VV03_4
HEL_ES_Menorca_329PV02_7
HEL_ES_LinasdeBroto_335PV02_9
HEL_FR_StChillian_229PV06
HEL_ES_LinasdeBroto_335PV02_1
HIB_IE_Cashel_180PV10_5
HIB_IE_Cashel_180PV10_1
HIB_ES_Bollacin_127PV01_3
HIB_ES_Bollacin_127PV01_10
HIB_ES_BeniadeOnis_412PV00
HIB_IE_TorcWaterfall_171PV10_5
HIB_IE_Crookstown_170PV10
HEL_ES_Antequera_08VV02_12
HIB_ES_Penarrubia_4615_6
HIB_ES_Penarrubia_4615_2
HIB_ES_Angeiras_221OF00
HIB_ES_Penarrubia_4615_7
HIB_ES_Penarrubia_4615_8
HIB_ES_SierraEstrela_220OF00
HIB_ES_SierradoCaramulo_336PR
HIB_IE_Glengarriff_177PV10_1
HIB_ES_Penarrubia_4615_4
HIB_IE_Glengarriff_177PV10_5
HEL_ES_Antequera_08VV02_2
HIB_ES_Fondon_12PV05_1
IBE_PT_Villuercas_03VV18_5
IBE_ES_Fuenteheridos_11VV18_1
IBE_ES_Fuenteheridos_11VV18_3
IBE_ES_Fuenteheridos_11VV18_10
IBE_ES_Fuenteheridos_11VV18_5
IBE_PT_Villuercas_03VV18_1
HIB_ES_Piedralaves_02VV18_10
HIB_ES_Piedralaves_02VV18_1
IBE_PT_Villuercas_03VV18_4
IBE_PT_Villuercas_03VV18_2
IBE_PT_Villuercas_03VV18_3
IBE_ES_Fuenteheridos_11VV18_7
IBE_PT_Arrabida_04VV18_1
IBE_ES_MonteradelTorero_10VV18_1
IBE_ES_MonteradelTorero_10VV18_3
IBE_ES_MonteradelTorero_10VV18_5
IBE_ES_MonteradelTorero_10VV18_6
IBE_ES_MonteradelTorero_10VV18_8
IBE_PT_MonchiqueSPicoFoia_07VV18
IBE_PT_CanutoSPicoFoia_08VV18_5
IBE_PT_SaoMArcosAlferce_06VV18
IBE_PT_CanutoSPicoFoia_08VV18_1
IBE_PT_PicoFoia_09VV18_2
IBE_PT_PicoFoia_09VV18_1
IBE_PT_Arrabida_04VV18_2
IBE_PT_Arrabida_04VV18_7
IBE_PT_Arrabida_04VV18_9
IBE_PT_Arrabida_04VV18_4
MAD_PT_FaialSantana_01VV08_2
MAD_PT_FaialSantana_01VV08_3
MAD_PT_SaoJorgeSaoVicente_04VV08_4
MAD_PT_Santa_05VV08_2
MAD_PT_SaoJorgeSaoVicente_04VV08_3
MAD_PT_DasQueimadas_03VV08_2
MAD_PT_SaoJorgeSaoVicente_04VV08_2
MAD_PT_DasQueimadas_03VV08_1
MAD_PT_DasQueimadas_03VV08_3
MAD_PT_SaoJorgeSaoVicente_04VV08_1
MAD_PT_Santa_05VV08_6
MAD_PT_Funchal_9818MV
MAD_PT_FaialSantana_01VV08_1
MAD_PT_Santa_05VV08_3
MAD_PT_Santa_05VV08_5
MAD_PT_Funchal_3394CN
MAD_PT_CaboPontadoPargo_2
MAD_PT_Santa_05VV08_4
MAD_PT_CaboPontadoPargo_1
MAD_PT_CaboPontadoPargo_3
MAD_PT_CaboPontadoPargo_4
c80m4p6r1 (RAxML)
0.004

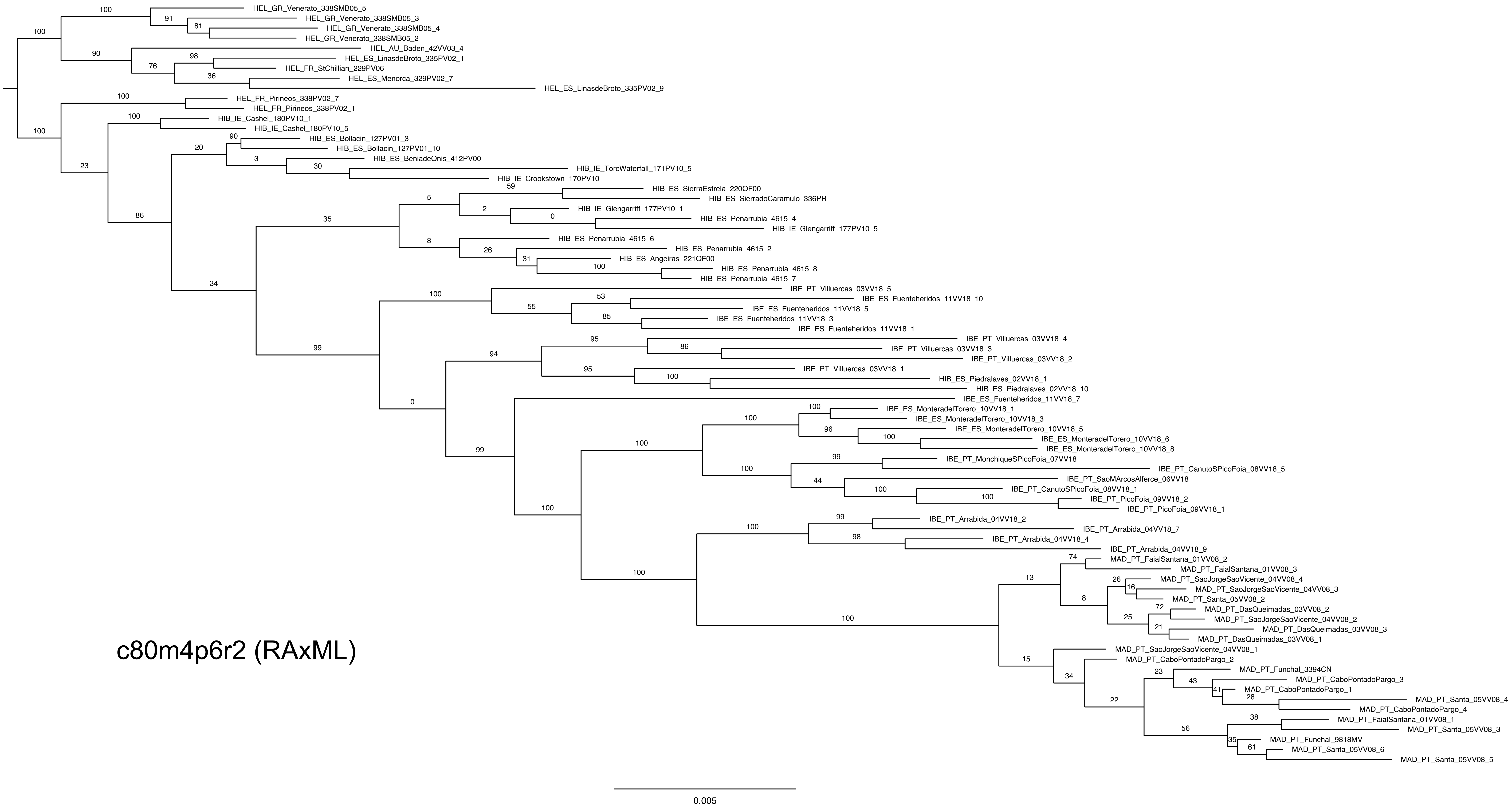
HEL_GR_Venerato_338SMB05_5
HEL_GR_Venerato_338SMB05_3
HEL_GR_Venerato_338SMB05_4
HEL_GR_Venerato_338SMB05_2
HEL_AU_Baden_42VV03_4
HEL_ES_LinasdeBroto_335PV02_1
HEL_FR_StChillian_229PV06
HEL_ES_Menorca_329PV02_7
HEL_ES_LinasdeBroto_335PV02_9
HEL_FR_Pirineos_338PV02_7
HEL_FR_Pirineos_338PV02_1
HIB_IE_Cashel_180PV10_1
HIB_IE_Cashel_180PV10_5
HIB_ES_Bollacin_127PV01_3
HIB_ES_Bollacin_127PV01_10
HIB_ES_BeniadeOnis_412PV00
HIB_IE_TorcWaterfall_171PV10_5
HIB_IE_Crookstown_170PV10
HIB_ES_SierraEstrela_220OF00
HIB_ES_SierradoCaramulo_336PR
HIB_IE_Glengarriff_177PV10_1
HIB_ES_Penarrubia_4615_4
HIB_IE_Glengarriff_177PV10_5
HIB_ES_Penarrubia_4615_6
HIB_ES_Penarrubia_4615_2
HIB_ES_Angeiras_221OF00
HIB_ES_Penarrubia_4615_8
HIB_ES_Penarrubia_4615_7
IBE_PT_Villuercas_03VV18_5
IBE_ES_Fuenteheridos_11VV18_10
IBE_ES_Fuenteheridos_11VV18_5
IBE_ES_Fuenteheridos_11VV18_3
IBE_ES_Fuenteheridos_11VV18_1
IBE_PT_Villuercas_03VV18_4
IBE_PT_Villuercas_03VV18_3
IBE_PT_Villuercas_03VV18_2
IBE_PT_Villuercas_03VV18_1
HIB_ES_Piedralaves_02VV18_1
HIB_ES_Piedralaves_02VV18_10
IBE_ES_Fuenteheridos_11VV18_7
IBE_ES_MonteradelTorero_10VV18_1
IBE_ES_MonteradelTorero_10VV18_3
IBE_ES_MonteradelTorero_10VV18_5
IBE_ES_MonteradelTorero_10VV18_6
IBE_ES_MonteradelTorero_10VV18_8
IBE_PT_MonchiqueSPicoFoia_07VV18
IBE_PT_CanutoSPicoFoia_08VV18_5
IBE_PT_SaoMArcosAlferce_06VV18
IBE_PT_CanutoSPicoFoia_08VV18_1
IBE_PT_PicoFoia_09VV18_2
IBE_PT_PicoFoia_09VV18_1
IBE_PT_Arrabida_04VV18_2
IBE_PT_Arrabida_04VV18_7
IBE_PT_Arrabida_04VV18_4
IBE_PT_Arrabida_04VV18_9
MAD_PT_FaialSantana_01VV08_2
MAD_PT_FaialSantana_01VV08_3
MAD_PT_SaoJorgeSaoVicente_04VV08_4
MAD_PT_SaoJorgeSaoVicente_04VV08_3
MAD_PT_Santa_05VV08_2
MAD_PT_DasQueimadas_03VV08_2
MAD_PT_SaoJorgeSaoVicente_04VV08_2
MAD_PT_DasQueimadas_03VV08_3
MAD_PT_DasQueimadas_03VV08_1
MAD_PT_SaoJorgeSaoVicente_04VV08_1
MAD_PT_CaboPontadoPargo_2
MAD_PT_Funchal_3394CN
MAD_PT_CaboPontadoPargo_3
MAD_PT_CaboPontadoPargo_1
MAD_PT_Santa_05VV08_4
MAD_PT_CaboPontadoPargo_4
MAD_PT_FaialSantana_01VV08_1
MAD_PT_Santa_05VV08_3
MAD_PT_Funchal_9818MV
MAD_PT_Santa_05VV08_6
MAD_PT_Santa_05VV08_5
c80m4p6r2 (RAxML)
0.005

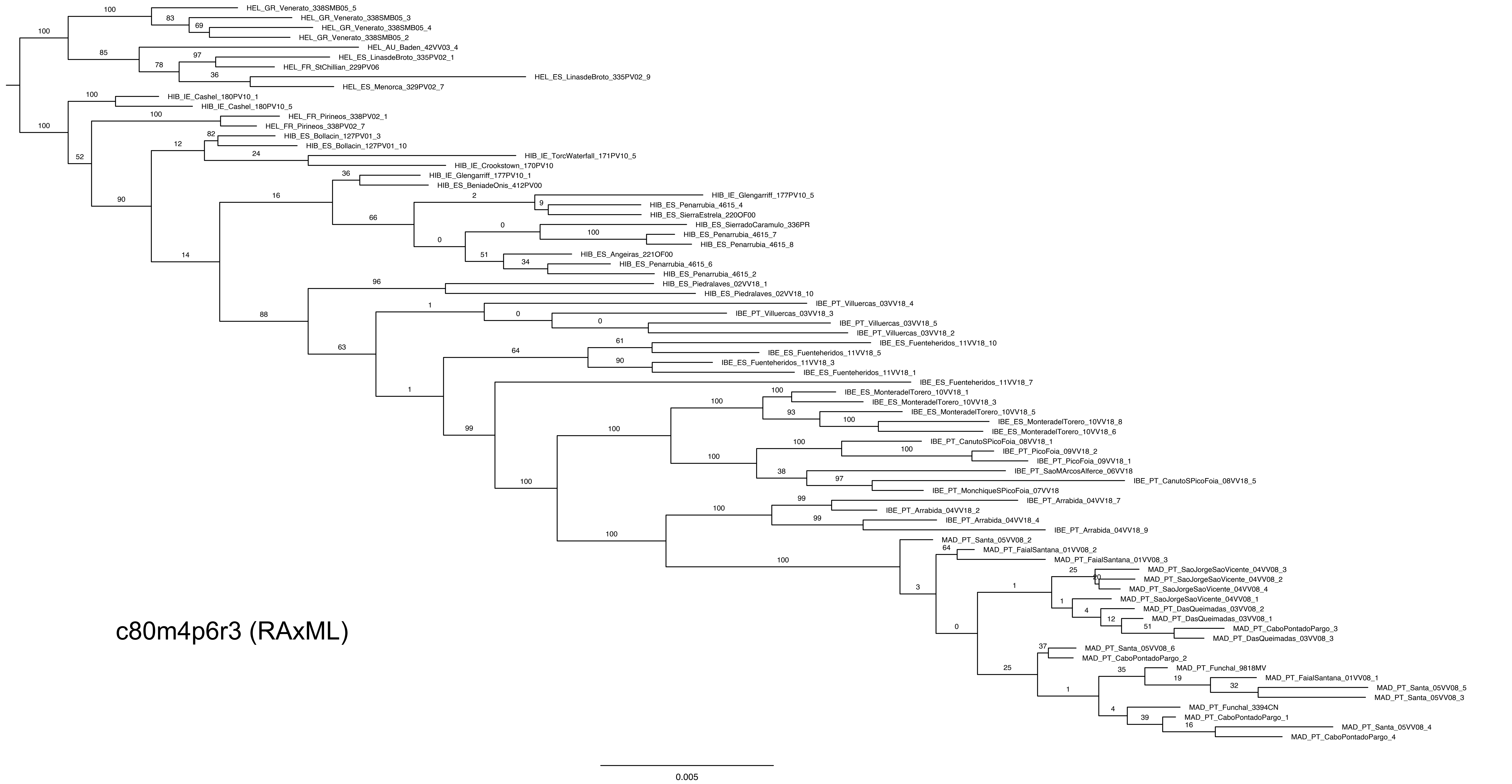
c80m4p6r3 (RAxML)
HEL_GR_Venerato_338SMB05_5
HEL_GR_Venerato_338SMB05_3
HEL_GR_Venerato_338SMB05_4
HEL_GR_Venerato_338SMB05_2
HEL_AU_Baden_42VV03_4
HEL_ES_LinasdeBroto_335PV02_1
HEL_FR_StChillian_229PV06
HEL_ES_LinasdeBroto_335PV02_9
HEL_ES_Menorca_329PV02_7
HIB_IE_Cashel_180PV10_1
HIB_IE_Cashel_180PV10_5
HEL_FR_Pirineos_338PV02_1
HEL_FR_Pirineos_338PV02_7
HIB_ES_Bollacin_127PV01_3
HIB_ES_Bollacin_127PV01_10
HIB_IE_TorcWaterfall_171PV10_5
HIB_IE_Crookstown_170PV10
HIB_IE_Glengarriff_177PV10_1
HIB_ES_BeniadeOnis_412PV00
HIB_IE_Glengarriff_177PV10_5
HIB_ES_Penarrubia_4615_4
HIB_ES_SierraEstrela_220OF00
HIB_ES_SierradoCaramulo_336PR
HIB_ES_Penarrubia_4615_7
HIB_ES_Penarrubia_4615_8
HIB_ES_Angeiras_221OF00
HIB_ES_Penarrubia_4615_6
HIB_ES_Penarrubia_4615_2
HIB_ES_Piedralaves_02VV18_1
HIB_ES_Piedralaves_02VV18_10
IBE_PT_Villuercas_03VV18_4
IBE_PT_Villuercas_03VV18_3
IBE_PT_Villuercas_03VV18_5
IBE_PT_Villuercas_03VV18_2
IBE_ES_Fuenteheridos_11VV18_10
IBE_ES_Fuenteheridos_11VV18_5
IBE_ES_Fuenteheridos_11VV18_3
IBE_ES_Fuenteheridos_11VV18_1
IBE_ES_Fuenteheridos_11VV18_7
IBE_ES_MonteradelTorero_10VV18_1
IBE_ES_MonteradelTorero_10VV18_3
IBE_ES_MonteradelTorero_10VV18_5
IBE_ES_MonteradelTorero_10VV18_8
IBE_ES_MonteradelTorero_10VV18_6
IBE_PT_CanutoSPicoFoia_08VV18_1
IBE_PT_PicoFoia_09VV18_2
IBE_PT_PicoFoia_09VV18_1
IBE_PT_SaoMArcosAlferce_06VV18
IBE_PT_CanutoSPicoFoia_08VV18_5
IBE_PT_MonchiqueSPicoFoia_07VV18
IBE_PT_Arrabida_04VV18_7
IBE_PT_Arrabida_04VV18_2
IBE_PT_Arrabida_04VV18_4
IBE_PT_Arrabida_04VV18_9
MAD_PT_Santa_05VV08_2
MAD_PT_FaialSantana_01VV08_2
MAD_PT_FaialSantana_01VV08_3
MAD_PT_SaoJorgeSaoVicente_04VV08_3
MAD_PT_SaoJorgeSaoVicente_04VV08_2
MAD_PT_SaoJorgeSaoVicente_04VV08_4
MAD_PT_SaoJorgeSaoVicente_04VV08_1
MAD_PT_DasQueimadas_03VV08_2
MAD_PT_DasQueimadas_03VV08_1
MAD_PT_CaboPontadoPargo_3
MAD_PT_DasQueimadas_03VV08_3
MAD_PT_Santa_05VV08_6
MAD_PT_CaboPontadoPargo_2
MAD_PT_Funchal_9818MV
MAD_PT_FaialSantana_01VV08_1
MAD_PT_Santa_05VV08_5
MAD_PT_Santa_05VV08_3
MAD_PT_Funchal_3394CN
MAD_PT_CaboPontadoPargo_1
MAD_PT_Santa_05VV08_4
MAD_PT_CaboPontadoPargo_4
0.005

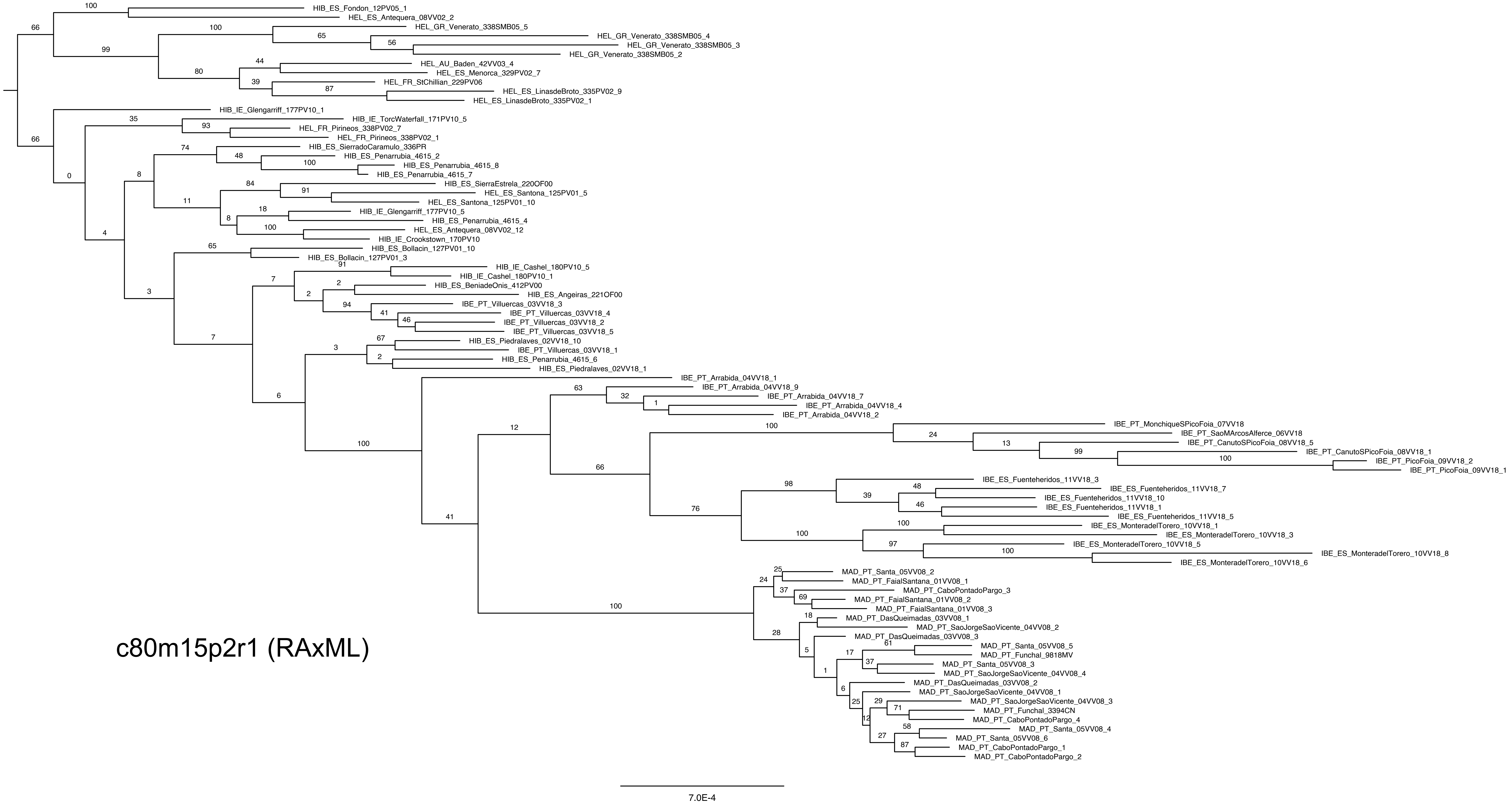
HIB_ES_Fondon_12PV05_1
HEL_ES_Antequera_08VV02_2
HEL_GR_Venerato_338SMB05_5
HEL_GR_Venerato_338SMB05_4
HEL_GR_Venerato_338SMB05_3
HEL_GR_Venerato_338SMB05_2
HEL_AU_Baden_42VV03_4
HEL_ES_Menorca_329PV02_7
HEL_FR_StChillian_229PV06
HEL_ES_LinasdeBroto_335PV02_9
HEL_ES_LinasdeBroto_335PV02_1
HIB_IE_Glengarriff_177PV10_1
HIB_IE_TorcWaterfall_171PV10_5
HEL_FR_Pirineos_338PV02_7
HEL_FR_Pirineos_338PV02_1
HIB_ES_SierradoCaramulo_336PR
HIB_ES_Penarrubia_4615_2
HIB_ES_Penarrubia_4615_8
HIB_ES_Penarrubia_4615_7
HIB_ES_SierraEstrela_220OF00
HEL_ES_Santona_125PV01_5
HEL_ES_Santona_125PV01_10
HIB_IE_Glengarriff_177PV10_5
HIB_ES_Penarrubia_4615_4
HEL_ES_Antequera_08VV02_12
HIB_IE_Crookstown_170PV10
HIB_ES_Bollacin_127PV01_10
HIB_ES_Bollacin_127PV01_3
HIB_IE_Cashel_180PV10_5
HIB_IE_Cashel_180PV10_1
HIB_ES_BeniadeOnis_412PV00
HIB_ES_Angeiras_221OF00
IBE_PT_Villuercas_03VV18_3
IBE_PT_Villuercas_03VV18_4
IBE_PT_Villuercas_03VV18_2
IBE_PT_Villuercas_03VV18_5
HIB_ES_Piedralaves_02VV18_10
IBE_PT_Villuercas_03VV18_1
HIB_ES_Penarrubia_4615_6
HIB_ES_Piedralaves_02VV18_1
IBE_PT_Arrabida_04VV18_1
IBE_PT_Arrabida_04VV18_9
IBE_PT_Arrabida_04VV18_7
IBE_PT_Arrabida_04VV18_4
IBE_PT_Arrabida_04VV18_2
IBE_PT_MonchiqueSPicoFoia_07VV18
IBE_PT_SaoMArcosAlferce_06VV18
IBE_PT_CanutoSPicoFoia_08VV18_5
IBE_PT_CanutoSPicoFoia_08VV18_1
IBE_PT_PicoFoia_09VV18_2
IBE_PT_PicoFoia_09VV18_1
IBE_ES_Fuenteheridos_11VV18_3
IBE_ES_Fuenteheridos_11VV18_7
IBE_ES_Fuenteheridos_11VV18_10
IBE_ES_Fuenteheridos_11VV18_1
IBE_ES_Fuenteheridos_11VV18_5
IBE_ES_MonteradelTorero_10VV18_1
IBE_ES_MonteradelTorero_10VV18_3
IBE_ES_MonteradelTorero_10VV18_5
IBE_ES_MonteradelTorero_10VV18_8
IBE_ES_MonteradelTorero_10VV18_6
MAD_PT_Santa_05VV08_2
MAD_PT_FaialSantana_01VV08_1
MAD_PT_CaboPontadoPargo_3
MAD_PT_FaialSantana_01VV08_2
MAD_PT_FaialSantana_01VV08_3
MAD_PT_DasQueimadas_03VV08_1
MAD_PT_SaoJorgeSaoVicente_04VV08_2
MAD_PT_DasQueimadas_03VV08_3
MAD_PT_Santa_05VV08_5
MAD_PT_Funchal_9818MV
MAD_PT_Santa_05VV08_3
MAD_PT_SaoJorgeSaoVicente_04VV08_4
MAD_PT_DasQueimadas_03VV08_2
MAD_PT_SaoJorgeSaoVicente_04VV08_1
MAD_PT_SaoJorgeSaoVicente_04VV08_3
MAD_PT_Funchal_3394CN
MAD_PT_CaboPontadoPargo_4
MAD_PT_Santa_05VV08_4
MAD_PT_Santa_05VV08_6
MAD_PT_CaboPontadoPargo_1
MAD_PT_CaboPontadoPargo_2
c80m15p2r1 (RAxML)
7.0E-4

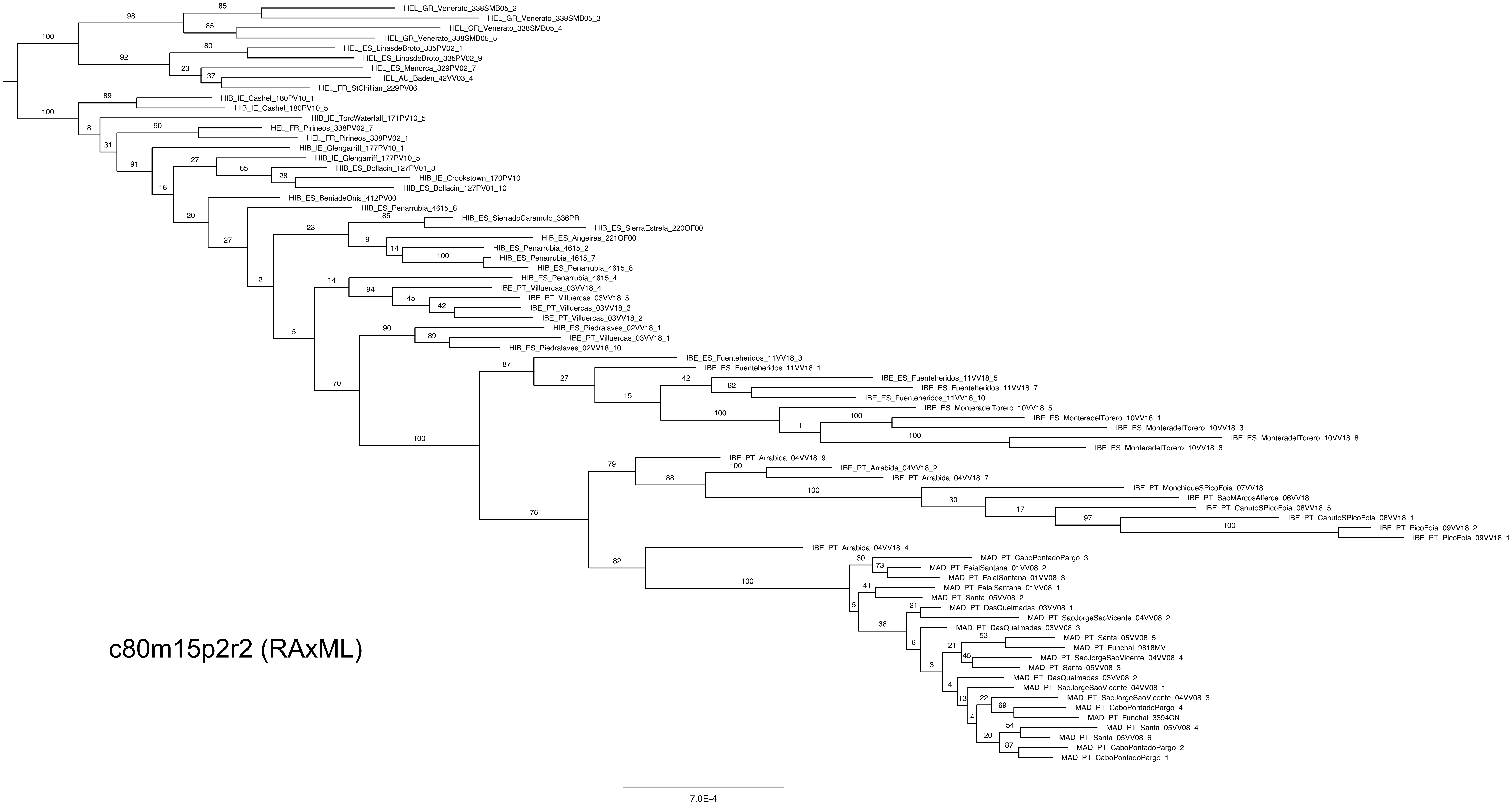
HEL_GR_Venerato_338SMB05_2
HEL_GR_Venerato_338SMB05_3
HEL_GR_Venerato_338SMB05_4
HEL_GR_Venerato_338SMB05_5
HEL_ES_LinasdeBroto_335PV02_1
HEL_ES_LinasdeBroto_335PV02_9
HEL_ES_Menorca_329PV02_7
HEL_AU_Baden_42VV03_4
HEL_FR_StChillian_229PV06
HIB_IE_Cashel_180PV10_1
HIB_IE_Cashel_180PV10_5
HIB_IE_TorcWaterfall_171PV10_5
HEL_FR_Pirineos_338PV02_7
HEL_FR_Pirineos_338PV02_1
HIB_IE_Glengarriff_177PV10_1
HIB_IE_Glengarriff_177PV10_5
HIB_ES_Bollacin_127PV01_3
HIB_IE_Crookstown_170PV10
HIB_ES_Bollacin_127PV01_10
HIB_ES_BeniadeOnis_412PV00
HIB_ES_Penarrubia_4615_6
HIB_ES_SierradoCaramulo_336PR
HIB_ES_SierraEstrela_220OF00
HIB_ES_Angeiras_221OF00
HIB_ES_Penarrubia_4615_2
HIB_ES_Penarrubia_4615_7
HIB_ES_Penarrubia_4615_8
HIB_ES_Penarrubia_4615_4
IBE_PT_Villuercas_03VV18_4
IBE_PT_Villuercas_03VV18_5
IBE_PT_Villuercas_03VV18_3
IBE_PT_Villuercas_03VV18_2
HIB_ES_Piedralaves_02VV18_1
IBE_PT_Villuercas_03VV18_1
HIB_ES_Piedralaves_02VV18_10
IBE_ES_Fuenteheridos_11VV18_3
IBE_ES_Fuenteheridos_11VV18_1
IBE_ES_Fuenteheridos_11VV18_5
IBE_ES_Fuenteheridos_11VV18_7
IBE_ES_Fuenteheridos_11VV18_10
IBE_ES_MonteradelTorero_10VV18_5
IBE_ES_MonteradelTorero_10VV18_1
IBE_ES_MonteradelTorero_10VV18_3
IBE_ES_MonteradelTorero_10VV18_8
IBE_ES_MonteradelTorero_10VV18_6
IBE_PT_Arrabida_04VV18_9
IBE_PT_Arrabida_04VV18_2
IBE_PT_Arrabida_04VV18_7
IBE_PT_MonchiqueSPicoFoia_07VV18
IBE_PT_SaoMArcosAlferce_06VV18
IBE_PT_CanutoSPicoFoia_08VV18_5
IBE_PT_CanutoSPicoFoia_08VV18_1
IBE_PT_PicoFoia_09VV18_2
IBE_PT_PicoFoia_09VV18_1
IBE_PT_Arrabida_04VV18_4
MAD_PT_CaboPontadoPargo_3
MAD_PT_FaialSantana_01VV08_2
MAD_PT_FaialSantana_01VV08_3
MAD_PT_FaialSantana_01VV08_1
MAD_PT_Santa_05VV08_2
MAD_PT_DasQueimadas_03VV08_1
MAD_PT_SaoJorgeSaoVicente_04VV08_2
MAD_PT_DasQueimadas_03VV08_3
MAD_PT_Santa_05VV08_5
MAD_PT_Funchal_9818MV
MAD_PT_SaoJorgeSaoVicente_04VV08_4
MAD_PT_Santa_05VV08_3
MAD_PT_DasQueimadas_03VV08_2
MAD_PT_SaoJorgeSaoVicente_04VV08_1
MAD_PT_SaoJorgeSaoVicente_04VV08_3
MAD_PT_CaboPontadoPargo_4
MAD_PT_Funchal_3394CN
MAD_PT_Santa_05VV08_4
MAD_PT_Santa_05VV08_6
MAD_PT_CaboPontadoPargo_2
MAD_PT_CaboPontadoPargo_1
c80m15p2r2 (RAxML)
7.0E-4

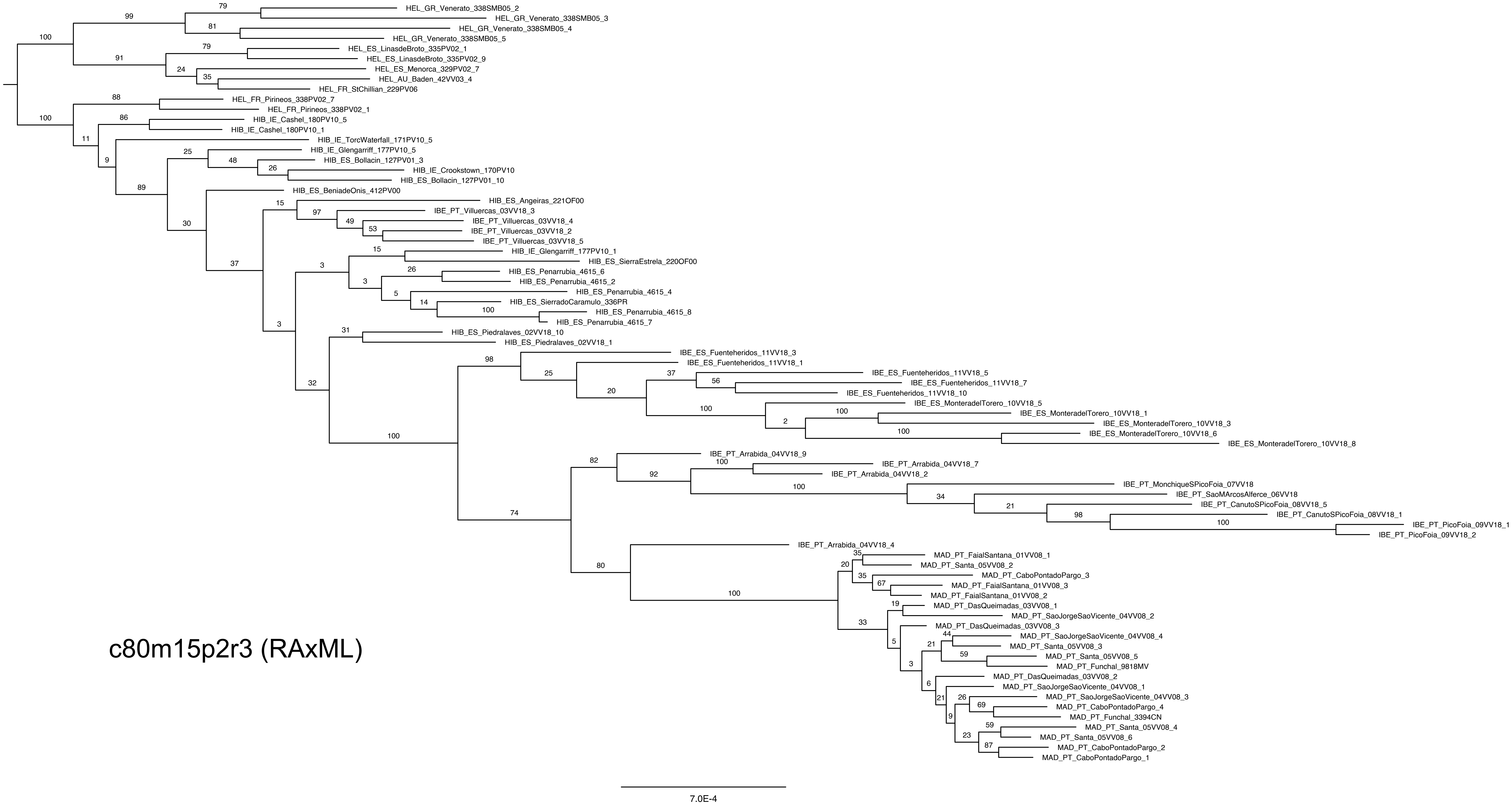
HEL_GR_Venerato_338SMB05_2
HEL_GR_Venerato_338SMB05_3
HEL_GR_Venerato_338SMB05_4
HEL_GR_Venerato_338SMB05_5
HEL_ES_LinasdeBroto_335PV02_1
HEL_ES_LinasdeBroto_335PV02_9
HEL_ES_Menorca_329PV02_7
HEL_AU_Baden_42VV03_4
HEL_FR_StChillian_229PV06
HEL_FR_Pirineos_338PV02_7
HEL_FR_Pirineos_338PV02_1
HIB_IE_Cashel_180PV10_5
HIB_IE_Cashel_180PV10_1
HIB_IE_TorcWaterfall_171PV10_5
HIB_IE_Glengarriff_177PV10_5
HIB_ES_Bollacin_127PV01_3
HIB_IE_Crookstown_170PV10
HIB_ES_Bollacin_127PV01_10
HIB_ES_BeniadeOnis_412PV00
HIB_ES_Angeiras_221OF00
IBE_PT_Villuercas_03VV18_3
IBE_PT_Villuercas_03VV18_4
IBE_PT_Villuercas_03VV18_2
IBE_PT_Villuercas_03VV18_5
HIB_IE_Glengarriff_177PV10_1
HIB_ES_SierraEstrela_220OF00
HIB_ES_Penarrubia_4615_6
HIB_ES_Penarrubia_4615_2
HIB_ES_Penarrubia_4615_4
HIB_ES_SierradoCaramulo_336PR
HIB_ES_Penarrubia_4615_8
HIB_ES_Penarrubia_4615_7
HIB_ES_Piedralaves_02VV18_10
HIB_ES_Piedralaves_02VV18_1
IBE_ES_Fuenteheridos_11VV18_3
IBE_ES_Fuenteheridos_11VV18_1
IBE_ES_Fuenteheridos_11VV18_5
IBE_ES_Fuenteheridos_11VV18_7
IBE_ES_Fuenteheridos_11VV18_10
IBE_ES_MonteradelTorero_10VV18_5
IBE_ES_MonteradelTorero_10VV18_1
IBE_ES_MonteradelTorero_10VV18_3
IBE_ES_MonteradelTorero_10VV18_6
IBE_ES_MonteradelTorero_10VV18_8
IBE_PT_Arrabida_04VV18_9
IBE_PT_Arrabida_04VV18_7
IBE_PT_Arrabida_04VV18_2
IBE_PT_MonchiqueSPicoFoia_07VV18
IBE_PT_SaoMArcosAlferce_06VV18
IBE_PT_CanutoSPicoFoia_08VV18_5
IBE_PT_CanutoSPicoFoia_08VV18_1
IBE_PT_PicoFoia_09VV18_1
IBE_PT_PicoFoia_09VV18_2
IBE_PT_Arrabida_04VV18_4
MAD_PT_FaialSantana_01VV08_1
MAD_PT_Santa_05VV08_2
MAD_PT_CaboPontadoPargo_3
MAD_PT_FaialSantana_01VV08_3
MAD_PT_FaialSantana_01VV08_2
MAD_PT_DasQueimadas_03VV08_1
MAD_PT_SaoJorgeSaoVicente_04VV08_2
MAD_PT_DasQueimadas_03VV08_3
MAD_PT_SaoJorgeSaoVicente_04VV08_4
MAD_PT_Santa_05VV08_3
MAD_PT_Santa_05VV08_5
MAD_PT_Funchal_9818MV
MAD_PT_DasQueimadas_03VV08_2
MAD_PT_SaoJorgeSaoVicente_04VV08_1
MAD_PT_SaoJorgeSaoVicente_04VV08_3
MAD_PT_CaboPontadoPargo_4
MAD_PT_Funchal_3394CN
MAD_PT_Santa_05VV08_4
MAD_PT_Santa_05VV08_6
MAD_PT_CaboPontadoPargo_2
MAD_PT_CaboPontadoPargo_1
c80m15p2r3 (RAxML)
7.0E-4

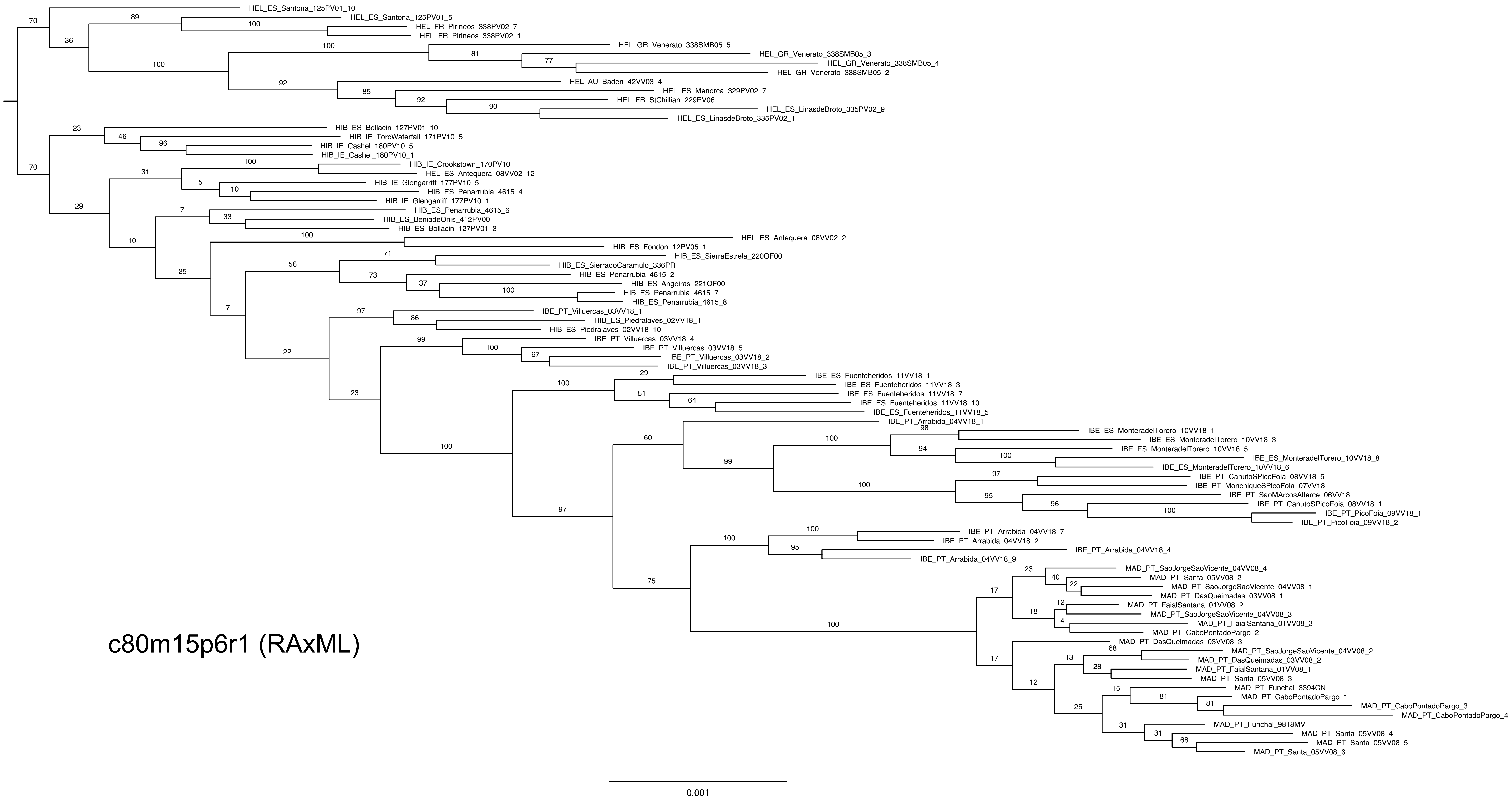

HEL_ES_Santona_125PV01_10
HEL_ES_Santona_125PV01_5
HEL_FR_Pirineos_338PV02_7
HEL_FR_Pirineos_338PV02_1
HEL_GR_Venerato_338SMB05_5
HEL_GR_Venerato_338SMB05_3
HEL_GR_Venerato_338SMB05_4
HEL_GR_Venerato_338SMB05_2
HEL_AU_Baden_42VV03_4
HEL_ES_Menorca_329PV02_7
HEL_FR_StChillian_229PV06
HEL_ES_LinasdeBroto_335PV02_9
HEL_ES_LinasdeBroto_335PV02_1
HIB_ES_Bollacin_127PV01_10
HIB_IE_TorcWaterfall_171PV10_5
HIB_IE_Cashel_180PV10_5
HIB_IE_Cashel_180PV10_1
HIB_IE_Crookstown_170PV10
HEL_ES_Antequera_08VV02_12
HIB_IE_Glengarriff_177PV10_5
HIB_ES_Penarrubia_4615_4
HIB_IE_Glengarriff_177PV10_1
HIB_ES_Penarrubia_4615_6
HIB_ES_BeniadeOnis_412PV00
HIB_ES_Bollacin_127PV01_3
HEL_ES_Antequera_08VV02_2
HIB_ES_Fondon_12PV05_1
HIB_ES_SierraEstrela_220OF00
HIB_ES_SierradoCaramulo_336PR
HIB_ES_Penarrubia_4615_2
HIB_ES_Angeiras_221OF00
HIB_ES_Penarrubia_4615_7
HIB_ES_Penarrubia_4615_8
IBE_PT_Villuercas_03VV18_1
HIB_ES_Piedralaves_02VV18_1
HIB_ES_Piedralaves_02VV18_10
IBE_PT_Villuercas_03VV18_4
IBE_PT_Villuercas_03VV18_5
IBE_PT_Villuercas_03VV18_2
IBE_PT_Villuercas_03VV18_3
IBE_ES_Fuenteheridos_11VV18_1
IBE_ES_Fuenteheridos_11VV18_3
IBE_ES_Fuenteheridos_11VV18_7
IBE_ES_Fuenteheridos_11VV18_10
IBE_ES_Fuenteheridos_11VV18_5
IBE_PT_Arrabida_04VV18_1
IBE_ES_MonteradelTorero_10VV18_1
IBE_ES_MonteradelTorero_10VV18_3
IBE_ES_MonteradelTorero_10VV18_5
IBE_ES_MonteradelTorero_10VV18_8
IBE_ES_MonteradelTorero_10VV18_6
IBE_PT_CanutoSPicoFoia_08VV18_5
IBE_PT_MonchiqueSPicoFoia_07VV18
IBE_PT_SaoMArcosAlferce_06VV18
IBE_PT_CanutoSPicoFoia_08VV18_1
IBE_PT_PicoFoia_09VV18_1
IBE_PT_PicoFoia_09VV18_2
IBE_PT_Arrabida_04VV18_7
IBE_PT_Arrabida_04VV18_2
IBE_PT_Arrabida_04VV18_4
IBE_PT_Arrabida_04VV18_9
MAD_PT_SaoJorgeSaoVicente_04VV08_4
MAD_PT_Santa_05VV08_2
MAD_PT_SaoJorgeSaoVicente_04VV08_1
MAD_PT_DasQueimadas_03VV08_1
MAD_PT_FaialSantana_01VV08_2
MAD_PT_SaoJorgeSaoVicente_04VV08_3
MAD_PT_FaialSantana_01VV08_3
MAD_PT_CaboPontadoPargo_2
MAD_PT_DasQueimadas_03VV08_3
MAD_PT_SaoJorgeSaoVicente_04VV08_2
MAD_PT_DasQueimadas_03VV08_2
MAD_PT_FaialSantana_01VV08_1
MAD_PT_Santa_05VV08_3
MAD_PT_Funchal_3394CN
MAD_PT_CaboPontadoPargo_1
MAD_PT_CaboPontadoPargo_3
MAD_PT_CaboPontadoPargo_4
MAD_PT_Funchal_9818MV
MAD_PT_Santa_05VV08_4
MAD_PT_Santa_05VV08_5
MAD_PT_Santa_05VV08_6
c80m15p6r1 (RAxML)
0.001

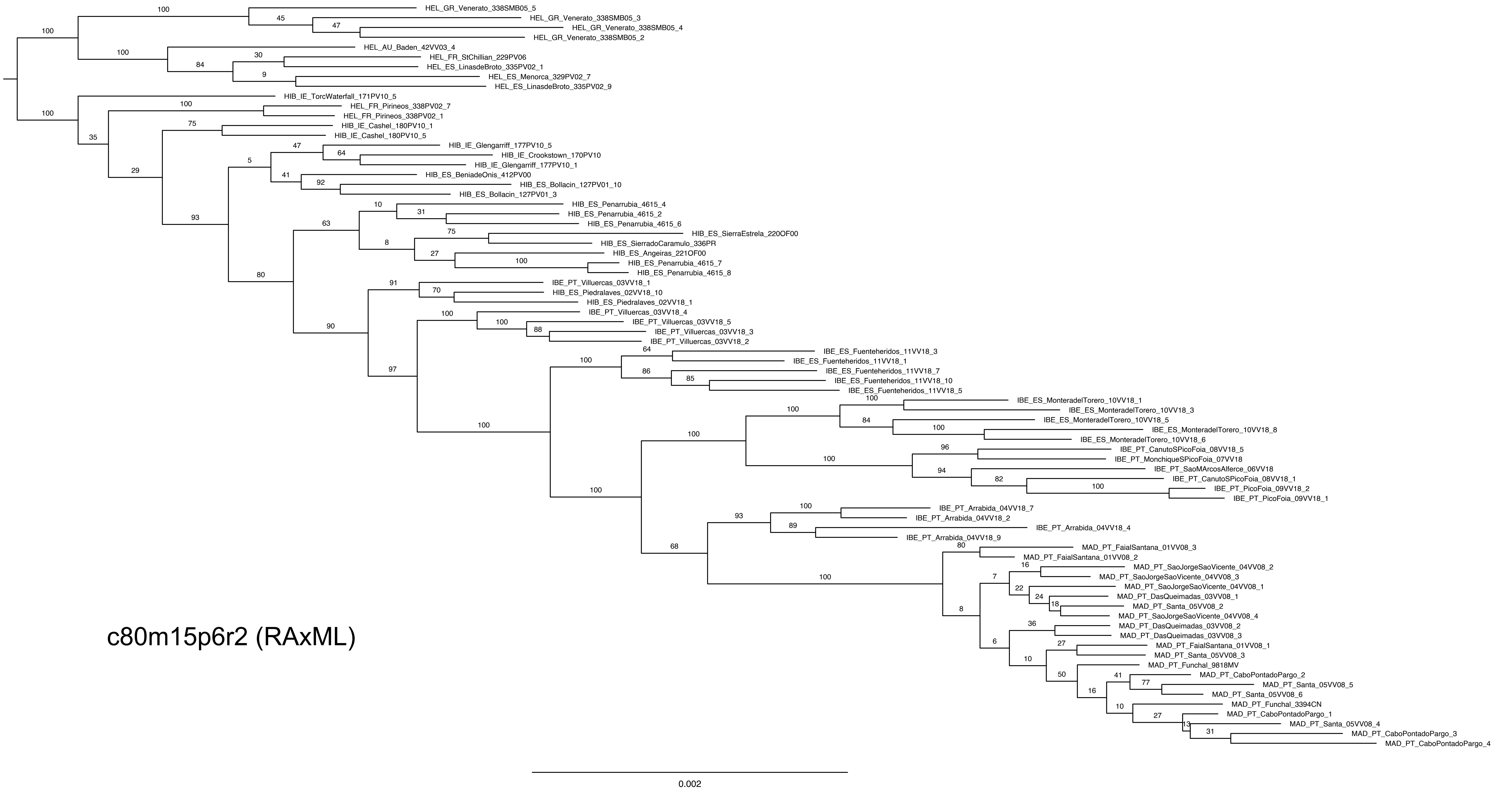
HEL_GR_Venerato_338SMB05_5
HEL_GR_Venerato_338SMB05_3
HEL_GR_Venerato_338SMB05_4
HEL_GR_Venerato_338SMB05_2
HEL_AU_Baden_42VV03_4
HEL_FR_StChillian_229PV06
HEL_ES_LinasdeBroto_335PV02_1
HEL_ES_Menorca_329PV02_7
HEL_ES_LinasdeBroto_335PV02_9
HIB_IE_TorcWaterfall_171PV10_5
HIB_FR_Pirineos_338PV02_7
HIB_FR_Pirineos_338PV02_1
HIB_IE_Cashel_180PV10_1
HIB_IE_Cashel_180PV10_5
HIB_IE_Glengarriff_177PV10_5
HIB_IE_Crookstown_170PV10
HIB_IE_Glengarriff_177PV10_1
HIB_ES_BeniadeOnis_412PV00
HIB_ES_Bollacin_127PV01_10
HIB_ES_Bollacin_127PV01_3
HIB_ES_Penarrubia_4615_4
HIB_ES_Penarrubia_4615_2
HIB_ES_Penarrubia_4615_6
HIB_ES_SierraEstrela_220OF00
HIB_ES_SierradoCaramulo_336PR
HIB_ES_Angeiras_221OF00
HIB_ES_Penarrubia_4615_7
HIB_ES_Penarrubia_4615_8
IBE_PT_Villuercas_03VV18_1
HIB_ES_Piedralaves_02VV18_10
HIB_ES_Piedralaves_02VV18_1
IBE_PT_Villuercas_03VV18_4
IBE_PT_Villuercas_03VV18_5
IBE_PT_Villuercas_03VV18_3
IBE_PT_Villuercas_03VV18_2
IBE_ES_Fuenteheridos_11VV18_3
IBE_ES_Fuenteheridos_11VV18_1
IBE_ES_Fuenteheridos_11VV18_7
IBE_ES_Fuenteheridos_11VV18_10
IBE_ES_Fuenteheridos_11VV18_5
IBE_ES_MonteradelTorero_10VV18_1
IBE_ES_MonteradelTorero_10VV18_3
IBE_ES_MonteradelTorero_10VV18_5
IBE_ES_MonteradelTorero_10VV18_8
IBE_ES_MonteradelTorero_10VV18_6
IBE_PT_CanutoSPicoFoia_08VV18_5
IBE_PT_MonchiqueSPicoFoia_07VV18
IBE_PT_SaoMArcosAlferce_06VV18
IBE_PT_CanutoSPicoFoia_08VV18_1
IBE_PT_PicoFoia_09VV18_2
IBE_PT_PicoFoia_09VV18_1
IBE_PT_Arrabida_04VV18_7
IBE_PT_Arrabida_04VV18_2
IBE_PT_Arrabida_04VV18_4
IBE_PT_Arrabida_04VV18_9
MAD_PT_FaialSantana_01VV08_3
MAD_PT_FaialSantana_01VV08_2
MAD_PT_SaoJorgeSaoVicente_04VV08_2
MAD_PT_SaoJorgeSaoVicente_04VV08_3
MAD_PT_SaoJorgeSaoVicente_04VV08_1
MAD_PT_DasQueimadas_03VV08_1
MAD_PT_Santa_05VV08_2
MAD_PT_SaoJorgeSaoVicente_04VV08_4
MAD_PT_DasQueimadas_03VV08_2
MAD_PT_DasQueimadas_03VV08_3
MAD_PT_FaialSantana_01VV08_1
MAD_PT_Santa_05VV08_3
MAD_PT_Funchal_9818MV
MAD_PT_CaboPontadoPargo_2
MAD_PT_Santa_05VV08_5
MAD_PT_Santa_05VV08_6
MAD_PT_Funchal_3394CN
MAD_PT_CaboPontadoPargo_1
MAD_PT_Santa_05VV08_4
MAD_PT_CaboPontadoPargo_3
MAD_PT_CaboPontadoPargo_4
c80m15p6r2 (RAxML)
0.002

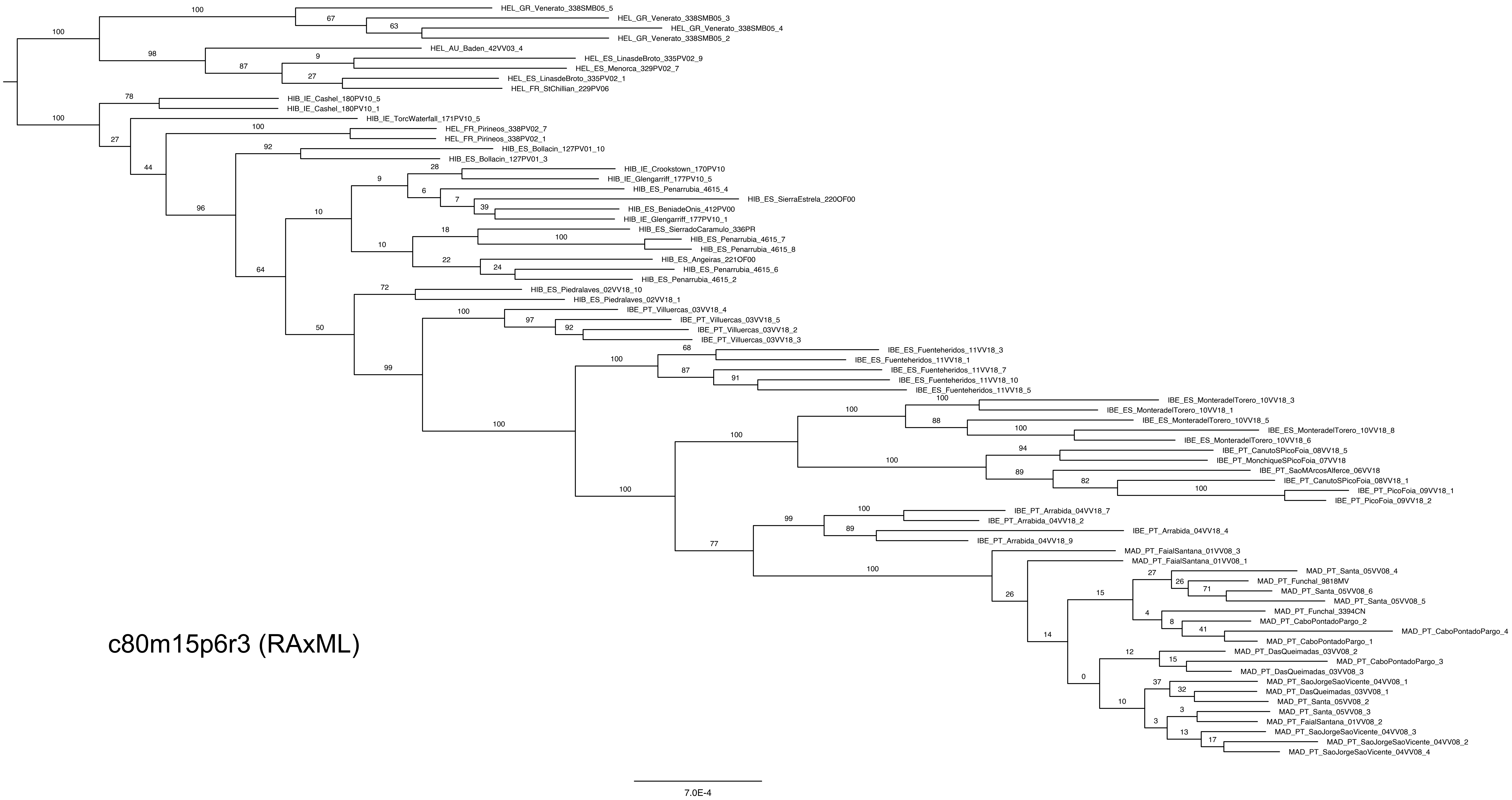
HEL_GR_Venerato_338SMB05_5
HEL_GR_Venerato_338SMB05_3
HEL_GR_Venerato_338SMB05_4
HEL_GR_Venerato_338SMB05_2
HEL_AU_Baden_42VV03_4
HEL_ES_LinasdeBroto_335PV02_9
HEL_ES_Menorca_329PV02_7
HEL_ES_LinasdeBroto_335PV02_1
HEL_FR_StChillian_229PV06
HIB_IE_Cashel_180PV10_5
HIB_IE_Cashel_180PV10_1
HIB_IE_TorcWaterfall_171PV10_5
HEL_FR_Pirineos_338PV02_7
HEL_FR_Pirineos_338PV02_1
HIB_ES_Bollacin_127PV01_10
HIB_ES_Bollacin_127PV01_3
HIB_IE_Crookstown_170PV10
HIB_IE_Glengarriff_177PV10_5
HIB_ES_Penarrubia_4615_4
HIB_ES_SierraEstrela_220OF00
HIB_ES_BeniadeOnis_412PV00
HIB_IE_Glengarriff_177PV10_1
HIB_ES_SierradoCaramulo_336PR
HIB_ES_Penarrubia_4615_7
HIB_ES_Penarrubia_4615_8
HIB_ES_Angeiras_221OF00
HIB_ES_Penarrubia_4615_6
HIB_ES_Penarrubia_4615_2
HIB_ES_Piedralaves_02VV18_10
HIB_ES_Piedralaves_02VV18_1
IBE_PT_Villuercas_03VV18_4
IBE_PT_Villuercas_03VV18_5
IBE_PT_Villuercas_03VV18_2
IBE_PT_Villuercas_03VV18_3
IBE_ES_Fuenteheridos_11VV18_3
IBE_ES_Fuenteheridos_11VV18_1
IBE_ES_Fuenteheridos_11VV18_7
IBE_ES_Fuenteheridos_11VV18_10
IBE_ES_Fuenteheridos_11VV18_5
IBE_ES_MonteradelTorero_10VV18_3
IBE_ES_MonteradelTorero_10VV18_1
IBE_ES_MonteradelTorero_10VV18_5
IBE_ES_MonteradelTorero_10VV18_8
IBE_ES_MonteradelTorero_10VV18_6
IBE_PT_CanutoSPicoFoia_08VV18_5
IBE_PT_MonchiqueSPicoFoia_07VV18
IBE_PT_SaoMArcosAlferce_06VV18
IBE_PT_CanutoSPicoFoia_08VV18_1
IBE_PT_PicoFoia_09VV18_1
IBE_PT_PicoFoia_09VV18_2
IBE_PT_Arrabida_04VV18_7
IBE_PT_Arrabida_04VV18_2
IBE_PT_Arrabida_04VV18_4
IBE_PT_Arrabida_04VV18_9
MAD_PT_FaialSantana_01VV08_3
MAD_PT_FaialSantana_01VV08_1
MAD_PT_Santa_05VV08_4
MAD_PT_Funchal_9818MV
MAD_PT_Santa_05VV08_6
MAD_PT_Santa_05VV08_5
MAD_PT_Funchal_3394CN
MAD_PT_CaboPontadoPargo_2
MAD_PT_CaboPontadoPargo_4
MAD_PT_CaboPontadoPargo_1
MAD_PT_DasQueimadas_03VV08_2
MAD_PT_CaboPontadoPargo_3
MAD_PT_DasQueimadas_03VV08_3
MAD_PT_SaoJorgeSaoVicente_04VV08_1
MAD_PT_DasQueimadas_03VV08_1
MAD_PT_Santa_05VV08_2
MAD_PT_Santa_05VV08_3
MAD_PT_FaialSantana_01VV08_2
MAD_PT_SaoJorgeSaoVicente_04VV08_3
MAD_PT_SaoJorgeSaoVicente_04VV08_2
MAD_PT_SaoJorgeSaoVicente_04VV08_4
c80m15p6r3 (RAxML)
7.0E-4

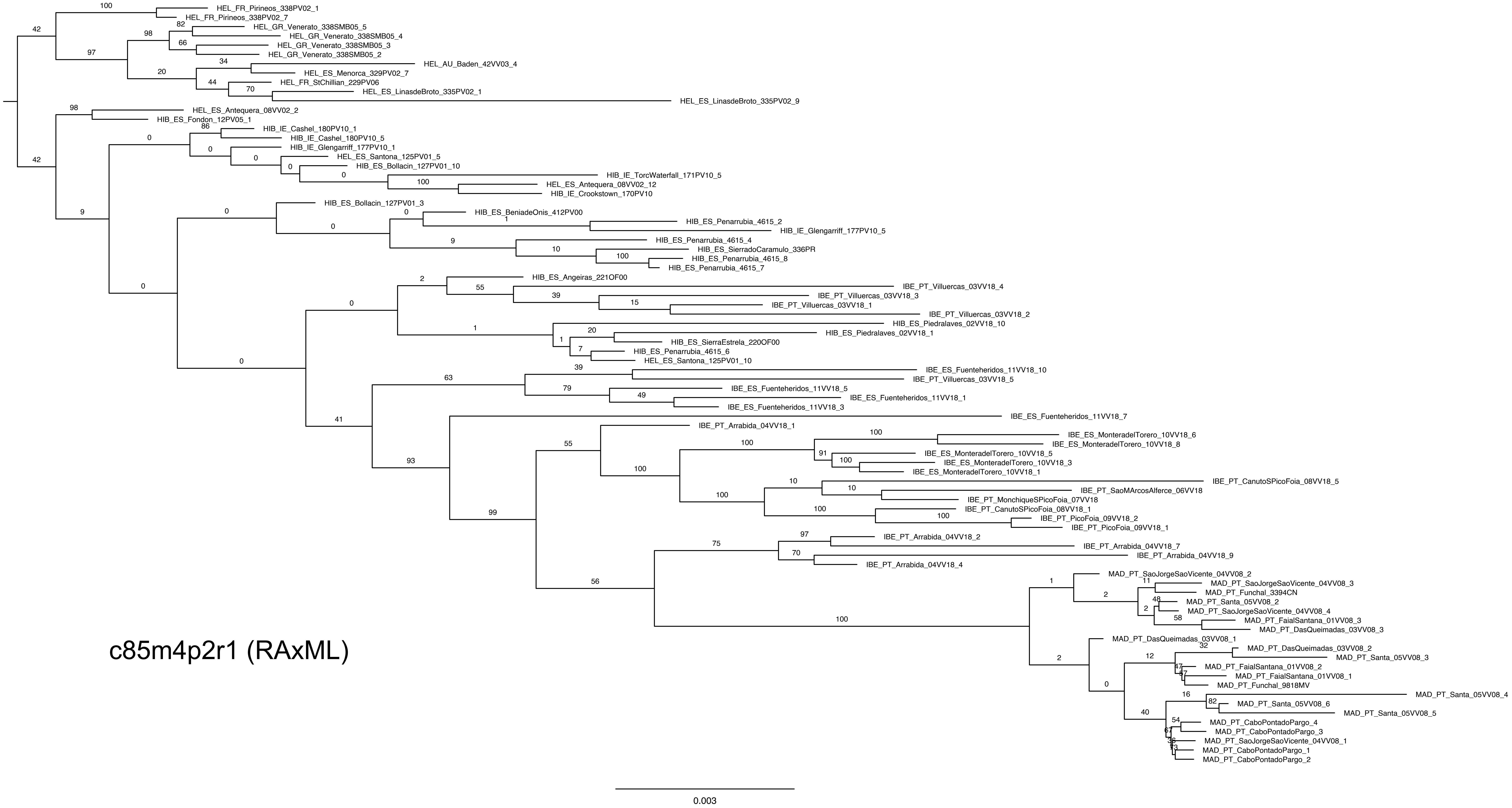
HEL_FR_Pirineos_338PV02_1
HEL_FR_Pirineos_338PV02_7
HEL_GR_Venerato_338SMB05_5
HEL_GR_Venerato_338SMB05_4
HEL_GR_Venerato_338SMB05_3
HEL_GR_Venerato_338SMB05_2
HEL_AU_Baden_42VV03_4
HEL_ES_Menorca_329PV02_7
HEL_FR_StChillian_229PV06
HEL_ES_LinasdeBroto_335PV02_1
HEL_ES_LinasdeBroto_335PV02_9
HEL_ES_Antequera_08VV02_2
HIB_ES_Fondon_12PV05_1
HIB_IE_Cashel_180PV10_1
HIB_IE_Cashel_180PV10_5
HIB_IE_Glengarriff_177PV10_1
HEL_ES_Santona_125PV01_5
HIB_ES_Bollacin_127PV01_10
HIB_IE_TorcWaterfall_171PV10_5
HEL_ES_Antequera_08VV02_12
HIB_IE_Crookstown_170PV10
HIB_ES_Bollacin_127PV01_3
HIB_ES_BeniadeOnis_412PV00
HIB_ES_Penarrubia_4615_2
HIB_IE_Glengarriff_177PV10_5
HIB_ES_Penarrubia_4615_4
HIB_ES_SierradoCaramulo_336PR
HIB_ES_Penarrubia_4615_8
HIB_ES_Penarrubia_4615_7
HIB_ES_Angeiras_221OF00
IBE_PT_Villuercas_03VV18_4
IBE_PT_Villuercas_03VV18_3
IBE_PT_Villuercas_03VV18_1
IBE_PT_Villuercas_03VV18_2
HIB_ES_Piedralaves_02VV18_10
HIB_ES_Piedralaves_02VV18_1
HIB_ES_SierraEstrela_220OF00
HIB_ES_Penarrubia_4615_6
HEL_ES_Santona_125PV01_10
IBE_ES_Fuenteheridos_11VV18_10
IBE_PT_Villuercas_03VV18_5
IBE_ES_Fuenteheridos_11VV18_5
IBE_ES_Fuenteheridos_11VV18_1
IBE_ES_Fuenteheridos_11VV18_3
IBE_ES_Fuenteheridos_11VV18_7
IBE_PT_Arrabida_04VV18_1
IBE_ES_MonteradelTorero_10VV18_6
IBE_ES_MonteradelTorero_10VV18_8
IBE_ES_MonteradelTorero_10VV18_5
IBE_ES_MonteradelTorero_10VV18_3
IBE_ES_MonteradelTorero_10VV18_1
IBE_PT_CanutoSPicoFoia_08VV18_5
IBE_PT_SaoMArcosAlferce_06VV18
IBE_PT_MonchiqueSPicoFoia_07VV18
IBE_PT_CanutoSPicoFoia_08VV18_1
IBE_PT_PicoFoia_09VV18_2
IBE_PT_PicoFoia_09VV18_1
IBE_PT_Arrabida_04VV18_2
IBE_PT_Arrabida_04VV18_7
IBE_PT_Arrabida_04VV18_9
IBE_PT_Arrabida_04VV18_4
MAD_PT_SaoJorgeSaoVicente_04VV08_2
MAD_PT_SaoJorgeSaoVicente_04VV08_3
MAD_PT_Funchal_3394CN
MAD_PT_Santa_05VV08_2
MAD_PT_SaoJorgeSaoVicente_04VV08_4
MAD_PT_FaialSantana_01VV08_3
MAD_PT_DasQueimadas_03VV08_3
MAD_PT_DasQueimadas_03VV08_1
MAD_PT_DasQueimadas_03VV08_2
MAD_PT_Santa_05VV08_3
MAD_PT_FaialSantana_01VV08_2
MAD_PT_FaialSantana_01VV08_1
MAD_PT_Funchal_9818MV
MAD_PT_Santa_05VV08_4
MAD_PT_Santa_05VV08_6
MAD_PT_Santa_05VV08_5
MAD_PT_CaboPontadoPargo_4
MAD_PT_CaboPontadoPargo_3
MAD_PT_SaoJorgeSaoVicente_04VV08_1
MAD_PT_CaboPontadoPargo_1
MAD_PT_CaboPontadoPargo_2
c85m4p2r1 (RAxML)
0.003

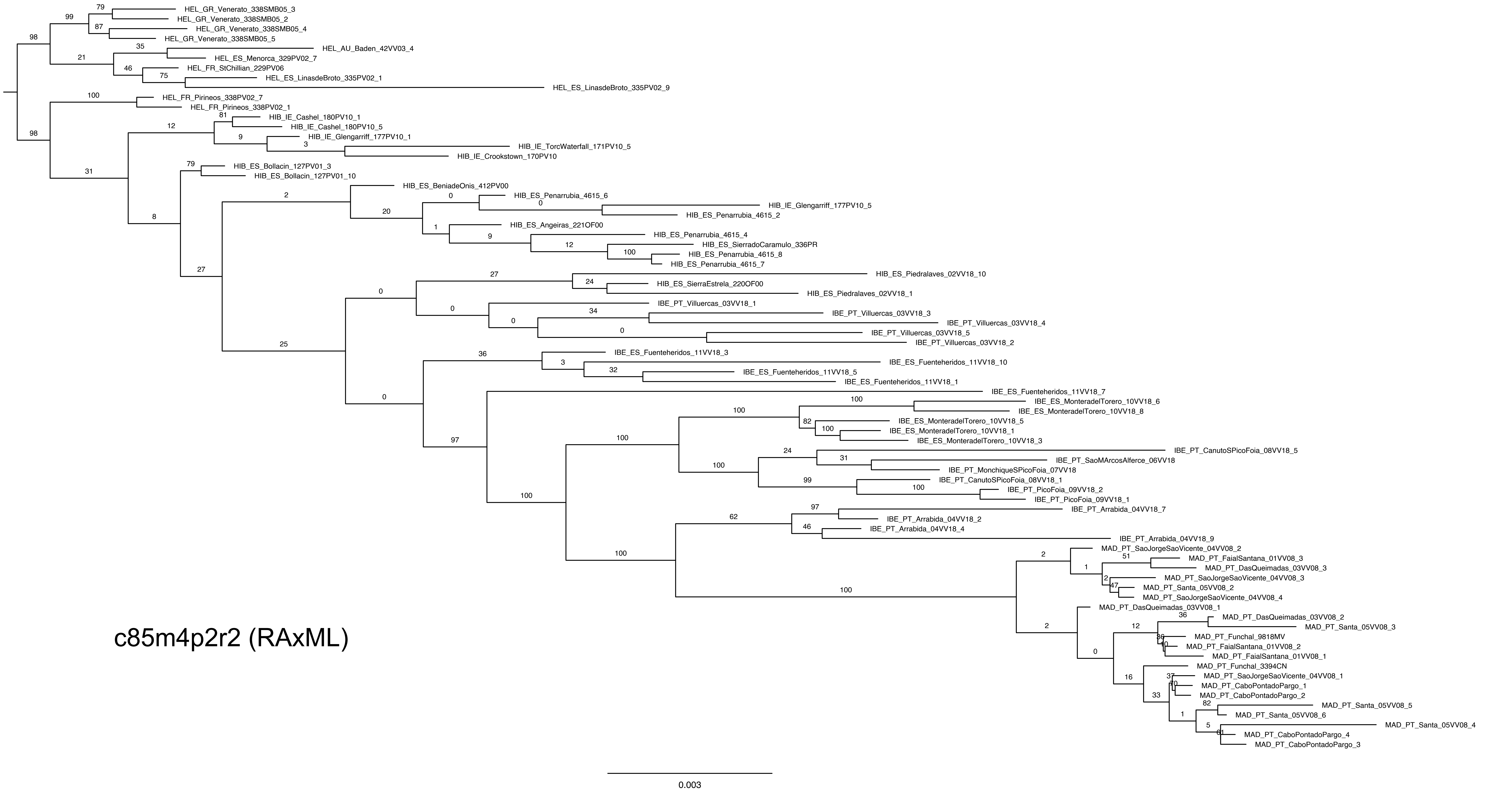
c85m4p2r2 (RAxML)
HEL_GR_Venerato_338SMB05_3
HEL_GR_Venerato_338SMB05_2
HEL_GR_Venerato_338SMB05_4
HEL_GR_Venerato_338SMB05_5
HEL_AU_Baden_42VV03_4
HEL_ES_Menorca_329PV02_7
HEL_FR_StChillian_229PV06
HEL_ES_LinasdeBroto_335PV02_1
HEL_ES_LinasdeBroto_335PV02_9
HEL_FR_Pirineos_338PV02_7
HEL_FR_Pirineos_338PV02_1
HIB_IE_Cashel_180PV10_1
HIB_IE_Cashel_180PV10_5
HIB_IE_Glengarriff_177PV10_1
HIB_IE_TorcWaterfall_171PV10_5
HIB_IE_Crookstown_170PV10
HIB_ES_Bollacin_127PV01_3
HIB_ES_Bollacin_127PV01_10
HIB_ES_BeniadeOnis_412PV00
HIB_ES_Penarrubia_4615_6
HIB_IE_Glengarriff_177PV10_5
HIB_ES_Penarrubia_4615_2
HIB_ES_Angeiras_221OF00
HIB_ES_Penarrubia_4615_4
HIB_ES_SierradoCaramulo_336PR
HIB_ES_Penarrubia_4615_8
HIB_ES_Penarrubia_4615_7
HIB_ES_Piedralaves_02VV18_10
HIB_ES_SierraEstrela_220OF00
HIB_ES_Piedralaves_02VV18_1
IBE_PT_Villuercas_03VV18_1
IBE_PT_Villuercas_03VV18_3
IBE_PT_Villuercas_03VV18_4
IBE_PT_Villuercas_03VV18_5
IBE_PT_Villuercas_03VV18_2
IBE_ES_Fuenteheridos_11VV18_3
IBE_ES_Fuenteheridos_11VV18_10
IBE_ES_Fuenteheridos_11VV18_5
IBE_ES_Fuenteheridos_11VV18_1
IBE_ES_Fuenteheridos_11VV18_7
IBE_ES_MonteradelTorero_10VV18_6
IBE_ES_MonteradelTorero_10VV18_8
IBE_ES_MonteradelTorero_10VV18_5
IBE_ES_MonteradelTorero_10VV18_1
IBE_ES_MonteradelTorero_10VV18_3
IBE_PT_CanutoSPicoFoia_08VV18_5
IBE_PT_SaoMArcosAlferce_06VV18
IBE_PT_MonchiqueSPicoFoia_07VV18
IBE_PT_CanutoSPicoFoia_08VV18_1
IBE_PT_PicoFoia_09VV18_2
IBE_PT_PicoFoia_09VV18_1
IBE_PT_Arrabida_04VV18_7
IBE_PT_Arrabida_04VV18_2
IBE_PT_Arrabida_04VV18_4
IBE_PT_Arrabida_04VV18_9
MAD_PT_SaoJorgeSaoVicente_04VV08_2
MAD_PT_FaialSantana_01VV08_3
MAD_PT_DasQueimadas_03VV08_3
MAD_PT_SaoJorgeSaoVicente_04VV08_3
MAD_PT_Santa_05VV08_2
MAD_PT_SaoJorgeSaoVicente_04VV08_4
MAD_PT_DasQueimadas_03VV08_1
MAD_PT_DasQueimadas_03VV08_2
MAD_PT_Santa_05VV08_3
MAD_PT_Funchal_9818MV
MAD_PT_FaialSantana_01VV08_2
MAD_PT_FaialSantana_01VV08_1
MAD_PT_Funchal_3394CN
MAD_PT_SaoJorgeSaoVicente_04VV08_1
MAD_PT_CaboPontadoPargo_1
MAD_PT_CaboPontadoPargo_2
MAD_PT_Santa_05VV08_5
MAD_PT_Santa_05VV08_6
MAD_PT_Santa_05VV08_4
MAD_PT_CaboPontadoPargo_4
MAD_PT_CaboPontadoPargo_3
0.003

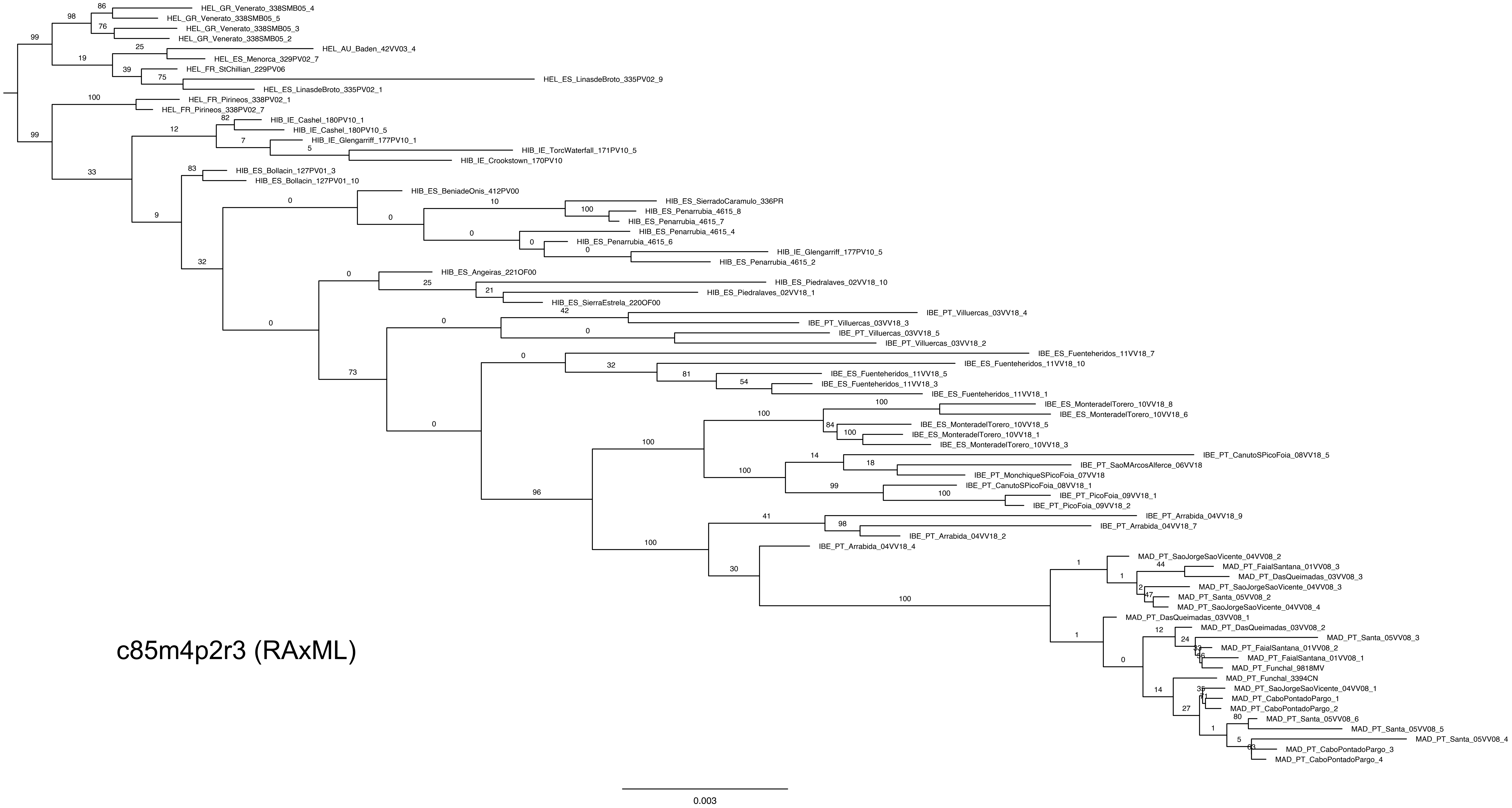
HEL_GR_Venerato_338SMB05_4
HEL_GR_Venerato_338SMB05_5
HEL_GR_Venerato_338SMB05_3
HEL_GR_Venerato_338SMB05_2
HEL_AU_Baden_42VV03_4
HEL_ES_Menorca_329PV02_7
HEL_FR_StChillian_229PV06
HEL_ES_LinasdeBroto_335PV02_9
HEL_ES_LinasdeBroto_335PV02_1
HEL_FR_Pirineos_338PV02_1
HEL_FR_Pirineos_338PV02_7
HIB_IE_Cashel_180PV10_1
HIB_IE_Cashel_180PV10_5
HIB_IE_Glengarriff_177PV10_1
HIB_IE_TorcWaterfall_171PV10_5
HIB_IE_Crookstown_170PV10
HIB_ES_Bollacin_127PV01_3
HIB_ES_Bollacin_127PV01_10
HIB_ES_BeniadeOnis_412PV00
HIB_ES_SierradoCaramulo_336PR
HIB_ES_Penarrubia_4615_8
HIB_ES_Penarrubia_4615_7
HIB_ES_Penarrubia_4615_4
HIB_ES_Penarrubia_4615_6
HIB_IE_Glengarriff_177PV10_5
HIB_ES_Penarrubia_4615_2
HIB_ES_Angeiras_221OF00
HIB_ES_Piedralaves_02VV18_10
HIB_ES_Piedralaves_02VV18_1
HIB_ES_SierraEstrela_220OF00
IBE_PT_Villuercas_03VV18_4
IBE_PT_Villuercas_03VV18_3
IBE_PT_Villuercas_03VV18_5
IBE_PT_Villuercas_03VV18_2
IBE_ES_Fuenteheridos_11VV18_7
IBE_ES_Fuenteheridos_11VV18_10
IBE_ES_Fuenteheridos_11VV18_5
IBE_ES_Fuenteheridos_11VV18_3
IBE_ES_Fuenteheridos_11VV18_1
IBE_ES_MonteradelTorero_10VV18_8
IBE_ES_MonteradelTorero_10VV18_6
IBE_ES_MonteradelTorero_10VV18_5
IBE_ES_MonteradelTorero_10VV18_1
IBE_ES_MonteradelTorero_10VV18_3
IBE_PT_CanutoSPicoFoia_08VV18_5
IBE_PT_SaoMArcosAlferce_06VV18
IBE_PT_MonchiqueSPicoFoia_07VV18
IBE_PT_CanutoSPicoFoia_08VV18_1
IBE_PT_PicoFoia_09VV18_1
IBE_PT_PicoFoia_09VV18_2
IBE_PT_Arrabida_04VV18_9
IBE_PT_Arrabida_04VV18_7
IBE_PT_Arrabida_04VV18_2
IBE_PT_Arrabida_04VV18_4
MAD_PT_SaoJorgeSaoVicente_04VV08_2
MAD_PT_FaialSantana_01VV08_3
MAD_PT_DasQueimadas_03VV08_3
MAD_PT_SaoJorgeSaoVicente_04VV08_3
MAD_PT_Santa_05VV08_2
MAD_PT_SaoJorgeSaoVicente_04VV08_4
MAD_PT_DasQueimadas_03VV08_1
MAD_PT_DasQueimadas_03VV08_2
MAD_PT_Santa_05VV08_3
MAD_PT_FaialSantana_01VV08_2
MAD_PT_FaialSantana_01VV08_1
MAD_PT_Funchal_9818MV
MAD_PT_Funchal_3394CN
MAD_PT_SaoJorgeSaoVicente_04VV08_1
MAD_PT_CaboPontadoPargo_1
MAD_PT_CaboPontadoPargo_2
MAD_PT_Santa_05VV08_6
MAD_PT_Santa_05VV08_5
MAD_PT_Santa_05VV08_4
MAD_PT_CaboPontadoPargo_3
MAD_PT_CaboPontadoPargo_4
c85m4p2r3 (RAxML)
0.003

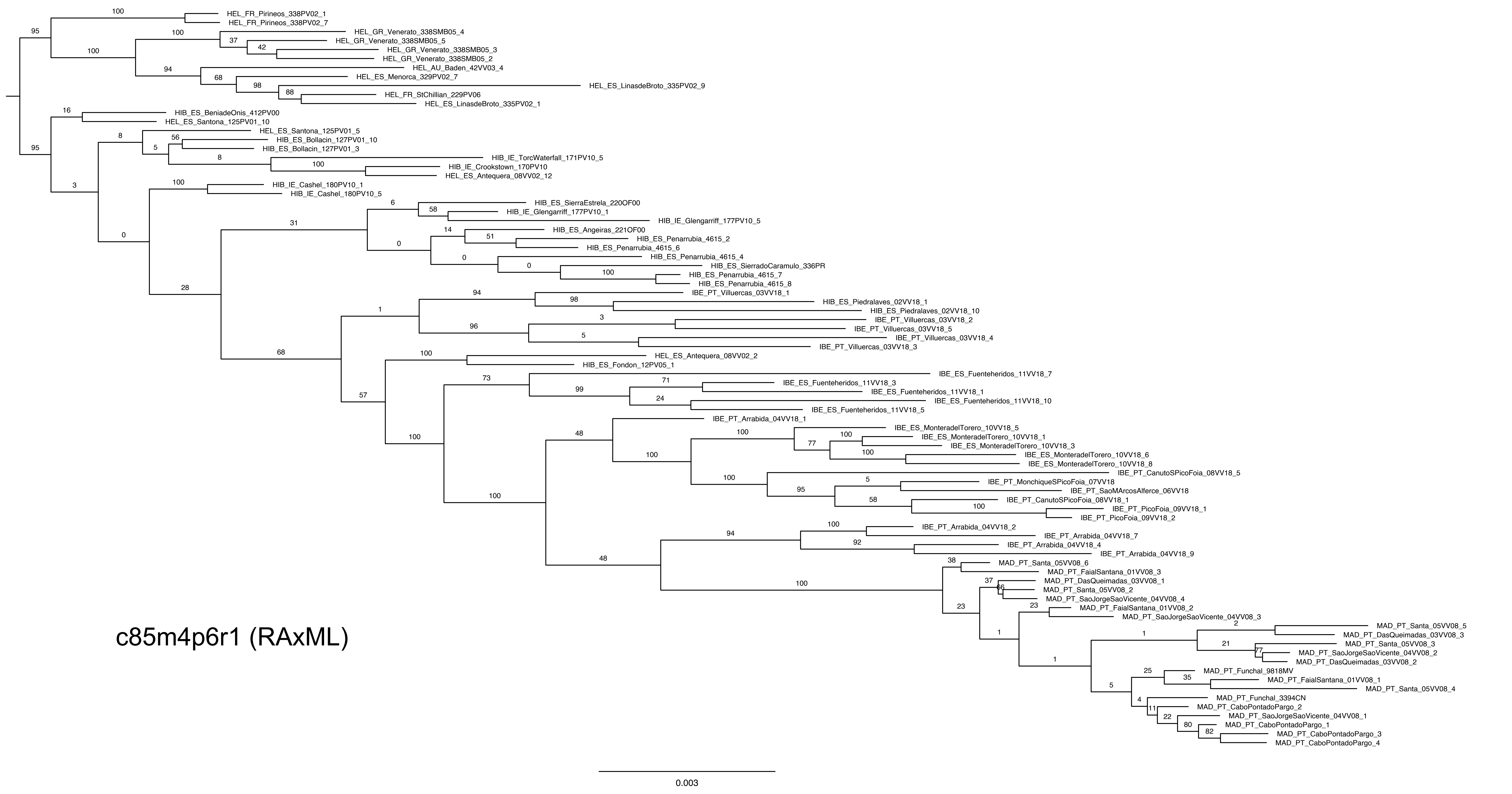

HEL_FR_Pirineos_338PV02_1
HEL_FR_Pirineos_338PV02_7
HEL_GR_Venerato_338SMB05_4
HEL_GR_Venerato_338SMB05_5
HEL_GR_Venerato_338SMB05_3
HEL_GR_Venerato_338SMB05_2
HEL_AU_Baden_42VV03_4
HEL_ES_Menorca_329PV02_7
HEL_ES_LinasdeBroto_335PV02_9
HEL_FR_StChillian_229PV06
HEL_ES_LinasdeBroto_335PV02_1
HIB_ES_BeniadeOnis_412PV00
HEL_ES_Santona_125PV01_10
HEL_ES_Santona_125PV01_5
HIB_ES_Bollacin_127PV01_10
HIB_ES_Bollacin_127PV01_3
HIB_IE_TorcWaterfall_171PV10_5
HIB_IE_Crookstown_170PV10
HEL_ES_Antequera_08VV02_12
HIB_IE_Cashel_180PV10_1
HIB_IE_Cashel_180PV10_5
HIB_ES_SierraEstrela_220OF00
HIB_IE_Glengarriff_177PV10_1
HIB_IE_Glengarriff_177PV10_5
HIB_ES_Angeiras_221OF00
HIB_ES_Penarrubia_4615_2
HIB_ES_Penarrubia_4615_6
HIB_ES_Penarrubia_4615_4
HIB_ES_SierradoCaramulo_336PR
HIB_ES_Penarrubia_4615_7
HIB_ES_Penarrubia_4615_8
IBE_PT_Villuercas_03VV18_1
HIB_ES_Piedralaves_02VV18_1
HIB_ES_Piedralaves_02VV18_10
IBE_PT_Villuercas_03VV18_2
IBE_PT_Villuercas_03VV18_5
IBE_PT_Villuercas_03VV18_4
IBE_PT_Villuercas_03VV18_3
HEL_ES_Antequera_08VV02_2
HIB_ES_Fondon_12PV05_1
IBE_ES_Fuenteheridos_11VV18_7
IBE_ES_Fuenteheridos_11VV18_3
IBE_ES_Fuenteheridos_11VV18_1
IBE_ES_Fuenteheridos_11VV18_10
IBE_ES_Fuenteheridos_11VV18_5
IBE_PT_Arrabida_04VV18_1
IBE_ES_MonteradelTorero_10VV18_5
IBE_ES_MonteradelTorero_10VV18_1
IBE_ES_MonteradelTorero_10VV18_3
IBE_ES_MonteradelTorero_10VV18_6
IBE_ES_MonteradelTorero_10VV18_8
IBE_PT_CanutoSPicoFoia_08VV18_5
IBE_PT_MonchiqueSPicoFoia_07VV18
IBE_PT_SaoMArcosAlferce_06VV18
IBE_PT_CanutoSPicoFoia_08VV18_1
IBE_PT_PicoFoia_09VV18_1
IBE_PT_PicoFoia_09VV18_2
IBE_PT_Arrabida_04VV18_2
IBE_PT_Arrabida_04VV18_7
IBE_PT_Arrabida_04VV18_4
IBE_PT_Arrabida_04VV18_9
MAD_PT_Santa_05VV08_6
MAD_PT_FaialSantana_01VV08_3
MAD_PT_DasQueimadas_03VV08_1
MAD_PT_Santa_05VV08_2
MAD_PT_SaoJorgeSaoVicente_04VV08_4
MAD_PT_FaialSantana_01VV08_2
MAD_PT_SaoJorgeSaoVicente_04VV08_3
MAD_PT_Santa_05VV08_5
MAD_PT_DasQueimadas_03VV08_3
MAD_PT_Santa_05VV08_3
MAD_PT_SaoJorgeSaoVicente_04VV08_2
MAD_PT_DasQueimadas_03VV08_2
MAD_PT_Funchal_9818MV
MAD_PT_FaialSantana_01VV08_1
MAD_PT_Santa_05VV08_4
MAD_PT_Funchal_3394CN
MAD_PT_CaboPontadoPargo_2
MAD_PT_SaoJorgeSaoVicente_04VV08_1
MAD_PT_CaboPontadoPargo_1
MAD_PT_CaboPontadoPargo_3
MAD_PT_CaboPontadoPargo_4
c85m4p6r1 (RAxML)
0.003

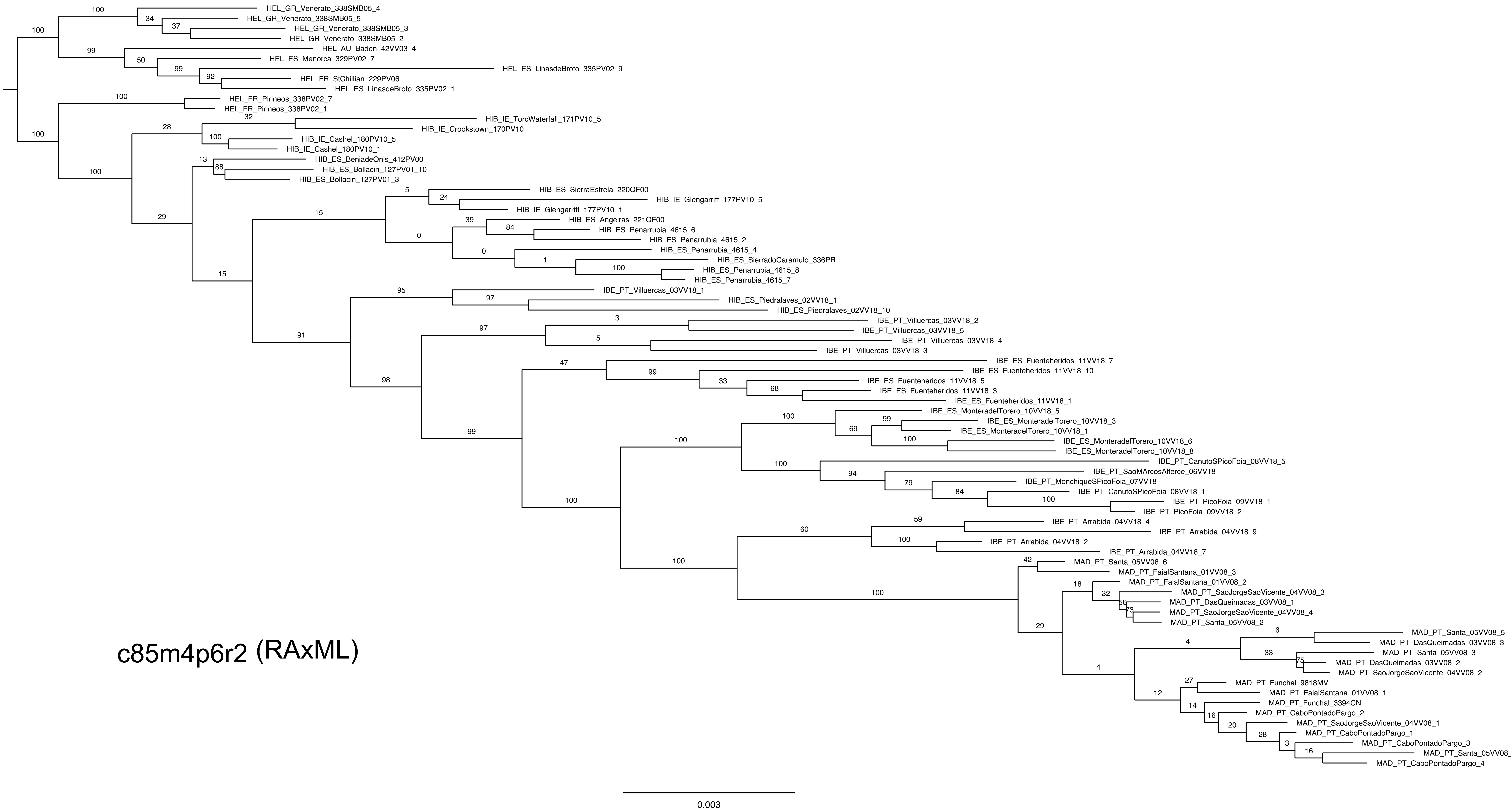
HEL_GR_Venerato_338SMB05_4
HEL_GR_Venerato_338SMB05_5
HEL_GR_Venerato_338SMB05_3
HEL_GR_Venerato_338SMB05_2
HEL_AU_Baden_42VV03_4
HEL_ES_Menorca_329PV02_7
HEL_ES_LinasdeBroto_335PV02_9
HEL_FR_StChillian_229PV06
HEL_ES_LinasdeBroto_335PV02_1
HEL_FR_Pirineos_338PV02_7
HEL_FR_Pirineos_338PV02_1
HIB_IE_TorcWaterfall_171PV10_5
HIB_IE_Crookstown_170PV10
HIB_IE_Cashel_180PV10_5
HIB_IE_Cashel_180PV10_1
HIB_ES_BeniadeOnis_412PV00
HIB_ES_Bollacin_127PV01_10
HIB_ES_Bollacin_127PV01_3
HIB_ES_SierraEstrela_220OF00
HIB_IE_Glengarriff_177PV10_5
HIB_IE_Glengarriff_177PV10_1
HIB_ES_Angeiras_221OF00
HIB_ES_Penarrubia_4615_6
HIB_ES_Penarrubia_4615_2
HIB_ES_Penarrubia_4615_4
HIB_ES_SierradoCaramulo_336PR
HIB_ES_Penarrubia_4615_8
HIB_ES_Penarrubia_4615_7
IBE_PT_Villuercas_03VV18_1
HIB_ES_Piedralaves_02VV18_1
HIB_ES_Piedralaves_02VV18_10
IBE_PT_Villuercas_03VV18_2
IBE_PT_Villuercas_03VV18_5
IBE_PT_Villuercas_03VV18_4
IBE_PT_Villuercas_03VV18_3
IBE_ES_Fuenteheridos_11VV18_7
IBE_ES_Fuenteheridos_11VV18_10
IBE_ES_Fuenteheridos_11VV18_5
IBE_ES_Fuenteheridos_11VV18_3
IBE_ES_Fuenteheridos_11VV18_1
IBE_ES_MonteradelTorero_10VV18_5
IBE_ES_MonteradelTorero_10VV18_3
IBE_ES_MonteradelTorero_10VV18_1
IBE_ES_MonteradelTorero_10VV18_6
IBE_ES_MonteradelTorero_10VV18_8
IBE_PT_CanutoSPicoFoia_08VV18_5
IBE_PT_SaoMArcosAlferce_06VV18
IBE_PT_MonchiqueSPicoFoia_07VV18
IBE_PT_CanutoSPicoFoia_08VV18_1
IBE_PT_PicoFoia_09VV18_1
IBE_PT_PicoFoia_09VV18_2
IBE_PT_Arrabida_04VV18_4
IBE_PT_Arrabida_04VV18_9
IBE_PT_Arrabida_04VV18_2
IBE_PT_Arrabida_04VV18_7
MAD_PT_Santa_05VV08_6
MAD_PT_FaialSantana_01VV08_3
MAD_PT_FaialSantana_01VV08_2
MAD_PT_SaoJorgeSaoVicente_04VV08_3
MAD_PT_DasQueimadas_03VV08_1
MAD_PT_SaoJorgeSaoVicente_04VV08_4
MAD_PT_Santa_05VV08_2
MAD_PT_Santa_05VV08_5
MAD_PT_DasQueimadas_03VV08_3
MAD_PT_Santa_05VV08_3
MAD_PT_DasQueimadas_03VV08_2
MAD_PT_SaoJorgeSaoVicente_04VV08_2
MAD_PT_Funchal_9818MV
MAD_PT_FaialSantana_01VV08_1
MAD_PT_Funchal_3394CN
MAD_PT_CaboPontadoPargo_2
MAD_PT_SaoJorgeSaoVicente_04VV08_1
MAD_PT_CaboPontadoPargo_1
MAD_PT_CaboPontadoPargo_3
MAD_PT_Santa_05VV08_4
MAD_PT_CaboPontadoPargo_4
c85m4p6r2 (RAxML)
0.003

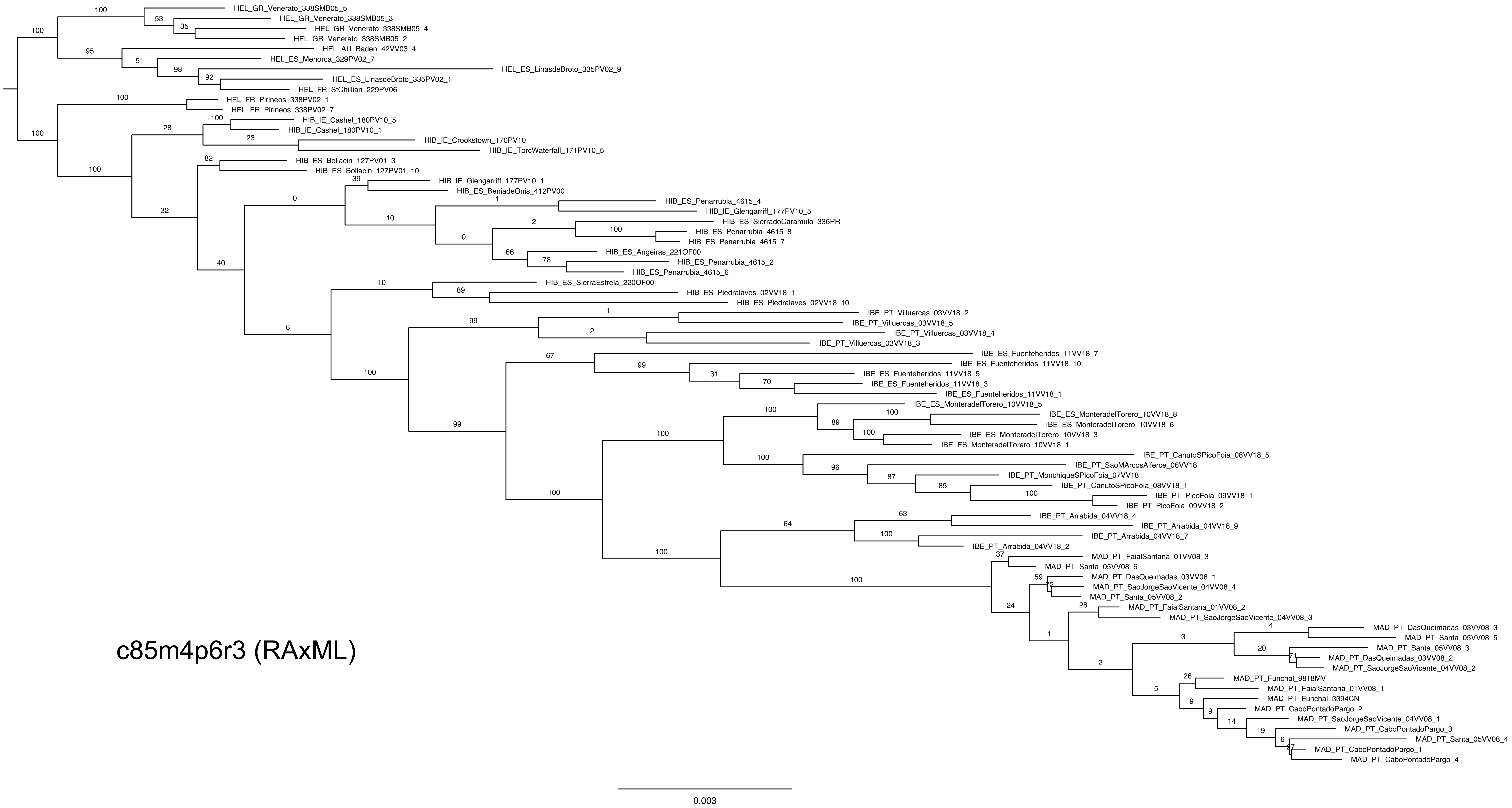

HEL_GR_Venerato_338SMB05_5
HEL_GR_Venerato_338SMB05_3
HEL_GR_Venerato_338SMB05_4
HEL_GR_Venerato_338SMB05_2
HEL_AU_Baden_42VV03_4
HEL_ES_Menorca_329PV02_7
HEL_ES_LinasdeBroto_335PV02_9
HEL_ES_LinasdeBroto_335PV02_1
HEL_FR_StChillian_229PV06
HEL_FR_Pirineos_338PV02_1
HEL_FR_Pirineos_338PV02_7
HIB_IE_Cashel_180PV10_5
HIB_IE_Cashel_180PV10_1
HIB_IE_Crookstown_170PV10
HIB_IE_TorcWaterfall_171PV10_5
HIB_ES_Bollacin_127PV01_3
HIB_ES_Bollacin_127PV01_10
HIB_IE_Glengarriff_177PV10_1
HIB_ES_BeniadeOnis_412PV00
HIB_ES_Penarrubia_4615_4
HIB_IE_Glengarriff_177PV10_5
HIB_ES_SierradoCaramulo_336PR
HIB_ES_Penarrubia_4615_8
HIB_ES_Penarrubia_4615_7
HIB_ES_Angeiras_221OF00
HIB_ES_Penarrubia_4615_2
HIB_ES_Penarrubia_4615_6
HIB_ES_SierraEstrela_220OF00
HIB_ES_Piedralaves_02VV18_1
HIB_ES_Piedralaves_02VV18_10
IBE_PT_Villuercas_03VV18_2
IBE_PT_Villuercas_03VV18_5
IBE_PT_Villuercas_03VV18_4
IBE_PT_Villuercas_03VV18_3
IBE_ES_Fuenteheridos_11VV18_7
IBE_ES_Fuenteheridos_11VV18_10
IBE_ES_Fuenteheridos_11VV18_5
IBE_ES_Fuenteheridos_11VV18_3
IBE_ES_Fuenteheridos_11VV18_1
IBE_ES_MonteradelTorero_10VV18_5
IBE_ES_MonteradelTorero_10VV18_8
IBE_ES_MonteradelTorero_10VV18_6
IBE_ES_MonteradelTorero_10VV18_3
IBE_ES_MonteradelTorero_10VV18_1
IBE_PT_CanutoSPicoFoia_08VV18_5
IBE_PT_SaoMArcosAlferce_06VV18
IBE_PT_MonchiqueSPicoFoia_07VV18
IBE_PT_CanutoSPicoFoia_08VV18_1
IBE_PT_PicoFoia_09VV18_1
IBE_PT_PicoFoia_09VV18_2
IBE_PT_Arrabida_04VV18_4
IBE_PT_Arrabida_04VV18_9
IBE_PT_Arrabida_04VV18_7
IBE_PT_Arrabida_04VV18_2
MAD_PT_FaialSantana_01VV08_3
MAD_PT_Santa_05VV08_6
MAD_PT_DasQueimadas_03VV08_1
MAD_PT_SaoJorgeSaoVicente_04VV08_4
MAD_PT_Santa_05VV08_2
MAD_PT_FaialSantana_01VV08_2
MAD_PT_SaoJorgeSaoVicente_04VV08_3
MAD_PT_DasQueimadas_03VV08_3
MAD_PT_Santa_05VV08_5
MAD_PT_Santa_05VV08_3
MAD_PT_DasQueimadas_03VV08_2
MAD_PT_SaoJorgeSaoVicente_04VV08_2
MAD_PT_Funchal_9818MV
MAD_PT_FaialSantana_01VV08_1
MAD_PT_Funchal_3394CN
MAD_PT_CaboPontadoPargo_2
MAD_PT_SaoJorgeSaoVicente_04VV08_1
MAD_PT_CaboPontadoPargo_3
MAD_PT_Santa_05VV08_4
MAD_PT_CaboPontadoPargo_1
MAD_PT_CaboPontadoPargo_4
c85m4p6r3 (RAxML)
0.003

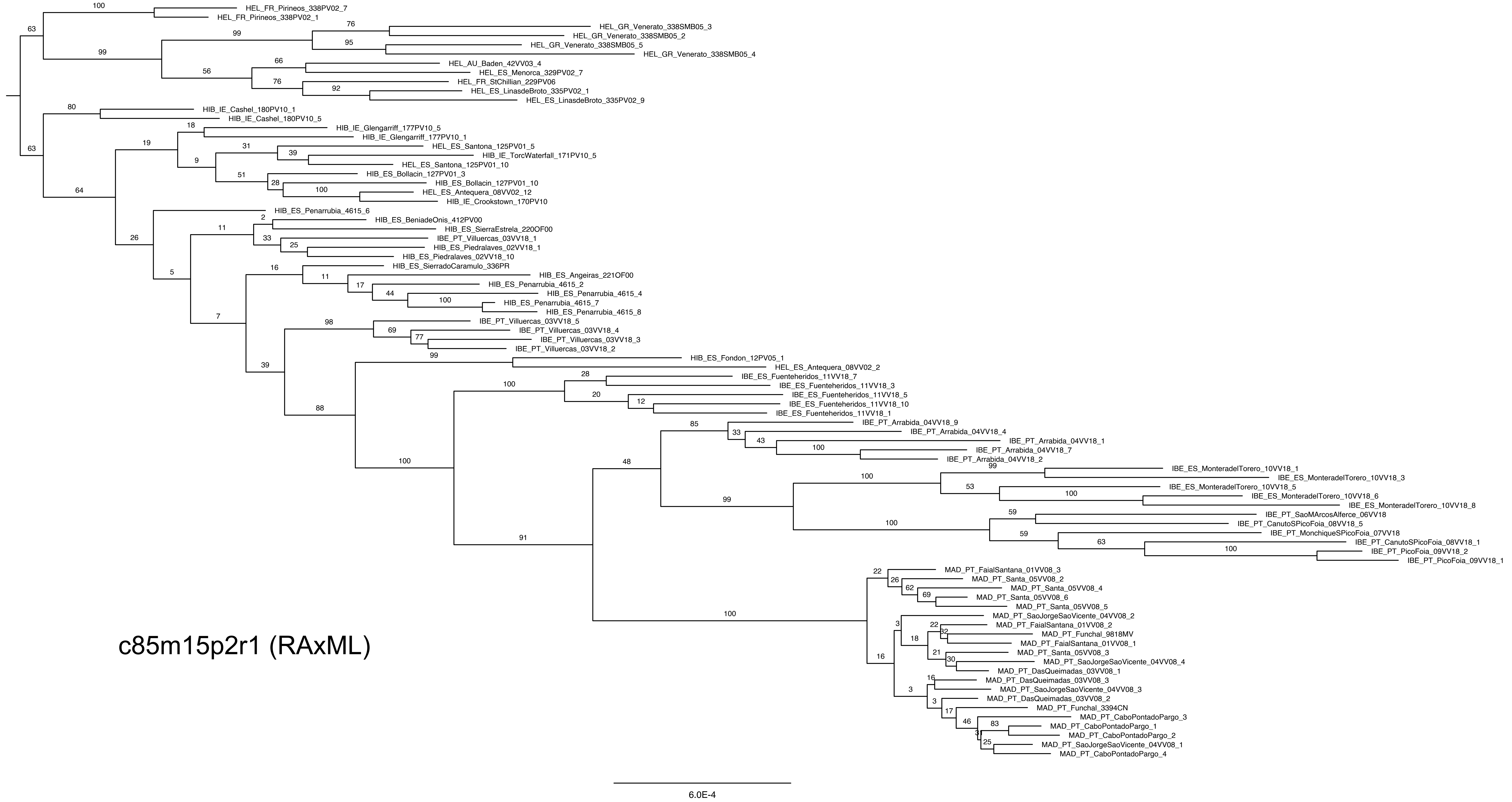
c85m15p2r1 (RAxML)
HEL_FR_Pirineos_338PV02_7
HEL_FR_Pirineos_338PV02_1
HEL_GR_Venerato_338SMB05_3
HEL_GR_Venerato_338SMB05_2
HEL_GR_Venerato_338SMB05_5
HEL_GR_Venerato_338SMB05_4
HEL_AU_Baden_42VV03_4
HEL_ES_Menorca_329PV02_7
HEL_FR_StChillian_229PV06
HEL_ES_LinasdeBroto_335PV02_1
HEL_ES_LinasdeBroto_335PV02_9
HIB_IE_Cashel_180PV10_1
HIB_IE_Cashel_180PV10_5
HIB_IE_Glengarriff_177PV10_5
HIB_IE_Glengarriff_177PV10_1
HEL_ES_Santona_125PV01_5
HIB_IE_TorcWaterfall_171PV10_5
HEL_ES_Santona_125PV01_10
HIB_ES_Bollacin_127PV01_3
HIB_ES_Bollacin_127PV01_10
HEL_ES_Antequera_08VV02_12
HIB_IE_Crookstown_170PV10
HIB_ES_Penarrubia_4615_6
HIB_ES_BeniadeOnis_412PV00
HIB_ES_SierraEstrela_220OF00
IBE_PT_Villuercas_03VV18_1
HIB_ES_Piedralaves_02VV18_1
HIB_ES_Piedralaves_02VV18_10
HIB_ES_SierradoCaramulo_336PR
HIB_ES_Angeiras_221OF00
HIB_ES_Penarrubia_4615_2
HIB_ES_Penarrubia_4615_4
HIB_ES_Penarrubia_4615_7
HIB_ES_Penarrubia_4615_8
IBE_PT_Villuercas_03VV18_5
IBE_PT_Villuercas_03VV18_4
IBE_PT_Villuercas_03VV18_3
IBE_PT_Villuercas_03VV18_2
HIB_ES_Fondon_12PV05_1
HEL_ES_Antequera_08VV02_2
IBE_ES_Fuenteheridos_11VV18_7
IBE_ES_Fuenteheridos_11VV18_3
IBE_ES_Fuenteheridos_11VV18_5
IBE_ES_Fuenteheridos_11VV18_10
IBE_ES_Fuenteheridos_11VV18_1
IBE_PT_Arrabida_04VV18_9
IBE_PT_Arrabida_04VV18_4
IBE_PT_Arrabida_04VV18_1
IBE_PT_Arrabida_04VV18_7
IBE_PT_Arrabida_04VV18_2
IBE_ES_MonteradelTorero_10VV18_1
IBE_ES_MonteradelTorero_10VV18_3
IBE_ES_MonteradelTorero_10VV18_5
IBE_ES_MonteradelTorero_10VV18_6
IBE_ES_MonteradelTorero_10VV18_8
IBE_PT_SaoMArcosAlferce_06VV18
IBE_PT_CanutoSPicoFoia_08VV18_5
IBE_PT_MonchiqueSPicoFoia_07VV18
IBE_PT_CanutoSPicoFoia_08VV18_1
IBE_PT_PicoFoia_09VV18_2
IBE_PT_PicoFoia_09VV18_1
MAD_PT_FaialSantana_01VV08_3
MAD_PT_Santa_05VV08_2
MAD_PT_Santa_05VV08_4
MAD_PT_Santa_05VV08_6
MAD_PT_Santa_05VV08_5
MAD_PT_SaoJorgeSaoVicente_04VV08_2
MAD_PT_FaialSantana_01VV08_2
MAD_PT_Funchal_9818MV
MAD_PT_FaialSantana_01VV08_1
MAD_PT_Santa_05VV08_3
MAD_PT_SaoJorgeSaoVicente_04VV08_4
MAD_PT_DasQueimadas_03VV08_1
MAD_PT_DasQueimadas_03VV08_3
MAD_PT_SaoJorgeSaoVicente_04VV08_3
MAD_PT_DasQueimadas_03VV08_2
MAD_PT_Funchal_3394CN
MAD_PT_CaboPontadoPargo_3
MAD_PT_CaboPontadoPargo_1
MAD_PT_CaboPontadoPargo_2
MAD_PT_SaoJorgeSaoVicente_04VV08_1
MAD_PT_CaboPontadoPargo_4
6.0E-4

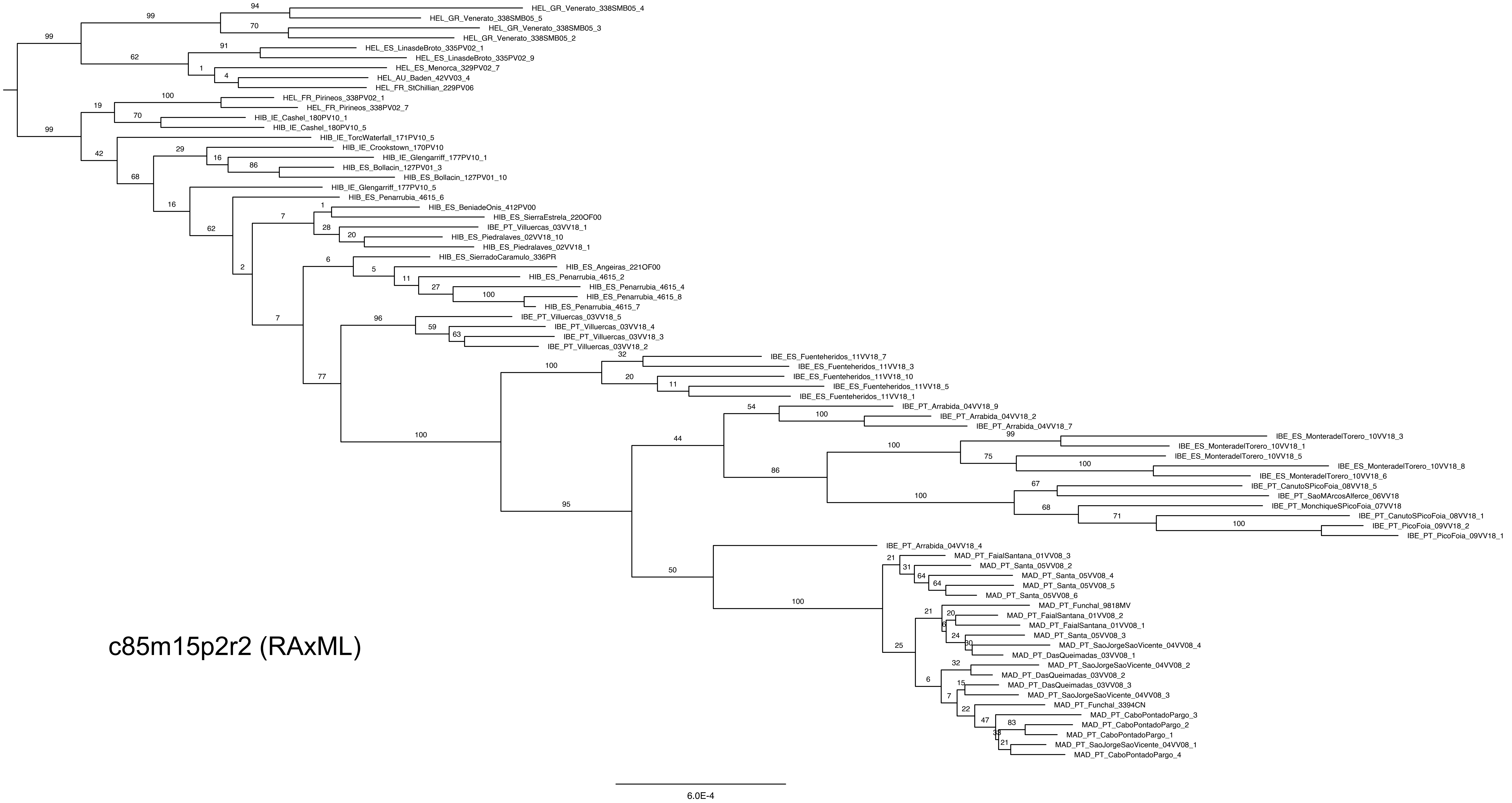

HEL_GR_Venerato_338SMB05_4
HEL_GR_Venerato_338SMB05_5
HEL_GR_Venerato_338SMB05_3
HEL_GR_Venerato_338SMB05_2
HEL_ES_LinasdeBroto_335PV02_1
HEL_ES_LinasdeBroto_335PV02_9
HEL_ES_Menorca_329PV02_7
HEL_AU_Baden_42VV03_4
HEL_FR_StChillian_229PV06
HEL_FR_Pirineos_338PV02_1
HEL_FR_Pirineos_338PV02_7
HIB_IE_Cashel_180PV10_1
HIB_IE_Cashel_180PV10_5
HIB_IE_TorcWaterfall_171PV10_5
HIB_IE_Crookstown_170PV10
HIB_IE_Glengarriff_177PV10_1
HIB_ES_Bollacin_127PV01_3
HIB_ES_Bollacin_127PV01_10
HIB_IE_Glengarriff_177PV10_5
HIB_ES_Penarrubia_4615_6
HIB_ES_BeniadeOnis_412PV00
HIB_ES_SierraEstrela_220OF00
IBE_PT_Villuercas_03VV18_1
HIB_ES_Piedralaves_02VV18_10
HIB_ES_Piedralaves_02VV18_1
HIB_ES_SierradoCaramulo_336PR
HIB_ES_Angeiras_221OF00
HIB_ES_Penarrubia_4615_2
HIB_ES_Penarrubia_4615_4
HIB_ES_Penarrubia_4615_8
HIB_ES_Penarrubia_4615_7
IBE_PT_Villuercas_03VV18_5
IBE_PT_Villuercas_03VV18_4
IBE_PT_Villuercas_03VV18_3
IBE_PT_Villuercas_03VV18_2
IBE_ES_Fuenteheridos_11VV18_7
IBE_ES_Fuenteheridos_11VV18_3
IBE_ES_Fuenteheridos_11VV18_10
IBE_ES_Fuenteheridos_11VV18_5
IBE_ES_Fuenteheridos_11VV18_1
IBE_PT_Arrabida_04VV18_9
IBE_PT_Arrabida_04VV18_2
IBE_PT_Arrabida_04VV18_7
IBE_ES_MonteradelTorero_10VV18_3
IBE_ES_MonteradelTorero_10VV18_1
IBE_ES_MonteradelTorero_10VV18_5
IBE_ES_MonteradelTorero_10VV18_8
IBE_ES_MonteradelTorero_10VV18_6
IBE_PT_CanutoSPicoFoia_08VV18_5
IBE_PT_SaoMArcosAlferce_06VV18
IBE_PT_MonchiqueSPicoFoia_07VV18
IBE_PT_CanutoSPicoFoia_08VV18_1
IBE_PT_PicoFoia_09VV18_2
IBE_PT_PicoFoia_09VV18_1
IBE_PT_Arrabida_04VV18_4
MAD_PT_FaialSantana_01VV08_3
MAD_PT_Santa_05VV08_2
MAD_PT_Santa_05VV08_4
MAD_PT_Santa_05VV08_5
MAD_PT_Santa_05VV08_6
MAD_PT_Funchal_9818MV
MAD_PT_FaialSantana_01VV08_2
MAD_PT_FaialSantana_01VV08_1
MAD_PT_Santa_05VV08_3
MAD_PT_SaoJorgeSaoVicente_04VV08_4
MAD_PT_DasQueimadas_03VV08_1
MAD_PT_SaoJorgeSaoVicente_04VV08_2
MAD_PT_DasQueimadas_03VV08_2
MAD_PT_DasQueimadas_03VV08_3
MAD_PT_SaoJorgeSaoVicente_04VV08_3
MAD_PT_Funchal_3394CN
MAD_PT_CaboPontadoPargo_3
MAD_PT_CaboPontadoPargo_2
MAD_PT_CaboPontadoPargo_1
MAD_PT_SaoJorgeSaoVicente_04VV08_1
MAD_PT_CaboPontadoPargo_4
c85m15p2r2 (RAxML)
6.0E-4

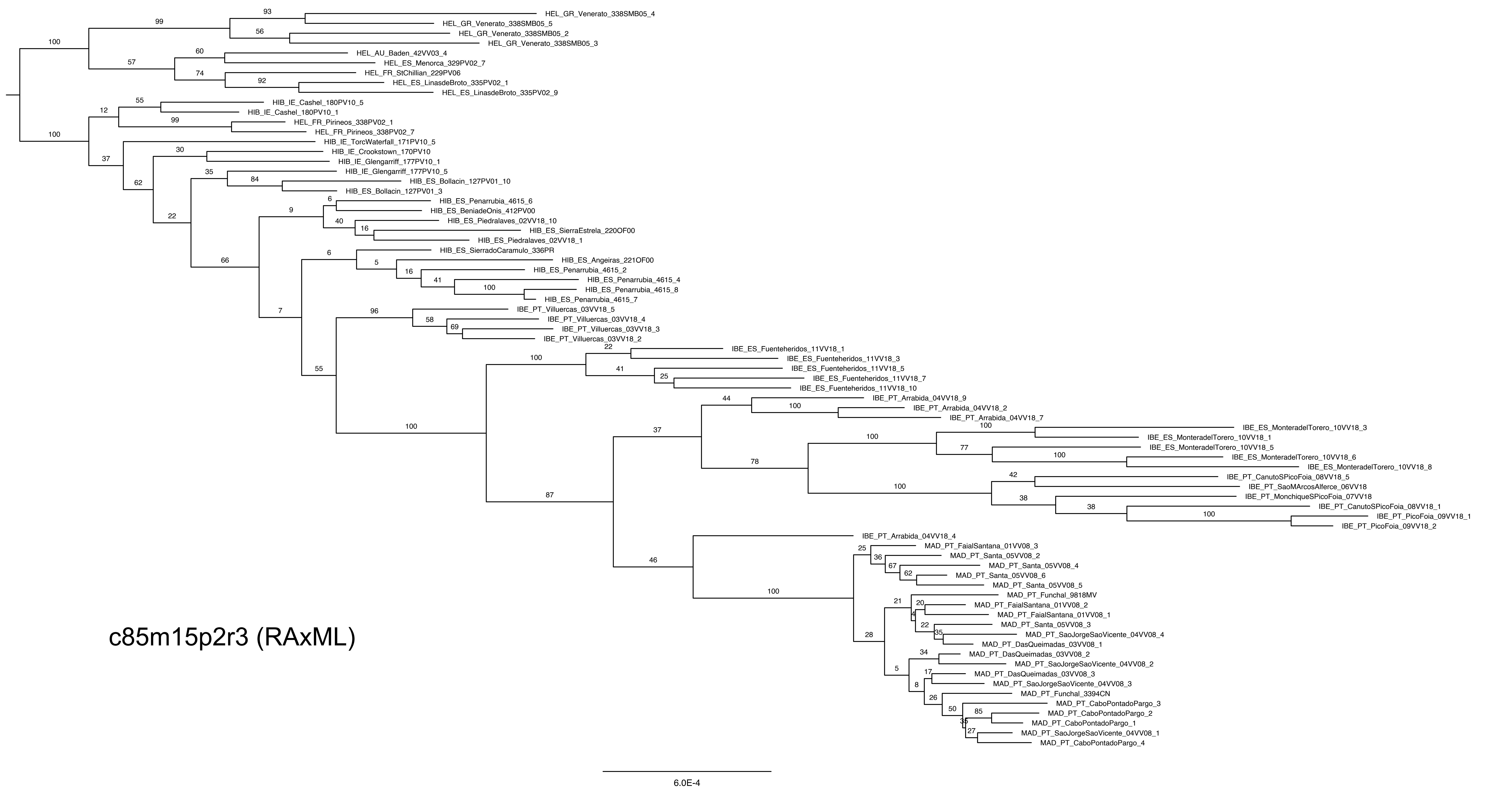
HEL_GR_Venerato_338SMB05_4
HEL_GR_Venerato_338SMB05_5
HEL_GR_Venerato_338SMB05_2
HEL_GR_Venerato_338SMB05_3
HEL_AU_Baden_42VV03_4
HEL_ES_Menorca_329PV02_7
HEL_FR_StChillian_229PV06
HEL_ES_LinasdeBroto_335PV02_1
HEL_ES_LinasdeBroto_335PV02_9
HIB_IE_Cashel_180PV10_5
HIB_IE_Cashel_180PV10_1
HEL_FR_Pirineos_338PV02_1
HEL_FR_Pirineos_338PV02_7
HIB_IE_TorcWaterfall_171PV10_5
HIB_IE_Crookstown_170PV10
HIB_IE_Glengarriff_177PV10_1
HIB_IE_Glengarriff_177PV10_5
HIB_ES_Bollacin_127PV01_10
HIB_ES_Bollacin_127PV01_3
HIB_ES_Penarrubia_4615_6
HIB_ES_BeniadeOnis_412PV00
HIB_ES_Piedralaves_02VV18_10
HIB_ES_SierraEstrela_220OF00
HIB_ES_Piedralaves_02VV18_1
HIB_ES_SierradoCaramulo_336PR
HIB_ES_Angeiras_221OF00
HIB_ES_Penarrubia_4615_2
HIB_ES_Penarrubia_4615_4
HIB_ES_Penarrubia_4615_8
HIB_ES_Penarrubia_4615_7
IBE_PT_Villuercas_03VV18_5
IBE_PT_Villuercas_03VV18_4
IBE_PT_Villuercas_03VV18_3
IBE_PT_Villuercas_03VV18_2
IBE_ES_Fuenteheridos_11VV18_1
IBE_ES_Fuenteheridos_11VV18_3
IBE_ES_Fuenteheridos_11VV18_5
IBE_ES_Fuenteheridos_11VV18_7
IBE_ES_Fuenteheridos_11VV18_10
IBE_PT_Arrabida_04VV18_9
IBE_PT_Arrabida_04VV18_2
IBE_PT_Arrabida_04VV18_7
IBE_ES_MonteradelTorero_10VV18_3
IBE_ES_MonteradelTorero_10VV18_1
IBE_ES_MonteradelTorero_10VV18_5
IBE_ES_MonteradelTorero_10VV18_6
IBE_ES_MonteradelTorero_10VV18_8
IBE_PT_CanutoSPicoFoia_08VV18_5
IBE_PT_SaoMArcosAlferce_06VV18
IBE_PT_MonchiqueSPicoFoia_07VV18
IBE_PT_CanutoSPicoFoia_08VV18_1
IBE_PT_PicoFoia_09VV18_1
IBE_PT_PicoFoia_09VV18_2
IBE_PT_Arrabida_04VV18_4
MAD_PT_FaialSantana_01VV08_3
MAD_PT_Santa_05VV08_2
MAD_PT_Santa_05VV08_4
MAD_PT_Santa_05VV08_6
MAD_PT_Santa_05VV08_5
MAD_PT_Funchal_9818MV
MAD_PT_FaialSantana_01VV08_2
MAD_PT_FaialSantana_01VV08_1
MAD_PT_Santa_05VV08_3
MAD_PT_SaoJorgeSaoVicente_04VV08_4
MAD_PT_DasQueimadas_03VV08_1
MAD_PT_DasQueimadas_03VV08_2
MAD_PT_SaoJorgeSaoVicente_04VV08_2
MAD_PT_DasQueimadas_03VV08_3
MAD_PT_SaoJorgeSaoVicente_04VV08_3
MAD_PT_Funchal_3394CN
MAD_PT_CaboPontadoPargo_3
MAD_PT_CaboPontadoPargo_2
MAD_PT_CaboPontadoPargo_1
MAD_PT_SaoJorgeSaoVicente_04VV08_1
MAD_PT_CaboPontadoPargo_4
c85m15p2r3 (RAxML)
6.0E-4

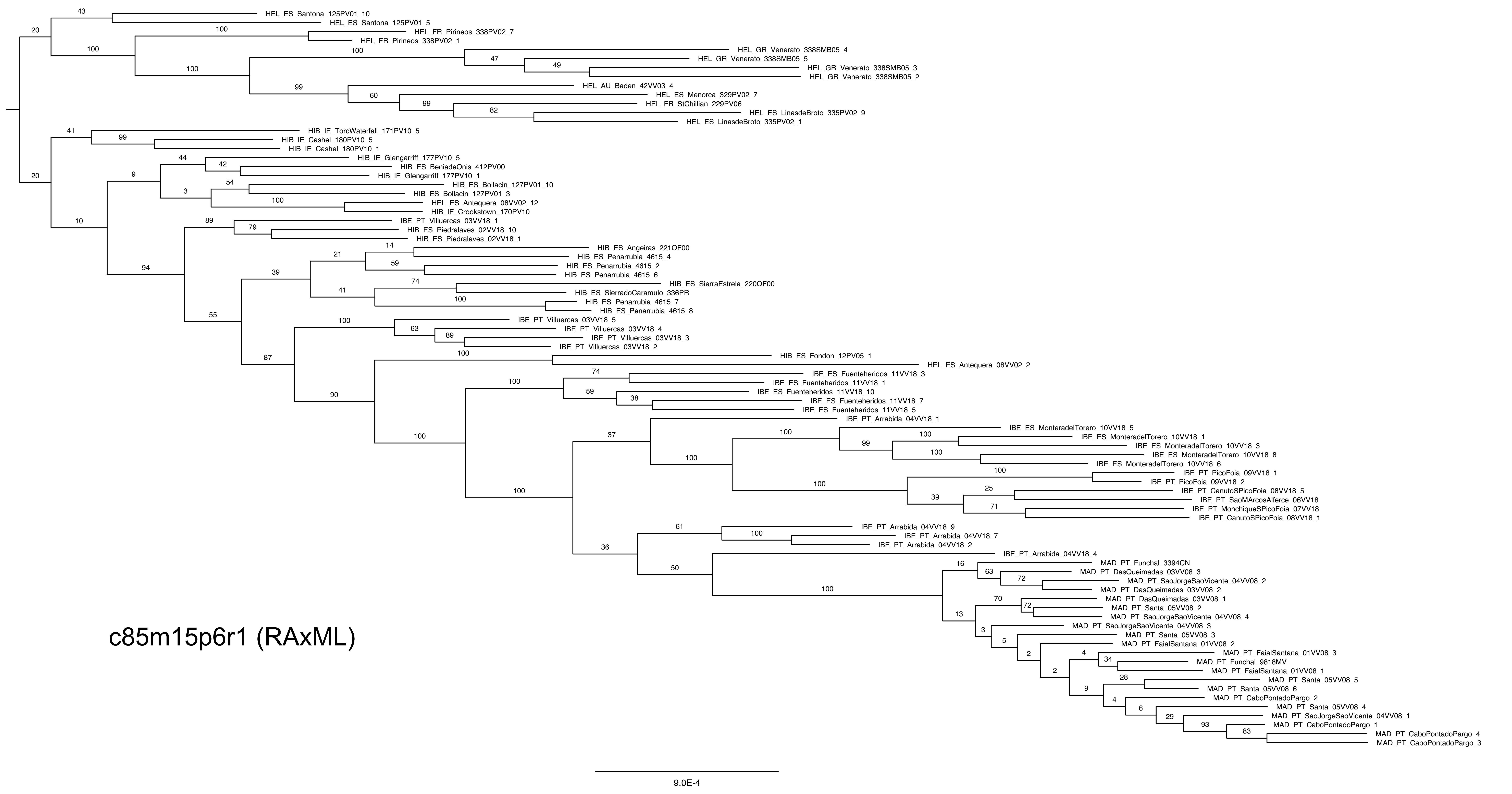
HEL_ES_Santona_125PV01_10
HEL_ES_Santona_125PV01_5
HEL_FR_Pirineos_338PV02_7
HEL_FR_Pirineos_338PV02_1
HEL_GR_Venerato_338SMB05_4
HEL_GR_Venerato_338SMB05_5
HEL_GR_Venerato_338SMB05_3
HEL_GR_Venerato_338SMB05_2
HEL_AU_Baden_42VV03_4
HEL_ES_Menorca_329PV02_7
HEL_FR_StChillian_229PV06
HEL_ES_LinasdeBroto_335PV02_9
HEL_ES_LinasdeBroto_335PV02_1
HIB_IE_TorcWaterfall_171PV10_5
HIB_IE_Cashel_180PV10_5
HIB_IE_Cashel_180PV10_1
HIB_IE_Glengarriff_177PV10_5
HIB_ES_BeniadeOnis_412PV00
HIB_IE_Glengarriff_177PV10_1
HIB_ES_Bollacin_127PV01_10
HIB_ES_Bollacin_127PV01_3
HEL_ES_Antequera_08VV02_12
HIB_IE_Crookstown_170PV10
IBE_PT_Villuercas_03VV18_1
HIB_ES_Piedralaves_02VV18_10
HIB_ES_Piedralaves_02VV18_1
HIB_ES_Angeiras_221OF00
HIB_ES_Penarrubia_4615_4
HIB_ES_Penarrubia_4615_2
HIB_ES_Penarrubia_4615_6
HIB_ES_SierraEstrela_220OF00
HIB_ES_SierradoCaramulo_336PR
HIB_ES_Penarrubia_4615_7
HIB_ES_Penarrubia_4615_8
IBE_PT_Villuercas_03VV18_5
IBE_PT_Villuercas_03VV18_4
IBE_PT_Villuercas_03VV18_3
IBE_PT_Villuercas_03VV18_2
HIB_ES_Fondon_12PV05_1
HEL_ES_Antequera_08VV02_2
IBE_ES_Fuenteheridos_11VV18_3
IBE_ES_Fuenteheridos_11VV18_1
IBE_ES_Fuenteheridos_11VV18_10
IBE_ES_Fuenteheridos_11VV18_7
IBE_ES_Fuenteheridos_11VV18_5
IBE_PT_Arrabida_04VV18_1
IBE_ES_MonteradelTorero_10VV18_5
IBE_ES_MonteradelTorero_10VV18_1
IBE_ES_MonteradelTorero_10VV18_3
IBE_ES_MonteradelTorero_10VV18_8
IBE_ES_MonteradelTorero_10VV18_6
IBE_PT_PicoFoia_09VV18_1
IBE_PT_PicoFoia_09VV18_2
IBE_PT_CanutoSPicoFoia_08VV18_5
IBE_PT_SaoMArcosAlferce_06VV18
IBE_PT_MonchiqueSPicoFoia_07VV18
IBE_PT_CanutoSPicoFoia_08VV18_1
IBE_PT_Arrabida_04VV18_9
IBE_PT_Arrabida_04VV18_7
IBE_PT_Arrabida_04VV18_2
IBE_PT_Arrabida_04VV18_4
MAD_PT_Funchal_3394CN
MAD_PT_DasQueimadas_03VV08_3
MAD_PT_SaoJorgeSaoVicente_04VV08_2
MAD_PT_DasQueimadas_03VV08_2
MAD_PT_DasQueimadas_03VV08_1
MAD_PT_Santa_05VV08_2
MAD_PT_SaoJorgeSaoVicente_04VV08_4
MAD_PT_SaoJorgeSaoVicente_04VV08_3
MAD_PT_Santa_05VV08_3
MAD_PT_FaialSantana_01VV08_2
MAD_PT_FaialSantana_01VV08_3
MAD_PT_Funchal_9818MV
MAD_PT_FaialSantana_01VV08_1
MAD_PT_Santa_05VV08_5
MAD_PT_Santa_05VV08_6
MAD_PT_CaboPontadoPargo_2
MAD_PT_Santa_05VV08_4
MAD_PT_SaoJorgeSaoVicente_04VV08_1
MAD_PT_CaboPontadoPargo_1
MAD_PT_CaboPontadoPargo_4
MAD_PT_CaboPontadoPargo_3
c85m15p6r1 (RAxML)
9.0E-4

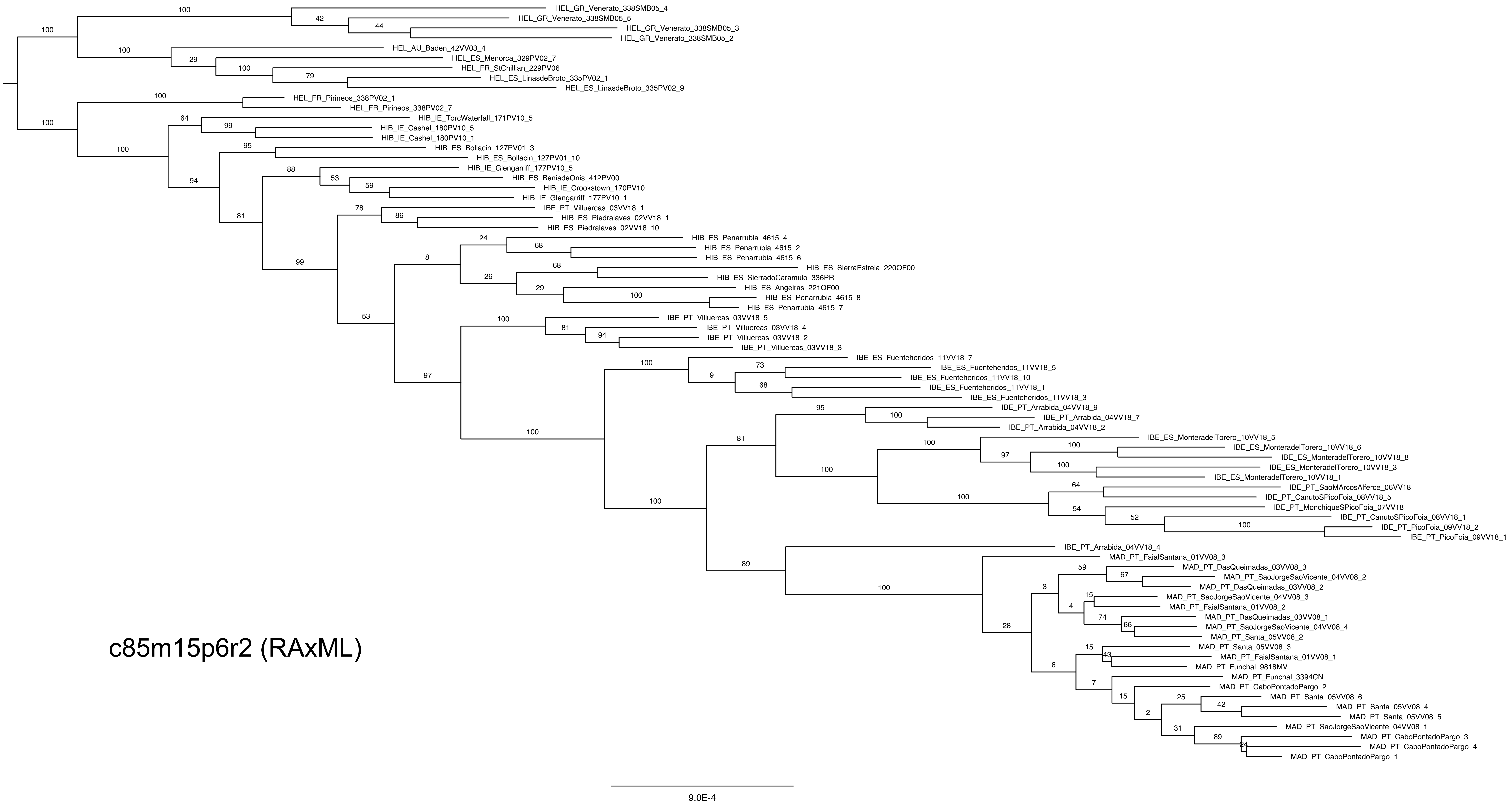
HEL_GR_Venerato_338SMB05_4
HEL_GR_Venerato_338SMB05_5
HEL_GR_Venerato_338SMB05_3
HEL_GR_Venerato_338SMB05_2
HEL_AU_Baden_42VV03_4
HEL_ES_Menorca_329PV02_7
HEL_FR_StChillian_229PV06
HEL_ES_LinasdeBroto_335PV02_1
HEL_ES_LinasdeBroto_335PV02_9
HEL_FR_Pirineos_338PV02_1
HEL_FR_Pirineos_338PV02_7
HIB_IE_TorcWaterfall_171PV10_5
HIB_IE_Cashel_180PV10_5
HIB_IE_Cashel_180PV10_1
HIB_ES_Bollacin_127PV01_3
HIB_ES_Bollacin_127PV01_10
HIB_IE_Glengarriff_177PV10_5
HIB_ES_BeniadeOnis_412PV00
HIB_IE_Crookstown_170PV10
HIB_IE_Glengarriff_177PV10_1
IBE_PT_Villuercas_03VV18_1
HIB_ES_Piedralaves_02VV18_1
HIB_ES_Piedralaves_02VV18_10
HIB_ES_Penarrubia_4615_4
HIB_ES_Penarrubia_4615_2
HIB_ES_Penarrubia_4615_6
HIB_ES_SierraEstrela_220OF00
HIB_ES_SierradoCaramulo_336PR
HIB_ES_Angeiras_221OF00
HIB_ES_Penarrubia_4615_8
HIB_ES_Penarrubia_4615_7
IBE_PT_Villuercas_03VV18_5
IBE_PT_Villuercas_03VV18_4
IBE_PT_Villuercas_03VV18_2
IBE_PT_Villuercas_03VV18_3
IBE_ES_Fuenteheridos_11VV18_7
IBE_ES_Fuenteheridos_11VV18_5
IBE_ES_Fuenteheridos_11VV18_10
IBE_ES_Fuenteheridos_11VV18_1
IBE_ES_Fuenteheridos_11VV18_3
IBE_PT_Arrabida_04VV18_9
IBE_PT_Arrabida_04VV18_7
IBE_PT_Arrabida_04VV18_2
IBE_ES_MonteradelTorero_10VV18_5
IBE_ES_MonteradelTorero_10VV18_6
IBE_ES_MonteradelTorero_10VV18_8
IBE_ES_MonteradelTorero_10VV18_3
IBE_ES_MonteradelTorero_10VV18_1
IBE_PT_SaoMArcosAlferce_06VV18
IBE_PT_CanutoSPicoFoia_08VV18_5
IBE_PT_MonchiqueSPicoFoia_07VV18
IBE_PT_CanutoSPicoFoia_08VV18_1
IBE_PT_PicoFoia_09VV18_2
IBE_PT_PicoFoia_09VV18_1
IBE_PT_Arrabida_04VV18_4
MAD_PT_FaialSantana_01VV08_3
MAD_PT_DasQueimadas_03VV08_3
MAD_PT_SaoJorgeSaoVicente_04VV08_2
MAD_PT_DasQueimadas_03VV08_2
MAD_PT_SaoJorgeSaoVicente_04VV08_3
MAD_PT_FaialSantana_01VV08_2
MAD_PT_DasQueimadas_03VV08_1
MAD_PT_SaoJorgeSaoVicente_04VV08_4
MAD_PT_Santa_05VV08_2
MAD_PT_Santa_05VV08_3
MAD_PT_FaialSantana_01VV08_1
MAD_PT_Funchal_9818MV
MAD_PT_Funchal_3394CN
MAD_PT_CaboPontadoPargo_2
MAD_PT_Santa_05VV08_6
MAD_PT_Santa_05VV08_4
MAD_PT_Santa_05VV08_5
MAD_PT_SaoJorgeSaoVicente_04VV08_1
MAD_PT_CaboPontadoPargo_3
MAD_PT_CaboPontadoPargo_4
MAD_PT_CaboPontadoPargo_1
c85m15p6r2 (RAxML)
9.0E-4

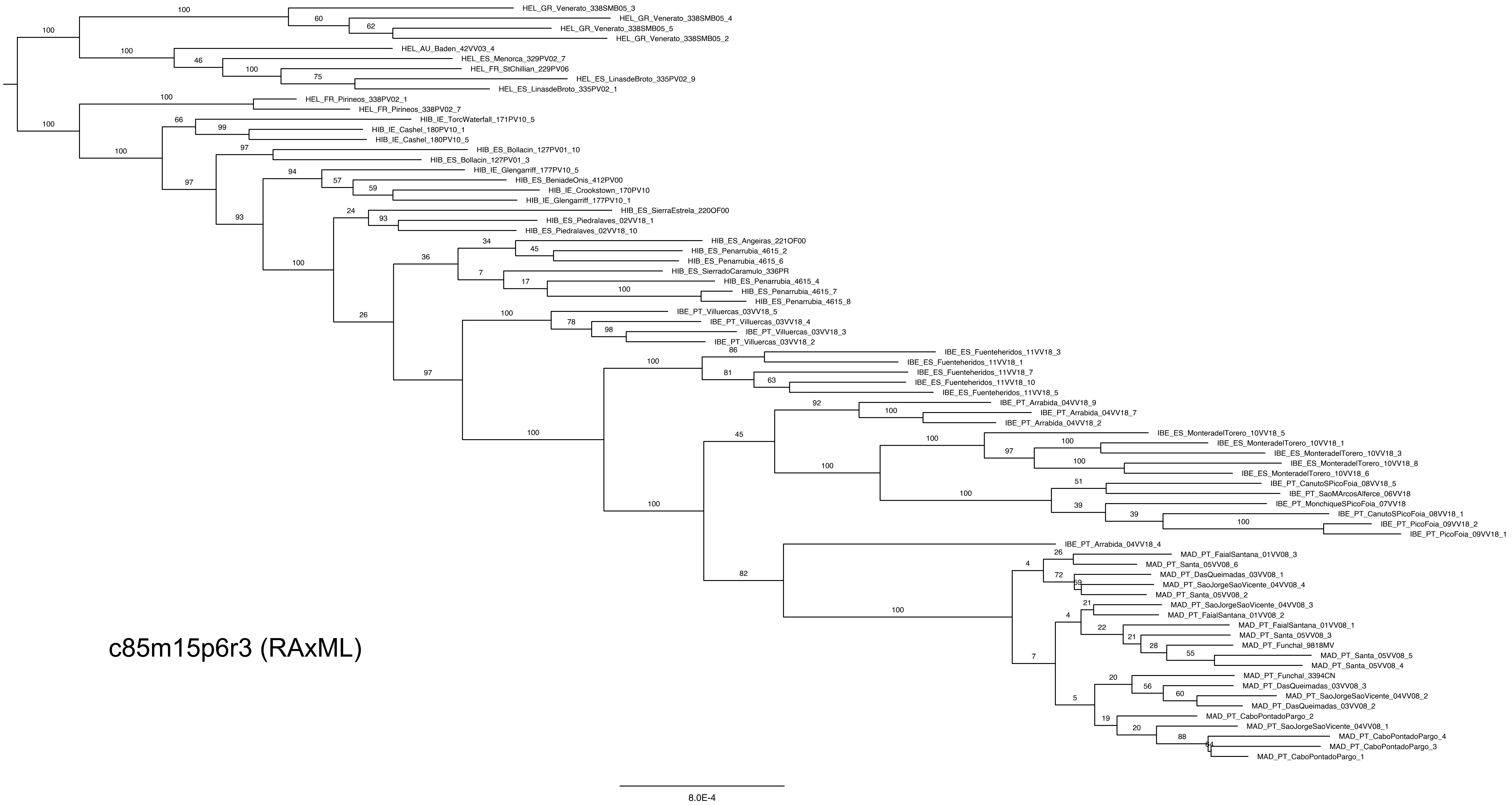
c85m15p6r3 (RAxML)
HEL_GR_Venerato_338SMB05_3
HEL_GR_Venerato_338SMB05_4
HEL_GR_Venerato_338SMB05_5
HEL_GR_Venerato_338SMB05_2
HEL_AU_Baden_42VV03_4
HEL_ES_Menorca_329PV02_7
HEL_FR_StChillian_229PV06
HEL_ES_LinasdeBroto_335PV02_9
HEL_ES_LinasdeBroto_335PV02_1
HEL_FR_Pirineos_338PV02_1
HEL_FR_Pirineos_338PV02_7
HIB_IE_TorcWaterfall_171PV10_5
HIB_IE_Cashel_180PV10_1
HIB_IE_Cashel_180PV10_5
HIB_ES_Bollacin_127PV01_10
HIB_ES_Bollacin_127PV01_3
HIB_IE_Glengarriff_177PV10_5
HIB_ES_BeniadeOnis_412PV00
HIB_IE_Crookstown_170PV10
HIB_IE_Glengarriff_177PV10_1
HIB_ES_SierraEstrela_220OF00
HIB_ES_Piedralaves_02VV18_1
HIB_ES_Piedralaves_02VV18_10
HIB_ES_Angeiras_221OF00
HIB_ES_Penarrubia_4615_2
HIB_ES_Penarrubia_4615_6
HIB_ES_SierradoCaramulo_336PR
HIB_ES_Penarrubia_4615_4
HIB_ES_Penarrubia_4615_7
HIB_ES_Penarrubia_4615_8
IBE_PT_Villuercas_03VV18_5
IBE_PT_Villuercas_03VV18_4
IBE_PT_Villuercas_03VV18_3
IBE_PT_Villuercas_03VV18_2
IBE_ES_Fuenteheridos_11VV18_3
IBE_ES_Fuenteheridos_11VV18_1
IBE_ES_Fuenteheridos_11VV18_7
IBE_ES_Fuenteheridos_11VV18_10
IBE_ES_Fuenteheridos_11VV18_5
IBE_PT_Arrabida_04VV18_9
IBE_PT_Arrabida_04VV18_7
IBE_PT_Arrabida_04VV18_2
IBE_ES_MonteradelTorero_10VV18_5
IBE_ES_MonteradelTorero_10VV18_1
IBE_ES_MonteradelTorero_10VV18_3
IBE_ES_MonteradelTorero_10VV18_8
IBE_ES_MonteradelTorero_10VV18_6
IBE_PT_CanutoSPicoFoia_08VV18_5
IBE_PT_SaoMArcosAlferce_06VV18
IBE_PT_MonchiqueSPicoFoia_07VV18
IBE_PT_CanutoSPicoFoia_08VV18_1
IBE_PT_PicoFoia_09VV18_2
IBE_PT_PicoFoia_09VV18_1
IBE_PT_Arrabida_04VV18_4
MAD_PT_FaialSantana_01VV08_3
MAD_PT_Santa_05VV08_6
MAD_PT_DasQueimadas_03VV08_1
MAD_PT_SaoJorgeSaoVicente_04VV08_4
MAD_PT_Santa_05VV08_2
MAD_PT_SaoJorgeSaoVicente_04VV08_3
MAD_PT_FaialSantana_01VV08_2
MAD_PT_FaialSantana_01VV08_1
MAD_PT_Santa_05VV08_3
MAD_PT_Funchal_9818MV
MAD_PT_Santa_05VV08_5
MAD_PT_Santa_05VV08_4
MAD_PT_Funchal_3394CN
MAD_PT_DasQueimadas_03VV08_3
MAD_PT_SaoJorgeSaoVicente_04VV08_2
MAD_PT_DasQueimadas_03VV08_2
MAD_PT_CaboPontadoPargo_2
MAD_PT_SaoJorgeSaoVicente_04VV08_1
MAD_PT_CaboPontadoPargo_4
MAD_PT_CaboPontadoPargo_3
MAD_PT_CaboPontadoPargo_1
8.0E-4

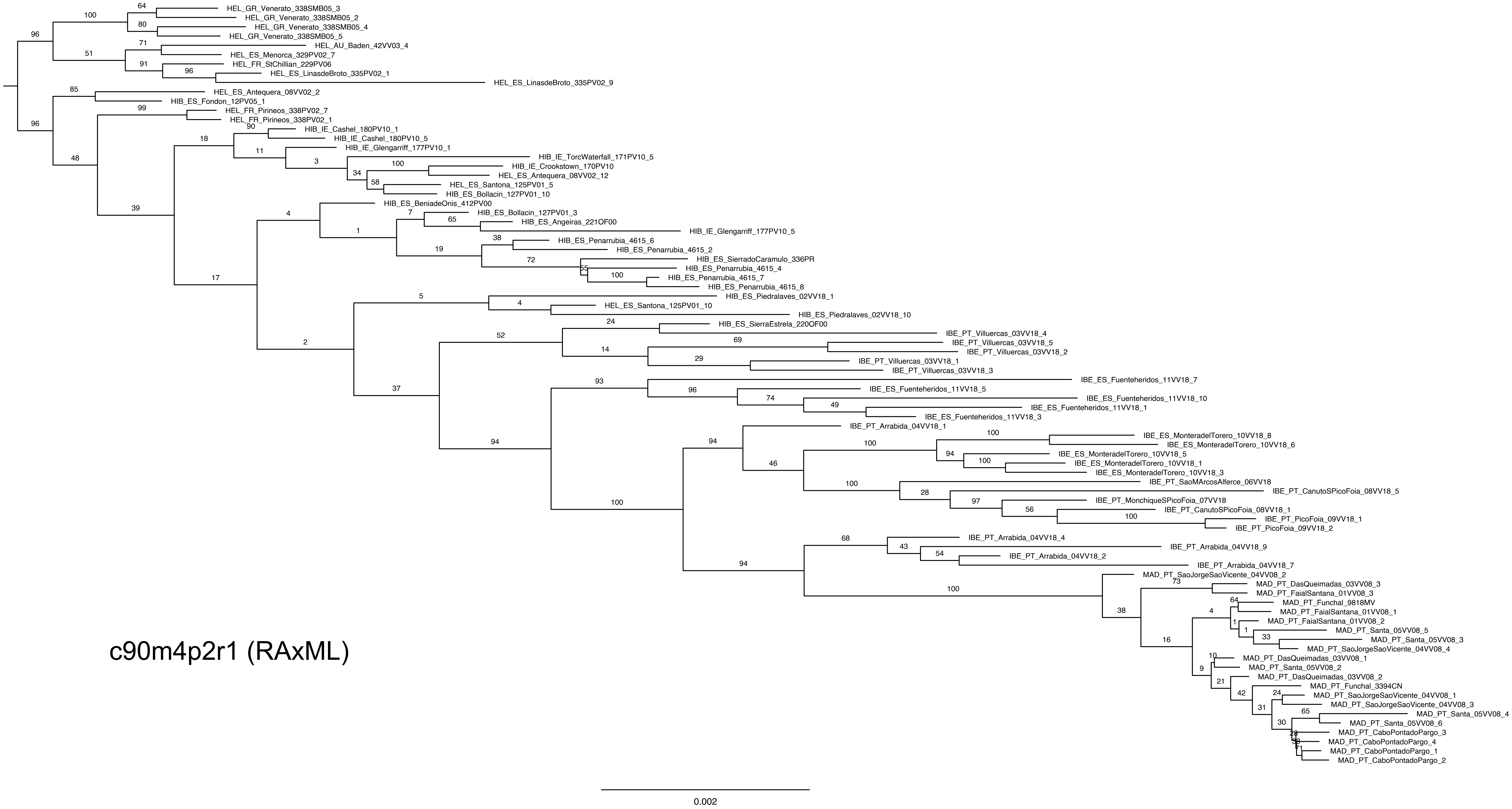
HEL_GR_Venerato_338SMB05_3
HEL_GR_Venerato_338SMB05_2
HEL_GR_Venerato_338SMB05_4
HEL_GR_Venerato_338SMB05_5
HEL_AU_Baden_42VV03_4
HEL_ES_Menorca_329PV02_7
HEL_FR_StChillian_229PV06
HEL_ES_LinasdeBroto_335PV02_1
HEL_ES_LinasdeBroto_335PV02_9
HEL_ES_Antequera_08VV02_2
HIB_ES_Fondon_12PV05_1
HEL_FR_Pirineos_338PV02_7
HEL_FR_Pirineos_338PV02_1
HIB_IE_Cashel_180PV10_1
HIB_IE_Cashel_180PV10_5
HIB_IE_Glengarriff_177PV10_1
HIB_IE_TorcWaterfall_171PV10_5
HIB_IE_Crookstown_170PV10
HEL_ES_Antequera_08VV02_12
HEL_ES_Santona_125PV01_5
HIB_ES_Bollacin_127PV01_10
HIB_ES_BeniadeOnis_412PV00
HIB_ES_Bollacin_127PV01_3
HIB_ES_Angeiras_221OF00
HIB_IE_Glengarriff_177PV10_5
HIB_ES_Penarrubia_4615_6
HIB_ES_Penarrubia_4615_2
HIB_ES_SierradoCaramulo_336PR
HIB_ES_Penarrubia_4615_4
HIB_ES_Penarrubia_4615_7
HIB_ES_Penarrubia_4615_8
HIB_ES_Piedralaves_02VV18_1
HEL_ES_Santona_125PV01_10
HIB_ES_Piedralaves_02VV18_10
HIB_ES_SierraEstrela_220OF00
IBE_PT_Villuercas_03VV18_4
IBE_PT_Villuercas_03VV18_5
IBE_PT_Villuercas_03VV18_2
IBE_PT_Villuercas_03VV18_1
IBE_PT_Villuercas_03VV18_3
IBE_ES_Fuenteheridos_11VV18_7
IBE_ES_Fuenteheridos_11VV18_5
IBE_ES_Fuenteheridos_11VV18_10
IBE_ES_Fuenteheridos_11VV18_1
IBE_ES_Fuenteheridos_11VV18_3
IBE_PT_Arrabida_04VV18_1
IBE_ES_MonteradelTorero_10VV18_8
IBE_ES_MonteradelTorero_10VV18_6
IBE_ES_MonteradelTorero_10VV18_5
IBE_ES_MonteradelTorero_10VV18_1
IBE_ES_MonteradelTorero_10VV18_3
IBE_PT_SaoMArcosAlferce_06VV18
IBE_PT_CanutoSPicoFoia_08VV18_5
IBE_PT_MonchiqueSPicoFoia_07VV18
IBE_PT_CanutoSPicoFoia_08VV18_1
IBE_PT_PicoFoia_09VV18_1
IBE_PT_PicoFoia_09VV18_2
IBE_PT_Arrabida_04VV18_4
IBE_PT_Arrabida_04VV18_9
IBE_PT_Arrabida_04VV18_2
IBE_PT_Arrabida_04VV18_7
MAD_PT_SaoJorgeSaoVicente_04VV08_2
MAD_PT_DasQueimadas_03VV08_3
MAD_PT_FaialSantana_01VV08_3
MAD_PT_Funchal_9818MV
MAD_PT_FaialSantana_01VV08_1
MAD_PT_FaialSantana_01VV08_2
MAD_PT_Santa_05VV08_5
MAD_PT_Santa_05VV08_3
MAD_PT_SaoJorgeSaoVicente_04VV08_4
MAD_PT_DasQueimadas_03VV08_1
MAD_PT_Santa_05VV08_2
MAD_PT_DasQueimadas_03VV08_2
MAD_PT_Funchal_3394CN
MAD_PT_SaoJorgeSaoVicente_04VV08_1
MAD_PT_SaoJorgeSaoVicente_04VV08_3
MAD_PT_Santa_05VV08_4
MAD_PT_Santa_05VV08_6
MAD_PT_CaboPontadoPargo_3
MAD_PT_CaboPontadoPargo_4
MAD_PT_CaboPontadoPargo_1
MAD_PT_CaboPontadoPargo_2
c90m4p2r1 (RAxML)
0.002

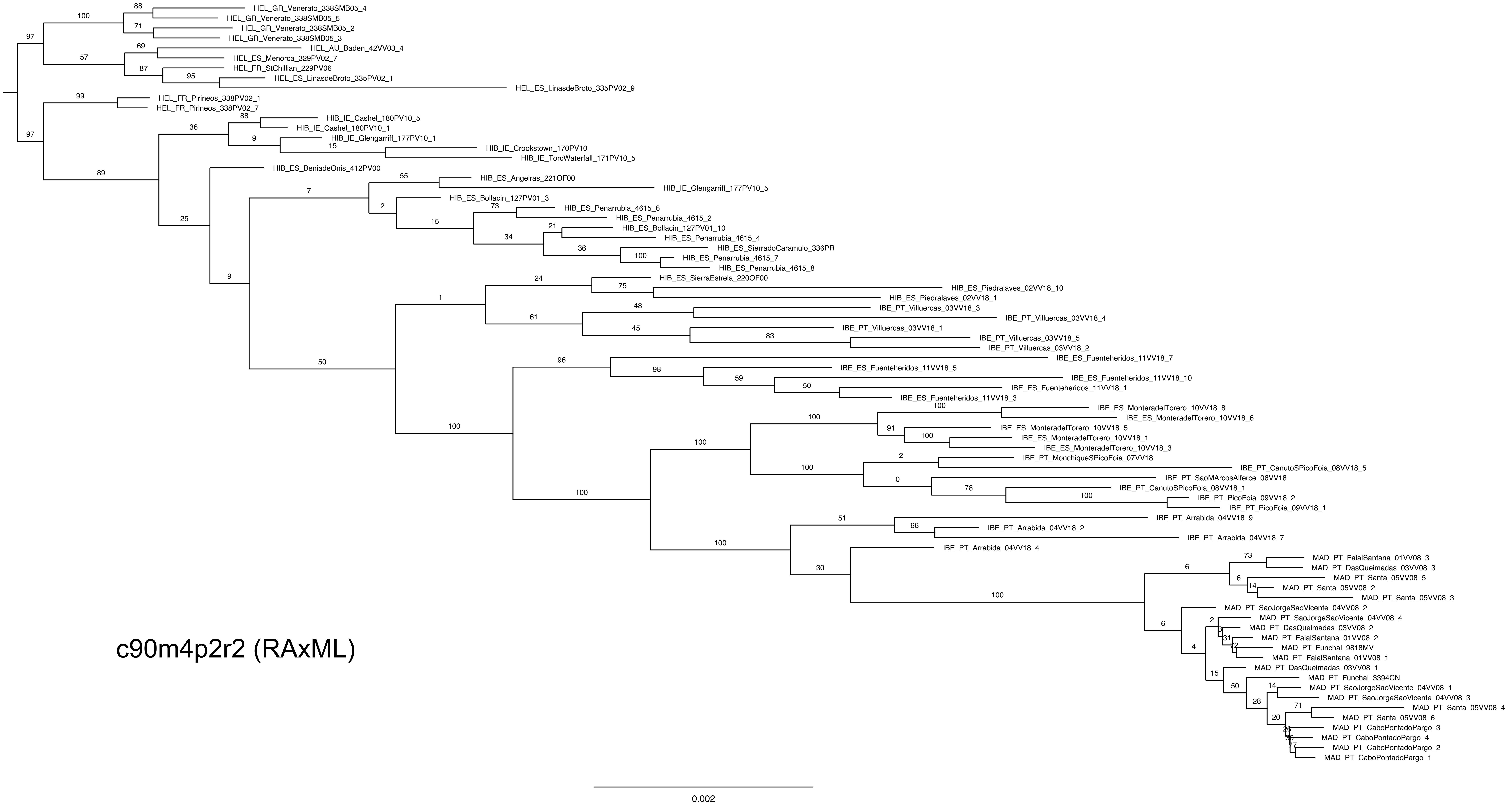
HEL_GR_Venerato_338SMB05_4
HEL_GR_Venerato_338SMB05_5
HEL_GR_Venerato_338SMB05_2
HEL_GR_Venerato_338SMB05_3
HEL_AU_Baden_42VV03_4
HEL_ES_Menorca_329PV02_7
HEL_FR_StChillian_229PV06
HEL_ES_LinasdeBroto_335PV02_1
HEL_ES_LinasdeBroto_335PV02_9
HEL_FR_Pirineos_338PV02_1
HEL_FR_Pirineos_338PV02_7
HIB_IE_Cashel_180PV10_5
HIB_IE_Cashel_180PV10_1
HIB_IE_Glengarriff_177PV10_1
HIB_IE_Crookstown_170PV10
HIB_IE_TorcWaterfall_171PV10_5
HIB_ES_BeniadeOnis_412PV00
HIB_ES_Angeiras_221OF00
HIB_IE_Glengarriff_177PV10_5
HIB_ES_Bollacin_127PV01_3
HIB_ES_Penarrubia_4615_6
HIB_ES_Penarrubia_4615_2
HIB_ES_Bollacin_127PV01_10
HIB_ES_Penarrubia_4615_4
HIB_ES_SierradoCaramulo_336PR
HIB_ES_Penarrubia_4615_7
HIB_ES_Penarrubia_4615_8
HIB_ES_SierraEstrela_220OF00
HIB_ES_Piedralaves_02VV18_10
HIB_ES_Piedralaves_02VV18_1
IBE_PT_Villuercas_03VV18_3
IBE_PT_Villuercas_03VV18_4
IBE_PT_Villuercas_03VV18_1
IBE_PT_Villuercas_03VV18_5
IBE_PT_Villuercas_03VV18_2
IBE_ES_Fuenteheridos_11VV18_7
IBE_ES_Fuenteheridos_11VV18_5
IBE_ES_Fuenteheridos_11VV18_10
IBE_ES_Fuenteheridos_11VV18_1
IBE_ES_Fuenteheridos_11VV18_3
IBE_ES_MonteradelTorero_10VV18_8
IBE_ES_MonteradelTorero_10VV18_6
IBE_ES_MonteradelTorero_10VV18_5
IBE_ES_MonteradelTorero_10VV18_1
IBE_ES_MonteradelTorero_10VV18_3
IBE_PT_MonchiqueSPicoFoia_07VV18
IBE_PT_CanutoSPicoFoia_08VV18_5
IBE_PT_SaoMArcosAlferce_06VV18
IBE_PT_CanutoSPicoFoia_08VV18_1
IBE_PT_PicoFoia_09VV18_2
IBE_PT_PicoFoia_09VV18_1
IBE_PT_Arrabida_04VV18_9
IBE_PT_Arrabida_04VV18_2
IBE_PT_Arrabida_04VV18_7
IBE_PT_Arrabida_04VV18_4
MAD_PT_FaialSantana_01VV08_3
MAD_PT_DasQueimadas_03VV08_3
MAD_PT_Santa_05VV08_5
MAD_PT_Santa_05VV08_2
MAD_PT_Santa_05VV08_3
MAD_PT_SaoJorgeSaoVicente_04VV08_2
MAD_PT_SaoJorgeSaoVicente_04VV08_4
MAD_PT_DasQueimadas_03VV08_2
MAD_PT_FaialSantana_01VV08_2
MAD_PT_Funchal_9818MV
MAD_PT_FaialSantana_01VV08_1
MAD_PT_DasQueimadas_03VV08_1
MAD_PT_Funchal_3394CN
MAD_PT_SaoJorgeSaoVicente_04VV08_1
MAD_PT_SaoJorgeSaoVicente_04VV08_3
MAD_PT_Santa_05VV08_4
MAD_PT_Santa_05VV08_6
MAD_PT_CaboPontadoPargo_3
MAD_PT_CaboPontadoPargo_4
MAD_PT_CaboPontadoPargo_2
MAD_PT_CaboPontadoPargo_1
c90m4p2r2 (RAxML)
0.002

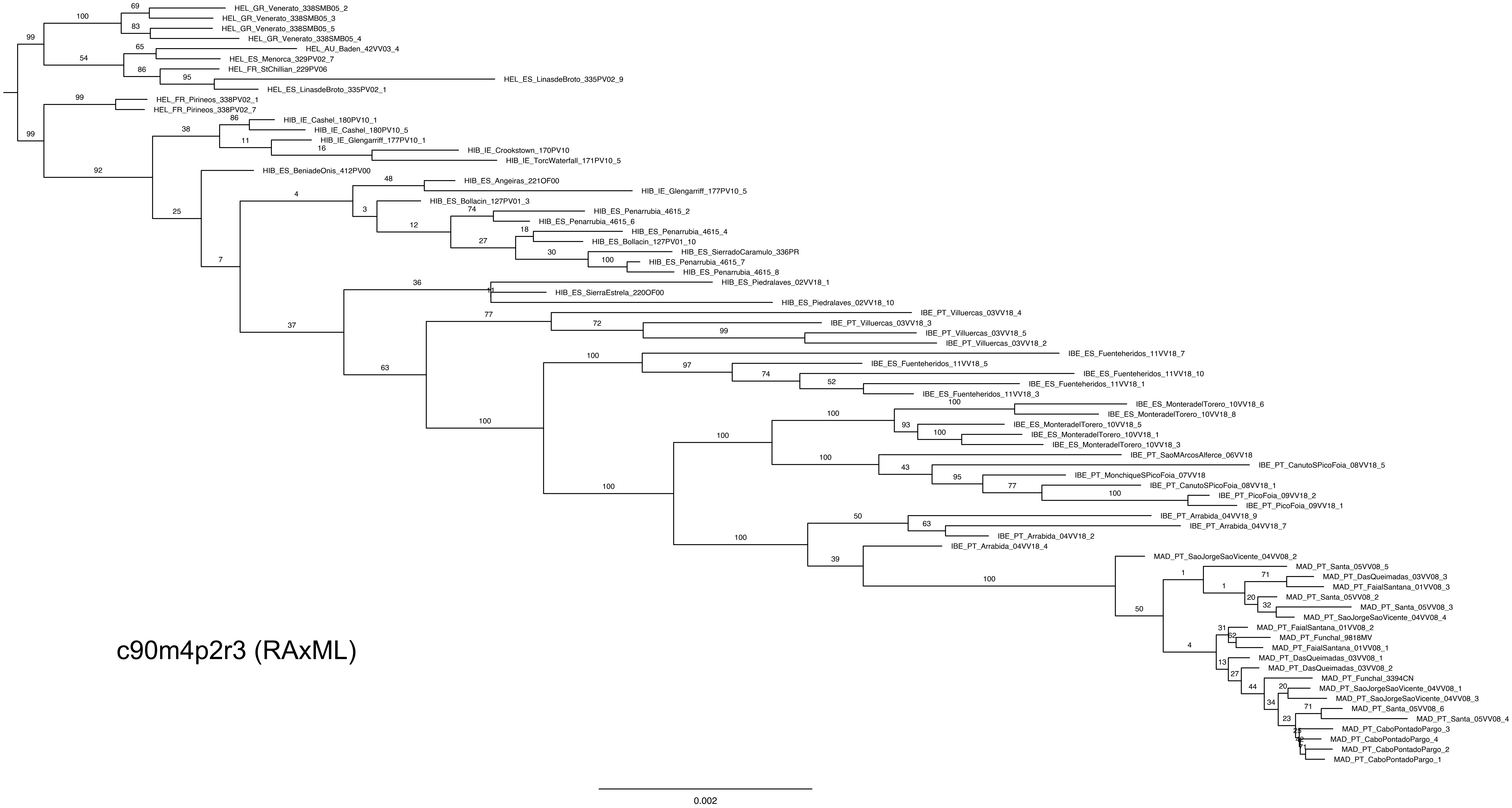

c90m4p2r3 (RAxML)

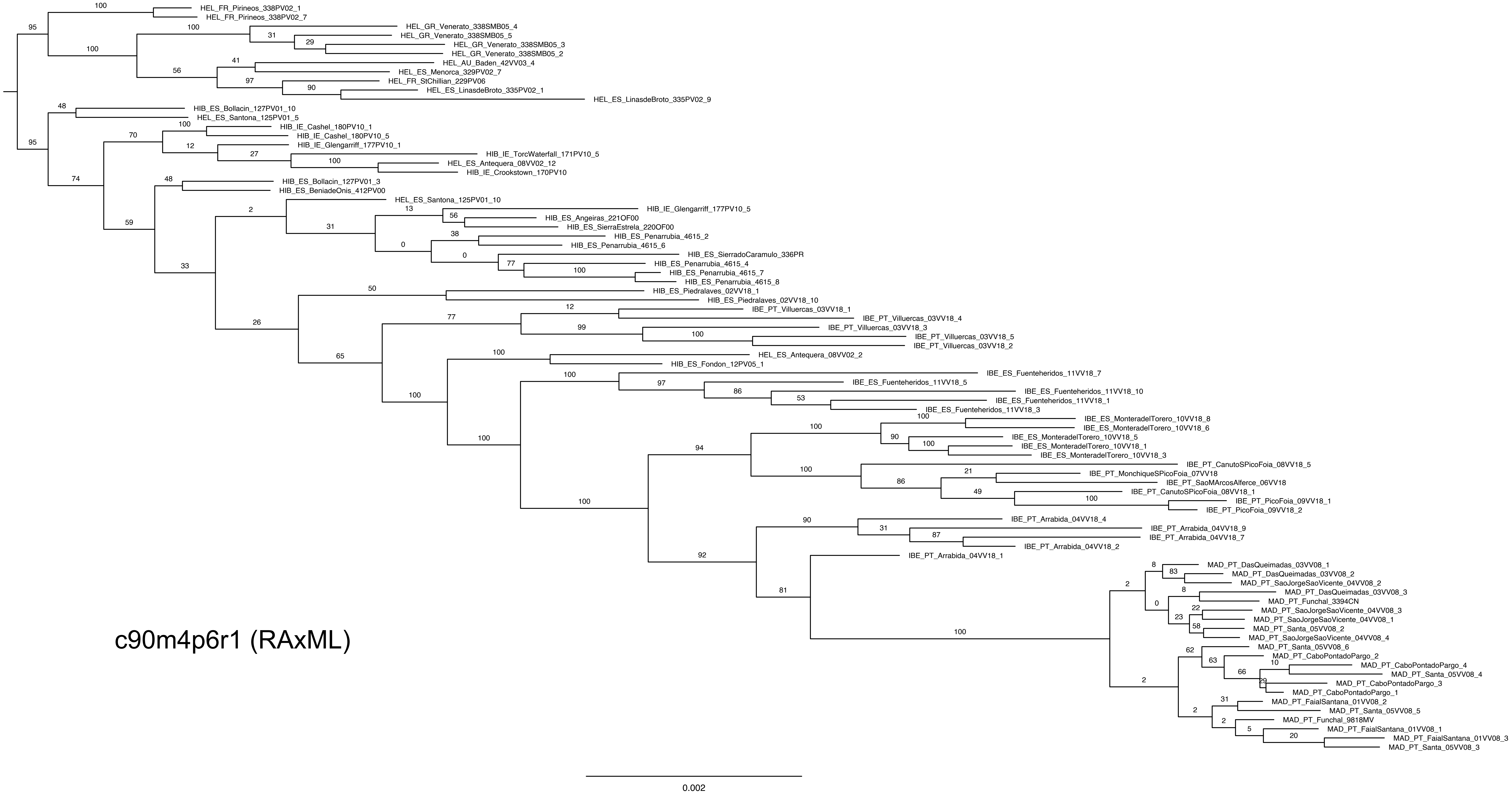
HEL_FR_Pirineos_338PV02_1
HEL_FR_Pirineos_338PV02_7
HEL_GR_Venerato_338SMB05_4
HEL_GR_Venerato_338SMB05_5
HEL_GR_Venerato_338SMB05_3
HEL_GR_Venerato_338SMB05_2
HEL_AU_Baden_42VV03_4
HEL_ES_Menorca_329PV02_7
HEL_FR_StChillian_229PV06
HEL_ES_LinasdeBroto_335PV02_1
HEL_ES_LinasdeBroto_335PV02_9
HIB_ES_Bollacin_127PV01_10
HEL_ES_Santona_125PV01_5
HIB_IE_Cashel_180PV10_1
HIB_IE_Cashel_180PV10_5
HIB_IE_Glengarriff_177PV10_1
HIB_IE_TorcWaterfall_171PV10_5
HEL_ES_Antequera_08VV02_12
HIB_IE_Crookstown_170PV10
HIB_ES_Bollacin_127PV01_3
HIB_ES_BeniadeOnis_412PV00
HEL_ES_Santona_125PV01_10
HIB_IE_Glengarriff_177PV10_5
HIB_ES_Angeiras_221OF00
HIB_ES_SierraEstrela_220OF00
HIB_ES_Penarrubia_4615_2
HIB_ES_Penarrubia_4615_6
HIB_ES_SierradoCaramulo_336PR
HIB_ES_Penarrubia_4615_4
HIB_ES_Penarrubia_4615_7
HIB_ES_Penarrubia_4615_8
HIB_ES_Piedralaves_02VV18_1
HIB_ES_Piedralaves_02VV18_10
IBE_PT_Villuercas_03VV18_1
IBE_PT_Villuercas_03VV18_4
IBE_PT_Villuercas_03VV18_3
IBE_PT_Villuercas_03VV18_5
IBE_PT_Villuercas_03VV18_2
HEL_ES_Antequera_08VV02_2
HIB_ES_Fondon_12PV05_1
IBE_ES_Fuenteheridos_11VV18_7
IBE_ES_Fuenteheridos_11VV18_5
IBE_ES_Fuenteheridos_11VV18_10
IBE_ES_Fuenteheridos_11VV18_1
IBE_ES_Fuenteheridos_11VV18_3
IBE_ES_MonteradelTorero_10VV18_8
IBE_ES_MonteradelTorero_10VV18_6
IBE_ES_MonteradelTorero_10VV18_5
IBE_ES_MonteradelTorero_10VV18_1
IBE_ES_MonteradelTorero_10VV18_3
IBE_PT_CanutoSPicoFoia_08VV18_5
IBE_PT_MonchiqueSPicoFoia_07VV18
IBE_PT_SaoMArcosAlferce_06VV18
IBE_PT_CanutoSPicoFoia_08VV18_1
IBE_PT_PicoFoia_09VV18_1
IBE_PT_PicoFoia_09VV18_2
IBE_PT_Arrabida_04VV18_4
IBE_PT_Arrabida_04VV18_9
IBE_PT_Arrabida_04VV18_7
IBE_PT_Arrabida_04VV18_2
IBE_PT_Arrabida_04VV18_1
MAD_PT_DasQueimadas_03VV08_1
MAD_PT_DasQueimadas_03VV08_2
MAD_PT_SaoJorgeSaoVicente_04VV08_2
MAD_PT_DasQueimadas_03VV08_3
MAD_PT_Funchal_3394CN
MAD_PT_SaoJorgeSaoVicente_04VV08_3
MAD_PT_SaoJorgeSaoVicente_04VV08_1
MAD_PT_Santa_05VV08_2
MAD_PT_SaoJorgeSaoVicente_04VV08_4
MAD_PT_Santa_05VV08_6
MAD_PT_CaboPontadoPargo_2
MAD_PT_CaboPontadoPargo_4
MAD_PT_Santa_05VV08_4
MAD_PT_CaboPontadoPargo_3
MAD_PT_CaboPontadoPargo_1
MAD_PT_FaialSantana_01VV08_2
MAD_PT_Santa_05VV08_5
MAD_PT_Funchal_9818MV
MAD_PT_FaialSantana_01VV08_1
MAD_PT_FaialSantana_01VV08_3
MAD_PT_Santa_05VV08_3
c90m4p6r1 (RAxML)
0.002

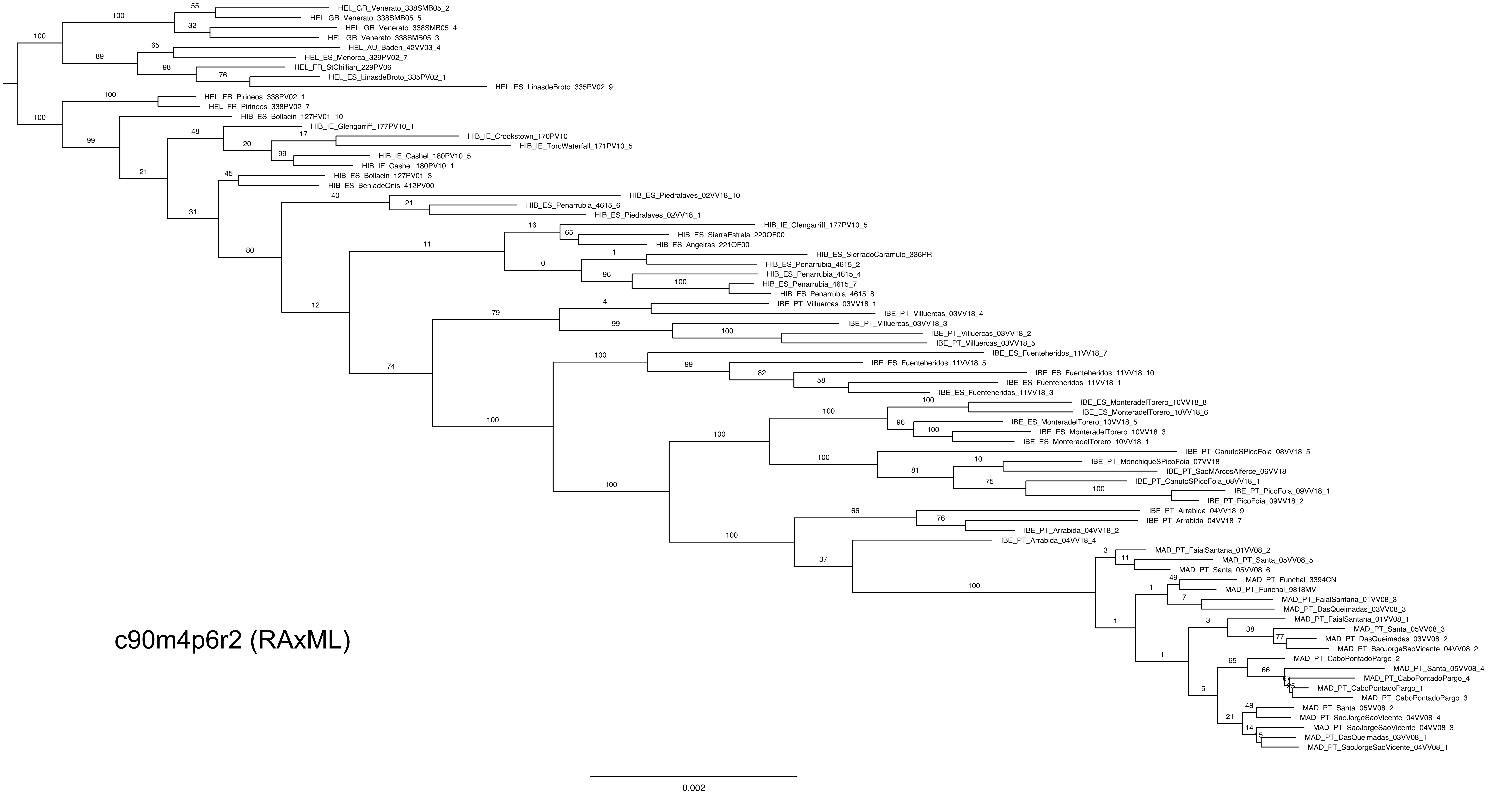
HEL_GR_Venerato_338SMB05_2
HEL_GR_Venerato_338SMB05_5
HEL_GR_Venerato_338SMB05_4
HEL_GR_Venerato_338SMB05_3
HEL_AU_Baden_42VV03_4
HEL_ES_Menorca_329PV02_7
HEL_FR_StChillian_229PV06
HEL_ES_LinasdeBroto_335PV02_1
HEL_ES_LinasdeBroto_335PV02_9
HEL_FR_Pirineos_338PV02_1
HEL_FR_Pirineos_338PV02_7
HIB_ES_Bollacin_127PV01_10
HIB_IE_Glengarriff_177PV10_1
HIB_IE_Crookstown_170PV10
HIB_IE_TorcWaterfall_171PV10_5
HIB_IE_Cashel_180PV10_5
HIB_IE_Cashel_180PV10_1
HIB_ES_Bollacin_127PV01_3
HIB_ES_BeniadeOnis_412PV00
HIB_ES_Piedralaves_02VV18_10
HIB_ES_Penarrubia_4615_6
HIB_ES_Piedralaves_02VV18_1
HIB_IE_Glengarriff_177PV10_5
HIB_ES_SierraEstrela_220OF00
HIB_ES_Angeiras_221OF00
HIB_ES_SierradoCaramulo_336PR
HIB_ES_Penarrubia_4615_2
HIB_ES_Penarrubia_4615_4
HIB_ES_Penarrubia_4615_7
HIB_ES_Penarrubia_4615_8
IBE_PT_Villuercas_03VV18_1
IBE_PT_Villuercas_03VV18_4
IBE_PT_Villuercas_03VV18_3
IBE_PT_Villuercas_03VV18_2
IBE_PT_Villuercas_03VV18_5
IBE_ES_Fuenteheridos_11VV18_7
IBE_ES_Fuenteheridos_11VV18_5
IBE_ES_Fuenteheridos_11VV18_10
IBE_ES_Fuenteheridos_11VV18_1
IBE_ES_Fuenteheridos_11VV18_3
IBE_ES_MonteradelTorero_10VV18_8
IBE_ES_MonteradelTorero_10VV18_6
IBE_ES_MonteradelTorero_10VV18_5
IBE_ES_MonteradelTorero_10VV18_3
IBE_ES_MonteradelTorero_10VV18_1
IBE_PT_CanutoSPicoFoia_08VV18_5
IBE_PT_MonchiqueSPicoFoia_07VV18
IBE_PT_SaoMArcosAlferce_06VV18
IBE_PT_CanutoSPicoFoia_08VV18_1
IBE_PT_PicoFoia_09VV18_1
IBE_PT_PicoFoia_09VV18_2
IBE_PT_Arrabida_04VV18_9
IBE_PT_Arrabida_04VV18_7
IBE_PT_Arrabida_04VV18_2
IBE_PT_Arrabida_04VV18_4
MAD_PT_FaialSantana_01VV08_2
MAD_PT_Santa_05VV08_5
MAD_PT_Santa_05VV08_6
MAD_PT_Funchal_3394CN
MAD_PT_Funchal_9818MV
MAD_PT_FaialSantana_01VV08_3
MAD_PT_DasQueimadas_03VV08_3
MAD_PT_FaialSantana_01VV08_1
MAD_PT_Santa_05VV08_3
MAD_PT_DasQueimadas_03VV08_2
MAD_PT_SaoJorgeSaoVicente_04VV08_2
MAD_PT_CaboPontadoPargo_2
MAD_PT_Santa_05VV08_4
MAD_PT_CaboPontadoPargo_4
MAD_PT_CaboPontadoPargo_1
MAD_PT_CaboPontadoPargo_3
MAD_PT_Santa_05VV08_2
MAD_PT_SaoJorgeSaoVicente_04VV08_4
MAD_PT_SaoJorgeSaoVicente_04VV08_3
MAD_PT_DasQueimadas_03VV08_1
MAD_PT_SaoJorgeSaoVicente_04VV08_1
c90m4p6r2 (RAxML)
0.002

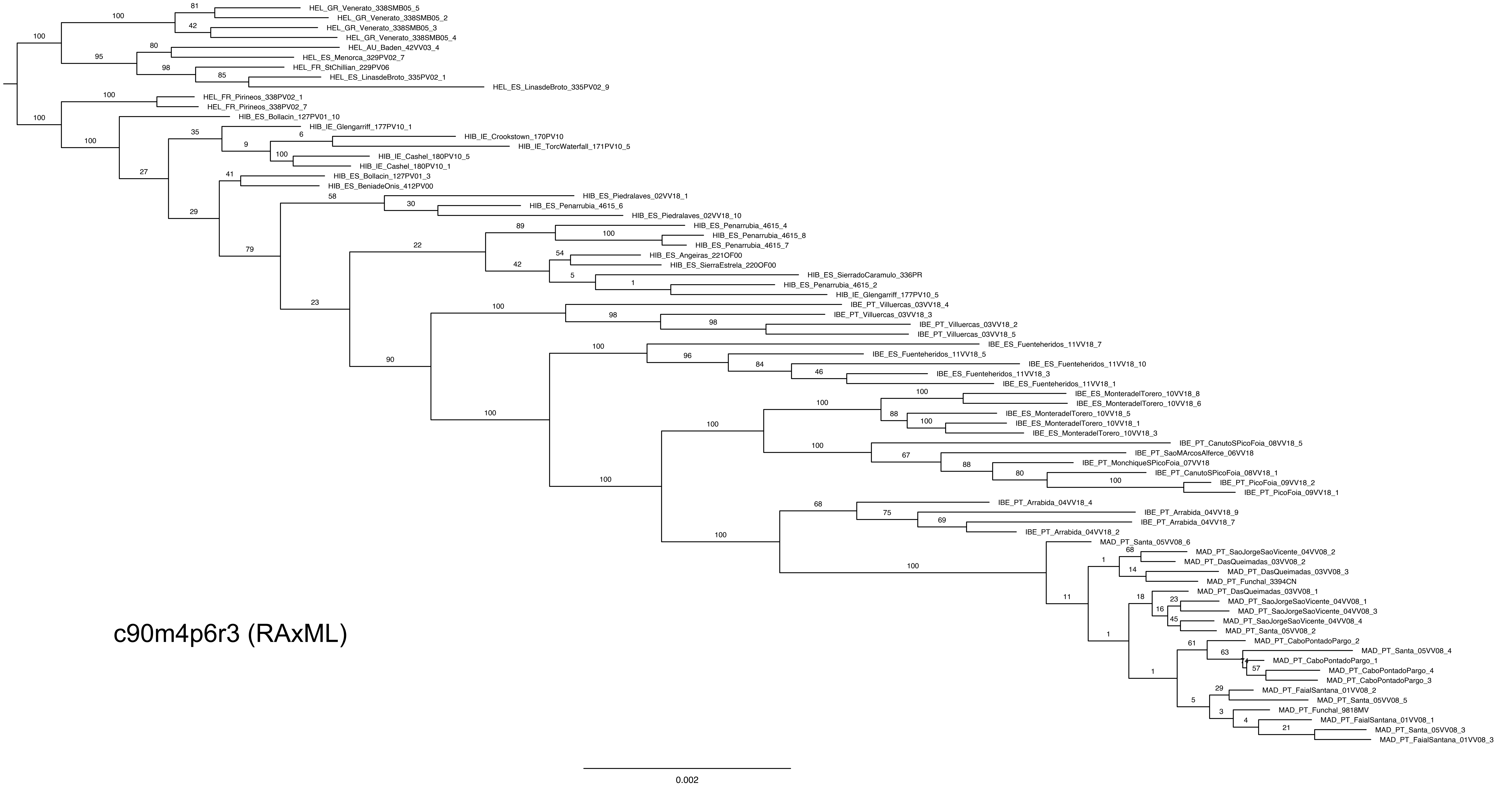
HEL_GR_Venerato_338SMB05_5
HEL_GR_Venerato_338SMB05_2
HEL_GR_Venerato_338SMB05_3
HEL_GR_Venerato_338SMB05_4
HEL_AU_Baden_42VV03_4
HEL_ES_Menorca_329PV02_7
HEL_FR_StChillian_229PV06
HEL_ES_LinasdeBroto_335PV02_1
HEL_ES_LinasdeBroto_335PV02_9
HEL_FR_Pirineos_338PV02_1
HEL_FR_Pirineos_338PV02_7
HIB_ES_Bollacin_127PV01_10
HIB_IE_Glengarriff_177PV10_1
HIB_IE_Crookstown_170PV10
HIB_IE_TorcWaterfall_171PV10_5
HIB_IE_Cashel_180PV10_5
HIB_IE_Cashel_180PV10_1
HIB_ES_Bollacin_127PV01_3
HIB_ES_BeniadeOnis_412PV00
HIB_ES_Piedralaves_02VV18_1
HIB_ES_Penarrubia_4615_6
HIB_ES_Piedralaves_02VV18_10
HIB_ES_Penarrubia_4615_4
HIB_ES_Penarrubia_4615_8
HIB_ES_Penarrubia_4615_7
HIB_ES_Angeiras_221OF00
HIB_ES_SierraEstrela_220OF00
HIB_ES_SierradoCaramulo_336PR
HIB_ES_Penarrubia_4615_2
HIB_IE_Glengarriff_177PV10_5
IBE_PT_Villuercas_03VV18_4
IBE_PT_Villuercas_03VV18_3
IBE_PT_Villuercas_03VV18_2
IBE_PT_Villuercas_03VV18_5
IBE_ES_Fuenteheridos_11VV18_7
IBE_ES_Fuenteheridos_11VV18_5
IBE_ES_Fuenteheridos_11VV18_10
IBE_ES_Fuenteheridos_11VV18_3
IBE_ES_Fuenteheridos_11VV18_1
IBE_ES_MonteradelTorero_10VV18_8
IBE_ES_MonteradelTorero_10VV18_6
IBE_ES_MonteradelTorero_10VV18_5
IBE_ES_MonteradelTorero_10VV18_1
IBE_ES_MonteradelTorero_10VV18_3
IBE_PT_CanutoSPicoFoia_08VV18_5
IBE_PT_SaoMArcosAlferce_06VV18
IBE_PT_MonchiqueSPicoFoia_07VV18
IBE_PT_CanutoSPicoFoia_08VV18_1
IBE_PT_PicoFoia_09VV18_2
IBE_PT_PicoFoia_09VV18_1
IBE_PT_Arrabida_04VV18_4
IBE_PT_Arrabida_04VV18_9
IBE_PT_Arrabida_04VV18_7
IBE_PT_Arrabida_04VV18_2
MAD_PT_Santa_05VV08_6
MAD_PT_SaoJorgeSaoVicente_04VV08_2
MAD_PT_DasQueimadas_03VV08_2
MAD_PT_DasQueimadas_03VV08_3
MAD_PT_Funchal_3394CN
MAD_PT_DasQueimadas_03VV08_1
MAD_PT_SaoJorgeSaoVicente_04VV08_1
MAD_PT_SaoJorgeSaoVicente_04VV08_3
MAD_PT_SaoJorgeSaoVicente_04VV08_4
MAD_PT_Santa_05VV08_2
MAD_PT_CaboPontadoPargo_2
MAD_PT_Santa_05VV08_4
MAD_PT_CaboPontadoPargo_1
MAD_PT_CaboPontadoPargo_4
MAD_PT_CaboPontadoPargo_3
MAD_PT_FaialSantana_01VV08_2
MAD_PT_Santa_05VV08_5
MAD_PT_Funchal_9818MV
MAD_PT_FaialSantana_01VV08_1
MAD_PT_Santa_05VV08_3
MAD_PT_FaialSantana_01VV08_3
c90m4p6r3 (RAxML)
0.002

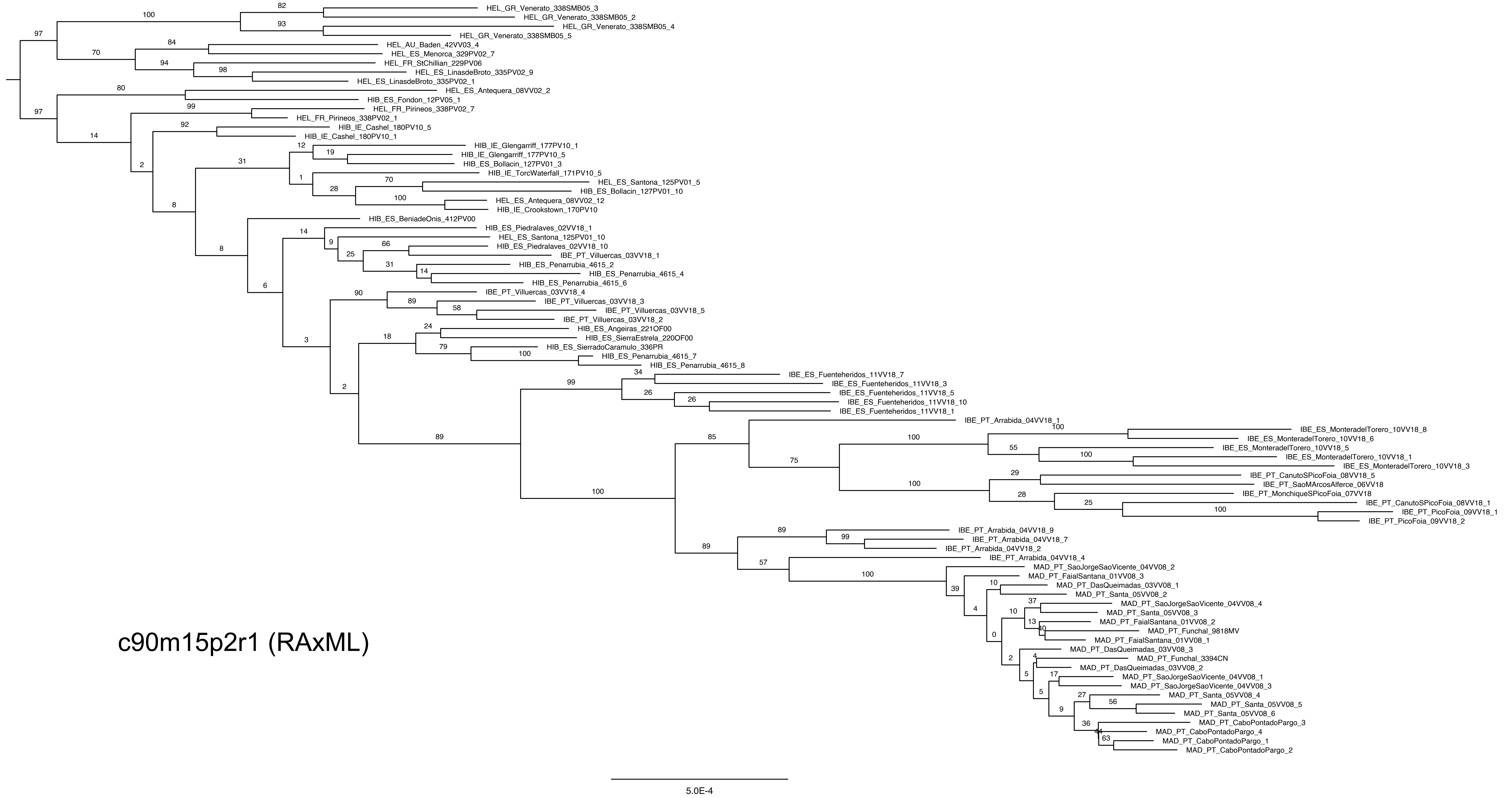
HEL_GR_Venerato_338SMB05_3
HEL_GR_Venerato_338SMB05_2
HEL_GR_Venerato_338SMB05_4
HEL_GR_Venerato_338SMB05_5
HEL_AU_Baden_42VV03_4
HEL_ES_Menorca_329PV02_7
HEL_FR_StChillian_229PV06
HEL_ES_LinasdeBroto_335PV02_9
HEL_ES_LinasdeBroto_335PV02_1
HEL_ES_Antequera_08VV02_2
HIB_ES_Fondon_12PV05_1
HEL_FR_Pirineos_338PV02_7
HEL_FR_Pirineos_338PV02_1
HIB_IE_Cashel_180PV10_5
HIB_IE_Cashel_180PV10_1
HIB_IE_Glengarriff_177PV10_1
HIB_IE_Glengarriff_177PV10_5
HIB_ES_Bollacin_127PV01_3
HIB_IE_TorcWaterfall_171PV10_5
HEL_ES_Santona_125PV01_5
HIB_ES_Bollacin_127PV01_10
HEL_ES_Antequera_08VV02_12
HIB_IE_Crookstown_170PV10
HIB_ES_BeniadeOnis_412PV00
HIB_ES_Piedralaves_02VV18_1
HEL_ES_Santona_125PV01_10
HIB_ES_Piedralaves_02VV18_10
IBE_PT_Villuercas_03VV18_1
HIB_ES_Penarrubia_4615_2
HIB_ES_Penarrubia_4615_4
HIB_ES_Penarrubia_4615_6
IBE_PT_Villuercas_03VV18_4
IBE_PT_Villuercas_03VV18_3
IBE_PT_Villuercas_03VV18_5
IBE_PT_Villuercas_03VV18_2
HIB_ES_Angeiras_221OF00
HIB_ES_SierraEstrela_220OF00
HIB_ES_SierradoCaramulo_336PR
HIB_ES_Penarrubia_4615_7
HIB_ES_Penarrubia_4615_8
IBE_ES_Fuenteheridos_11VV18_7
IBE_ES_Fuenteheridos_11VV18_3
IBE_ES_Fuenteheridos_11VV18_5
IBE_ES_Fuenteheridos_11VV18_10
IBE_ES_Fuenteheridos_11VV18_1
IBE_PT_Arrabida_04VV18_1
IBE_ES_MonteradelTorero_10VV18_8
IBE_ES_MonteradelTorero_10VV18_6
IBE_ES_MonteradelTorero_10VV18_5
IBE_ES_MonteradelTorero_10VV18_1
IBE_ES_MonteradelTorero_10VV18_3
IBE_PT_CanutoSPicoFoia_08VV18_5
IBE_PT_SaoMArcosAlferce_06VV18
IBE_PT_MonchiqueSPicoFoia_07VV18
IBE_PT_CanutoSPicoFoia_08VV18_1
IBE_PT_PicoFoia_09VV18_1
IBE_PT_PicoFoia_09VV18_2
IBE_PT_Arrabida_04VV18_9
IBE_PT_Arrabida_04VV18_7
IBE_PT_Arrabida_04VV18_2
IBE_PT_Arrabida_04VV18_4
MAD_PT_SaoJorgeSaoVicente_04VV08_2
MAD_PT_FaialSantana_01VV08_3
MAD_PT_DasQueimadas_03VV08_1
MAD_PT_Santa_05VV08_2
MAD_PT_SaoJorgeSaoVicente_04VV08_4
MAD_PT_Santa_05VV08_3
MAD_PT_FaialSantana_01VV08_2
MAD_PT_Funchal_9818MV
MAD_PT_FaialSantana_01VV08_1
MAD_PT_DasQueimadas_03VV08_3
MAD_PT_Funchal_3394CN
MAD_PT_DasQueimadas_03VV08_2
MAD_PT_SaoJorgeSaoVicente_04VV08_1
MAD_PT_SaoJorgeSaoVicente_04VV08_3
MAD_PT_Santa_05VV08_4
MAD_PT_Santa_05VV08_5
MAD_PT_Santa_05VV08_6
MAD_PT_CaboPontadoPargo_3
MAD_PT_CaboPontadoPargo_4
MAD_PT_CaboPontadoPargo_1
MAD_PT_CaboPontadoPargo_2
c90m15p2r1 (RAxML)
5.0E-4

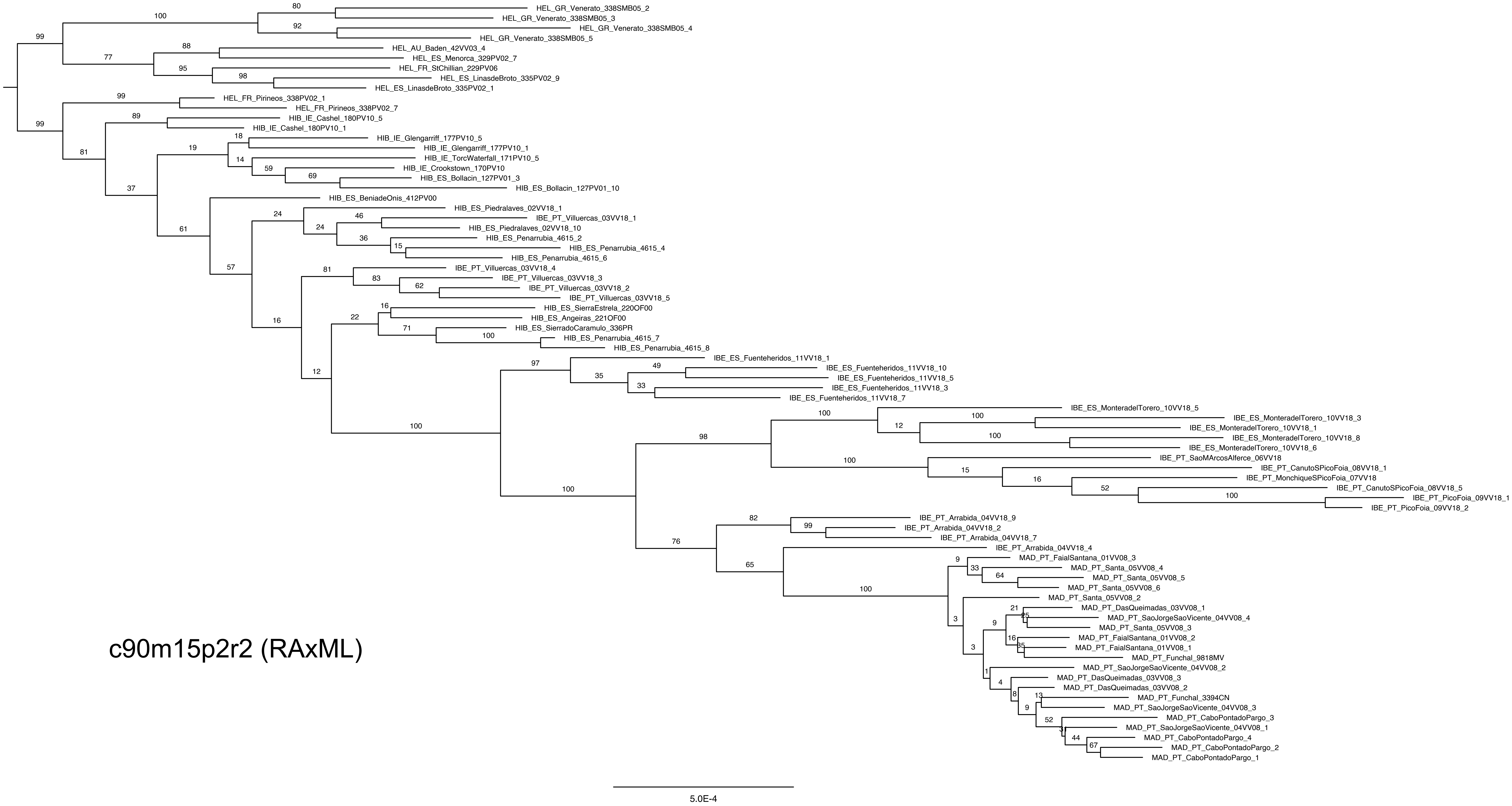
c90m15p2r2 (RAxML)
HEL_GR_Venerato_338SMB05_2
HEL_GR_Venerato_338SMB05_3
HEL_GR_Venerato_338SMB05_4
HEL_GR_Venerato_338SMB05_5
HEL_AU_Baden_42VV03_4
HEL_ES_Menorca_329PV02_7
HEL_FR_StChillian_229PV06
HEL_ES_LinasdeBroto_335PV02_9
HEL_ES_LinasdeBroto_335PV02_1
HEL_FR_Pirineos_338PV02_1
HEL_FR_Pirineos_338PV02_7
HIB_IE_Cashel_180PV10_5
HIB_IE_Cashel_180PV10_1
HIB_IE_Glengarriff_177PV10_5
HIB_IE_Glengarriff_177PV10_1
HIB_IE_TorcWaterfall_171PV10_5
HIB_IE_Crookstown_170PV10
HIB_ES_Bollacin_127PV01_3
HIB_ES_Bollacin_127PV01_10
HIB_ES_BeniadeOnis_412PV00
HIB_ES_Piedralaves_02VV18_1
IBE_PT_Villuercas_03VV18_1
HIB_ES_Piedralaves_02VV18_10
HIB_ES_Penarrubia_4615_2
HIB_ES_Penarrubia_4615_4
HIB_ES_Penarrubia_4615_6
IBE_PT_Villuercas_03VV18_4
IBE_PT_Villuercas_03VV18_3
IBE_PT_Villuercas_03VV18_2
IBE_PT_Villuercas_03VV18_5
HIB_ES_SierraEstrela_220OF00
HIB_ES_Angeiras_221OF00
HIB_ES_SierradoCaramulo_336PR
HIB_ES_Penarrubia_4615_7
HIB_ES_Penarrubia_4615_8
IBE_ES_Fuenteheridos_11VV18_1
IBE_ES_Fuenteheridos_11VV18_10
IBE_ES_Fuenteheridos_11VV18_5
IBE_ES_Fuenteheridos_11VV18_3
IBE_ES_Fuenteheridos_11VV18_7
IBE_ES_MonteradelTorero_10VV18_5
IBE_ES_MonteradelTorero_10VV18_3
IBE_ES_MonteradelTorero_10VV18_1
IBE_ES_MonteradelTorero_10VV18_8
IBE_ES_MonteradelTorero_10VV18_6
IBE_PT_SaoMArcosAlferce_06VV18
IBE_PT_CanutoSPicoFoia_08VV18_1
IBE_PT_MonchiqueSPicoFoia_07VV18
IBE_PT_CanutoSPicoFoia_08VV18_5
IBE_PT_PicoFoia_09VV18_1
IBE_PT_PicoFoia_09VV18_2
IBE_PT_Arrabida_04VV18_9
IBE_PT_Arrabida_04VV18_2
IBE_PT_Arrabida_04VV18_7
IBE_PT_Arrabida_04VV18_4
MAD_PT_FaialSantana_01VV08_3
MAD_PT_Santa_05VV08_4
MAD_PT_Santa_05VV08_5
MAD_PT_Santa_05VV08_6
MAD_PT_Santa_05VV08_2
MAD_PT_DasQueimadas_03VV08_1
MAD_PT_SaoJorgeSaoVicente_04VV08_4
MAD_PT_Santa_05VV08_3
MAD_PT_FaialSantana_01VV08_2
MAD_PT_FaialSantana_01VV08_1
MAD_PT_Funchal_9818MV
MAD_PT_SaoJorgeSaoVicente_04VV08_2
MAD_PT_DasQueimadas_03VV08_3
MAD_PT_DasQueimadas_03VV08_2
MAD_PT_Funchal_3394CN
MAD_PT_SaoJorgeSaoVicente_04VV08_3
MAD_PT_CaboPontadoPargo_3
MAD_PT_SaoJorgeSaoVicente_04VV08_1
MAD_PT_CaboPontadoPargo_4
MAD_PT_CaboPontadoPargo_2
MAD_PT_CaboPontadoPargo_1
5.0E-4

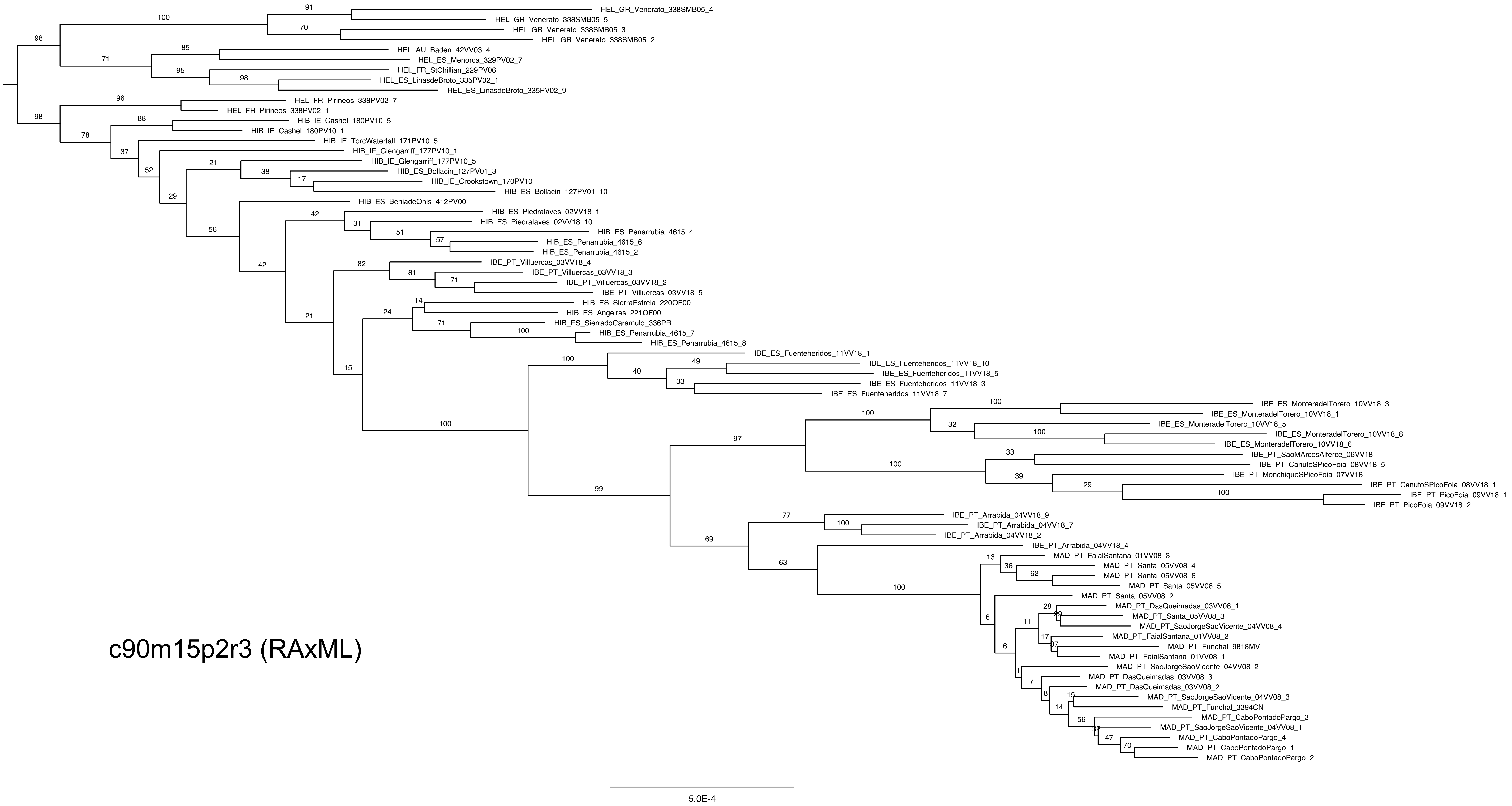

HEL_GR_Venerato_338SMB05_4
HEL_GR_Venerato_338SMB05_5
HEL_GR_Venerato_338SMB05_3
HEL_GR_Venerato_338SMB05_2
HEL_AU_Baden_42VV03_4
HEL_ES_Menorca_329PV02_7
HEL_FR_StChillian_229PV06
HEL_ES_LinasdeBroto_335PV02_1
HEL_ES_LinasdeBroto_335PV02_9
HEL_FR_Pirineos_338PV02_7
HEL_FR_Pirineos_338PV02_1
HIB_IE_Cashel_180PV10_5
HIB_IE_Cashel_180PV10_1
HIB_IE_TorcWaterfall_171PV10_5
HIB_IE_Glengarriff_177PV10_1
HIB_IE_Glengarriff_177PV10_5
HIB_ES_Bollacin_127PV01_3
HIB_IE_Crookstown_170PV10
HIB_ES_Bollacin_127PV01_10
HIB_ES_BeniadeOnis_412PV00
HIB_ES_Piedralaves_02VV18_1
HIB_ES_Piedralaves_02VV18_10
HIB_ES_Penarrubia_4615_4
HIB_ES_Penarrubia_4615_6
HIB_ES_Penarrubia_4615_2
IBE_PT_Villuercas_03VV18_4
IBE_PT_Villuercas_03VV18_3
IBE_PT_Villuercas_03VV18_2
IBE_PT_Villuercas_03VV18_5
HIB_ES_SierraEstrela_220OF00
HIB_ES_Angeiras_221OF00
HIB_ES_SierradoCaramulo_336PR
HIB_ES_Penarrubia_4615_7
HIB_ES_Penarrubia_4615_8
IBE_ES_Fuenteheridos_11VV18_1
IBE_ES_Fuenteheridos_11VV18_10
IBE_ES_Fuenteheridos_11VV18_5
IBE_ES_Fuenteheridos_11VV18_3
IBE_ES_Fuenteheridos_11VV18_7
IBE_ES_MonteradelTorero_10VV18_3
IBE_ES_MonteradelTorero_10VV18_1
IBE_ES_MonteradelTorero_10VV18_5
IBE_ES_MonteradelTorero_10VV18_8
IBE_ES_MonteradelTorero_10VV18_6
IBE_PT_SaoMArcosAlferce_06VV18
IBE_PT_CanutoSPicoFoia_08VV18_5
IBE_PT_MonchiqueSPicoFoia_07VV18
IBE_PT_CanutoSPicoFoia_08VV18_1
IBE_PT_PicoFoia_09VV18_1
IBE_PT_PicoFoia_09VV18_2
IBE_PT_Arrabida_04VV18_9
IBE_PT_Arrabida_04VV18_7
IBE_PT_Arrabida_04VV18_2
IBE_PT_Arrabida_04VV18_4
MAD_PT_FaialSantana_01VV08_3
MAD_PT_Santa_05VV08_4
MAD_PT_Santa_05VV08_6
MAD_PT_Santa_05VV08_5
MAD_PT_Santa_05VV08_2
MAD_PT_DasQueimadas_03VV08_1
MAD_PT_Santa_05VV08_3
MAD_PT_SaoJorgeSaoVicente_04VV08_4
MAD_PT_FaialSantana_01VV08_2
MAD_PT_Funchal_9818MV
MAD_PT_FaialSantana_01VV08_1
MAD_PT_SaoJorgeSaoVicente_04VV08_2
MAD_PT_DasQueimadas_03VV08_3
MAD_PT_DasQueimadas_03VV08_2
MAD_PT_SaoJorgeSaoVicente_04VV08_3
MAD_PT_Funchal_3394CN
MAD_PT_CaboPontadoPargo_3
MAD_PT_SaoJorgeSaoVicente_04VV08_1
MAD_PT_CaboPontadoPargo_4
MAD_PT_CaboPontadoPargo_1
MAD_PT_CaboPontadoPargo_2
c90m15p2r3 (RAxML)
5.0E-4

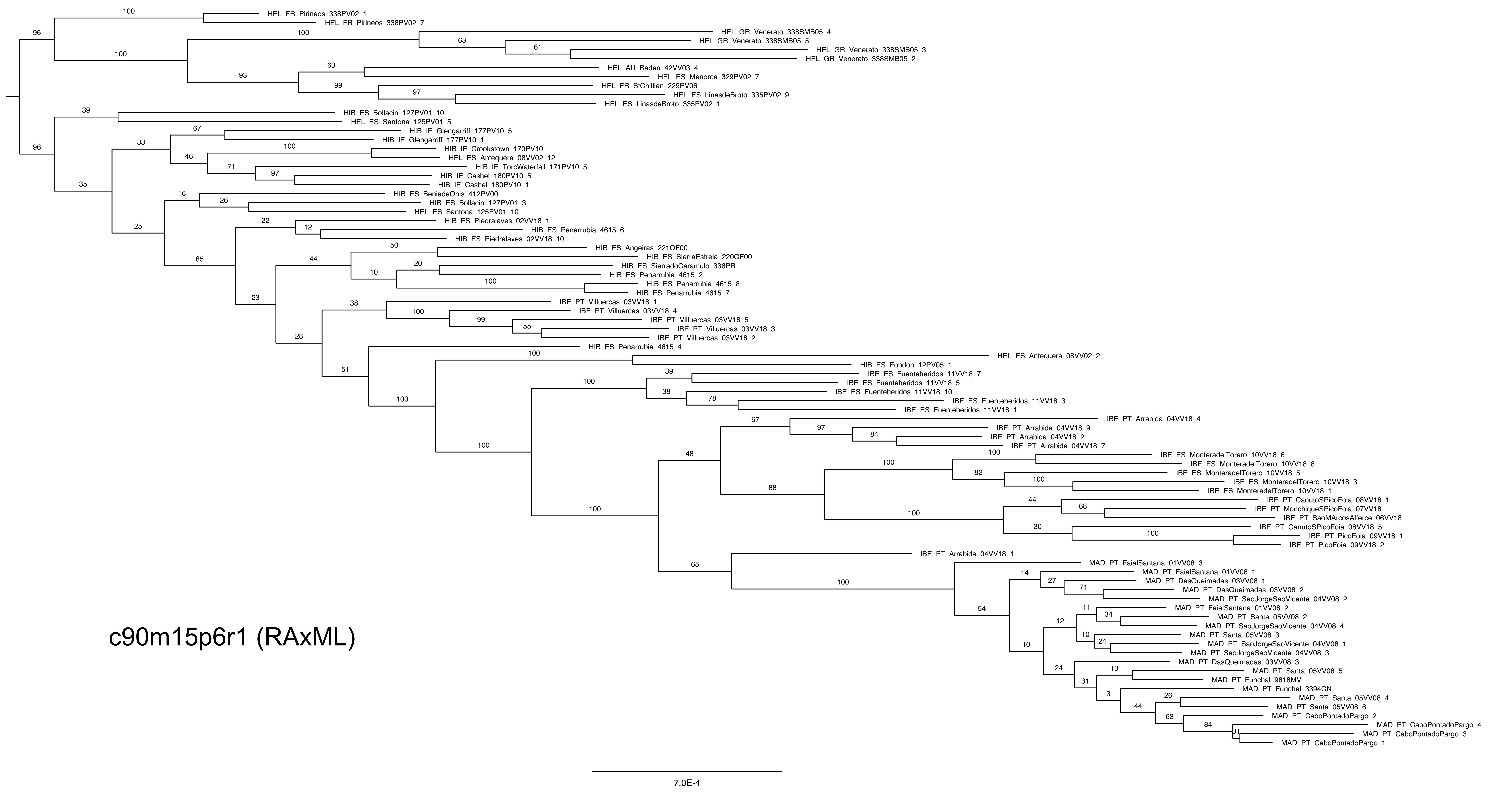
c90m15p6r1 (RAxML)
HEL_FR_Pirineos_338PV02_1
HEL_FR_Pirineos_338PV02_7
HEL_GR_Venerato_338SMB05_4
HEL_GR_Venerato_338SMB05_5
HEL_GR_Venerato_338SMB05_3
HEL_GR_Venerato_338SMB05_2
HEL_AU_Baden_42VV03_4
HEL_ES_Menorca_329PV02_7
HEL_FR_StChillian_229PV06
HEL_ES_LinasdeBroto_335PV02_9
HEL_ES_LinasdeBroto_335PV02_1
HIB_ES_Bollacin_127PV01_10
HEL_ES_Santona_125PV01_5
HIB_IE_Glengarriff_177PV10_5
HIB_IE_Glengarriff_177PV10_1
HIB_IE_Crookstown_170PV10
HEL_ES_Antequera_08VV02_12
HIB_IE_TorcWaterfall_171PV10_5
HIB_IE_Cashel_180PV10_5
HIB_IE_Cashel_180PV10_1
HIB_ES_BeniadeOnis_412PV00
HIB_ES_Bollacin_127PV01_3
HEL_ES_Santona_125PV01_10
HIB_ES_Piedralaves_02VV18_1
HIB_ES_Penarrubia_4615_6
HIB_ES_Piedralaves_02VV18_10
HIB_ES_Angeiras_221OF00
HIB_ES_SierraEstrela_220OF00
HIB_ES_SierradoCaramulo_336PR
HIB_ES_Penarrubia_4615_2
HIB_ES_Penarrubia_4615_8
HIB_ES_Penarrubia_4615_7
IBE_PT_Villuercas_03VV18_1
IBE_PT_Villuercas_03VV18_4
IBE_PT_Villuercas_03VV18_5
IBE_PT_Villuercas_03VV18_3
IBE_PT_Villuercas_03VV18_2
HIB_ES_Penarrubia_4615_4
HEL_ES_Antequera_08VV02_2
HIB_ES_Fondon_12PV05_1
IBE_ES_Fuenteheridos_11VV18_7
IBE_ES_Fuenteheridos_11VV18_5
IBE_ES_Fuenteheridos_11VV18_10
IBE_ES_Fuenteheridos_11VV18_3
IBE_ES_Fuenteheridos_11VV18_1
IBE_PT_Arrabida_04VV18_4
IBE_PT_Arrabida_04VV18_9
IBE_PT_Arrabida_04VV18_2
IBE_PT_Arrabida_04VV18_7
IBE_ES_MonteradelTorero_10VV18_6
IBE_ES_MonteradelTorero_10VV18_8
IBE_ES_MonteradelTorero_10VV18_5
IBE_ES_MonteradelTorero_10VV18_3
IBE_ES_MonteradelTorero_10VV18_1
IBE_PT_CanutoSPicoFoia_08VV18_1
IBE_PT_MonchiqueSPicoFoia_07VV18
IBE_PT_SaoMArcosAlferce_06VV18
IBE_PT_CanutoSPicoFoia_08VV18_5
IBE_PT_PicoFoia_09VV18_1
IBE_PT_PicoFoia_09VV18_2
IBE_PT_Arrabida_04VV18_1
MAD_PT_FaialSantana_01VV08_3
MAD_PT_FaialSantana_01VV08_1
MAD_PT_DasQueimadas_03VV08_1
MAD_PT_DasQueimadas_03VV08_2
MAD_PT_SaoJorgeSaoVicente_04VV08_2
MAD_PT_FaialSantana_01VV08_2
MAD_PT_Santa_05VV08_2
MAD_PT_SaoJorgeSaoVicente_04VV08_4
MAD_PT_Santa_05VV08_3
MAD_PT_SaoJorgeSaoVicente_04VV08_1
MAD_PT_SaoJorgeSaoVicente_04VV08_3
MAD_PT_DasQueimadas_03VV08_3
MAD_PT_Santa_05VV08_5
MAD_PT_Funchal_9818MV
MAD_PT_Funchal_3394CN
MAD_PT_Santa_05VV08_4
MAD_PT_Santa_05VV08_6
MAD_PT_CaboPontadoPargo_2
MAD_PT_CaboPontadoPargo_4
MAD_PT_CaboPontadoPargo_3
MAD_PT_CaboPontadoPargo_1
7.0E-4

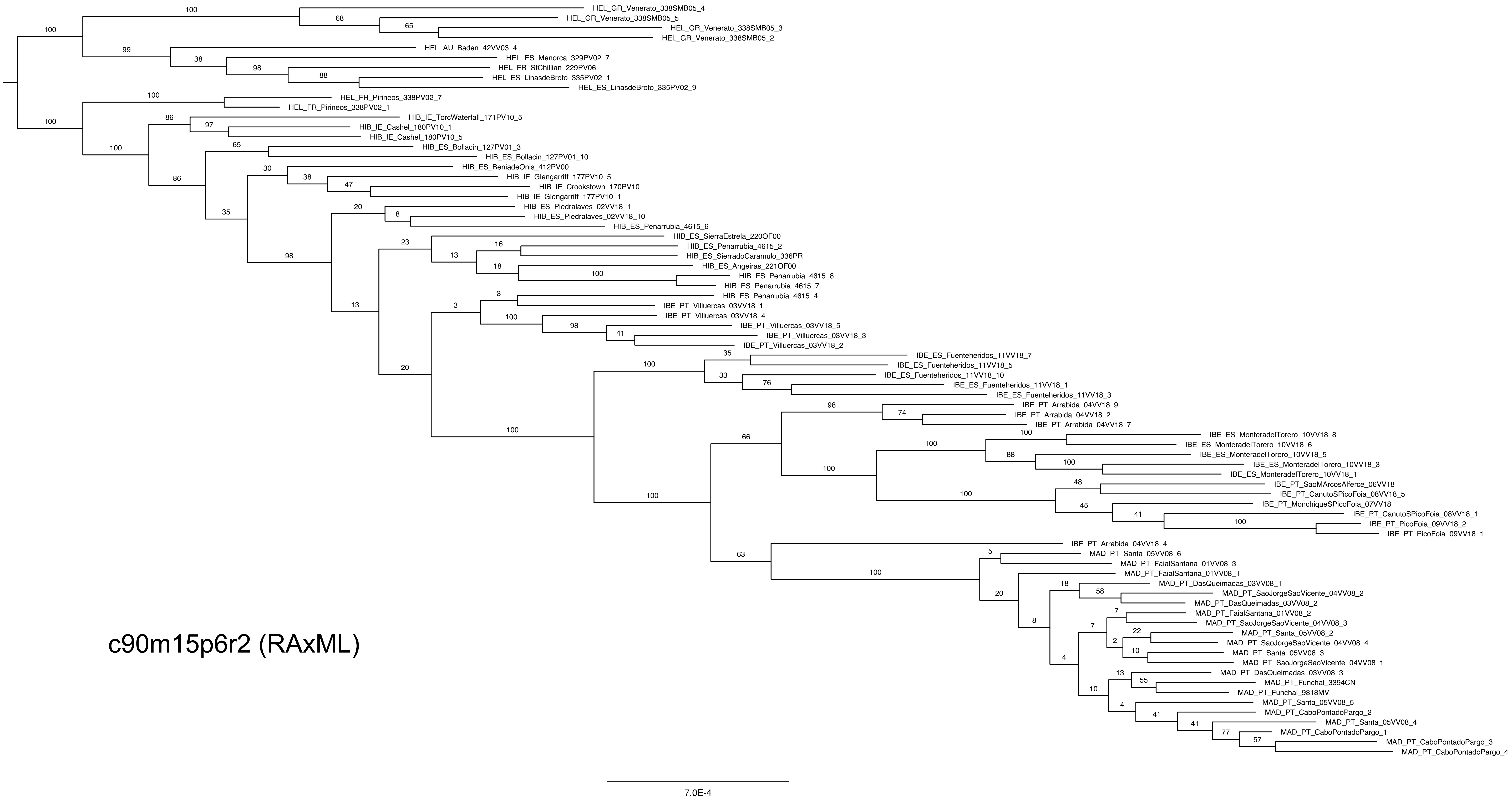
c90m15p6r2 (RAxML)
HEL_GR_Venerato_338SMB05_4
HEL_GR_Venerato_338SMB05_5
HEL_GR_Venerato_338SMB05_3
HEL_GR_Venerato_338SMB05_2
HEL_AU_Baden_42VV03_4
HEL_ES_Menorca_329PV02_7
HEL_FR_StChillian_229PV06
HEL_ES_LinasdeBroto_335PV02_1
HEL_ES_LinasdeBroto_335PV02_9
HEL_FR_Pirineos_338PV02_7
HEL_FR_Pirineos_338PV02_1
HIB_IE_TorcWaterfall_171PV10_5
HIB_IE_Cashel_180PV10_1
HIB_IE_Cashel_180PV10_5
HIB_ES_Bollacin_127PV01_3
HIB_ES_Bollacin_127PV01_10
HIB_ES_BeniadeOnis_412PV00
HIB_IE_Glengarriff_177PV10_5
HIB_IE_Crookstown_170PV10
HIB_IE_Glengarriff_177PV10_1
HIB_ES_Piedralaves_02VV18_1
HIB_ES_Piedralaves_02VV18_10
HIB_ES_Penarrubia_4615_6
HIB_ES_SierraEstrela_220OF00
HIB_ES_Penarrubia_4615_2
HIB_ES_SierradoCaramulo_336PR
HIB_ES_Angeiras_221OF00
HIB_ES_Penarrubia_4615_8
HIB_ES_Penarrubia_4615_7
HIB_ES_Penarrubia_4615_4
IBE_PT_Villuercas_03VV18_1
IBE_PT_Villuercas_03VV18_4
IBE_PT_Villuercas_03VV18_5
IBE_PT_Villuercas_03VV18_3
IBE_PT_Villuercas_03VV18_2
IBE_ES_Fuenteheridos_11VV18_7
IBE_ES_Fuenteheridos_11VV18_5
IBE_ES_Fuenteheridos_11VV18_10
IBE_ES_Fuenteheridos_11VV18_1
IBE_ES_Fuenteheridos_11VV18_3
IBE_PT_Arrabida_04VV18_9
IBE_PT_Arrabida_04VV18_2
IBE_PT_Arrabida_04VV18_7
IBE_ES_MonteradelTorero_10VV18_8
IBE_ES_MonteradelTorero_10VV18_6
IBE_ES_MonteradelTorero_10VV18_5
IBE_ES_MonteradelTorero_10VV18_3
IBE_ES_MonteradelTorero_10VV18_1
IBE_PT_SaoMArcosAlferce_06VV18
IBE_PT_CanutoSPicoFoia_08VV18_5
IBE_PT_MonchiqueSPicoFoia_07VV18
IBE_PT_CanutoSPicoFoia_08VV18_1
IBE_PT_PicoFoia_09VV18_2
IBE_PT_PicoFoia_09VV18_1
IBE_PT_Arrabida_04VV18_4
MAD_PT_Santa_05VV08_6
MAD_PT_FaialSantana_01VV08_3
MAD_PT_FaialSantana_01VV08_1
MAD_PT_DasQueimadas_03VV08_1
MAD_PT_SaoJorgeSaoVicente_04VV08_2
MAD_PT_DasQueimadas_03VV08_2
MAD_PT_FaialSantana_01VV08_2
MAD_PT_SaoJorgeSaoVicente_04VV08_3
MAD_PT_Santa_05VV08_2
MAD_PT_SaoJorgeSaoVicente_04VV08_4
MAD_PT_Santa_05VV08_3
MAD_PT_SaoJorgeSaoVicente_04VV08_1
MAD_PT_DasQueimadas_03VV08_3
MAD_PT_Funchal_3394CN
MAD_PT_Funchal_9818MV
MAD_PT_Santa_05VV08_5
MAD_PT_CaboPontadoPargo_2
MAD_PT_Santa_05VV08_4
MAD_PT_CaboPontadoPargo_1
MAD_PT_CaboPontadoPargo_3
MAD_PT_CaboPontadoPargo_4
7.0E-4

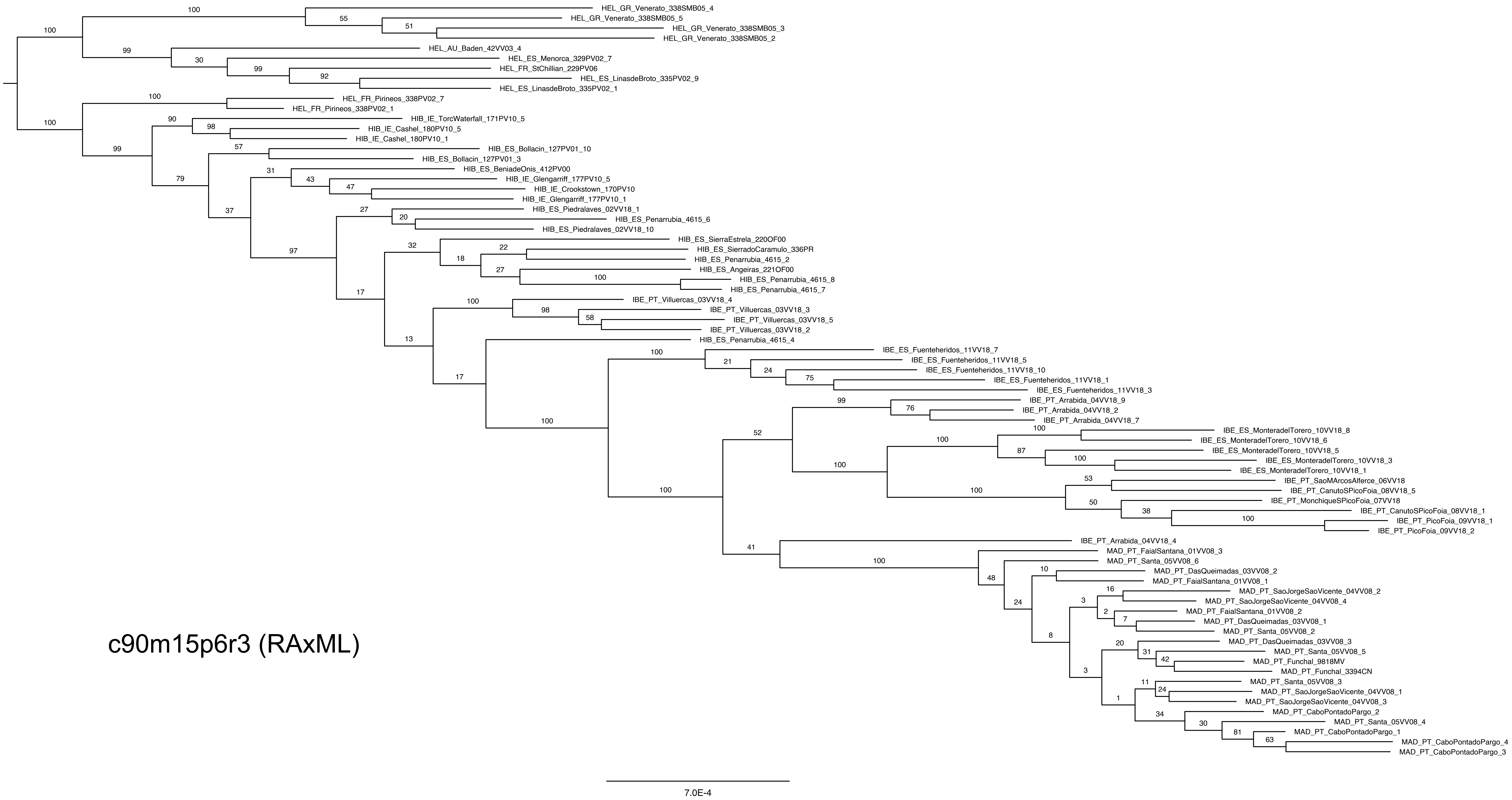

c90m15p6r3 (RAxML)

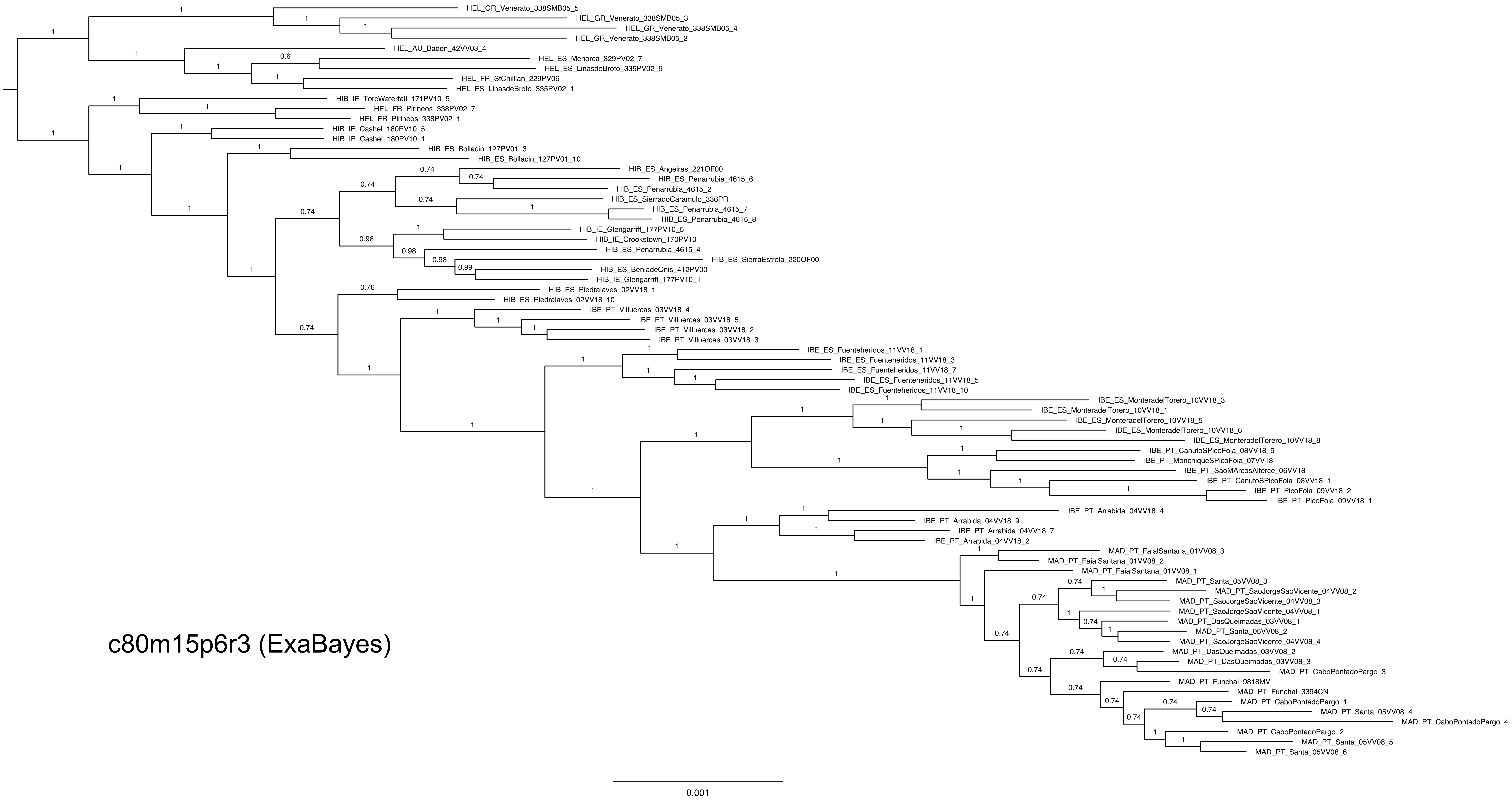

HEL_GR_Venerato_338SMB05_5
HEL_GR_Venerato_338SMB05_3
HEL_GR_Venerato_338SMB05_4
HEL_GR_Venerato_338SMB05_2
HEL_AU_Baden_42VV03_4
HEL_ES_Menorca_329PV02_7
HEL_ES_LinasdeBroto_335PV02_9
HEL_FR_StChillian_229PV06
HEL_ES_LinasdeBroto_335PV02_1
HIB_IE_TorcWaterfall_171PV10_5
HEL_FR_Pirineos_338PV02_7
HEL_FR_Pirineos_338PV02_1
HIB_IE_Cashel_180PV10_5
HIB_IE_Cashel_180PV10_1
HIB_ES_Bollacin_127PV01_3
HIB_ES_Bollacin_127PV01_10
HIB_ES_Angeiras_221OF00
HIB_ES_Penarrubia_4615_6
HIB_ES_Penarrubia_4615_2
HIB_ES_SierradoCaramulo_336PR
HIB_ES_Penarrubia_4615_7
HIB_ES_Penarrubia_4615_8
HIB_IE_Glengarriff_177PV10_5
HIB_IE_Crookstown_170PV10
HIB_ES_Penarrubia_4615_4
HIB_ES_SierraEstrela_220OF00
HIB_ES_BeniadeOnis_412PV00
HIB_IE_Glengarriff_177PV10_1
HIB_ES_Piedralaves_02VV18_1
HIB_ES_Piedralaves_02VV18_10
IBE_PT_Villuercas_03VV18_4
IBE_PT_Villuercas_03VV18_5
IBE_PT_Villuercas_03VV18_2
IBE_PT_Villuercas_03VV18_3
IBE_ES_Fuenteheridos_11VV18_1
IBE_ES_Fuenteheridos_11VV18_3
IBE_ES_Fuenteheridos_11VV18_7
IBE_ES_Fuenteheridos_11VV18_5
IBE_ES_Fuenteheridos_11VV18_10
IBE_ES_MonteradelTorero_10VV18_3
IBE_ES_MonteradelTorero_10VV18_1
IBE_ES_MonteradelTorero_10VV18_5
IBE_ES_MonteradelTorero_10VV18_6
IBE_ES_MonteradelTorero_10VV18_8
IBE_PT_CanutoSPicoFoia_08VV18_5
IBE_PT_MonchiqueSPicoFoia_07VV18
IBE_PT_SaoMArcosAlferce_06VV18
IBE_PT_CanutoSPicoFoia_08VV18_1
IBE_PT_PicoFoia_09VV18_2
IBE_PT_PicoFoia_09VV18_1
IBE_PT_Arrabida_04VV18_4
IBE_PT_Arrabida_04VV18_9
IBE_PT_Arrabida_04VV18_7
IBE_PT_Arrabida_04VV18_2
MAD_PT_FaialSantana_01VV08_3
MAD_PT_FaialSantana_01VV08_2
MAD_PT_FaialSantana_01VV08_1
MAD_PT_Santa_05VV08_3
MAD_PT_SaoJorgeSaoVicente_04VV08_2
MAD_PT_SaoJorgeSaoVicente_04VV08_3
MAD_PT_SaoJorgeSaoVicente_04VV08_1
MAD_PT_DasQueimadas_03VV08_1
MAD_PT_Santa_05VV08_2
MAD_PT_SaoJorgeSaoVicente_04VV08_4
MAD_PT_DasQueimadas_03VV08_2
MAD_PT_DasQueimadas_03VV08_3
MAD_PT_CaboPontadoPargo_3
MAD_PT_Funchal_9818MV
MAD_PT_Funchal_3394CN
MAD_PT_CaboPontadoPargo_1
MAD_PT_Santa_05VV08_4
MAD_PT_CaboPontadoPargo_4
MAD_PT_CaboPontadoPargo_2
MAD_PT_Santa_05VV08_5
MAD_PT_Santa_05VV08_6
c80m15p6r3 (ExaBayes)
0.001
